# Personalized Profiling of Lipoprotein and Lipid Metabolism Based on 1018 Measures from Combined Quantitative NMR and LC-MS/MS Platforms

**DOI:** 10.1021/acs.analchem.4c03229

**Published:** 2024-12-16

**Authors:** Siyu Zhao, Corey Giles, Kevin Huynh, Johannes Kettunen, Marjo-Riitta Järvelin, Mika Kähönen, Jorma Viikari, Terho Lehtimäki, Olli T. Raitakari, Peter J. Meikle, Ville-Petteri Mäkinen, Mika Ala-Korpela

**Affiliations:** † Systems Epidemiology, Faculty of Medicine, University of Oulu, 90014 Oulu, Finland; ‡ Research Unit of Population Health, Faculty of Medicine, University of Oulu, 90014 Oulu, Finland; § Biocenter Oulu, 90014 Oulu, Finland; ∥ Baker Heart and Diabetes Institute, Melbourne 3004, Australia; ⊥ Baker Department of Cardiometabolic Health, University of Melbourne, Melbourne 3004, Australia; # Department of Public Health and Welfare, Finnish Institute for Health and Welfare, 00271 Helsinki, Finland; ∇ Department of Epidemiology and Biostatistics, MRC Centre for Environment and Health, School of Public Health, Imperial College London, London W12 0BZ, U.K.; ○ Department of Life Sciences, College of Health and Life Sciences, Brunel University London, London UB8 3PH, U.K.; ◆ Department of Clinical Physiology, Tampere University Hospital, and Finnish Cardiovascular Research Center Tampere, Faculty of Medicine and Health Technology, Tampere University, 33270 Tampere, Finland; ¶ Department of Medicine, University of Turku, 20014 Turku, Finland; & Division of Medicine, Turku University Hospital, 20014 Turku, Finland; ● Department of Clinical Chemistry, Fimlab Laboratories, and Finnish Cardiovascular Research Center Tampere, Faculty of Medicine and Health Technology, Tampere University, 33270 Tampere, Finland; ◊ Research Centre of Applied and Preventive Cardiovascular Medicine, University of Turku, 20014 Turku, Finland; ▲ Centre for Population Health Research, University of Turku and Turku University Hospital, 20014 Turku, Finland; □ Department of Clinical Physiology and Nuclear Medicine, Turku University Hospital, 20014 Turku, Finland; ^ InFLAMES Research Flagship, University of Turku, 20014 Turku, Finland; ¢ Monash University, Melbourne 3004, Australia; + NMR Metabolomics Laboratory, School of Pharmacy, University of Eastern Finland, 70210 Kuopio, Finland

## Abstract

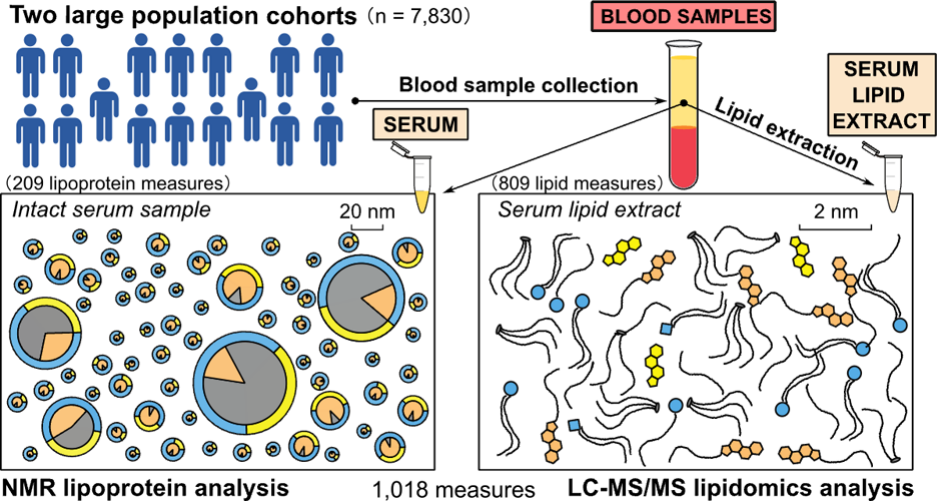

Applications of advanced omics methodologies are increasingly popular in biomedicine. However, large-scale studies aiming at clinical translation are typically siloed to single technologies. Here, we present the first comprehensive large-scale population data combining 209 lipoprotein measures from a quantitative NMR spectroscopy platform and 809 lipid classes and species from a quantitative LC-MS/MS platform. These data with 1018 molecular measures were analyzed in two population cohorts totaling 7830 participants. The association and cluster analyses revealed excellent coherence between the methodologically independent data domains and confirmed their quantitative compatibility and suitability for large-scale studies. The analyses elucidated the detailed molecular characteristics of the heterogeneous circulatory macromolecular lipid transport system and the underlying structural and compositional relationships. Unsupervised neural network analysis—the so-called self-organizing maps (SOMs)—revealed that these deep molecular and metabolic data are inherently related to key physiological and clinical population characteristics. The data-driven population subgroups uncovered marked differences in the population distribution of multiple cardiometabolic risk factors. These include, e.g., multiple lipoprotein lipids, apolipoprotein B, ceramides, and oxidized lipids. All 79 structurally unique triglyceride species showed similar associations over the entire lipoprotein cascade and indicated systematically increased risk for carotid intima media thickening and other atherosclerosis risk factors, including obesity and inflammation. The metabolic attributes for 27 individual cholesteryl ester species, which formed six distinct clusters, were more intricate with associations both with higher—e.g., CE(16:1)—and lower—e.g., CE(20:4)—cardiometabolic risk. The molecular details provided by these combined data are unprecedented for molecular epidemiology and demonstrate a new potential avenue for population studies.

Lipoprotein lipids have had a central role in clinical risk assessment for cardiometabolic diseases since the 1950s.^1^ The realization that ^1^H NMR spectroscopy would be inherently suited for lipoprotein analytics took place in the early 1990s.^2^ In addition to the fundamental advantages of ^1^H NMR spectroscopy in producing quantitative data, the spherical monolayered structure of lipoprotein particles provides a compelling attribute that the chemical shifts of lipid molecules transported by these particles are dependent on the particle diameter. This phenomenon arises from the orientational order of the surface lipids and the resulting anisotropy of the magnetic susceptibility, and leads to distinctive particle size-dependent frequency shifts. This facilitates comprehensive lipoprotein analytics that is pertinent to the metabolic constituents of the lipoprotein cascade. Practical applications and statistical considerations have indicated that 14 lipoprotein subclasses and their main lipid constituents can be robustly quantified.^3,4^ Multiple commercial methodologies have also been developed.^5−7^ Here we applied a platform that has been widely applied in large-scale epidemiology and genetics over the last 15 years with some 500 publications and 2 M samples analyzed, including all the 0.5 M serum samples in the UK Biobank.^3,5,8^ This particular platform has also played a key role in yielding open access genetic data, with the most recent genome-wide association study meta-analysis being carried out in 136,000 individuals.^9^

Mass spectrometry based lipidomics methodologies have been developed independent of the above and are technologically more challenging than the applications of ^1^H NMR spectroscopy.^10−12^ Nevertheless, epidemiological applications have become feasible over the recent years and clinical translation has been suggested, for example, with respect to assessing cardiometabolic risk^13,14^ and cognitive impairment.^15^ Initial genetic studies have also been published.^16,17^

The NMR spectroscopy and MS lipidomics approaches to lipid characterization and quantification are fundamentally different (Figure 1). In NMR the serum sample is directly analyzed with the lipoprotein particles intact; this allows quantification of the comprehensive and specific data on the lipoprotein subclasses and lipids.^18^ Unlike NMR, LC-MS/MS lipidomics calls for lipid extraction of the serum samples as an integral prerequisite for the analyses.^19^ The lipidomics sample preparations thus represent pooled mixtures of all the lipid molecules from all the circulating lipoprotein particles. All the information from the specific lipoprotein origin of a certain lipid in the circulation is lost in the extraction phase. However, with LC-MS/MS hundreds of unique lipid species can be analyzed from dozens of different molecular lipid classes.^12,20^

**Figure 1:**
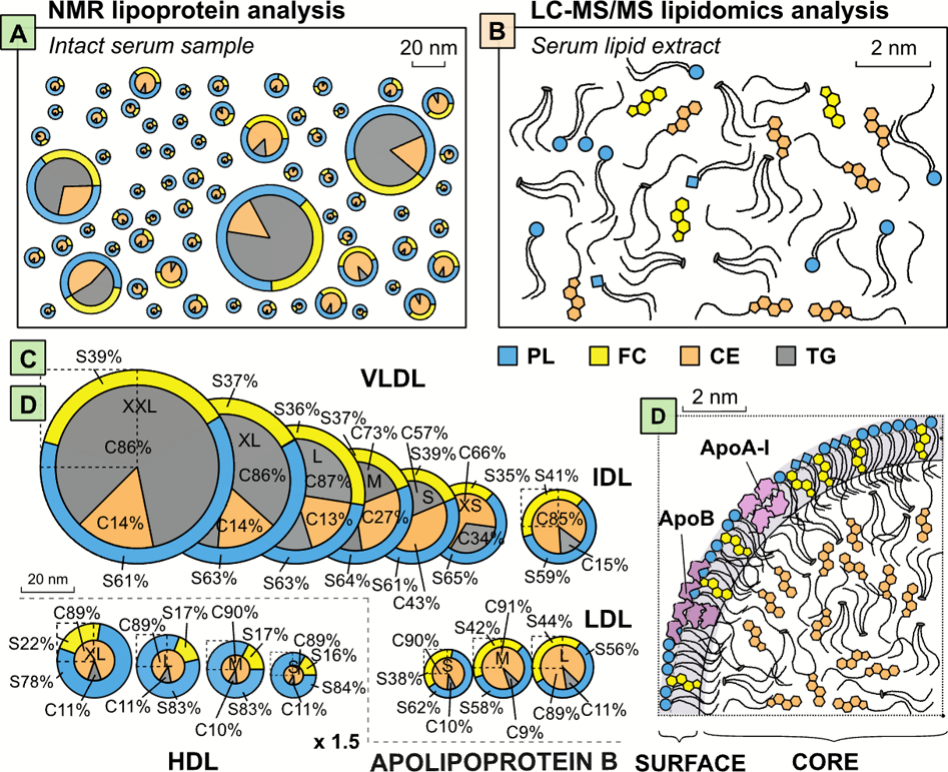
Schematic illustration of the methodological differences and analytical characteristics between ^1^H NMR and LC-MS/MS lipidomics and their molecular views into serum lipids. (A) The lipoprotein analysis with NMR is performed directly from the intact serum sample. (B) For the LC-MS/MS analysis all the lipids need to be extracted to a homogeneous lipid-soluble mixture. This allows LC-MS/MS to reach detailed molecular quantification, however, as a total serum concentration without any information on the originating lipoproteins. (C,D) The NMR analysis gives molecular information on 14 lipoprotein subclasses with the abundant core and surface lipids quantified. The percentages depict the compositional variation of these lipids in different lipoprotein particles.

Lipoprotein particles are the sole transport vehicles for all lipid molecules in serum,^18,21,22^ though albumin contains the bulk of free fatty acids and acylcarnitines, and transports also various lyso-type of lipid molecules.^23,24^ The NMR and the LC-MS/MS data on serum essentially represent the same lipids as noted by Ghorasaini et al.^25^ The NMR provides the size-resolution and metabolic information on lipoprotein particles (209 measures) and the LC-MS/MS lipidomics opens up a prolific and detailed molecular view on all the circulating lipids (809 measures).

These different methodologies and molecular views to study serum lipids have independently advanced toward large-scale population studies. Here, we integrate these state-of-the-art data—over 1000 quantitative molecular lipid measures per individual—in two large-scale population cohorts of over 7800 participants. These data reveal the fundamental relationships between the lipoprotein subclass cascade and metabolism (NMR) and the plethora of specific molecular information (LC-MS/MS). The molecular and metabolic corollaries are numerous, and we elaborate on some pertinent findings. Among the various analyses we also demonstrate the admirable coherence within the independent experiments and data domains. It is also uncovered—via data-driven, unsupervised neural network analysis—that these molecular lipid measures per se can be utilized for metabolically and clinically meaningful subgrouping of populations. The results illustrate that these combined comprehensive data lead to improved molecular characterization of population-level metabolic health and cardiometabolic risk.

## Materials and Methods

### Population Cohorts

Two cohorts were studied: the Northern Finland Birth Cohort 1966 (NFBC66) with 5657 participants (median age 46 years, 56% women) and the Young Finns Study (YFS) with 2036 participants (43 years, 55% women). Details on these cohorts are given in the Supporting Information, including the clinical characteristics in Table S1. NFBC66 is one of the biggest epidemiological cohorts with LC-MS/MS data available and these cohorts comprise a unique combination of large-scale molecular data for comprehensive lipoprotein panels and lipidomics measures.

### ^1^H NMR Spectroscopy

**Figure 2:**
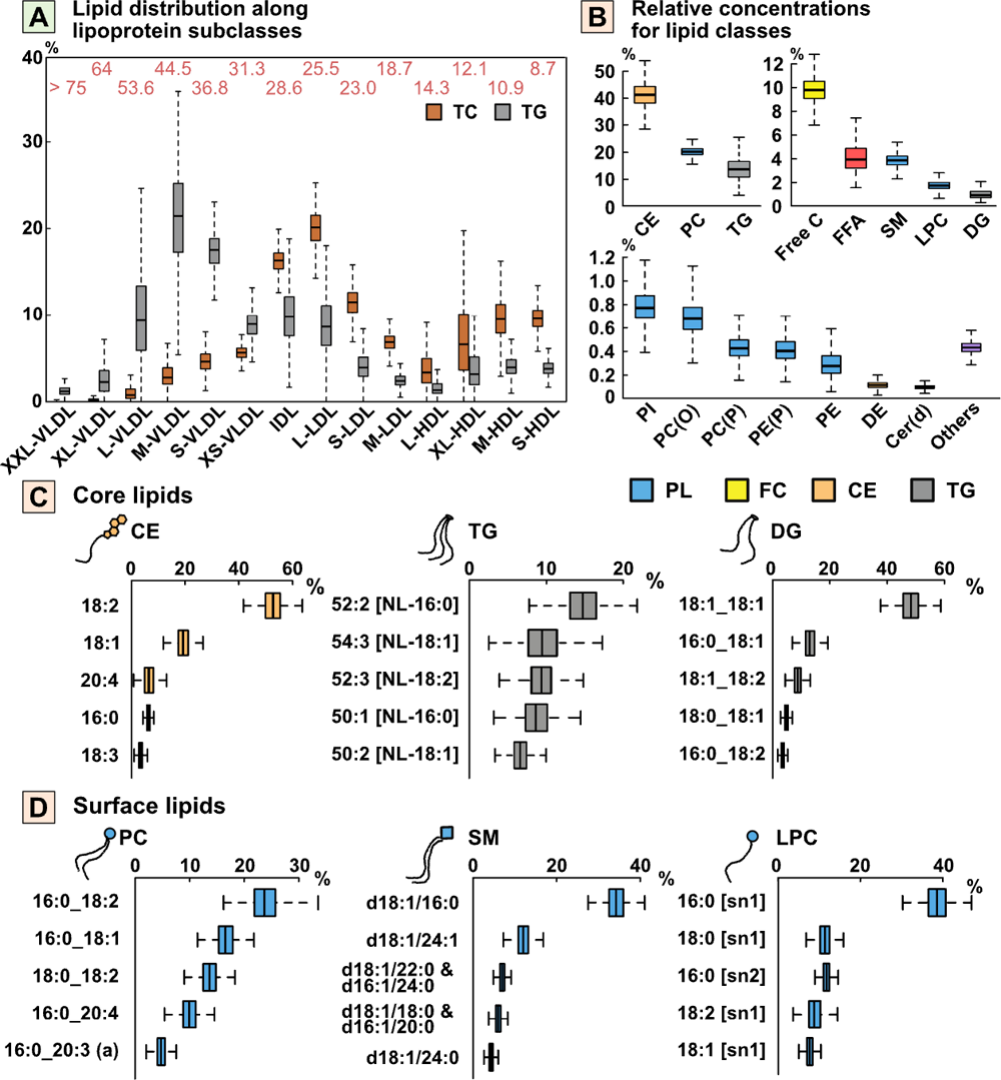
Illustration of the key data from the ^1^H NMR and LC-MS/MS analyses. (A) NMR quantifies the most abundant lipid classes in 14 size-specific lipoprotein subclasses and results in a comprehensive view on systemic lipoprotein metabolism. For example, the main transport of TG takes place in VLDL particles (over 60% of circulating amount) and that of cholesterol in IDL and LDL particles (over 50%). (B–D) LC-MS/MS quantifies also low abundance lipid classes and related molecular species. (B) Relative concentrations for the 16 most abundant lipid classes in the circulation. The five most abundant species for the most abundant core (C) and surface lipids (D) in lipoprotein particles.

We applied an NMR platform that has been widely used in epidemiology and for which the general methodological issues have been published.^3,5,8,9,18^ The separation of lipoprotein subclasses by proton NMR spectroscopy is based on particle size,^2^ the platform resolution being 14 subclasses (Figure 2).^3,18^ Independent verification for the number of subclasses has been published by Mihaleva and co-workers with an in-depth handling of statistical grounds.^4^ Particle (P), phospholipid (PL), free cholesterol (FC), cholesteryl ester (CE), and triglyceride (TG) concentrations are quantified for all 14 subclasses, also allowing calculation of the relative lipid composition for each lipoprotein subclass. The platform also provides all key clinically used lipid measures (e.g., total TG, cholesterol, and HDL-C) as well as apolipoprotein B (apoB) and A-I (apoA-I) concentrations. In total 209 lipoprotein-related measures were analyzed (98 concentration and 70 compositional measures from 14 lipoprotein subclasses, 19 clinical lipid measures, and 22 measures related to fatty acids, average particle sizes, and apoA-I and apoB).

### Lipoprotein Subclasses

The lipoprotein subclass information can be partitioned into three key categories of variables: (1) circulating particle concentrations, (2) circulating lipid concentrations, and (3) particle lipid compositions as the percentage of a certain lipid class of total lipids (mol %). All the measures are listed in Supporting Information Table S2 together with their median concentrations for both cohorts. The relative circulating concentrations for TG and cholesterol in the 14 lipoprotein subclasses are illustrated in Figure 2.

### LC-MS/MS Mass Spectrometry

Serum lipids were extracted as previously described.^19^ The LC-MS/MS profiling was performed using the standardized methodology described recently by Huynh et al.^12^ and updated with a larger set of targets. The mass spectrometric details and parameters are given in the Supporting Information.

### Lipid Classes and Species

The LC-MS/MS platform resulted in quantitative data for 809 measures: 38 lipid classes with 767 individual lipid species plus four classes with a single detectable molecular species, namely free cholesterol, ubiquinone, ceramide-1-phosphate, and the GM1 ganglioside. Relative concentrations for the 16 most abundant lipid classes in the circulation are shown as a summary in Figure 2. All the measures are listed in Supporting Information Tables S3 and S4 together with their median concentrations for both cohorts. These data provide valuable reference concentrations for key lipid measures at population level (individual variation is depicted in Supporting Information Figure S1). Notably only eight lipid classes constitute over 97% of circulating lipids. In general, within each lipid class, a few molecular species are clearly more abundant than the rest. The most abundant lipid classes are also rich in molecular variety, for example, 79 individual molecular species are identified in the TG class, 64 for phosphatidylcholines, and 44 for sphingomyelins. This detailed panel of individual lipid species provides a novel and compelling opportunity to study the molecular intricacies of lipoprotein metabolism and extends the molecular epidemiology approach to a mostly unknown area.

### Statistical Analyses

Partial Spearman’s correlations were calculated between all the lipoprotein and lipidomics measures. Both cohorts were analyzed separately and then combined via inverse variance weighted meta-analysis. All correlations were adjusted for sex in both cohorts and age in YFS. Hierarchical clustering was applied to the lipidomics data domain to facilitate visual viewing of the results from the correlation analyses. Two-hundred principal components explained over 95% of the variation in the 1018 lipoprotein and lipid measures in both cohorts. Therefore, we set the 5% Bonferroni-adjusted type 1 error threshold at *p* < 0.05/200 = 0.00025. Extreme values for all measures were truncated in all analyses to third quartile +8 × interquartile range. Extreme values were rare and had negligible effects on the results. All analyses were done with the R software (version 4.2.1).

### Self-Organizing Maps

**Figure 3:**
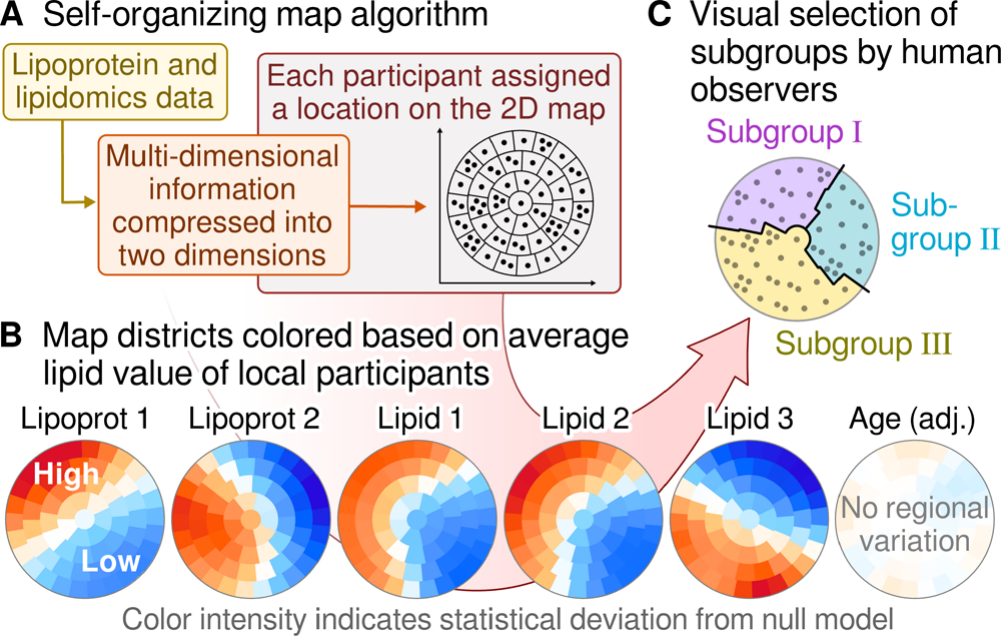
Schematic illustration of the self-organizing map (SOM) framework. (A) First, the SOM algorithm enables the representation of the multidimensional data set as a two-dimensional map in which proximity between participants (black dots) indicates similarity in the metabolic profile. (B) Second, the map is colored by each measure to compare the regions, i.e., how the average values of these measures for the participants compare between the map districts. (C) Lastly, the map is divided into (population) subgroups of participants; here this was based on the lipoprotein and lipidomics measures used as inputs. Clinical and biochemical data, not used in the training of the SOM, can then be visualized similarly to the metabolic inputs.

The Numero R software package^26^ was used to derive the data-driven metabolic subgroups. The NMR and LC-MS/MS data were combined to generate 1018 quantitative molecular inputs for the 7830 participants. The overall process and principles of SOM analysis are depicted in Figure 3. This framework has been used in numerous metabolic studies^27^ and here we followed the de facto standard procedure as instructed in the software documentation (see also Supporting Information).^26^

## Results and Discussion

### Characteristics of Integrated Data

The NMR and LC-MS/MS data domains with key measures are depicted in Figures 1 and 2. The two essential background issues, namely the serum sample and the related measurements as well as the biological system and lipoprotein and lipid metabolism are briefly introduced above, and more details are given in the Supporting Information.

NMR is unique in the ability to comprehensively quantify a multitude of lipoprotein subclasses.^2,3,5,8,9,18^Figure 1 illustrates the ten apoB-containing and four HDL particles that can be analyzed together with their main lipid constituents, namely PL, FC, CE, total cholesterol (TC), and TG. The VLDL particles are large and buoyant with TG-enriched cores (up to 70% of all particle lipids). The IDL and LDL particles are smaller, at around 20–30 nm in diameter with CE-enriched cores (up to 70% of all particle lipids). Consistent with these compositional features, VLDL particles are the main carriers of TG (some 60% of circulating TG) and IDL and LDL particles the cholesterol (some 50% of circulating TC) in the bloodstream. All the particles have a functional monolayer that allows the particles to be soluble in water and controls the particle–particle and particle–cell interactions.^18,21,22^ The main molecular constituents in the surface monolayer are apolipoproteins, PL, and FC. HDL particles represent over 90% of circulating particles but apoB particles carry 65% of circulating lipids.

LC-MS/MS is unique in the ability to comprehensively identify and quantify hundreds of individual lipid species in dozens of different lipid classes.^10−12,19^ In Figure 2, the relative percentages of the 16 most abundant lipid classes in serum are depicted (data for all the lipid classes are given in Supporting Information Table S3 and a literature comparison for the most abundant lipid classes in Table S5). CE, TG, phosphatidylcholines (PC), and FC are the most abundant lipid classes amounting to almost 90% of all circulating lipids. Sphingomyelins (SM), lyso-PC, and diacylglycerols (DG) together contribute around 5%. The rest of the lipid classes contribute less than 1% each of all the circulating lipid molecules, except free fatty acids (FFA) that account for some 3% and are transported by albumin. It is also noticeable that within each lipid class, there is a tendency that the three to five most abundant species account for over 50% of the concentration of the entire class. For example, CE(18:2) alone contributes over 50% and CE(18:1) almost 20% of all CE. TG(52:2), TG(52:3), and TG(50:2) contribute around 40% of all TG (Figure 2).

### Associations between the Lipoprotein Subclasses and Lipid Classes

**Figure 4:**
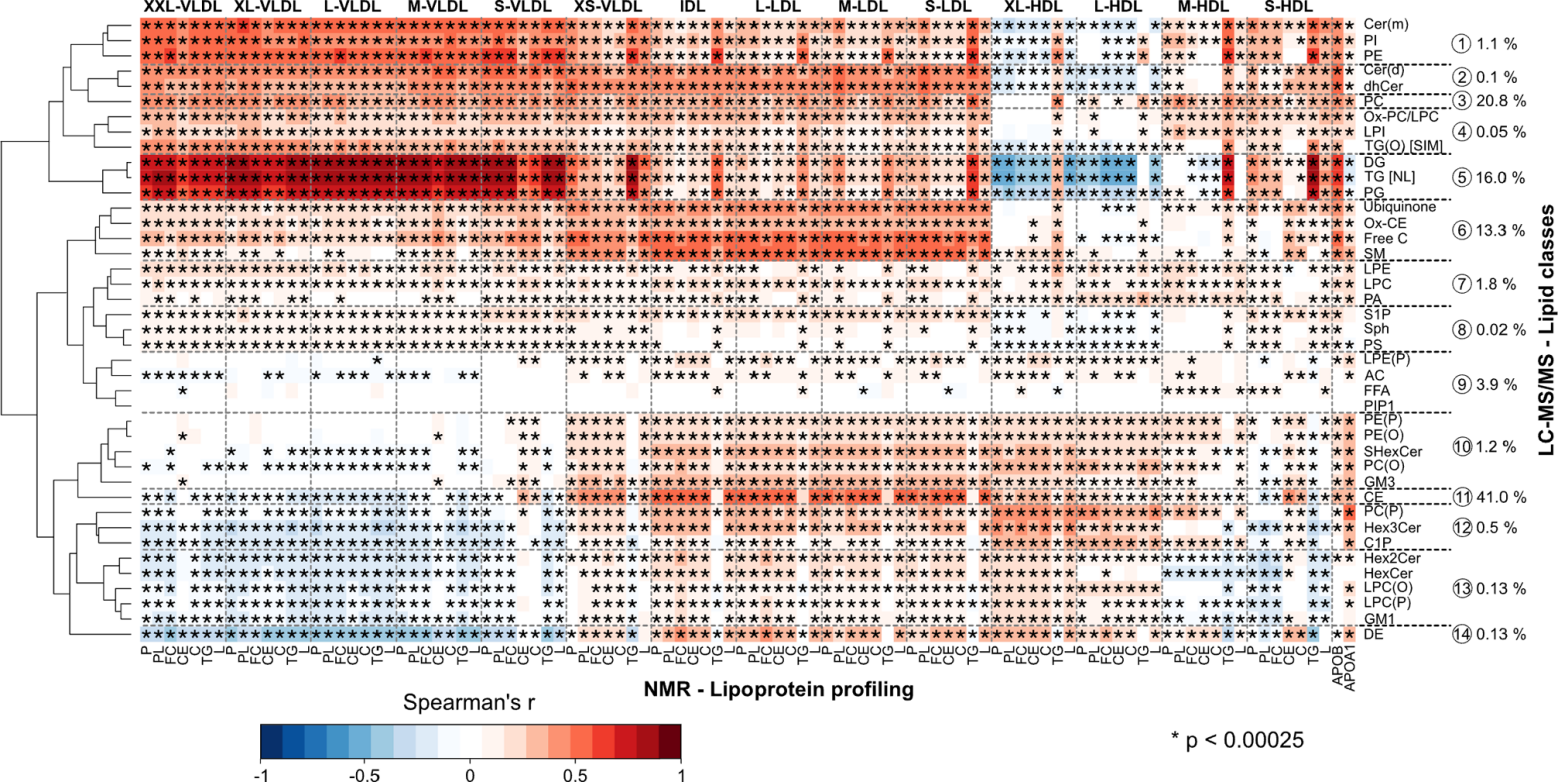
Associations between the concentrations of 40 lipid classes (LC-MS/MS) and the particle and lipid concentrations of 14 lipoprotein subclasses (NMR) as indicated by partial Spearman’s rank correlations. The data are from two independent large population studies, NFBC66 (5657 participants) and YFS (2173 participants). All correlations were adjusted for sex in both cohorts and age in YFS. Both cohorts were analyzed separately and then meta-analyzed. The LC-MS/MS data are organized to clusters and the NMR-based lipoprotein data are presented in the order of decreasing particle size, since these data blocks intrinsically correspond the dominant metabolic pathways. The general association characteristics for the lipid classes are summarized via 14 metabolic clusters. The percentages shown depict the contribution of each cluster to the circulating total lipid concentration. *P*-value <0.00025 is marked with an asterisk to indicate a multiple testing corrected association. Abbreviations are in the Supporting Information.

Partial Spearman’s correlations between the NMR-based lipoprotein and the LC-MS/MS-based lipid class measures are illustrated in Figure 4. The NMR axis represents lipoprotein subclass particle (denoted by P) and main lipid concentrations (total lipids denoted by L). The LC-MS/MS axis shows concentrations for 40 specific molecular lipid classes. The clustered heatmap is organized for the LC-MS/MS data, while the NMR-based lipoprotein data are presented in the order of decreasing particle size from the largest VLDL to the smallest HDL particles, since these data blocks intrinsically correspond to the two dominant metabolic pathways.^18,25^

The most pronounced positive correlation block is noted between the five largest VLDL subclasses and the LC-MS/MS lipidomics cluster no. 5 that consists of di-, tri-, and phosphatidylglycerols. This is anticipated due to the key role of VLDL particles in the transport of these molecular components in the bloodstream. The related strong negative correlation block between cluster no. 5 and the XL-, L-, and M-HDL subclasses arises from the strong systemic negative metabolic association between triglycerides and HDL-C. This is an important notion in the sense that, even though we have specific molecular measures in both data domains, the circulating lipoprotein particles are metabolically constrained for both absolute and relative concentrations.^18^ The population-level correlation between triglycerides and HDL-C is typically up to −0.7:^28^ for the NFBC66 and YFS it is −0.64 and −0.56, respectively.

Another demonstration of the excellent coherence within the independent data domains is the strong positive correlation block between lipidomics cluster no. 6, the main components of which are FC and SM, and the XS-VLDL, IDL, and LDL particles, which transport some 60% of circulating cholesterol. The associations of CE (lipidomics cluster no. 11) are similar but show weak negative associations for the XXL- to S-VLDL subclasses and positive associations to all HDL subclasses (that transport some 30% of circulating cholesterol).

The molecular information in Figure 4 is substantial and shows many attributes that have not been elucidated before. Since ceramides have been suggested as independent biomarkers for cardiovascular disease risk,^29^ it is of interest to note that the lipidomics cluster no. 2, consisting of circulating ceramides (Cer) and dihydroceramides (dhCer), is strongly positively associated with the entire metabolic cascade of apoB-containing lipoprotein particles. Several components of this pathway, including TG, LDL-C, and apoB, are known to be causal for the development of coronary heart disease.^1,30,31^ It is also notable that this ceramide cluster constitutes only 0.1% of the total circulating lipid concentration. Nonetheless, the low circulating concentration per se does not prevent ceramides (or any low abundance lipid class or species) to be relevant or causal for the atherosclerotic process, particularly since it has been demonstrated that the interindividual differences in LDL particle lipidome can affect the susceptibility of these particles for enzymatic modifications and influence the development and progression of atherosclerosis.^32^ Based on abundant genetic epidemiology evidence, it is however more probable that the key mediating factor is intrinsically the apoB.^31^ The minor compositional differences in the LDL particles may not matter much over decades of apoB overload.^33^

Notwithstanding, complementary studies on the (potentially causal) role of ceramides are needed to put these findings on the population associations of ceramides and cardiometabolic outcomes under scrutiny together with comprehensive lipoprotein data. This notion is not relevant only for ceramides, but to all lipidomics measures. Coming to robust conclusions on these individual constituents of lipoprotein particles is demanding as recently elaborated by Borges et al.^34^ in the case of circulating polyunsaturated fatty acids and calling attention to the potential bias in the analyses of individual lipid measures due to the lipoprotein-related mediation.

The molecular characteristics of the various subclass particles are heterogeneous with many lipid classes and species being distributed unevenly (see also Figure 1C).^18,23^ Within these fundamental impediments, the overall view from Figure 4 makes perfect sense between the NMR-based lipoprotein profiling and the LC-MS/MS-based comprehensive lipid analyses. This unique outlook would also serve well as a detailed guide for the potential key lipoprotein mediators for various lipidomics associations in epidemiology and biomedical studies when comprehensive lipoprotein profiles are unavailable (Spearman’s rank correlations between all the NMR and LC-MS/MS measures are given in a numerical form in Supporting Information Table S6).

The associations of the lipidomics cluster no. 9 with the lipoprotein cascade are very weak. The main component of this cluster is FFA, that are known to be almost entirely transported by albumin. It is therefore likely that, at least up to a noticeable portion, the other lipids, namely lysoalkenylphosphatidylethanolamines (plasmalogens; LPE(P)), acylcarnitines (AC), and phosphatidylinositol monophosphate (PIP1), are transported by albumin, or other proteins than lipoproteins, in the bloodstream. These molecules exemplify the unique benefits of the lipidomics analyses in providing specific biological information beyond the lipoprotein cascade.

### Associations between the Lipoprotein Subclasses and Lipid Species

**Figure 5:**
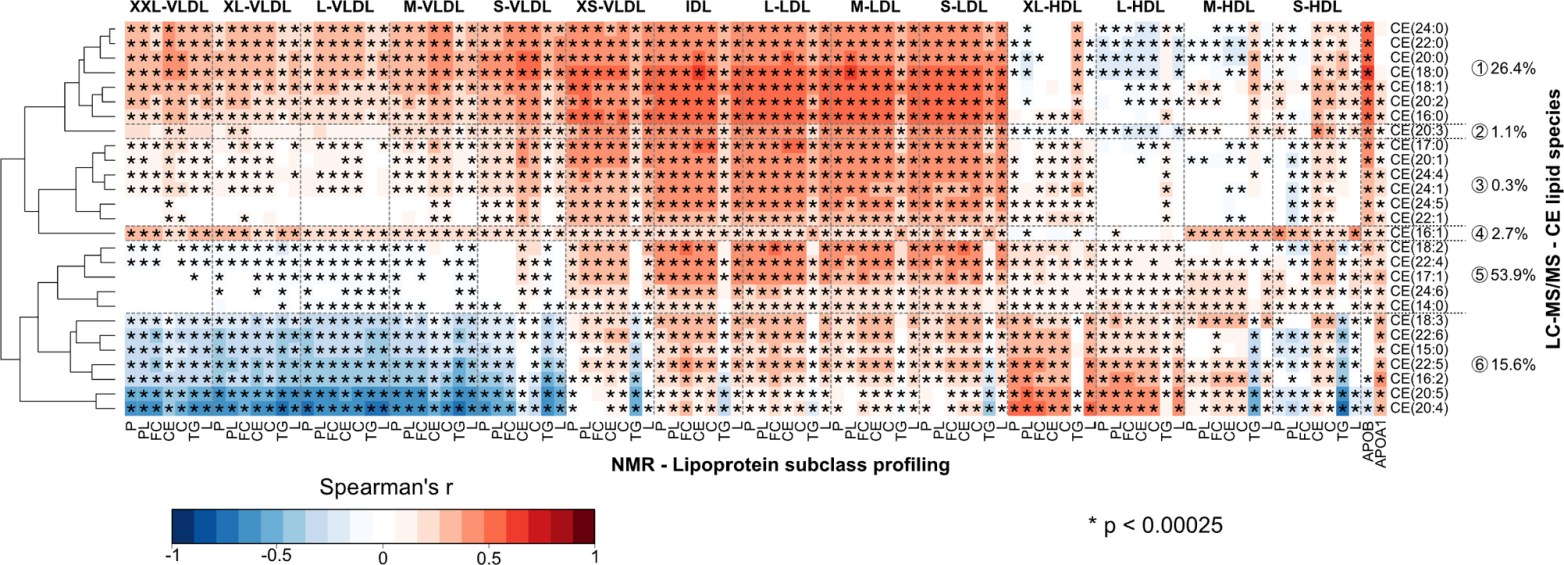
Associations between the concentrations of 27 individual CE species (LC-MS/MS) and the particle and lipid concentrations of 14 lipoprotein subclasses (NMR) as indicated by partial Spearman’s rank correlations. The general association characteristics for the CE species are summarized via six metabolic clusters. The percentages shown depict the contribution of each cluster to the circulating total CE concentration. Otherwise, the data and analyses are as detailed in the caption for Figure 4.

The LC-MS/MS lipidomics data for the lipid classes (Figures 2 and 4), represent concentrations of all the individual lipid species summed up for a total concentration for each lipid class. Therefore, the most abundant lipid species in each lipid class are those that contribute nearly all to these total concentrations and can potentially overwhelm the associations of less abundant molecular species. Triglycerides are a representative example of a lipid class within which all the analyzed individual molecular species appear to associate in a very similar manner with the entire lipoprotein cascade (Supporting Information Figure S2). Thereby, the individual TG species are likely to behave similarly to the total TG class associations. However, as depicted in Figure 5, the metabolic attributes for the analyzed 27 cholesteryl ester species are more intricate with six distinct molecular clusters that substantially differ in their associations with the lipoprotein cascade. CE clusters no. 1 (contributing 26.4% of total CE concentration and including CE(18:1), the second most abundant species), nos. 2, 3, and 4 (solely CE(16:1)) have positive associations for the XXL- to S-VLDL subclasses. CE clusters no. 5 (with CE(18:2), the most abundant CE species) and particularly 6 (with CE(20:4), the third most abundant species) manifest negative corresponding associations as also the total CE lipid class in Figure 4. This is logical since CE clusters no. 5 and 6 contain almost 70% of the total circulating CE concentration.

Thus, with any lipid class within which the lipid species differ in their associations with the lipoproteins, it should be noted that the class association reflects only the most abundant species. The associations of the lipid species also vary for different lipoprotein subclasses: e.g., all individual CE species associate positively with all the LDL subclasses, but there are strongly positively as well as strongly negatively associated CE species with respect to various VLDL subclasses.

In general, there are multiple lipid classes that have heterogeneous lipoprotein associations with their constituent lipid species, e.g., lysophosphatidylcholines and GM3 gangliosides. Like with the lipid classes (Figure 4), these novel association maps between the hundreds of individual lipid species and the lipoprotein subclasses can assist in understanding the intertwined metabolic issues and help in interpreting the specific lipidomics data (Supporting Information Figures S2–S11).

### Self-Organizing Maps and Population Stratification

We demonstrated in the UK Biobank, with clinical biochemistry data from 329 908 participants, that cross-sectional metabolic subgroups relate to future disease burden and multimorbidity.^27^ Here, we have extensive molecular data (over 1000 measures) on lipoprotein and lipid metabolism for two large populations (over 7800 participants) and we are interested in understanding how these lipid measures–intrinsically–relate to the population health characteristics and disease risk, and–concomitantly–how the lipoprotein metabolism and the related attributes (from NMR) associate with the molecular details of multiple lipid classes and species (from LC-MS/MS). With the SOM analysis both objectives were achieved.

**Figure 6:**
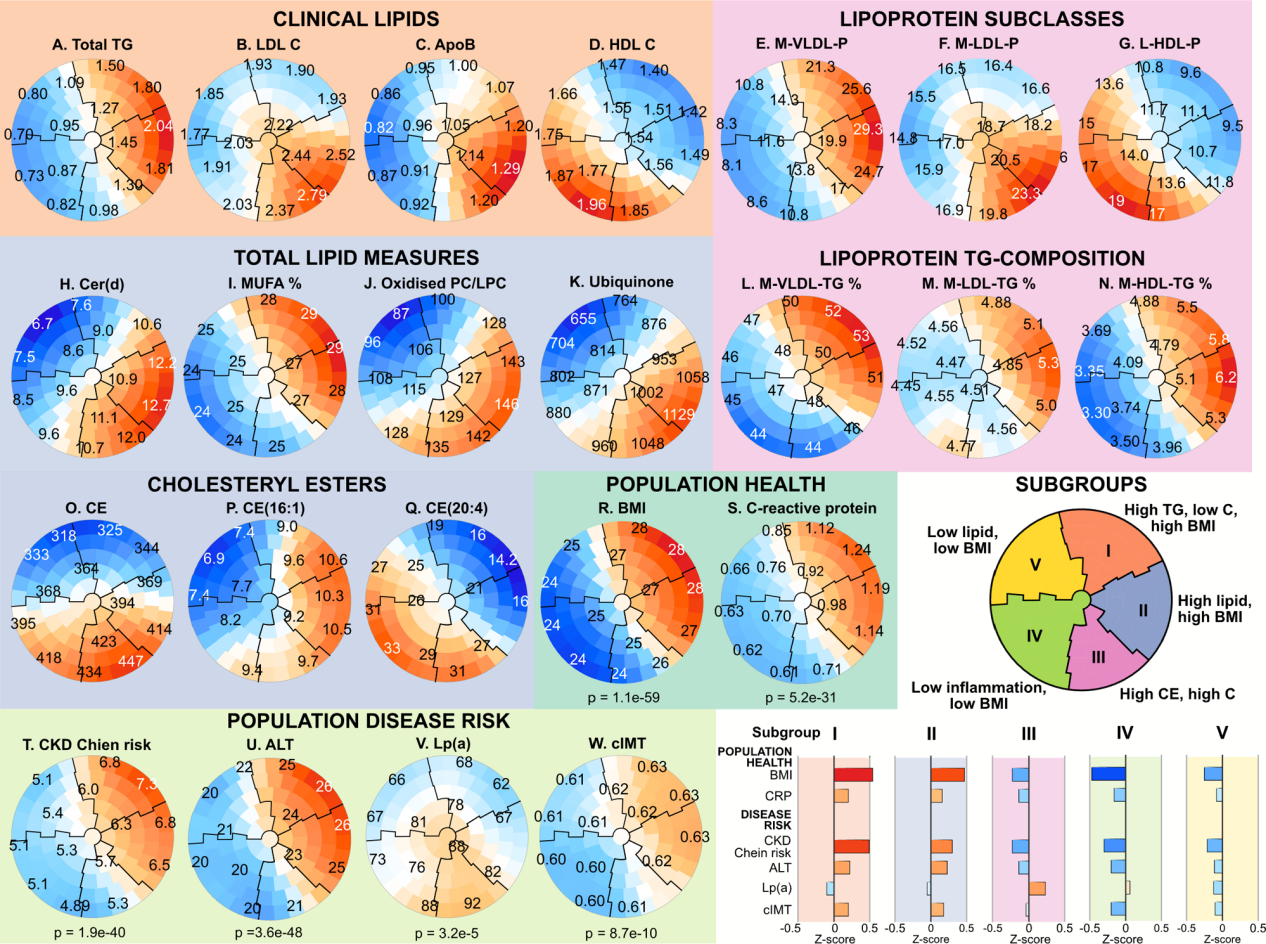
SOM-based subgrouping with the combined lipoprotein and lipid data from NMR and LC-MS/MS (209 and 809 quantitative measures, respectively) in two population cohorts, NFBC66 with 5657 and YFS with 2173 participants. The SOM was applied with all 7830 participants and all 1018 molecular inputs. In each plot, the same participants reside in the same district. The district colors indicate the regional deviation from the global mean and the numbers indicate local mean values. The biomarker planes for all the inputs are given in Supporting Information Figure S12. The histograms are to clarify and emphasize the inherent capability of the comprehensive lipoprotein and lipid data to reveal key aspects of population health and disease risk.

Figure 6 depicts representative SOM biomarker planes to visually tie together clinical lipid measures, lipoprotein subclass metabolism, lipidomics, and how these molecular measures associate with population health and disease aspects for the resultant five population subgroups. One of the first messages conveyed by an overall look is the consistently high triglycerides (biomarker plane A) for subgroups I and II. This pattern is almost identical with the one for body mass index (BMI, R) and C-reactive protein (CRP, S), indicating that, at the population level, obesity, inflammation, and triglycerides are strongly positively associated. This is a well-known metabolic characteristic of obesity^35^ and serves here as a confirmation to support the SOM analysis. The compositional information on the lipoprotein subclass particles (L, M, and N) reveals that high circulating triglycerides (A) are associated with TG-enrichment of all lipoprotein particles, including HDL, and possibly reflecting higher risk of coronary heart disease, independent of total cholesterol and triglycerides.^36^

The SOM analysis connotate that high circulating concentrations of ceramides (Figure 6H) are strongly associated with high triglycerides (A), particularly for the population subgroup II, and with high LDL-C (B), for the population subgroups II and III. The associations of Cer with apoB (C) are like with LDL-C. The subgroups I and II are characterized by high BMI, high CRP, and TG-enriched lipoprotein particles. In addition, the individuals with these metabolic profiles are at higher risk for kidney (T) and liver dysfunction (U) as well as for coronary heart disease (W) than individuals characterized by the metabolic profiles in subgroups III, IV, and V. The population subgroup III is the only one characterized also by high lipoprotein (a) (Lp(a)) (Figure 6V), a causal cardiometabolic risk marker that is highly genetically controlled and has only minor links to other lipoprotein measures.^37,38^

The highest ceramide concentrations at the population level therefore coincide with the highest apoB (subgroup II) as well as with the highest Lp(a) concentrations (subgroup III) but are not clearly elevated within subgroups I with elevated TG. Thereby, even though highly correlated with the TG-related lipoprotein metabolism (Figure 4), its associations with cardiometabolic outcomes are likely to be more related to cholesterol- than TG-metabolism. Notably, this is a finding arising solely based on the combined comprehensive lipoprotein and lipid data, without any additional information on cardiometabolic risk or disease outcomes. This does not confirm an independent role for ceramides in the cardiometabolic risk assessment, but it does give support for further studies on its potential additive role as a cardiometabolic biomarker.

As discussed in relation to Figure 5, the individual cholesteryl ester species form six distinct molecular clusters that differ in their associations with the lipoprotein cascade. The integration of lipoprotein and individual CE species data gives a unique perspective also for the SOM analysis. The highest concentrations for the total CE class (Figure 6O) coincide with population subgroups II, III and IV. The highest concentrations for the individual CE species CE(16:1) (P) coincide with subgroups I, II and III, but not IV. On the contrary, CE(20:4) (Q) coincide with subgroups III, IV and V, but not II.

In one of the first population level lipidomics studies, CE(16:1) was found to be associated with the development of cardiovascular disease.^39^ In a related editorial,^40^ mechanistic insights were proposed relating the, e.g., diet originating saturated fatty acids, to the generation of monounsaturated CEs and their integration into the VLDL particles in the liver, eventually resulting in LDL particles enriched in monounsaturated CEs, that might be more prone to advance the intimal lipid accumulation and the progression of atherosclerosis.

While this still remains a hypothesis, the current results reflect higher cardiometabolic risk for CE(16:1) than for CE(20:4). Since the LC-MS/MS measures reflect total circulating concentrations, both the lipoprotein subclass distribution and particle compositions affect these measures. It is the subclass distribution that contributes markedly more for the circulating lipid concentrations,^18^ so the primary interpretation on the heterogeneity regarding CE(16:1) and CE(20:4) is that it is due to differences in lipoprotein subclass profile (Figure 6E–G) with different lipoprotein subclasses being inhomogeneous regarding the transport of different CE species. Apparently CE(20:4) concentrations (Q) are strongly associated with large HDL particles (G), known to be associated negatively with cardiometabolic risk, as also indicated by various biomarker planes in Figure 6. The participants in both population studies are rather young, so there is a very limited number of disease outcomes. However, the carotid intima media thickness (cIMT, W), an indicator of vasculopathy associated with the development of atherosclerotic plaques, does show thickest values for subgroups I and II. All CE-measures appear high for subgroup III with also elevated LDL (F) and Lp(a) (V) concentrations. High CE(16:1) concentrations also mostly coincide with high oxidized (J and K) and monounsaturated lipids (I), all linked to increased risk of atherosclerosis. The current results therefore support the earlier findings related to CE(16:1) and the role of monounsaturated CEs as molecular risk factors for atherosclerosis.^39,40^ However, how much, if any, of this increased risk is related to the specific molecular species (e.g., monounsaturated CE(16:1) and CE(18:1)) or relative lipid compositions (e.g., enrichment of certain lipoprotein particles in monounsaturated lipids or triglycerides) and how much is mediated by the metabolic issues directly in relation to the lipoprotein subclass concentrations, remains to be determined.

## Conclusions

The presented combination of lipoprotein and lipid data from NMR spectroscopy and LC-MS/MS lipidomics led to an unprecedented metabolic and molecular detail with over 1000 measures per person in a population setting of over 7800 individuals. The analyses demonstrated notable coherence between the data from inherently different spectroscopic techniques. The data-driven analyses revealed multiple confirmatory and novel molecular results in relation to systemic lipid metabolism and illustrated the power of all-inclusive lipid data in understanding cardiometabolic population health, related clinical factors, and disease risk. The analyses emphasized the augmented value of interpreting lipidomics data (deep molecular phenotyping) together with lipoprotein data (deep metabolic phenotyping) and association heatmaps between all the lipidomics measures and the key measures characterizing lipoprotein subclass metabolism are provided. These unique motifs are useful as guidelines for the key lipoprotein mediators for various lipidomics associations in biomedical studies when comprehensive lipoprotein profiles are not available.

## Supporting Information Available

Detailed information regarding study populations, data, methods, funding, and supplementary results (PDF)
LC-MS/MS lipidomics measures for the concentrations of the circulating individual lipid species in NFBC66 and YFS (XLSX)
Spearman’s rank correlations (adjusted for sex) (XLSX)

## Notes

The abbreviations are given in the Supporting Information.
