# Correction to “Personalized Profiling of Lipoprotein and Lipid Metabolism Based on 1018 Measures from Combined Quantitative NMR and LC-MS/MS Platforms”

**DOI:** 10.1021/acs.analchem.5c01621

**Published:** 2025-04-01

**Authors:** Siyu Zhao, Corey Giles, Kevin Huynh, Johannes Kettunen, Marjo-Riitta Järvelin, Mika Kähönen, Jorma Viikari, Terho Lehtimäki, Olli T. Raitakari, Peter J. Meikle, Ville-Petteri Mäkinen, Mika Ala-Korpela

In our original article, two minor errors need corrected:

(1) On page 20363, in the Materials and Methods section, under the Population Cohorts subsection, there is a wrong number given for the number of participants in the Young Finns Study (YFS). Instead of “the Young Finns Study (YFS) with 2036 participants (43 years, 55% women)”, it should be “the Young Finns Study (YFS) with 2173 participants (39 years, 55% women)”.

Importantly, all analyses and data used in the work are correct; i.e., this is only a mistake with the number and the age of the participants. These numbers came from another time point of YFS, but no data from that time point were used. The numbers referring to YFS in all other places in the paper are correct.

(2) An identification line is missing from Figure S4 (page S27) of the Supporting Information regarding the heatmap for phosphatidylcholine (PC). The missing identification is PC(18:0_22:5)(n3) and PC(20:1_20:4). This is now corrected in the attached Supporting Information file.

## Supporting Information Available

Detailed information regarding study populations, data, methods, funding, and supplementary results (PDF)

