# Supplementary material for: Personalized Profiling of Lipoprotein and Lipid Metabolism Based on 1018 Measures from Combined Quantitative NMR and LC-MS/MS Platforms
Source: Anal Chem. 2024 Dec 16;96(52):20362–70. doi: 10.1021/acs.analchem.4c03229 (PMC11696825; doi:10.1021/acs.analchem.4c03229)
Supplement: Supplementary file 1 — ac4c03229_si_001.pdf [file ac4c03229_si_001.pdf]

Supporting Information

Personalized Profiling of Lipoprotein and Lipid Metabolism Based on 1018 Measures from Combined Quantitative NMR and LC-MS/MS Platforms

Siyu Zhao,[1,2,3] Corey Giles,[4,5] Kevin Huynh,[4,5] Johannes Kettunen,[1,2,3,6] Marjo-Riitta Järvelin,[2,7,8] Mika Kähönen,[9] Jorma Viikari,[10,11] Terho Lehtimäki,[12] Olli T. Raitakari,[13,14,15,16] Peter J. Meikle,[4,5,17] Ville-Petteri Mäkinen,[1,2,3] and Mika Ala-Korpela[1,2,3,17,18,*]

[1]Systems Epidemiology, Faculty of Medicine, University of Oulu, 90014 Oulu, Finland; [2]Research Unit of Population Health, Faculty of Medicine, University of Oulu, 90014 Oulu, Finland; [3]Biocenter Oulu, 90014 Oulu, Finland; [4]Baker Heart and Diabetes Institute, Melbourne 3004, Australia; [5]Baker Department of Cardiometabolic Health, University of Melbourne, Melbourne 3004, Australia; [6]Department of Public Health and Welfare, Finnish Institute for Health and Welfare, 00271 Helsinki, Finland; [7]Department of Epidemiology and Biostatistics, MRC Centre for Environment and Health, School of Public Health, Imperial College London, London W12 0BZ, UK; [8]Department of Life Sciences, College of Health and Life Sciences, Brunel University London, UB8 3PH, UK; [9]Department of Clinical Physiology, Tampere University Hospital, and Finnish Cardiovascular Research Center Tampere, Faculty of Medicine and Health Technology, Tampere University, 33270 Tampere, Finland; [10]Department of Medicine, University of Turku, 20014 Turku, Finland; [11]Division of Medicine, Turku University Hospital, 20014 Turku, Finland; [12]Department of Clinical Chemistry, Fimlab Laboratories, and Finnish Cardiovascular Research Center Tampere, Faculty of Medicine and Health Technology, Tampere University, 33270Tampere, Finland; [13]Research Centre of Applied and Preventive Cardiovascular Medicine, University of Turku, 20014 Turku, Finland; [14]Centre for Population Health Research, University of Turku and Turku University Hospital, 20014 Turku, Finland; [15]Department of Clinical Physiology and Nuclear Medicine, Turku University Hospital, 20014 Turku, Finland; [16]InFLAMES Research Flagship, University of Turku, 20014 Turku, Finland; [17]Monash University, Melbourne 3004, Australia; [18]NMR Metabolomics Laboratory, School of Pharmacy, University of Eastern Finland, 70210 Kuopio, Finland.

Table of Content

Supplement Note

Study Populations **S4**

LC-MS/MS Mass Spectrometry Analysis for lipidomics **S4**

Self-Organizing Maps **S5**

Characteristics of Integrated Data **S6**

Results and Discussion **S6**

References **S8**

Funding **S9**

Abbreviations **S10**

Supplement Tables

Table S1. Clinical characteristics in NFBC66 and YFS. **S13**

Table S2. NMR-based lipoprotein measures in NFBC66 and YFS. **S14**

Table S3. LC-MS/MS lipidomics measures for the total concentrations of the circulating lipid classes in NFBC66 and YFS. **S21**

Table S4. (Excel) LC-MS/MS lipidomics measures for the concentrations of the circulating individual lipid species in NFBC66 and YFS.

Table S5. Comparison of the relative abundances (mol-%) of the most common serum lipid classes in lipidomics studies. **S23**

Table S6. (Excel) A. Spearman's rank correlations (adjusted for sex) for the 209 lipoprotein and 809 lipidomics measures in NFBC66. **B**. Spearman's rank correlations (adjusted for sex) for the 209 lipoprotein and 809 lipidomics measures in YFS. **C.** Meta-analyzed Spearman's rank correlations (adjusted for sex) for the 209 lipoprotein and 809 lipidomics measures in NFBC66 and YFS.

Supplement Figures

Figure S1. Individual variation in the relative abundances of the four most abundant serum lipid classes in NFBC66 and YFS based on the LC-MS/MS data. **S24**

Figure S2. Associations between the concentrations of 25 individual DG species, 34 individual TG[SIM], and 79 TG[NL] species (LC-MS/MS) and the particle and lipid concentrations of 14 lipoprotein subclasses (NMR) as indicated by partial Spearman's rank correlations. **S25**

Figure S3. Associations between the concentrations of 3 individual Sph, 4 individual S1P, 6 individual dhCer, 50 individual Cer(d), 11individual Cer(m), and one C1P species (LC-MS/MS) and the particle and lipid concentrations of 14 lipoprotein subclasses (NMR) as indicated by partial Spearman's rank correlations. **S26**

Figure S4. Associations between the concentrations of 14 individual HexCer, 10 Hex2Cer, 6 Hex3Cer, 6 individual GM3, one GM1, 6 ShexCer, and 44 individual SM species (LC-MS/MS) and the particle and lipid concentrations of 14 lipoprotein subclasses (NMR) as indicated by partial Spearman's rank correlations. **S27**

Figure S5. Associations between the concentrations of 6 individual PA species and 68 individual PC species (LC-MS/MS) and the particle and lipid concentrations of 14 lipoprotein subclasses (NMR) as indicated by partial Spearman's rank correlations. **S28**

Figure S6. Associations between the concentrations of 61 individual LPC and 37 individual PE species (LC-MS/MS) and the particle and lipid concentrations of 14 lipoprotein subclasses (NMR) as indicated by partial Spearman's rank correlations. **S29**

Figure S7. Associations between the concentrations of 22 individual PC(O), 26 individual PC(P), 10 individual LPC(O), 6 individual LPC(P), 14 individual PE(O), 14 LPE, and 4 LPE(P) species (LC-MS/MS) and the particle and lipid concentrations of 14 lipoprotein subclasses (NMR) as indicated by partial Spearman's rank correlations. **S30**

Figure S8. Associations between the concentrations of 54 individual PE(P) species (LC-MS/MS) and the particle and lipid concentrations of 14 lipoprotein subclasses (NMR) as indicated by partial Spearman's rank correlations. **S31**

Figure S9. Associations between the concentrations of 38 individual PI, one PIP1, 8 individual LPI, 7 PS, and 3 PG species (LC-MS/MS) and the particle and lipid concentrations of 14 lipoprotein subclasses (NMR) as indicated by partial Spearman's rank correlations. **S32**

Figure S10. Associations between the concentrations of 27 individual CE, one Free C, 6 individual DE, 18 individual FFA, and 14 AC species (LC-MS/MS) and the particle and lipid concentrations of 14 lipoprotein subclasses (NMR) as indicated by partial Spearman's rank correlations. **S33**

Figure S11. Associations between the concentrations of 9 individual TG(O)[SIM], 20 TG(O)[NL], one individual ubiquinone, 3 oxidized CE, and 6 oxidized PC/LPC species (LC-MS/MS) and the particle and lipid concentrations of 14 lipoprotein subclasses (NMR) as indicated by partial Spearman's rank correlations. **S34**

Figure S12. Colored SOM component planes for all the 1,018 molecular inputs. **S35**

Supplement Note

Study Populations

Northern Finland Birth Cohorts 1966

The Northern Finland Birth Cohort (NFBC) studies are two longitudinal birth cohorts established to study factors affecting preterm birth and consequent morbidity in the two northernmost provinces of Finland, Oulu and Lapland. The NFBC66 includes 12,058 live births (12,231 children) covering 96% of all eligible births in this region during January – December 1966. Data collection conducted in 2012 at their age of 46 years, including clinical examination and questionnaires.[1] The serum samples (n=5,657) in this study used for NMR spectroscopy and MS analysis were taken after overnight fasting. The research protocols were approved by the Ethics Committee of University of Oulu and the Ethics Committee of Northern Ostrobotnia Hospital District, Finland. More information on the cohort and the data collection can be found at http://www.oulu.fi/nfbc.

Cardiovascular Risk in Young Finns Study

The Cardiovascular Risk in Young Finns Study (YFS) is a population based prospective cohort study conducted with the aim of studying the levels of cardiovascular risk factors in children and adolescents in different parts of the country.[2] The first cross-sectional survey was conducted in 1980. Total sample size was 4,320 boys and girls in 6 age cohorts (aged 3, 6, 9, 12, 15 and 18), these subjects were randomly chosen from the national register. After that, several follow-up studies of this cohort have been conducted. A total of 7,349 participants attended these three generational field studies. Examinations have included comprehensive data collection using questionnaires, physical measurements, and blood tests. The follow-up during 2007 used in this study comprise 2,173 subjects. All serum samples were taken after overnight fasting. All participants gave written informed consent, and the study was approved by the local ethics committees and conducted in accordance with the Declaration of Helsinki. More information on the cohort can be found at https://youngfinnsstudy.utu.fi/index.html.

LC-MS/MS Mass Spectrometry Analysis for lipidomics

The lipid analyses were performed using triple quadruple mass spectrometer (Agilent 6490 QQQ) with an Agilent 1290 series HPLC system and a ZORBAX eclipse plus C18 column (2.1 × 100 mm × 1.8 μm). Solvent A consisted of 50% H_2O/ 30% acetonitrile / 20% isopropanol with 10mM ammonium formate and 5μM medronic acid, while solvent B consisted of 1% H_2O/ 9% acetonitrile / 90% isopropanol with 10mM ammonium formate. Mass spectrometry analysis was performed in a positive ion mode with dynamic

scheduled multiple reaction monitoring (MRM). We modified the methodology to include a dual column setup (in which one of the columns is set to equilibrate while the other is running a sample). The temperature within the column compartment was set to 45 °C with the chromatographic conditions as follows: starting at 15% solvent B and increasing to 50% B over 2.5 min, then quickly ramping to 57% B for 0.1 min. For 6.4 min, %B was increased to 70%, then increased to 93% over 0.1 min and increased to 96% over 1.9 min. The gradient was quickly ramped up to 100% B for 0.1 min and held at 100% B for a further 0.9 min. This was a total run time of 12 min. The column was then brought back down to 15% B for 0.2 min and held for another 0.7 min prior to switching to the alternate column for running the next sample. The column that was being equilibrated was run as follows: 0.9 min of 15% B, 0.1 min increase to 100% B and held for 5 min, decreasing back to 15% B over 0.1 min and held until it was switched for the next sample. We used a 1 µL injection per sample and the following mass spectrometer conditions were used: gas temperature, 150 °C; gas flow rate, 17 L/min; nebulizer, 20 psi; sheath gas temperature, 200 °C; capillary voltage, 3500 V and sheath gas flow, 10 L/min.

The Baker lipidomics platform has been under development for over 15 years. It represents a state of the art, high-throughput lipidomic methodology using reverse-phase liquid chromatography coupled tandem mass spectrometry. Lipid species are measured using polarity switching and dynamic multiple reaction monitoring. Detailed information on the characterization of lipid species is available in Huynh et al.[3] A detailed description of the methodology is highlighted in the Baker Institute-Agilent Application Note.[4] Extensive details of each lipid species, including chromatograms and integration bounds are available in our web portal.[5] We have provided transition, retention times, and collision energy for all measured species and internal standards.

Self-Organizing Maps

All individuals from both cohorts were analyzed together. Inputs were rank transformed to remove scale differences and to mitigate outliers. This was done separately for the cohorts to also remove potential cohort-specific biases. The impact of each lipid variable on the structure of the map was calculated using permutation analysis. Visually, the statistical impact is represented via the intensity of the map colors (Figure 3). Here various key measures from both data domains were considered when assessing the subgroup boundaries. These included, for example, apoB, TG, and lipoprotein subclass particle concentrations from NMR as well as, for example, concentration data for various lipid classes and some individual CE species from LC-MS/MS.

Characteristics of Integrated Data

Firstly, the lipoprotein analysis with NMR is performed directly from the intact serum sample without any sample pretreatments. The LC-MS/MS analysis, however, calls for a lipid extraction procedure, i.e., all the individual lipid molecules in the serum sample need to be isolated into a homogeneous lipid-soluble mixture (Figure 1). Along this process all the lipoprotein-related heterogeneity and information in the original sample is lost and thereby the LC-MS/MS lipid measures (both classes and species) represent total concentrations in all the circulating lipoprotein particles (Figure 2).

Secondly, almost the entire molecular transport in the bloodstream is regulated via a lipoprotein-centered metabolic cascade in which a complicated interplay with various enzymes, lipid transfer proteins and cell surface receptors result in, e.g., the delivery of TG in the VLDL particles, synthesized in the liver, to the adipose tissue. The resultant particles are further modified to LDL particles that, in unfavorable circumstances, can accumulate in the intimal spaces of arteries and cause atherosclerosis. The HDL particles – that do not contain apoB – interact with and are important players in the continuous conversions of the apoB particles. They also have other biological roles, for example, in relation to inflammation.

The path from the site of synthesis to the site of delivery or action for the individual lipid molecules can be extremely tangled with lipoprotein particles. Only a few lipid classes are transported by albumin, e.g., free fatty acids, acylcarnitines, and a portion of lipid species with only one fatty acid chain.[6,7] Thus, almost every metabolic pathway related to lipids in the systemic circulation is mediated by lipoprotein particles. This accentuates the importance of comprehensive lipoprotein data for proper analyses and interpretations of lipidomics data, accomplished here for the first time.

Results and Discussion

Associations between the Lipoprotein Subclasses and Lipid Classes

The entire lipoprotein cascade, as well as the individual particles, are metabolically and biophysically constrained with strong innate correlations.[8] The entirety of systemic lipoprotein metabolism can thus have wide-ranging effects even for correlations between a single lipoprotein subclass and a single lipidomics measure. This makes specific molecular interpretations challenging.

Associations between the Lipoprotein Subclasses and Lipid Species

This kind of opposite behaviour with various CE clusters bears resemblance to total cholesterol. It sums up all the circulating cholesterol with two main components, LDL-C and HDL-C, inherently representing opposing metabolic roles. This has led to the routine use of both LDL-C and HDL-C as key parts of the clinical lipid measurements.[9,10]

Self-Organizing Maps and Population Stratification

We have demonstrated previously that the SOM analysis is a prime method in solving complicated biomedical challenges, for example, in assessing risk for type 1 diabetes complications based on clinical and biochemical data[11] and in conformational analyses of molecular dynamic simulations in relation to the structural features of biological membranes.[12] The key distinction between the SOM analysis and many common unsupervised chemometric techniques, e.g., principal component analysis, is the means to handle multi-dimensional inputs and transform them to a 2-dimensional (human interpretable) map that is fully and directly connected with the unaltered input data (Figure 3).[13] In SOM analyses neither vast numbers of inputs nor individuals pose particular obstacles to the statistical algorithm.

In Figure 6 red color means higher and blue color lower values in relative to the population mean in each map district. The subgroup boundaries are based on the key characteristics of both data domains, including multiple lipoprotein-related measures (NMR) together with some of the lipid classes and species (LC-MS/MS). They should not be taken too strictly but to provide a guide to the human eye to understand and interpret the overall metabolic population characteristics. This is the strength of the SOM analysis.[13]

The compositional information on the lipoprotein subclass particles (L, M, and N) reveals that high circulating triglycerides (A) are associated with TG-enrichment of all circulating lipoprotein particles, including the HDL particles. In fact, compositional TG-enrichment of HDL particles has been indicated to result in higher risk of coronary heart disease, independently of total cholesterol and triglycerides.[14] This is an intriguing finding, also supported by the current results, linking the molecular details of lipoprotein particles to the risk of atherosclerosis. Nonetheless, it is our supposition that the TG-enrichment of the lipoprotein particles is not the primary metabolic cause of the disease risk, but the increased concentrations of circulating total triglycerides.

References

(1) Nordström, T.; Miettunen, J.; Auvinen, J.; Ala-Mursula, L.; Keinänen-Kiukaanniemi, S.; Veijola, J.; Järvelin, M.-R.; Sebert, S.; Männikkö, M. Cohort Profile: 46 Years of Follow-up of the Northern Finland Birth Cohort 1966 (NFBC1966). *Int. J. Epidemiol.* **2022**, *50*, 1786–1787j.
(2) Raitakari, O. T.; Juonala, M.; Rönnemaa, T.; Keltikangas-Järvinen, L.; Räsänen, L.; Pietikäinen, M.; Hutri-Kähönen, N.; Taittonen, L.; Jokinen, E.; Marniemi, J.; Jula, A.; Telama, R.; Kähönen, M.; Lehtimäki, T.; Akerblom, H. K.; Viikari, J. S. A. Cohort Profile: The Cardiovascular Risk in Young Finns Study. *Int. J. Epidemiol.* **2008**, *37*, 1220–1226.
(3) Huynh, K.; Barlow, C. K.; Jayawardana, K. S.; Weir, J. M.; Mellett, N. A.; Cinel, M.; Magliano, D. J.; Shaw, J. E.; Drew, B. G.; Meikle, P. J. High-Throughput Plasma Lipidomics: Detailed Mapping of the Associations with Cardiometabolic Risk Factors. *Cell Chem. Biol.* **2019**, *26*, 71-84.e4.
(4) Huynh, K.; Mellett, N. A.; Duong, T.; Nguyen, A.; Meikle, T. G.; Giles, C.; Meikle, P. J. A Comprehensive, Curated, High-Throughput Method for the Detailed Analysis of the Plasma Lipidome. *Application Note - Agilent.* **2021**, 1-45. https://www.agilent.com/cs/library/applications/an-plasma-lipidomics-6495-lc-ms-ms-5994-3747en-agilent.pdf
(5) https://metabolomics.baker.edu.au/method/
(6) Wiesner, P.; Leidl, K.; Boettcher, A.; Schmitz, G.; Liebisch, G. Lipid Profiling of FPLC-Separated Lipoprotein Fractions by Electrospray Ionization Tandem Mass Spectrometry. *J. Lipid Res.* **2009**, *50*, 574–585.
(7) Dambrova, M.; Makrecka-Kuka, M.; Kuka, J.; Vilskersts, R.; Nordberg, D.; Attwood, M. M.; Smesny, S.; Sen, Z. D.; Guo, A. C.; Oler, E.; Tian, S.; Zheng, J.; Wishart, D. S.; Liepinsh, E.; Schiöth, H. B. Acylcarnitines: Nomenclature, Biomarkers, Therapeutic Potential, Drug Targets, and Clinical Trials. *Pharmacol. Rev.* **2022**, *74*, 506–551.
(8) Ala-Korpela, M.; Zhao, S.; Järvelin, M.-R.; Mäkinen, V.-P.; Ohukainen, P. Apt Interpretation of Comprehensive Lipoprotein Data in Large-Scale Epidemiology: Disclosure of Fundamental Structural and Metabolic Relationships. *Int. J. Epidemiol.* **2022**, *51*, 996–1011.
(9) Goldstein, J. L.; Brown, M. S. A Century of Cholesterol and Coronaries: From Plaques to Genes to Statins. *Cell* **2015**, *161*, 161–172.
(10) Schmidt, A. F.; Joshi, R.; Gordillo-Marañón, M.; Drenos, F.; Charoen, P.; Giambartolomei, C.; Bis, J. C.; Gaunt, T. R.; Hughes, A. D.; Lawlor, D. A.; Wong, A.; Price, J. F.; Chaturvedi, N.; Wannamethee, G.; Franceschini, N.; Kivimaki, M.; Hingorani, A. D.; Finan, C. Biomedical Consequences of Elevated Cholesterol-Containing Lipoproteins and Apolipoproteins on Cardiovascular and Non-Cardiovascular Outcomes. *Commun. Med.* **2023**, *3*, 9.
(11) Mäkinen, V.-P.; Forsblom, C.; Thorn, L. M.; Wadén, J.; Gordin, D.; Heikkilä, O.; Hietala, K.; Kyllönen, L.; Kytö, J.; Rosengård-Bärlund, M.; Saraheimo, M.; Tolonen, N.; Parkkonen, M.; Kaski, K.; Ala-Korpela, M.; Groop, P.-H.; FinnDiane Study Group. Metabolic Phenotypes, Vascular Complications, and Premature Deaths in a Population of 4,197 Patients with Type 1 Diabetes. *Diabetes* **2008**, *57*, 2480–2487.
(12) Hyvönen, M. T.; Hiltunen, Y.; El-Deredy, W.; Ojala, T.; Vaara, J.; Kovanen, P. T.; Ala-Korpela, M. Application of Self-Organizing Maps in Conformational Analysis of Lipids. *J. Am. Chem. Soc.* **2001**, *123*, 810–816.
(13) Gao, S.; Mutter, S.; Casey, A.; Mäkinen, V.-P. Numero: A Statistical Framework to Define Multivariable Subgroups in Complex Population-Based Datasets. *Int. J. Epidemiol.* **2019**, *48*, 369–374.
(14) Kettunen, J.; Holmes, M. V.; Allara, E.; Anufrieva, O.; Ohukainen, P.; Oliver-Williams, C.; Wang, Q.; Tillin, T.; Hughes, A. D.; Kähönen, M.; Lehtimäki, T.; Viikari, J.; Rai-takari, O. T.; Salomaa, V.; Järvelin, M.-R.; Perola, M.; Smith, G. D.; Chaturvedi, N.; Danesh, J.; Angelantonio, E. D.; Butter-worth, A. S.; Ala-Korpela, M. Lipoprotein Signatures of Cho-lesteryl Ester Transfer Protein and HMG-CoA Reductase Inhibition. PLOS Biol. 2019, 17, e3000572.

Funding

The NFBC has been supported, for instance, by EU, Academy of Finland, PREcisE, Joint Programming Initiative a Healthy Diet for a Healthy Life (no. 655), UK Medical Research Council, Biotechnology and Biological Sciences Research Council (MR/S03658X/1), and European Regional Development Fund Grant no. 539/2010 A31592. The Young Finns Study has been financially supported by the Academy of Finland: grants 356405, 322098, 286284, 134309 (Eye), 126925, 121584, 124282, 129378 (Salve), 117797 (Gendi), and 141071 (Skidi); the Social Insurance Institution of Finland; Competitive State Research Financing of the Expert Responsibility area of Kuopio, Tampere and Turku University Hospitals (grant X51001); Juho Vainio Foundation; Paavo Nurmi Foundation; Finnish Foundation for Cardiovascular Research; Finnish Cultural Foundation; The Sigrid Juselius Foundation; Tampere Tuberculosis Foundation; Emil Aaltonen Foundation; Yrjö Jahnsson Foundation; Signe and Ane Gyllenberg Foundation; Diabetes Research Foundation of Finnish Diabetes Association; EU Horizon 2020 (grant 755320 for TAXINOMISIS and grant 848146 for To Aition); European Research Council (grant 742927 for MULTIEPIGEN project); Tampere University Hospital Supporting Foundation; Finnish Society of Clinical Chemistry; the Cancer Foundation Finland; pBETTER4U_EU (Preventing obesity through Biologically and bEhaviorally Tailored inTERventions for you; project number: 101080117); CVDLink (EU grant nro.) and the Jane and Aatos Erkko Foundation. C.G., K.H., and P.J.M. were supported by Investigator grants (2027256, 1197190, and 2009965, respectively) from the National Health and Medical Research Council of Australia. In addition, their work was supported by the Victorian Government's Operational Infrastructure Support Program. M.A.-K. was supported by a research grant from the Sigrid Juselius Foundation, the Finnish Foundation for Cardiovascular Research, and the Research Council of Finland (grant no. 357183).

Abbreviations

AC, Acylcarnitines
ALT, Alanine aminotransferase
APOA1, Apolipoprotein A-I
APOB, Apolipoprotein B
APOB/APOA1, Ratio of apolipoprotein B to apolipoprotein A-I
BMI, Body mass index
C, Cholesterol
C1P, Ceramide-1-phosphates
CE, Cholesteryl esters
Cer(d), Ceramides
Cer(m), Deoxyceramides
cIMT, Carotid artery intima-media thickness
CKD, Chronic kidney disease
CRP, C-reactive protein
DE, Dehydrocholesterol esters (refers to all cholesterol precursors with an additional double bond; see Table S4 for a note)
DG, Diacylglycerols
DHA, Docosahexaenoic acid (22:6)
DHA/FA, Ratio of docosahexaenoic acid to total fatty acids
dhCer, Dihydroceramides
ESTC, Esterified cholesterol
FAW3, Omega-3 fatty acids
FAW3/FA, Ratio of omega-3 fatty acids to total fatty acids
FAW6, Omega-6 fatty acids
FAW6/FA, Ratio of omega-6 fatty acids to total fatty acids
FC, Free cholesterol
FFA, Free fatty acids
GM1, GM1 gangliosides
GM3, GM3 gangliosides
HDL, High-density lipoprotein
HDL-C, Total cholesterol in HDL particles
HDL-TG, Triglycerides in HDL particles
HDL2-C, Total cholesterol in HDL2 particles
HDL3-C, Total cholesterol in HDL3 particles
Hex2Cer, Dihexosylceramides
Hex3Cer, Trihexosylceramides
HexCer, Monohexosylceramides
IDL, Intermediate-density lipoprotein
L, Large (in relation to lipoprotein subclass particle size)

L, Total lipid concentration (in relation to lipoprotein subclasses)
LA, Linoleic acid (18:2)
LA/FA, Ratio of linoleic acid to total fatty acids
LC, Liquid chromatography
LDL, Low-density lipoprotein
LDL-C, Total cholesterol in LDL particles
LDL-TG, Triglycerides in LDL particles
Lp(a), Lipoprotein (a)
LPC, Lysophosphatidylcholines
LPC(O), Lysoalkylphosphatidylcholines
LPC(P), Lysoalkenylphosphatidylcholines (plasmalogens)
LPE, Lysophosphatidylethanolamines
LPE(P), Lysoalkenylphosphatidylethanolamines (plasmalogens)
LPI, Lysophosphatidylinositols
M, Medium (in relation to lipoprotein subclass particle size)
MS, Mass spectrometry
MUFA, Monounsaturated fatty acids
MUFA/FA, Ratio of monounsaturated fatty acids to total fatty acids
NMR, Nuclear magnetic resonance
NFBC66, Northern Finland Birth Cohort 1966
Ox-CE, Oxidised cholesteryl esters
Ox-PC/LPC, Oxidised Phosphatidylcholines and lysophosphatidylcholines
PA, Phosphatidic acids
PC, Phosphatidylcholine and other cholines
PC, Phosphatidylcholines
PC(O), Alkylphosphatidylcholines
PC(P), Alkenylphosphatidylcholines (plasmalogens)
PE, Phosphatidylethanolamines
PE(O), Alkylphosphatidylethanolamines
PE(P), Alkenylphosphatidylethanolamines (plasmalogens)
PG, Phosphatidylglycerols
PI, Phosphatidylinositols
PIP1, Phosphatidylinositol monophosphates
PL, Phospholipids
PS, Phosphatidylserines
PUFA, Polyunsaturated fatty acids
PUFA/FA, Ratio of polyunsaturated fatty acids to total fatty acids
REMNANT-C, Remnant cholesterol, i.e., (non-HDL, non-LDL)-cholesterol
S, Small (in relation to lipoprotein subclass particle size)
S1P, Sphingosine-1-phosphates
SERUM-C, Serum total cholesterol

SERUM-TG, Serum total triglycerides
SFA, Saturated fatty acids
SFA/FA, Ratio of saturated fatty acids to total fatty acids
SHexCer, Sulfatides
SM, Sphingomyelins
SM, Sphingomyelins
SOM, Self-organizing map
Sph, Sphingosines
TG, Triglycerides (NMR)
TG [NL], Triglycerides (LC-MS/MS)
TG [SIM], Triglycerides (LC-MS/MS)
TG(O) [SIM], Alkyldiacylglycerols
TG/PG, Ratio of triglycerides to phosphoglycerides
FA, Total fatty acids
TOTPG, Total phosphoglycerides
UNSAT, Estimated degree of unsaturation
VLDL, very-low-density lipoprotein
VLDL-C, Total cholesterol in VLDL particles
VLDL-TG, Triglycerides in VLDL particles
XL, Very large (in relation to lipoprotein subclass particle size)
XS, Very small (in relation to lipoprotein subclass particle size)
XXL, Extremely large (in relation to lipoprotein subclass particle size)
YFS, Cardiovascular Risk in Young Finns Study

Supplement Tables

Table S1.

Clinical characteristics in NFBC66 and YFS. The values are median [Q1 – Q3].

Clinical characteristic	NFBC66	YFS
Number of participants	5,657	2,173
Number of females (%)	3,168 (56.0 %)	1,190 (54.8 %)
Age (year)	46.6 [46.2 - 47.1]	39.0 [33.0 - 42.0]
BMI (kg/m2)	26.1 [23.5 - 29.3]	25.8 [23.1 - 29.1]
C-reactive protein [CRP] (mg/L)	0.83 [0.45 - 1.70]	0.88 [0.41 - 1.91]
CKD Chien risk	6.55 [4.94 - 9.00]	3.50 [2.2 - 5.23]
Alanine aminotransferase [ALT] (U/L)	25.0 [18.0 -36.0]	14.0 [10.0 - 22.0]
Lipoprotein (a) [Lp(a)] (mg/L)	NA	75.0 [33.0 - 188.0]
Carotid artery intima-media thickness [cIMT] (mm)	NA	0.62 [0.56 – 0.68]

NA refers to not available.

Table S2.

NMR-based lipoprotein measures in NFBC66 and YFS. The values are median [Q1 – Q3].

	NFBC66	YFS
**Lipoprotein subclass concentration measures**		
**Extremely large very-low-density lipoprotein subclass (XXL-VLDL)**		
XXL-VLDL-P (mol/L)	9.14E-11 [5.6E-11 - 1.6E-11]	7.4E-11 [3.7E-11 - 1.5E-10]
XXL-VLDL-L (mmol/L)	0.02 [0.01 - 0.03]	0.016 [0.0079 - 0.0325]
XXL-VLDL-PL (mmol/L)	0.0023 [0.0014 - 0.0042]	0.0018 [0.0008 - 0.0039]
XXL-VLDL-C (mmol/L)	0.0039 [0.0022 - 0.0066]	0.0033 [0.0015 - 0.0067]
XXL-VLDL-CE (mmol/L)	0.0025 [0.0014 - 0.0041]	0.0021 [0.0009 - 0.0041]
XXL-VLDL-FC (mmol/L)	0.0014 [0.0008 - 0.0026]	0.0012 [0.0005 - 0.0027]
XXL-VLDL-TG (mmol/L)	0.0134 [0.0083 - 0.0233]	0.0108 [0.0056 - 0.022]
**Very large very-low-density lipoprotein subclass (XL-VLDL)**		
XL-VLDL-P (mol/L)	4.5E-10 [2.0E-10 - 9.4E-10]	3.7E-10 [1.3E-10 - 8.6E-10]
XL-VLDL-L (mmol/L)	0.04 [0.02 - 0.09]	0.036 [0.013 - 0.085]
XL-VLDL-PL (mmol/L)	0.0083 [0.0041 - 0.016]	0.007 [0.002 - 0.015]
XL-VLDL-C (mmol/L)	0.0095 [0.0049 - 0.0182]	0.009 [0.003 - 0.019]
XL-VLDL-CE (mmol/L)	0.0046 [0.0021 - 0.0093]	0.005 [0.001 - 0.01]
XL-VLDL-FC (mmol/L)	0.0049 [0.0028 - 0.009]	0.004 [0.002 - 0.009]
XL-VLDL-TG (mmol/L)	0.0268 [0.011 - 0.0576]	0.021 [0.007 - 0.052]
**Large very-low-density lipoprotein subclass (L-VLDL)**		
L-VLDL-P (mol/L)	3.3E-9 [1.6E-9 - 6.4E-9]	2.8E-9 [1.3E-9 - 5.8E-9]
L-VLDL-L (mmol/L)	0.19 [0.093 - 0.371]	0.17 [0.08 - 0.34]
L-VLDL-PL (mmol/L)	0.04 [0.02 - 0.07]	0.03 [0.02 - 0.06]
L-VLDL-C (mmol/L)	0.04 [0.02 - 0.08]	0.04 [0.02 - 0.08]
L-VLDL-CE (mmol/L)	0.02 [0.01 - 0.04]	0.02 [0.01 - 0.05]
L-VLDL-FC (mmol/L)	0.02 [0.01 - 0.04]	0.02 [0.01 - 0.04]
L-VLDL-TG (mmol/L)	0.11 [0.05 - 0.22]	0.09 [0.04 - 0.19]
**Medium very-low-density lipoprotein subclass (M-VLDL)**		
M-VLDL-P (mol/L)	1.5E-8 [9.7E-9 - 2.3E-8]	1.4E-8 [9.2E-9 - 2.2E-8]
M-VLDL-L (mmol/L)	0.5 [0.33 - 0.79]	0.48 [0.32 - 0.76]
M-VLDL-PL (mmol/L)	0.11 [0.07 - 0.16]	0.1 [0.07 - 0.15]
M-VLDL-C (mmol/L)	0.16 [0.11 - 0.23]	0.15 [0.11 - 0.23]
M-VLDL-CE (mmol/L)	0.1 [0.07 - 0.13]	0.1 [0.07 - 0.13]
M-VLDL-FC (mmol/L)	0.06 [0.04 - 0.09]	0.06 [0.04 - 0.09]
M-VLDL-TG (mmol/L)	0.24 [0.15 - 0.4]	0.22 [0.14 - 0.38]

**Small very-low-density lipoprotein subclass (S-VLDL)**		
S-VLDL-P (mol/L)	3.0E-8 [2.2E-8 - 4.0E-8]	2.9E-8 [2.2E-8 - 3.9E-8]
S-VLDL-L (mmol/L)	0.6 [0.44 - 0.79]	0.59 [0.44 - 0.78]
S-VLDL-PL (mmol/L)	0.14 [0.1 - 0.18]	0.13 [0.1 - 0.17]
S-VLDL-C (mmol/L)	0.26 [0.2 - 0.32]	0.25 [0.19 - 0.31]
S-VLDL-CE (mmol/L)	0.17 [0.13 - 0.21]	0.16 [0.12 - 0.2]
S-VLDL-FC (mmol/L)	0.09 [0.06 - 0.11]	0.09 [0.06 - 0.11]
S-VLDL-TG (mmol/L)	0.2 [0.14 - 0.29]	0.21 [0.14 - 0.3]
**Very small very-low-density lipoprotein subclass (XS-VLDL)**		
XS-VLDL-P (mol/L)	4.7E-8 [3.9E-8 - 5.5E-8]	4.5E-8 [3.7E-8 - 5.3E-8]
XS-VLDL-L (mmol/L)	0.6 [0.51 - 0.71]	0.57 [0.48 - 0.68]
XS-VLDL-PL (mmol/L)	0.18 [0.16 - 0.22]	0.17 [0.15 - 0.2]
XS-VLDL-C (mmol/L)	0.32 [0.27 - 0.37]	0.29 [0.24 - 0.34]
XS-VLDL-CE (mmol/L)	0.22 [0.18 - 0.25]	0.2 [0.16 - 0.23]
XS-VLDL-FC (mmol/L)	0.1 [0.09 - 0.12]	0.09 [0.08 - 0.11]
XS-VLDL-TG (mmol/L)	0.1 [0.08 - 0.13]	0.11 [0.08 - 0.14]
**Intermediate-density lipoprotein subclass (IDL)**		
IDL-P (mol/L)	1.4E-7 [1.2E-7 - 1.6E-7]	1.3E-7 [1.1E-7 - 1.5E-7]
IDL-L (mmol/L)	1.4 [1.2 - 1.63]	1.3 [1.11 - 1.52]
IDL-PL (mmol/L)	0.37 [0.32 - 0.43]	0.35 [0.3 - 0.41]
IDL-C (mmol/L)	0.92 [0.78 - 1.08]	0.83 [0.7 - 0.97]
IDL-CE (mmol/L)	0.66 [0.57 - 0.77]	0.59 [0.5 - 0.69]
IDL-FC (mmol/L)	0.26 [0.22 - 0.3]	0.24 [0.2 - 0.28]
IDL-TG (mmol/L)	0.11 [0.09 - 0.13]	0.11 [0.09 - 0.15]
**Large low-density lipoprotein subclass (L-LDL)**		
L-LDL-P (mol/L)	2.3E-7 [2.0E-7 - 2.7E-7]	2.1E-7 [1.8E-7 - 2.5E-7]
L-LDL-L (mmol/L)	1.63 [1.39 - 1.92]	1.5 [1.27 - 1.78]
L-LDL-PL (mmol/L)	0.4 [0.35 - 0.46]	0.37 [0.33 - 0.43]
L-LDL-C (mmol/L)	1.13 [0.95 - 1.35]	1.02 [0.85 - 1.22]
L-LDL-CE (mmol/L)	0.82 [0.68 - 0.99]	0.73 [0.6 - 0.88]
L-LDL-FC (mmol/L)	0.31 [0.27 - 0.36]	0.29 [0.24 - 0.34]
L-LDL-TG (mmol/L)	0.09 [0.08 - 0.11]	0.1 [0.08 - 0.13]
**Medium low-density lipoprotein subclass (M-LDL)**		
M-LDL-P (mol/L)	1.8E-7 [1.5E-7 - 2.2E-7]	1.6E-7 [1.4E-7 - 2.0E-7]
M-LDL-L (mmol/L)	0.94 [0.78 - 1.13]	0.84 [0.7 - 1.02]
M-LDL-PL (mmol/L)	0.24 [0.21 - 0.28]	0.23 [0.2 - 0.26]
M-LDL-C (mmol/L)	0.66 [0.54 - 0.8]	0.57 [0.46 - 0.7]
M-LDL-CE (mmol/L)	0.48 [0.38 - 0.6]	0.41 [0.32 - 0.52]
M-LDL-FC (mmol/L)	0.17 [0.15 - 0.2]	0.16 [0.14 - 0.18]
M-LDL-TG (mmol/L)	0.04 [0.03 - 0.05]	0.05 [0.04 - 0.06]

**Small low-density lipoprotein subclass (S-LDL)**		
S-LDL-P (mol/L)	2.1E-7 [1.8E-7 - 2.5E-7]	1.9E-7 [1.6E-7 - 2.3E-7]
S-LDL-L (mmol/L)	0.59 [0.5 - 0.71]	0.53 [0.44 - 0.64]
S-LDL-PL (mmol/L)	0.17 [0.15 - 0.2]	0.16 [0.14 - 0.18]
S-LDL-C (mmol/L)	0.39 [0.32 - 0.48]	0.34 [0.27 - 0.42]
S-LDL-CE (mmol/L)	0.29 [0.23 - 0.36]	0.24 [0.19 - 0.31]
S-LDL-FC (mmol/L)	0.11 [0.09 - 0.12]	0.1 [0.08 - 0.11]
S-LDL-TG (mmol/L)	0.027 [0.021 - 0.035]	0.03 [0.02 - 0.04]
**Very large high-density lipoprotein subclass (XL-HDL)**		
XL-HDL-P (mol/L)	2.9E-7 [2.4E-7 - 5.9E-7]	3.7E-7 [2.3E-7 - 5.6E-7]
XL-HDL-L (mmol/L)	0.39 [0.24 - 0.6]	0.37 [0.23 - 0.57]
XL-HDL-PL (mmol/L)	0.18 [0.09 - 0.3]	0.19 [0.1 - 0.3]
XL-HDL-C (mmol/L)	0.19 [0.13 - 0.28]	0.17 [0.11 - 0.25]
XL-HDL-CE (mmol/L)	0.14 [0.09 - 0.2]	0.12 [0.07 - 0.18]
XL-HDL-FC (mmol/L)	0.05 [0.03 - 0.08]	0.05 [0.03 - 0.08]
XL-HDL-TG (mmol/L)	0.02 [0.01 - 0.02]	0.02 [0.01 - 0.02]
**Large high-density lipoprotein subclass (L-HDL)**		
L-HDL-P	1.3E-6 [3.2E-7 - 1.8E-6]	1.3E-6 [8.8E-7 - 1.8E-6]
L-HDL-L (mmol/L)	0.81 [0.52 - 1.12]	0.82 [0.54 - 1.14]
L-HDL-PL (mmol/L)	0.41 [0.28 - 0.54]	0.41 [0.29 - 0.55]
L-HDL-C (mmol/L)	0.36 [0.2 - 0.53]	0.38 [0.23 - 0.55]
L-HDL-CE (mmol/L)	0.28 [0.16 - 0.41]	0.29 [0.18 - 0.42]
L-HDL-FC (mmol/L)	0.08 [0.04 - 0.12]	0.08 [0.04 - 0.12]
L-HDL-TG (mmol/L)	0.04 [0.03 - 0.05]	0.03 [0.02 - 0.05]
**Medium high-density lipoprotein subclass (M-HDL)**		
M-HDL-P (mol/L)	2.4E-6 [2.1E-6 - 2.7E-6]	2.5E-6 [2.2E-6 - 2.8E-6]
M-HDL-L (mmol/L)	1.03 [0.91 - 1.16]	1.04 [0.92 - 1.21]
M-HDL-PL (mmol/L)	0.47 [0.42 - 0.52]	0.47 [0.42 - 0.55]
M-HDL-C (mmol/L)	0.52 [0.45 - 0.59]	0.52 [0.45 - 0.61]
M-HDL-CE (mmol/L)	0.42 [0.36 - 0.48]	0.42 [0.36 - 0.49]
M-HDL-FC (mmol/L)	0.1 [0.08 - 0.11]	0.1 [0.08 - 0.12]
M-HDL-TG (mmol/L)	0.05 [0.04 - 0.06]	0.05 [0.04 - 0.06]
**Small high-density lipoprotein subclass (S-HDL)**		
S-HDL-P (mol/L)	5.5E-6 [5.1E-6 - 5.9E-6]	5.4E-6 [5.0E-6 -5.9E-6]
S-HDL-L (mmol/L)	1.23 [1.14 - 1.32]	1.2 [1.1 - 1.31]
S-HDL-PL (mmol/L)	0.64 [0.59 - 0.7]	0.64 [0.59 - 0.71]
S-HDL-C (mmol/L)	0.54 [0.49 - 0.59]	0.51 [0.45 - 0.57]
S-HDL-CE (mmol/L)	0.41 [0.36 - 0.46]	0.38 [0.33 - 0.44]
S-HDL-FC (mmol/L)	0.13 [0.12 - 0.14]	0.13 [0.12 - 0.14]
S-HDL-TG (mmol/L)	0.05 [0.04 - 0.06]	0.04 [0.03 - 0.06]

**Lipoprotein subclass lipid compositions**		
**Lipid percentages of extremely large very-low-density lipoprotein subclass (XXL-VLDL)**		
XXL-VLDL-PL %	12.02 [11.03 - 12.74]	11.28 [9.83 - 12.18]
XXL-VLDL-C %	19.06 [16.72 - 21.48]	20.08 [17.52 - 22.69]
XXL-VLDL-CE %	12.11 [9.49 - 14.62]	12.49 [9.66 - 15.39]
XXL-VLDL-FC %	7.21 [6.1 - 8.02]	7.7 [6.41 - 8.47]
XXL-VLDL-TG %	69.02 [66.73 - 71.37]	68.74 [66.18 - 71.59]
**Lipid percentage of very large very-low-density lipoprotein subclass (XL-VLDL)**		
XL-VLDL-PL %	17.84 [16.72 - 19.86]	17.36 [16.49 - 19.24]
XL-VLDL-C %	21.01 [18.18 - 24.85]	23.41 [20.42 - 26.94]
XL-VLDL-CE %	10.37 [8.43 - 12.4]	12.07 [9.87 - 14.29]
XL-VLDL-FC %	10.48 [9.14 - 12.87]	10.99 [9.79 - 13.16]
XL-VLDL-TG %	61.14 [55.64 - 64.66]	59.16 [54.43 - 62.37]
**Lipid percentage of large very-low-density lipoprotein subclass (L-VLDL)**		
L-VLDL-PL %	18.96 [18.37 - 19.96]	18.8 [18.24 - 19.87]
L-VLDL-C %	22.51 [20.62 - 24.75]	24.73 [22.66 - 27.07]
L-VLDL-CE %	12.18 [10.37 - 14.38]	14.3 [12.05 - 16.75]
L-VLDL-FC %	10.54 [9.61 - 11.19]	10.75 [9.22 - 11.68]
L-VLDL-TG %	58.39 [55.61 - 60.55]	56.3 [53.46 - 58.61]
**Lipid percentage of medium very-low-density lipoprotein subclass (M-VLDL)**		
M-VLDL-PL %	21.08 [20.35 - 21.91]	21.03 [20.33 - 21.85]
M-VLDL-C %	30.48 [27.73 - 33.94]	31.78 [28.95 - 35.08]
M-VLDL-CE %	19.15 [16.12 - 22.89]	20.17 [17.16 - 23.75]
M-VLDL-FC %	11.48 [10.88 - 11.88]	11.68 [11.01 - 12.17]
M-VLDL-TG %	48.4 [44.19 - 51.87]	47.13 [43.04 - 50.55]
**Lipid percentage of small very-low-density lipoprotein subclass (S-VLDL)**		
S-VLDL-PL %	22.63 [21.88 - 23.49]	22.44 [21.69 - 23.44]
S-VLDL-C %	42.91 [39.19 - 46.8]	41.39 [37.86 - 45.63]
S-VLDL-CE %	28.64 [25.07 - 32.39]	27.02 [23.73 - 31.11]
S-VLDL-FC %	14.18 [13.73 - 14.62]	14.24 [13.77 - 14.8]
S-VLDL-TG %	34.13 [30.1 - 38.31]	35.73 [31.52 - 39.8]
**Lipid percentage of very small very-low-density lipoprotein subclass (XS-VLDL)**		
XS-VLDL-PL %	30.56 [29.43 - 31.62]	30.47 [28.92 - 32.08]
XS-VLDL-C %	52.85 [50.39 - 55.04]	50.39 [47.31 - 53.17]
XS-VLDL-CE %	36.04 [33.77 - 38.15]	34.13 [31.3 - 36.82]
XS-VLDL-FC %	16.77 [16.16 - 17.32]	16.27 [15.62 - 16.84]
XS-VLDL-TG %	16.48 [14.12 - 19.32]	18.97 [15.99 - 22.32]
**Lipid percentage of intermediate-density lipoprotein subclass (IDL)**		
IDL-PL %	26.75 [26.34 - 27.16]	27.01 [26.54 - 27.55]

IDL-C %	65.76 [64.57 - 66.69]	63.95 [62.33 - 65.21]
IDL-CE %	47.18 [46.05 - 48.19]	45.44 [44.03 - 46.64]
IDL-FC %	18.62 [18 - 19.05]	18.57 [17.92 - 19.05]
IDL-TG %	7.48 [6.53 - 8.73]	8.96 [7.69 - 10.5]
**Lipid percentage of large low-density lipoprotein subclass (L-LDL)**		
L-LDL-PL %	24.69 [24.05 - 25.44]	25.02 [24.26 - 25.86]
L-LDL-C %	69.56 [68.32 - 70.5]	68.07 [66.48 - 69.34]
L-LDL-CE %	50.45 [49.02 - 51.63]	48.87 [47.17 - 50.15]
L-LDL-FC %	19.2 [18.64 - 19.68]	19.29 [18.65 - 19.82]
L-LDL-TG %	5.67 [4.97 - 6.55]	6.72 [5.84 - 8.03]
**Lipid percentage of medium low-density lipoprotein subclass (M-LDL)**		
M-LDL-PL %	25.68 [24.55 - 27.1]	26.75 [25.29 - 28.32]
M-LDL-C %	69.79 [67.96 - 71.11]	67.63 [65.24 - 69.47]
M-LDL-CE %	51.21 [48.67 - 53.17]	48.66 [45.54 - 51.21]
M-LDL-FC %	18.55 [17.71 - 19.52]	18.84 [17.96 - 19.99]
M-LDL-TG %	4.48 [3.85 - 5.27]	5.51 [4.63 - 6.8]
**Lipid percentage of small low-density lipoprotein subclass (S-LDL)**		
S-LDL-PL %	29.25 [27.73 - 31.22]	29.72 [27.82 - 31.96]
S-LDL-C %	66.19 [63.9 - 67.8]	64.78 [61.82 - 66.92]
S-LDL-CE %	48.34 [45.37 - 50.5]	46.6 [43.08 - 49.48]
S-LDL-FC %	17.81 [17.07 - 18.69]	18.0 [17.18 - 19.02]
S-LDL-TG %	4.49 [3.85 - 5.37]	5.39 [4.42 - 6.75]
**Lipid percentage of very large high-density lipoprotein subclass (XL-HDL)**		
XL-HDL-PL %	48.05 [40.45 - 51.79]	51.15 [44.61 - 55.44]
XL-HDL-C %	47.92 [44.52 - 53.42]	45.23 [40.98 - 50.27]
XL-HDL-CE %	34.69 [31.31 - 40.03]	31.27 [27.37 - 35.6]
XL-HDL-FC %	13.31 [12.54 - 14.07]	14.04 [12.94 - 15.22]
XL-HDL-TG %	3.97 [2.97 - 6.32]	3.71 [2.64 - 6]
**Lipid percentage of large high-density lipoprotein subclass (L-HDL)**		
L-HDL-PL %	50.44 [47.76 - 54.01]	50.25 [47.71 - 53.69]
L-HDL-C %	45.06 [40.16 - 48.17]	45.76 [41.72 - 48.42]
L-HDL-CE %	35.43 [32.32 - 37.51]	36.05 [33.48 - 37.85]
L-HDL-FC %	9.69 [7.96 - 10.74]	9.74 [8.16 - 10.73]
L-HDL-TG %	4.65 [3.75 - 6.08]	4.06 [3.19 - 5.23]
**Lipid percentage of medium high-density lipoprotein subclass (M-HDL)**		
M-HDL-PL %	45.41 [44.79 - 46.15]	45.5 [44.82 - 46.22]
M-HDL-C %	50.16 [48.45 - 51.5]	49.91 [48.24 - 51.24]
M-HDL-CE %	40.62 [39.1 - 41.81]	40.28 [38.78 - 41.58]
M-HDL-FC %	9.59 [9.23 - 9.87]	9.6 [9.25 - 9.96]
M-HDL-TG %	4.39 [3.46 - 5.58]	4.58 [3.72 - 5.7]

**Lipid percentage of small high-density lipoprotein subclass (S-HDL)**		
S-HDL-PL %	52.67 [50.1 - 55.06]	54.05 [51.32 - 56.69]
S-HDL-C %	43.69 [41.26 - 46.16]	42.17 [39.42 - 45.04]
S-HDL-CE %	33.25 [30.61 - 36.08]	31.65 [28.51 - 34.66]
S-HDL-FC %	10.33 [9.91 - 10.76]	10.6 [10.19 - 11.04]
S-HDL-TG %	3.65 [2.92 - 4.52]	3.64 [2.88 - 4.59]
**Lipoprotein particle size (nm)**		
VLDL-D	36.02 [35.15 - 37.09]	35.96 [35.09 - 37.04]
LDL-D	23.57 [23.51 - 23.65]	23.63 [23.55 - 23.71]
HDL-D	9.93 [9.72 - 10.16]	9.95 [9.76 - 10.16]
**Cholesterol (mmol/L)**		
SERUM-C	5.62 [4.97 - 6.35]	5.16 [4.56 - 5.92]
VLDL-C	0.8 [0.63 - 1.02]	0.76 [0.59 - 0.98]
REMNANT-C	1.74 [1.45 - 2.09]	1.6 [1.32 - 1.94]
LDL-C	2.18 [1.81 - 2.63]	1.93 [1.58 - 2.33]
Non-HDL cholesterol	3.93 [3.3 - 4.69]	3.53 [2.92 - 4.24]
HDL-C	1.63 [1.36 - 1.92]	1.6 [1.34 - 1.9]
HDL2-C	1.1 [0.84 - 1.37]	1.08 [0.83 - 1.36]
HDL3-C	0.53 [0.51 - 0.55]	0.52 [0.5 - 0.55]
Esterified cholesterol	4.01 [3.53 - 4.54]	3.58 [3.13 - 4.13]
Free cholesterol	1.61 [1.43 - 1.82]	1.59 [1.42 - 1.8]
**Glycerides and phospholipids (mmol/L)**		
SERUM-TG	1.13 [0.83 - 1.61]	1.11 [0.81 - 1.61]
VLDL-TG	0.69 [0.44 - 1.13]	0.66 [0.43 - 1.08]
LDL-TG	0.16 [0.13 - 0.2]	0.18 [0.14 - 0.23]
HDL-TG	0.15 [0.13 - 0.18]	0.14 [0.12 - 0.18]
Total phosphoglycerides	2.38 [2.12 - 2.65]	2.42 [2.15 - 2.76]
Ratio of triglycerides to phosphoglycerides	0.55 [0.42 - 0.72]	0.49 [0.4 - 0.64]
Phosphatidylcholine and other cholines	2.41 [2.17 - 2.67]	2.24 [2.01 - 2.57]
Sphingomyelins	0.58 [0.53 - 0.64]	0.54 [0.48 - 0.6]
Total cholines	2.97 [2.69 - 3.25]	2.75 [2.49 - 3.09]
**Apolipoprotein**		
Apolipoprotein A-I (g/L)	1.73 [1.59 - 1.89]	1.66 [1.52 - 1.84]
Apolipoprotein B (g/L)	1.03 [0.88 - 1.21]	0.96 [0.82 - 1.13]
APOB/APOA-I	0.6 [0.49 - 0.72]	0.57 [0.48 - 0.69]

**Total fatty acids and saturation measures**		
Total fatty acids (mmol/L)	13.95 [12.59 - 15.63]	13.17 [11.55 - 15.31]
Estimated degree of unsaturation	1.17 [1.13 - 1.21]	1.15 [1.11 - 1.18]
**Fatty acids**		
DHA (mmol/L)	0.19 [0.16 - 0.23]	0.23 [0.19 - 0.28]
LA (mmol/L)	3.68 [3.33 - 4.07]	3.33 [2.96 - 3.77]
**Fatty acids by saturation measures**		
Omega-3 fatty acids (mmol/L)	0.6 [0.5 - 0.72]	0.56 [0.46 - 0.7]
Omega-6 fatty acids (mmol/L)	4.54 [4.11 - 5]	4.11 [3.67 - 4.67]
PUFA (mmol/L)	5.17 [4.66 - 5.69]	4.69 [4.17 - 5.34]
MUFA (mmol/L)	3.55 [3.04 - 4.24]	3.5 [2.97 - 4.33]
SFA (mmol/L)	5.22 [4.71 - 5.84]	4.93 [4.3 - 5.75]
**Fatty acids (%)**		
DHA/FA	1.37 [1.16 - 1.63]	1.74 [1.52 - 2.01]
LA/FA	26.46 [24.88 - 27.9]	25.28 [23.15 - 27.22]
FAW3/FA	4.24 [3.66 - 4.99]	4.23 [3.65 - 4.91]
FAW6/FA	32.62 [30.8 - 34.16]	31.38 [29.13 - 33.27]
PUFA/FA	36.85 [34.82 - 38.83]	35.66 [33.31 - 37.79]
MUFA/FA	25.7 [23.67 - 27.86]	26.89 [24.99 - 28.85]
SFA/FA	37.43 [36.09 - 38.75]	37.46 [36.23 - 38.72]

Table S3.

LC-MS/MS lipidomics measures for the total concentrations of the circulating lipid classes (μmol/L) in NFBC66 and YFS. The values are median [Q1 – Q3]. The clusters refer to Figure 4 in the main text.

	NFBC66	YFS
**Cluster no.1 (mol %)**	**1.1 %**	**1.1 %**
Deoxyceramide [Cer(m)]	0.49 [0.36 - 0.69]	0.41 [0.29 - 0.60]
Phosphatidylinositol [PI]	73.06 [61.12 - 87.26]	70.22 [56.31 - 85.44]
Phosphatidylethanolamine [PE]	26.12 [19.75 - 35.19]	26.12 [18.27 - 38.06]
**Cluster no.2 (mol %)**	**0.11 %**	**0.11 %**
Ceramide [Cer(d)]	9.95 [8.21 - 11.9]	8.96 [6.77 - 11.18]
Dihydroceramide [dhCer]	0.67 [0.55 - 0.81]	0.59 [0.46 - 0.75]
**Cluster no.3 (mol %)**	**21.1 %**	**20.2 %**
Phosphatidylcholine [PC]	1901.35 [1667.36 - 2133.32]	1964.87 [1656.30 - 2210.23]
**Cluster no.4 (mol %)**	**0.05 %**	**0.05 %**
Oxidised PC/LPC [Ox-PC/LPC]	0.12 [0.10 - 0.14]	0.11 [0.09 - 0.14]
Lysophosphatidylinositol [LPI]	1.31 [1.08 - 1.57]	1.33 [1.02 - 1.66]
Alkyldiacylglycerol [TG(O) [SIM]]	3.12 [2.69 - 3.65]	3.06 [2.63 - 3.63]
**Cluster no.5 (mol %)**	**16.3 %**	**15.1 %**
Diacylglycerol [DG]	88.72 [66.67 - 124.30]	84.87 [58.30 - 129.23]
Triacylglycerol [TG [NL]]	1232.4 [938.6 – 1624.2]	1299.8 [1028.1 – 1644.1]
Phosphatidylglycerol [PG]	0.49 [0.36 - 0.67]	0.51 [0.38 - 0.71]
**Cluster no.6 (mol %)**	**13.1 %**	**14.0 %**
Ubiquinone	1.00 [0.74 - 1.18]	0.77 [0.54 - 1.03]
Oxidised CE [Ox-CE]	5.45 [4.27 - 7.26]	4.93 [3.76 - 6.42]
Free cholesterol [Free C]	933.79 [828.36 - 1044.57]	856.67 [725.81 - 979.82]
Sphingomyelin [SM]	372.52 [317.91 - 428.45]	342.5 [270.27 - 400.84]
**Cluster no.7 (mol %)**	**1.8 %**	**1.9 %**
Lysophosphatidylethanolamine [LPE]	4.53 [3.76 - 5.48]	4.82 [3.65 - 6.11]
Lysophosphatidylcholine [LPC]	166.35 [136.85 - 200.61]	156.44 [117.17 - 195.57]
Phosphatidic acid [PA]	0.095 [0.083 - 0.11]	0.095 [0.074 - 0.12]
**Cluster no.8 (mol %)**	**0.01 %**	**0.02 %**
Sphingosine [Sph]	0.13 [0.12 - 0.14]	0.12 [0.11 - 0.13]
Sphingosine-1-phosphate [S1P]	0.79 [0.70 - 0.89]	0.73 [0.57 - 0.90]
Phosphatidylserine [PS]	1.04 [0.79 - 1.42]	0.26 [0.21 - 0.32]
**Cluster no.9 (mol %)**	**3.7 %**	**4.4 %**
Lysoalkenylphosphatidylethanolamine (plasmalogen) [LPE(P)]	0.18 [0.15 - 0.22]	0.17 [0.13 - 0.23]
Acylcarnitine [AC]	0.33 [0.27 - 0.41]	0.50 [0.37 - 0.67]

Free fatty acid [FFA]	389.05 [316.51 - 479.84]	306.59 [249.89 - 384.32]
Phosphatidylinositol monophosphate [PIP1]	0.052 [0.043 - 0.059]	0.047 [0.037 - 0.056]
**Cluster no.10 (mol %)**	**1.2 %**	**1.2 %**
Alkenylphosphatidylethanolamine (plasmalogen) [PE(P)]	39.42 [32.16 - 47.76]	35.79 [27.04 - 45.51]
Alkylphosphatidylethanolamine [PE(O)]	3.59 [2.86 - 4.56]	3.26 [2.45 - 4.21]
Sulfatide [ShexCer]	0.68 [0.56 - 0.81]	0.58 [0.45 - 0.71]
Alkylphosphatidylcholine [PC(O)]	63.87 [54.12 - 75.20]	63.29 [50.47 - 76.66]
GM3 ganglioside [GM3]	2.58 [2.16 - 3.05]	2.40 [1.93 - 2.89]
**Cluster no.11 (mol %)**	**41.0 %**	**41.0 %**
Cholesteryl ester [CE]	3860.76 [3423.71 - 4329.89]	3661.83 [3198.54 - 4111.50]
**Cluster no.12 (mol %)**	**0.45 %**	**0.45 %**
Alkenylphosphatidylcholine (plasmalogen) [PC(P)]	40.54 [33.26 - 48.97]	40.03 [30.82 - 49.90]
Trihexosylcermide [Hex3Cer]	1.39 [1.16 - 1.66]	1.37 [1.09 - 1.65]
Ceramide-1-phosphate [C1P]	0.005 [0.004 - 0.007]	0.005 [0.004 - 0.006]
**Cluster no.13 (mol %)**	**0.12 %**	**0.13 %**
Dihexosylceramide[Hex2Cer]	3.54 [2.88 - 4.35]	3.65 [2.83 - 4.52]
Monohexosylceramide [HexCer]	4.91 [4.06 - 5.92]	4.62 [3.77 - 5.59]
Lysoalkylphosphatidylcholine [LPC(O)]	2.51 [2.10 - 2.96]	2.24 [1.69 - 2.78]
Lysoalkenylphosphatidylcholine (plasmalogen) [LPC(P)]	0.56 [0.46 - 0.68]	0.53 [0. 39 - 0.68]
GM1 ganglioside [GM1]	0.012 [0.010 - 0.015]	0.012 [0.010 - 0.015]
**Cluster no.14 (mol %)**	**0.13 %**	**0.12 %**
Dehydrocholesterol ester [DE]	10.96 [9.18 - 13.06]	11.27 [8.52 - 14.37]

Table S5.

Comparison of the relative abundances (mol-%) of the most common serum lipid classes in lipidomics studies.

Lipid species	(1)	(2)	(3)	(4)	(5)	(6)	(7)	(8)	(9)	NFBC66 LC-MS/MS	YFS LC-MS/MS
**CE**	29.41%	78.13%	37.58%	52%	30.00%	55.50%	50.40%	36.85%	39.40%	**41.20%**	**40.90%**
**PC**	27.31%	12.09%	35.49%	29%	15.80%	9.30%	13.10%	24.62%	30.90%	**20.20%**	**21.10%**
**TG**	12.61%	3.80%	6.54%	9%	39.70%	7.60%	8.00%	13.20%	15.50%	**14.00%**	**15.20%**
**Free C**						9.60%	12.70%	10.30%		**10%**	**9.40%**
**FFA**	21.01%				3.17%			2.67%	13.00%	**4.40%**	**3.70%**
**SM**	3.99%	4.63%	2.09%	8%	3.17%	4.80%	5.00%	3.79%	8.00%	**4.00%**	**3.60%**
**LPC**	2.10%	0.82%	10.44%	1.90%	3.57%	3.70%	2.60%	1.29%	3.80%	**1.80%**	**1.70%**
**DG**	0.29%	0.06%	1.39%	0.70%	1.43%	1.90%	0.40%	0.65%	0.50%	**1.10%**	**1.10%**
**PI**	0.34%		0.11%	0.60%	0.67%	1.00%	0.70%	0.39%		**0.80%**	**0.80%**
**PC(O)**		0.27%	5.43%		0.64%	0.90%	1.30%			**0.70%**	**0.70%**
**PC(P)**	0.84%				0.47%	1.00%	0.50%			**0.40%**	**0.40%**
**PE(P)**					0.54%	4.00%	4.20%			**0.40%**	**0.40%**
**PE**	1.68%	0.19%	0.15%	0.60%	0.23%	0.40%	0.60%	5.43%	1.70%	**0.3%**	**0.3%**
**DE**					0.08%					**0.11%**	**0.10%**
**Cer(d)**	0.04%		0.14%		0.09%	0.20%	0.10%	0.15%	0.10%	**0.09%**	**0.09%**
**Others**	0.38%	0.02%	0.64%	0.11%	0.41%	0.10%	0.20%	0.68%	0.10%	**0.36%**	**0.45%**

The NFBC66 and YFS LC-MS/MS results correspond to those given in Figure 2B. The numbers in the table refer to the following studies:

(1) Eichelmann F., et al. Deep Lipidomics in Human Plasma: Cardiometabolic Disease Risk and Effect of Dietary Fat Modulation. Circulation. 2022;146:21-35

(2) Fernandez C., et al. Plasma lipid composition and risk of developing cardiovascular disease. PLoS One. 2013;8:e71846

(3) Aristizabal-Henao J. J., et al. Nontargeted lipidomics of novel human plasma reference materials: hypertriglyceridemic, diabetic, and African-American. Anal Bioanal Chem. 2020;412:7373-7380

(4) Nilsson A. K., et al. Lipid profiling of suction blister fluid: comparison of lipids in interstitial fluid and plasma. Lipids Health Dis. 2019;18:164

(5) Yap C. X., et al. Interactions between the lipidome and genetic and environmental factors in autism. Nat Med. 2023;29:936-949

(6) Huynh K., et al. High-Throughput Plasma Lipidomics: Detailed Mapping of the Associations with Cardiometabolic Risk Factors. Cell Chem Biol. 2019;26:71-84.e4

(7) Bowden J. A., et al. Harmonizing lipidomics: NIST interlaboratory comparison exercise for lipidomics using SRM 1950-Metabolites in Frozen Human Plasma. J Lipid Res. 2017;58:2275-2288

(8) Quehenberger O., et al. Lipidomics reveals a remarkable diversity of lipids in human plasma. J Lipid Res. 2010;51:3299-305

(9) Ghorasaini M., et al. Cross-Laboratory Standardization of Preclinical Lipidomics Using Differential Mobility Spectrometry and Multiple Reaction Monitoring. Anal Chem. 2021;93:16369-16378

Supplement Figures

Figure S1.

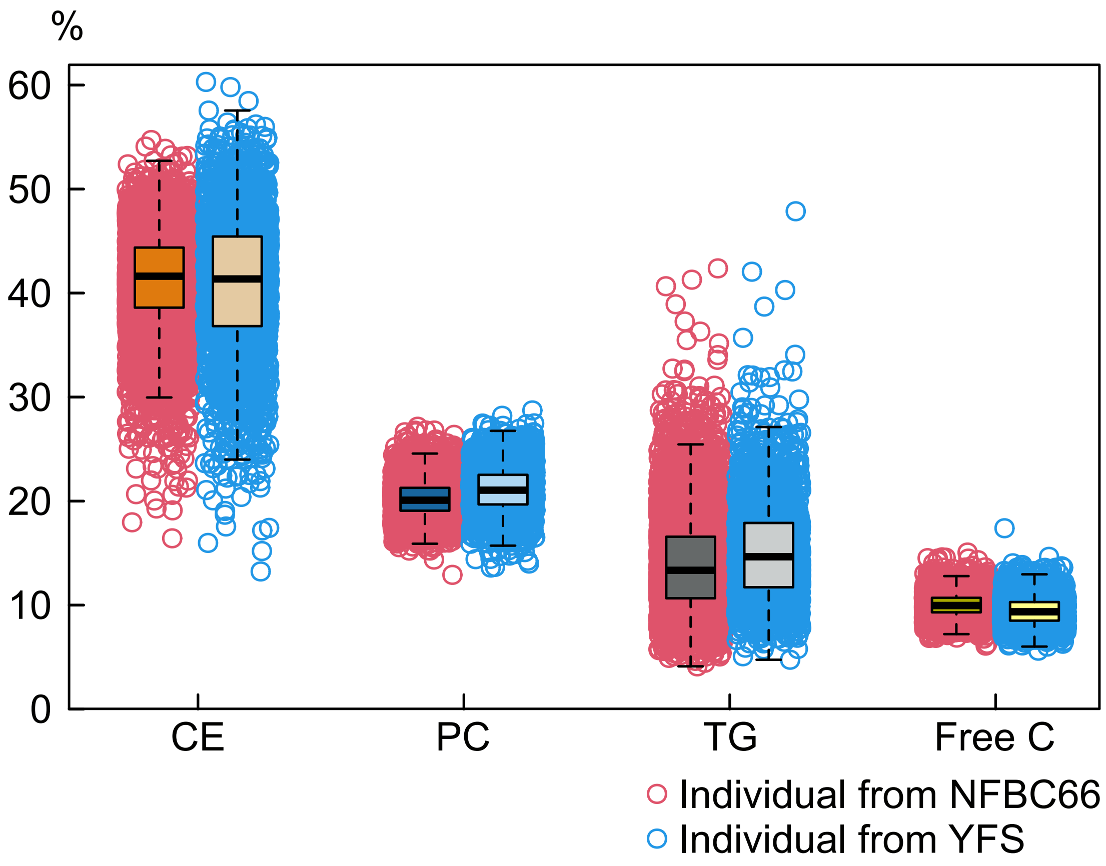

Figure S2.

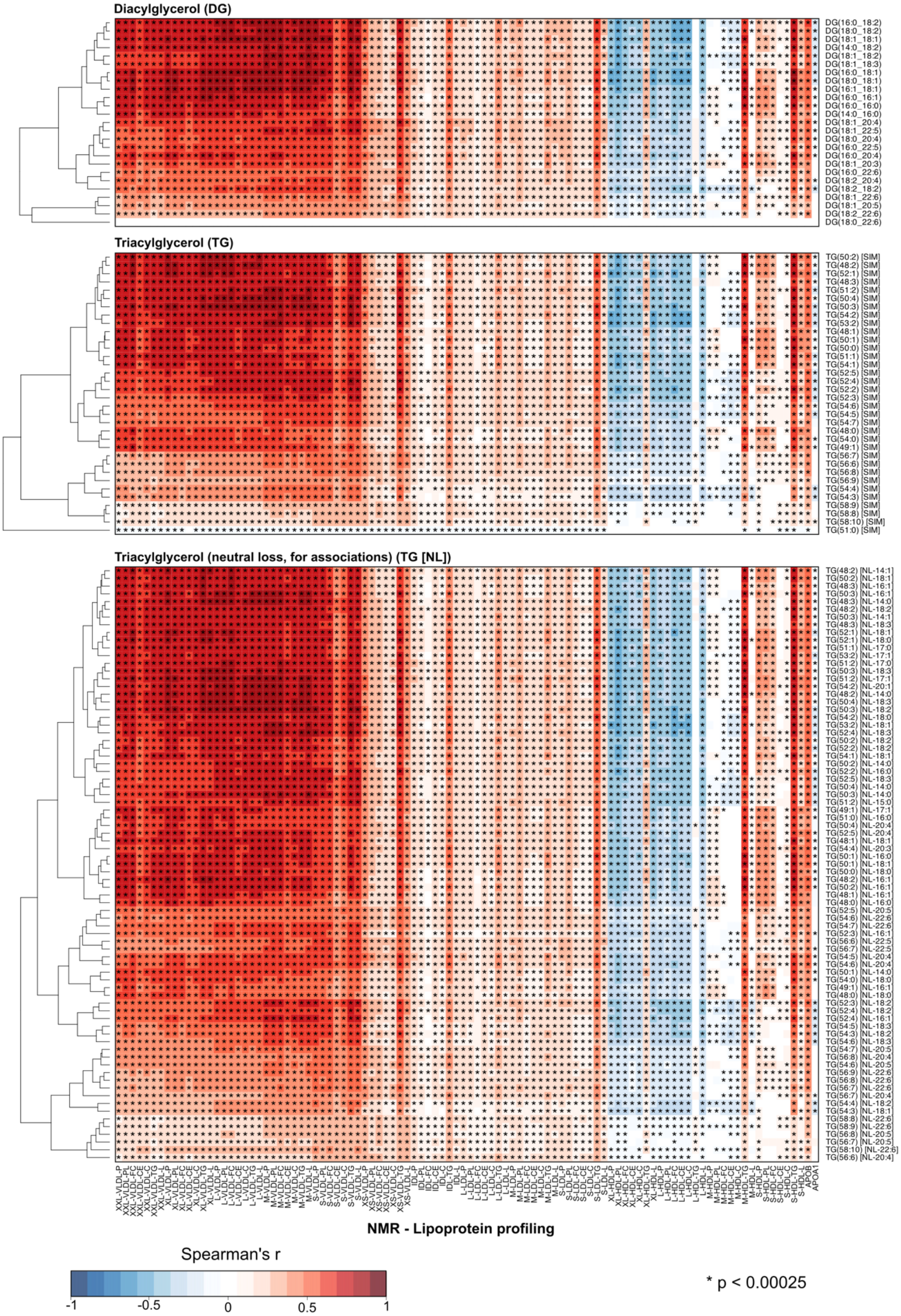
Diacylglycerol (DG)
Triacylglycerol (TG)
Triacylglycerol (neutral loss, for associations) (TG [NL])
NMR - Lipoprotein profiling
Spearman's r
-1
-0.5
0
0.5
1
* p < 0.00025

Figure S3.

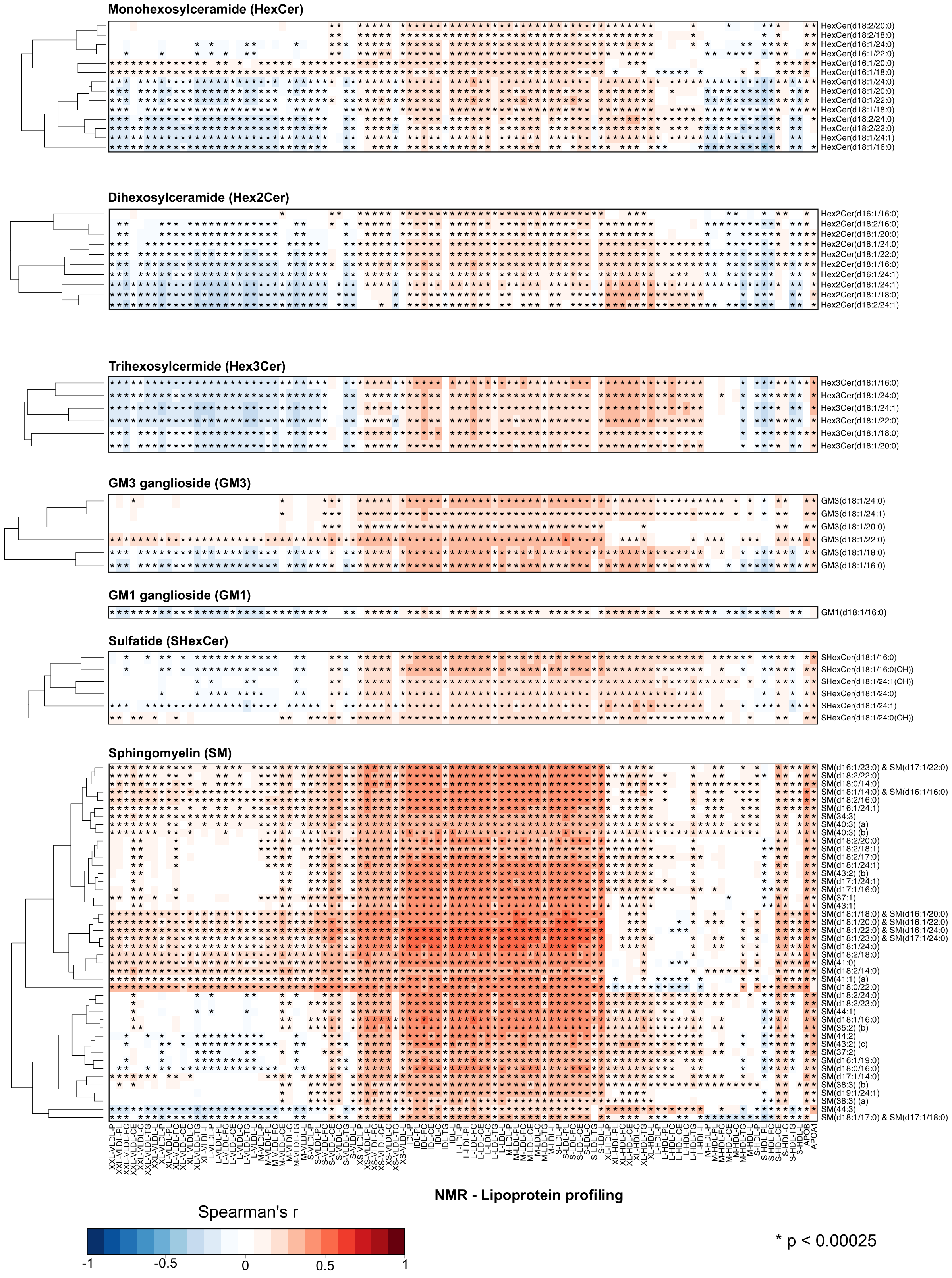

Monohexosylceramide (HexCer)
HexCer(d18:2/20:0)
HexCer(d18:2/18:0)
HexCer(d16:1/24:0)
HexCer(d16:1/22:0)
HexCer(d16:1/20:0)
HexCer(d16:1/18:0)
HexCer(d18:1/24:0)
HexCer(d18:1/20:0)
HexCer(d18:1/22:0)
HexCer(d18:1/18:0)
HexCer(d18:2/24:0)
HexCer(d18:2/22:0)
HexCer(d18:1/24:1)
HexCer(d18:1/16:0)
Dihexosylceramide (Hex2Cer)
Hex2Cer(d16:1/16:0)
Hex2Cer(d18:2/16:0)
Hex2Cer(d18:1/20:0)
Hex2Cer(d18:1/24:0)
Hex2Cer(d18:1/22:0)
Hex2Cer(d18:1/16:0)
Hex2Cer(d16:1/24:1)
Hex2Cer(d18:1/24:1)
Hex2Cer(d18:1/18:0)
Hex2Cer(d18:2/24:1)
Trihexosylcermide (Hex3Cer)
Hex3Cer(d18:1/16:0)
Hex3Cer(d18:1/24:0)
Hex3Cer(d18:1/24:1)
Hex3Cer(d18:1/22:0)
Hex3Cer(d18:1/18:0)
Hex3Cer(d18:1/20:0)
GM3 ganglioside (GM3)
GM3(d18:1/24:0)
GM3(d18:1/24:1)
GM3(d18:1/20:0)
GM3(d18:1/22:0)
GM3(d18:1/18:0)
GM3(d18:1/16:0)
GM1 ganglioside (GM1)
GM1(d18:1/16:0)
Sulfatide (SHexCer)
SHexCer(d18:1/16:0)
SHexCer(d18:1/16:0(OH))
SHexCer(d18:1/24:1(OH))
SHexCer(d18:1/24:0)
SHexCer(d18:1/24:1)
SHexCer(d18:1/24:0(OH))
Sphingomyelin (SM)
SM(d16:1/23:0) & SM(d17:1/22:0)
SM(d18:2/22:0)
SM(d18:0/14:0)
SM(d18:1/14:0) & SM(d16:1/16:0)
SM(d18:2/16:0)
SM(d16:1/24:1)
SM(34:3)
SM(40:3) (a)
SM(40:3) (b)
SM(d18:2/20:0)
SM(d18:2/18:1)
SM(d18:2/17:0)
SM(d18:1/24:1)
SM(43:2) (b)
SM(d17:1/24:1)
SM(d17:1/16:0)
SM(37:1)
SM(43:1)
SM(d18:1/18:0) & SM(d16:1/20:0)
SM(d18:1/20:0) & SM(d16:1/22:0)
SM(d18:1/22:0) & SM(d16:1/24:0)
SM(d18:1/23:0) & SM(d17:1/24:0)
SM(d18:1/24:0)
SM(d18:2/18:0)
SM(41:0)
SM(d18:2/14:0)
SM(41:1) (a)
SM(d18:0/22:0)
SM(d18:2/24:0)
SM(d18:2/23:0)
SM(44:1)
SM(d18:1/16:0)
SM(35:2) (b)
SM(44:2)
SM(43:2) (c)
SM(37:2)
SM(d16:1/19:0)
SM(d18:0/16:0)
SM(d17:1/14:0)
SM(38:3) (b)
SM(d19:1/24:1)
SM(38:3) (a)
SM(44:3)
SM(d18:1/17:0) & SM(d17:1/18:0)
NMR - Lipoprotein profiling
Spearman's r
-1
-0.5
0
0.5
1
* p < 0.00025

Figure S4.

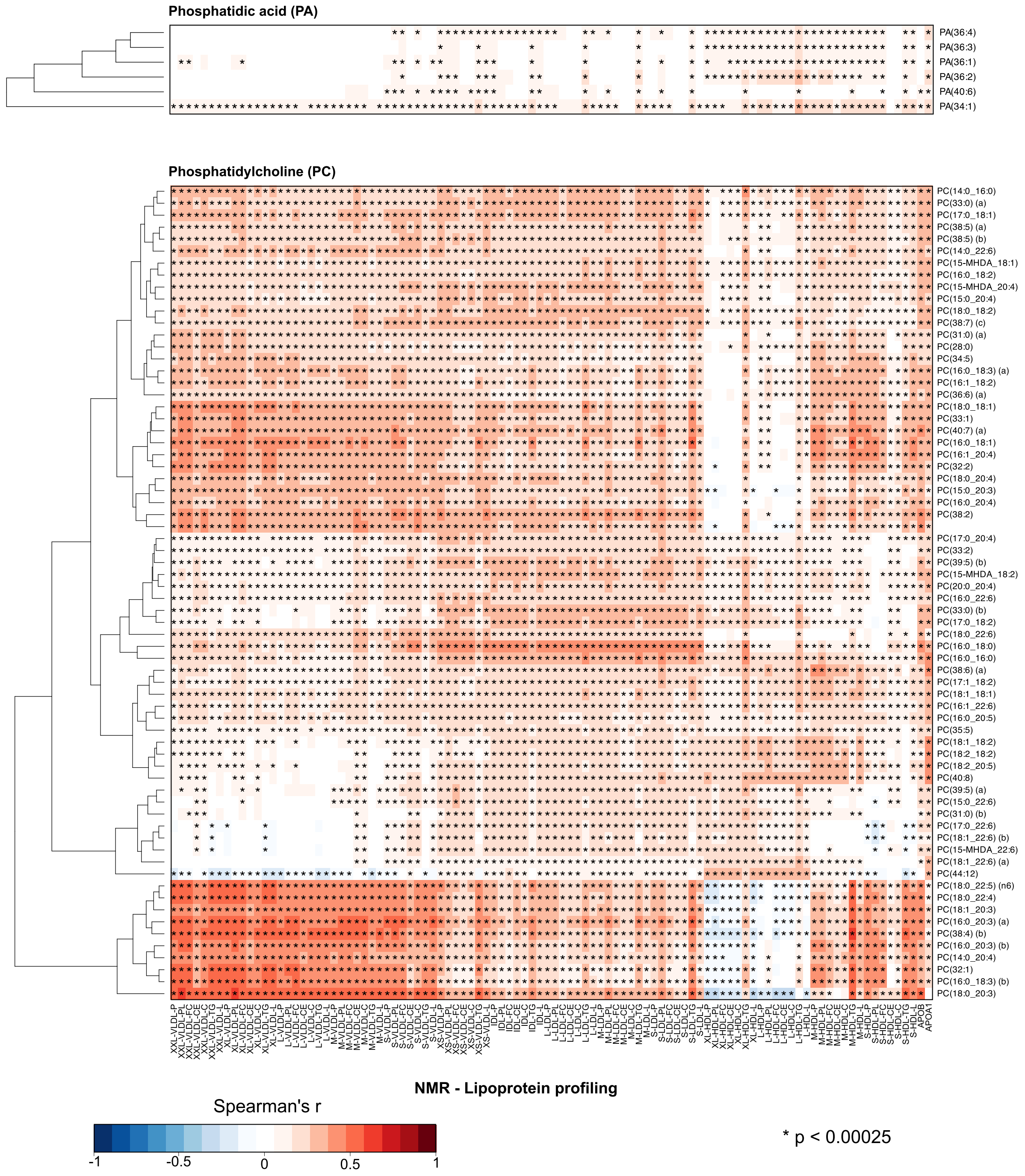

Phosphatidic acid (PA)
PA(36:4)
PA(36:3)
PA(36:1)
PA(36:2)
PA(40:6)
PA(34:1)
Phosphatidylcholine (PC)
PC(14:0_16:0)
PC(33:0) (a)
PC(17:0_18:1)
PC(38:5) (a)
PC(38:5) (b)
PC(14:0_22:6)
PC(15-MHDA_18:1)
PC(16:0_18:2)
PC(15-MHDA_20:4)
PC(15:0_20:4)
PC(18:0_18:2)
PC(38:7) (c)
PC(31:0) (a)
PC(28:0)
PC(34:5)
PC(16:0_18:3) (a)
PC(16:1_18:2)
PC(36:6) (a)
PC(18:0_18:1)
PC(33:1)
PC(40:7) (a)
PC(16:0_18:1)
PC(16:1_20:4)
PC(32:2)
PC(18:0_20:4)
PC(15:0_20:3)
PC(16:0_20:4)
PC(38:2)
PC(17:0_20:4)
PC(33:2)
PC(39:5) (b)
PC(15-MHDA_18:2)
PC(20:0_20:4)
PC(16:0_22:6)
PC(33:0) (b)
PC(17:0_18:2)
PC(18:0_22:6)
PC(16:0_18:0)
PC(16:0_16:0)
PC(38:6) (a)
PC(17:1_18:2)
PC(18:1_18:1)
PC(16:1_22:6)
PC(16:0_20:5)
PC(35:5)
PC(18:1_18:2)
PC(18:2_18:2)
PC(18:2_20:5)
PC(40:8)
PC(39:5) (a)
PC(15:0_22:6)
PC(31:0) (b)
PC(17:0_22:6)
PC(18:1_22:6) (b)
PC(15-MHDA_22:6)
PC(18:1_22:6) (a)
PC(44:12)
PC(18:0_22:5) (n6)
PC(18:0_22:4)
PC(18:1_20:3)
PC(16:0_20:3) (a)
PC(38:4) (b)
PC(16:0_20:3) (b)
PC(14:0_20:4)
PC(32:1)
PC(16:0_18:3) (b)
PC(18:0_20:3)
NMR - Lipoprotein profiling
Spearman's r
-1
-0.5
0
0.5
1
* p < 0.00025

Figure S5.

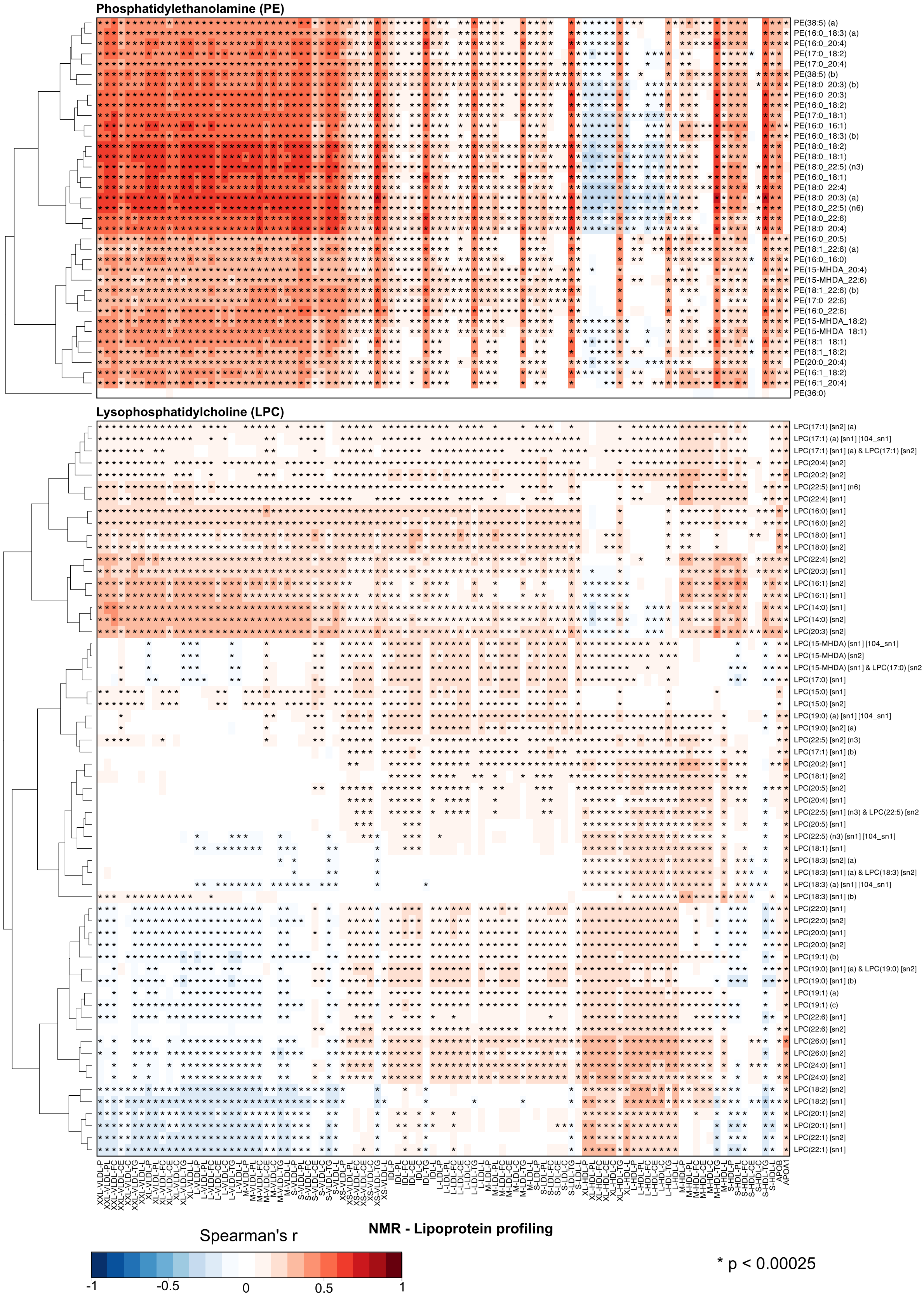

Phosphatidylethanolamine (PE)
Lysophosphatidylcholine (LPC)
NMR - Lipoprotein profiling
Spearman's r
-1
-0.5
0
0.5
1
* p < 0.00025

Figure S6.

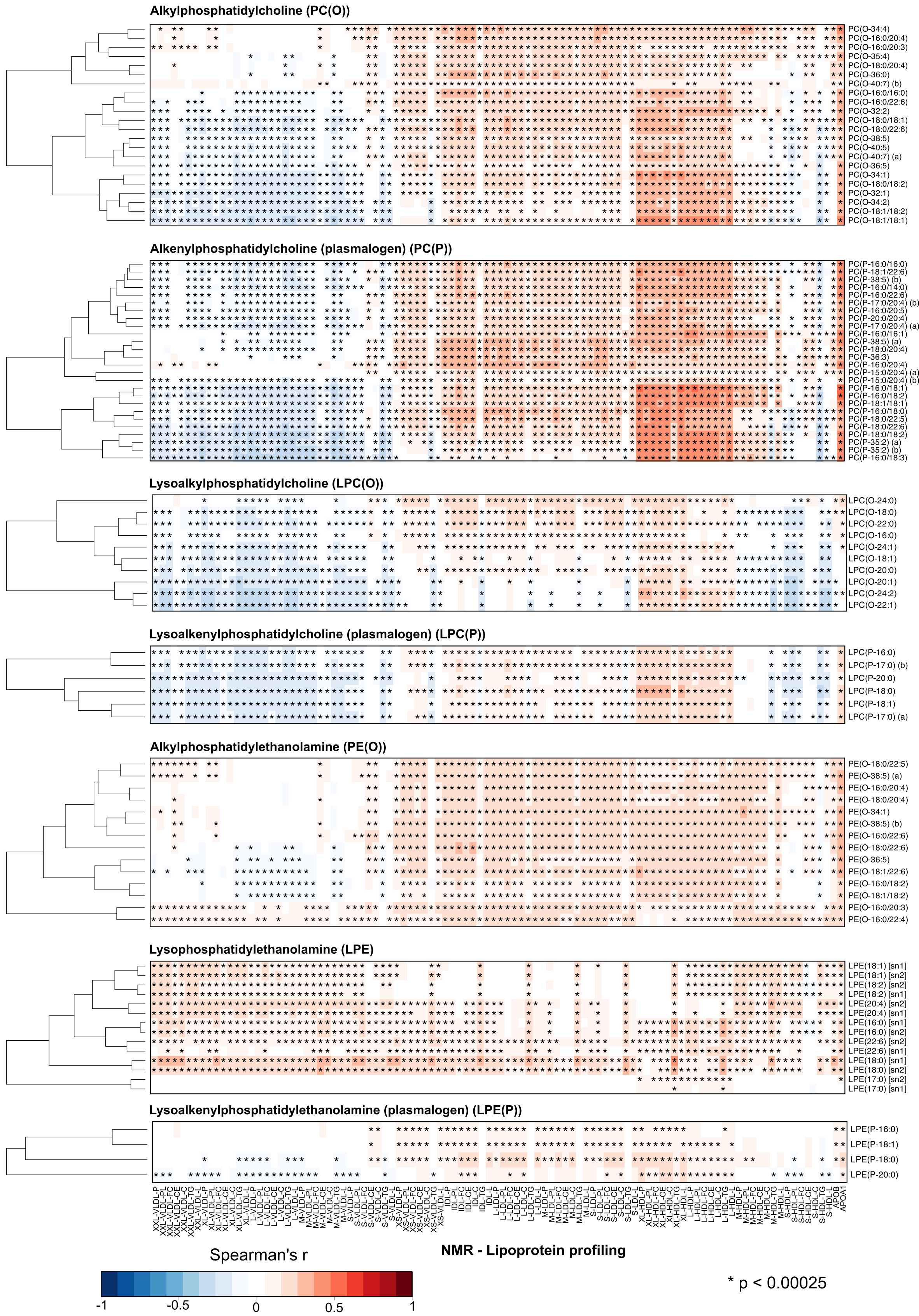

Alkylphosphatidylcholine (PC(O))
PC(O-34:4)
PC(O-16:0/20:4)
PC(O-16:0/20:3)
PC(O-35:4)
PC(O-18:0/20:4)
PC(O-36:0)
PC(O-40:7) (b)
PC(O-16:0/16:0)
PC(O-16:0/22:6)
PC(O-32:2)
PC(O-18:0/18:1)
PC(O-18:0/22:6)
PC(O-38:5)
PC(O-40:5)
PC(O-40:7) (a)
PC(O-36:5)
PC(O-34:1)
PC(O-18:0/18:2)
PC(O-32:1)
PC(O-34:2)
PC(O-18:1/18:2)
PC(O-18:1/18:1)
Alkenylphosphatidylcholine (plasmalogen) (PC(P))
PC(P-16:0/16:0)
PC(P-18:1/22:6)
PC(P-38:5) (b)
PC(P-16:0/14:0)
PC(P-16:0/22:6)
PC(P-17:0/20:4) (b)
PC(P-16:0/20:5)
PC(P-20:0/20:4)
PC(P-17:0/20:4) (a)
PC(P-16:0/16:1)
PC(P-38:5) (a)
PC(P-18:0/20:4)
PC(P-36:3)
PC(P-16:0/20:4)
PC(P-15:0/20:4) (a)
PC(P-15:0/20:4) (b)
PC(P-16:0/18:1)
PC(P-16:0/18:2)
PC(P-18:1/18:1)
PC(P-16:0/18:0)
PC(P-18:0/22:5)
PC(P-18:0/22:6)
PC(P-18:0/18:2)
PC(P-35:2) (a)
PC(P-35:2) (b)
PC(P-16:0/18:3)
Lysoalkylphosphatidylcholine (LPC(O))
LPC(O-24:0)
LPC(O-18:0)
LPC(O-22:0)
LPC(O-16:0)
LPC(O-24:1)
LPC(O-18:1)
LPC(O-20:0)
LPC(O-20:1)
LPC(O-24:2)
LPC(O-22:1)
Lysoalkenylphosphatidylcholine (plasmalogen) (LPC(P))
LPC(P-16:0)
LPC(P-17:0) (b)
LPC(P-20:0)
LPC(P-18:0)
LPC(P-18:1)
LPC(P-17:0) (a)
Alkylphosphatidylethanolamine (PE(O))
PE(O-18:0/22:5)
PE(O-38:5) (a)
PE(O-16:0/20:4)
PE(O-18:0/20:4)
PE(O-34:1)
PE(O-38:5) (b)
PE(O-16:0/22:6)
PE(O-18:0/22:6)
PE(O-36:5)
PE(O-18:1/22:6)
PE(O-16:0/18:2)
PE(O-18:1/18:2)
PE(O-16:0/20:3)
PE(O-16:0/22:4)
Lysophosphatidylethanolamine (LPE)
LPE(18:1) [sn1]
LPE(18:1) [sn2]
LPE(18:2) [sn2]
LPE(18:2) [sn1]
LPE(20:4) [sn2]
LPE(20:4) [sn1]
LPE(16:0) [sn1]
LPE(16:0) [sn2]
LPE(22:6) [sn2]
LPE(22:6) [sn1]
LPE(18:0) [sn1]
LPE(18:0) [sn2]
LPE(17:0) [sn2]
LPE(17:0) [sn1]
Lysoalkenylphosphatidylethanolamine (plasmalogen) (LPE(P))
LPE(P-16:0)
LPE(P-18:1)
LPE(P-18:0)
LPE(P-20:0)
XXL-VLDL-P
XXL-VLDL-PL
XXL-VLDL-FC
XXL-VLDL-CE
XXL-VLDL-C
XXL-VLDL-TG
XXL-VLDL-L
XL-VLDL-P
XL-VLDL-PL
XL-VLDL-FC
XL-VLDL-CE
XL-VLDL-C
XL-VLDL-TG
XL-VLDL-L
L-VLDL-P
L-VLDL-PL
L-VLDL-FC
L-VLDL-CE
L-VLDL-C
L-VLDL-TG
L-VLDL-L
M-VLDL-P
M-VLDL-PL
M-VLDL-FC
M-VLDL-CE
M-VLDL-C
M-VLDL-TG
M-VLDL-L
S-VLDL-P
S-VLDL-PL
S-VLDL-FC
S-VLDL-CE
S-VLDL-C
S-VLDL-TG
S-VLDL-L
XS-VLDL-P
XS-VLDL-PL
XS-VLDL-FC
XS-VLDL-CE
XS-VLDL-C
XS-VLDL-TG
XS-VLDL-L
IDL-P
IDL-PL
IDL-FC
IDL-CE
IDL-C
IDL-TG
IDL-L
L-LDL-P
L-LDL-PL
L-LDL-FC
L-LDL-CE
L-LDL-C
L-LDL-TG
L-LDL-L
M-LDL-P
M-LDL-PL
M-LDL-FC
M-LDL-CE
M-LDL-C
M-LDL-TG
M-LDL-L
S-LDL-P
S-LDL-PL
S-LDL-FC
S-LDL-CE
S-LDL-C
S-LDL-TG
S-LDL-L
XL-HDL-P
XL-HDL-PL
XL-HDL-FC
XL-HDL-CE
XL-HDL-C
XL-HDL-TG
XL-HDL-L
L-HDL-P
L-HDL-PL
L-HDL-FC
L-HDL-CE
L-HDL-C
L-HDL-TG
L-HDL-L
M-HDL-P
M-HDL-PL
M-HDL-FC
M-HDL-CE
M-HDL-C
M-HDL-TG
M-HDL-L
S-HDL-P
S-HDL-PL
S-HDL-FC
S-HDL-CE
S-HDL-C
S-HDL-TG
S-HDL-L
APOB
APOA1
NMR - Lipoprotein profiling
Spearman's r
-1
-0.5
0
0.5
1
* p < 0.00025

Figure S7.

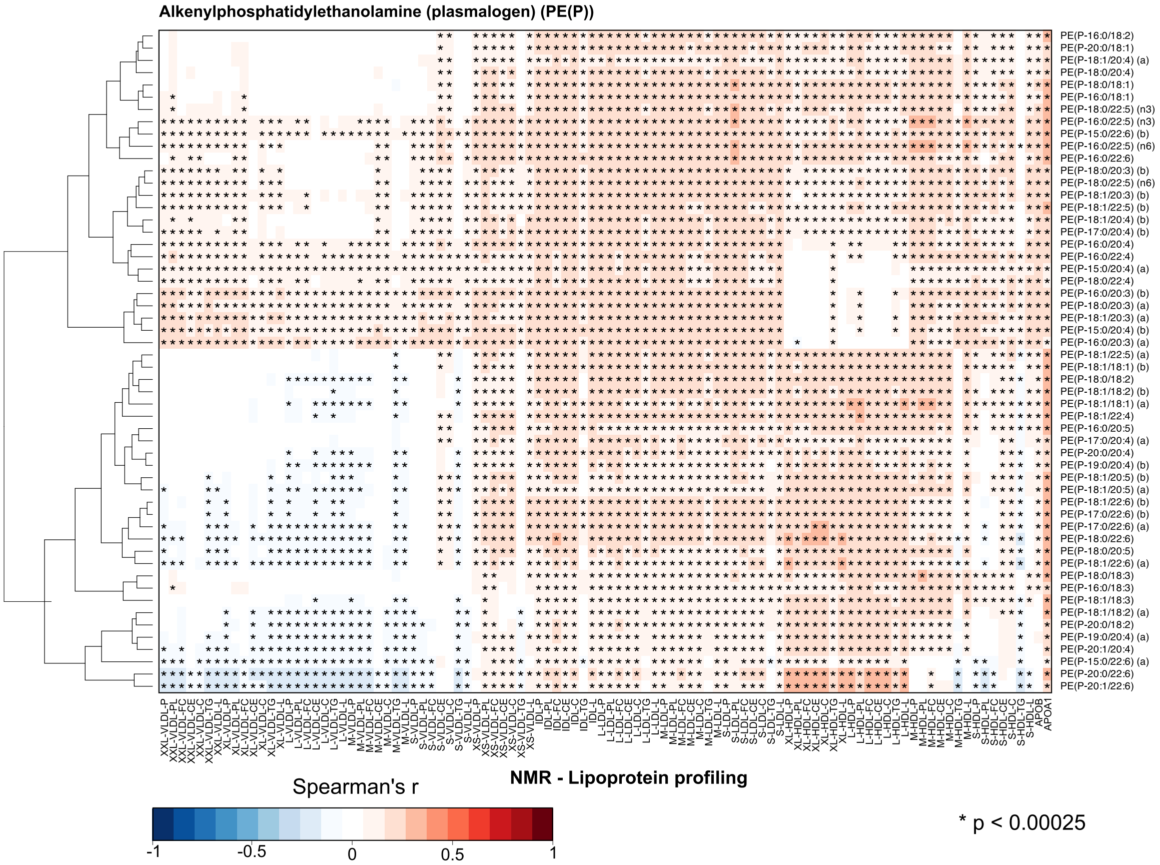

Alkenylphosphatidylethanolamine (plasmalogen) (PE(P))
PE(P-16:0/18:2)
PE(P-20:0/18:1)
PE(P-18:1/20:4) (a)
PE(P-18:0/20:4)
PE(P-18:0/18:1)
PE(P-16:0/18:1)
PE(P-18:0/22:5) (n3)
PE(P-16:0/22:5) (n3)
PE(P-15:0/22:6) (b)
PE(P-16:0/22:5) (n6)
PE(P-16:0/22:6)
PE(P-18:0/20:3) (b)
PE(P-18:0/22:5) (n6)
PE(P-18:1/20:3) (b)
PE(P-18:1/22:5) (b)
PE(P-18:1/20:4) (b)
PE(P-17:0/20:4) (b)
PE(P-16:0/20:4)
PE(P-16:0/22:4)
PE(P-15:0/20:4) (a)
PE(P-18:0/22:4)
PE(P-16:0/20:3) (b)
PE(P-18:0/20:3) (a)
PE(P-18:1/20:3) (a)
PE(P-15:0/20:4) (b)
PE(P-16:0/20:3) (a)
PE(P-18:1/22:5) (a)
PE(P-18:1/18:1) (b)
PE(P-18:0/18:2)
PE(P-18:1/18:2) (b)
PE(P-18:1/18:1) (a)
PE(P-18:1/22:4)
PE(P-16:0/20:5)
PE(P-17:0/20:4) (a)
PE(P-20:0/20:4)
PE(P-19:0/20:4) (b)
PE(P-18:1/20:5) (b)
PE(P-18:1/20:5) (a)
PE(P-18:1/22:6) (b)
PE(P-17:0/22:6) (b)
PE(P-17:0/22:6) (a)
PE(P-18:0/22:6)
PE(P-18:0/20:5)
PE(P-18:1/22:6) (a)
PE(P-18:0/18:3)
PE(P-16:0/18:3)
PE(P-18:1/18:3)
PE(P-18:1/18:2) (a)
PE(P-20:0/18:2)
PE(P-19:0/20:4) (a)
PE(P-20:1/20:4)
PE(P-15:0/22:6) (a)
PE(P-20:0/22:6)
PE(P-20:1/22:6)
XXL-VLDL-P
XXL-VLDL-PL
XXL-VLDL-FC
XXL-VLDL-CE
XXL-VLDL-C
XXL-VLDL-TG
XXL-VLDL-L
XL-VLDL-P
XL-VLDL-PL
XL-VLDL-FC
XL-VLDL-CE
XL-VLDL-C
XL-VLDL-TG
XL-VLDL-L
L-VLDL-P
L-VLDL-PL
L-VLDL-FC
L-VLDL-CE
L-VLDL-C
L-VLDL-TG
L-VLDL-L
M-VLDL-P
M-VLDL-PL
M-VLDL-FC
M-VLDL-CE
M-VLDL-C
M-VLDL-TG
M-VLDL-L
S-VLDL-P
S-VLDL-PL
S-VLDL-FC
S-VLDL-CE
S-VLDL-C
S-VLDL-TG
S-VLDL-L
XS-VLDL-P
XS-VLDL-PL
XS-VLDL-FC
XS-VLDL-CE
XS-VLDL-C
XS-VLDL-TG
XS-VLDL-L
IDL-P
IDL-PL
IDL-FC
IDL-CE
IDL-C
IDL-TG
IDL-L
L-LDL-P
L-LDL-PL
L-LDL-FC
L-LDL-CE
L-LDL-C
L-LDL-TG
L-LDL-L
M-LDL-P
M-LDL-PL
M-LDL-FC
M-LDL-CE
M-LDL-C
M-LDL-TG
M-LDL-L
S-LDL-P
S-LDL-PL
S-LDL-FC
S-LDL-CE
S-LDL-C
S-LDL-TG
S-LDL-L
XL-HDL-P
XL-HDL-PL
XL-HDL-FC
XL-HDL-CE
XL-HDL-C
XL-HDL-TG
XL-HDL-L
L-HDL-P
L-HDL-PL
L-HDL-FC
L-HDL-CE
L-HDL-C
L-HDL-TG
L-HDL-L
M-HDL-P
M-HDL-PL
M-HDL-FC
M-HDL-CE
M-HDL-C
M-HDL-TG
M-HDL-L
S-HDL-P
S-HDL-PL
S-HDL-FC
S-HDL-CE
S-HDL-C
S-HDL-TG
S-HDL-L
APOB
APOA1
NMR - Lipoprotein profiling
Spearman's r
-1
-0.5
0
0.5
1
* p < 0.00025

Figure S8.

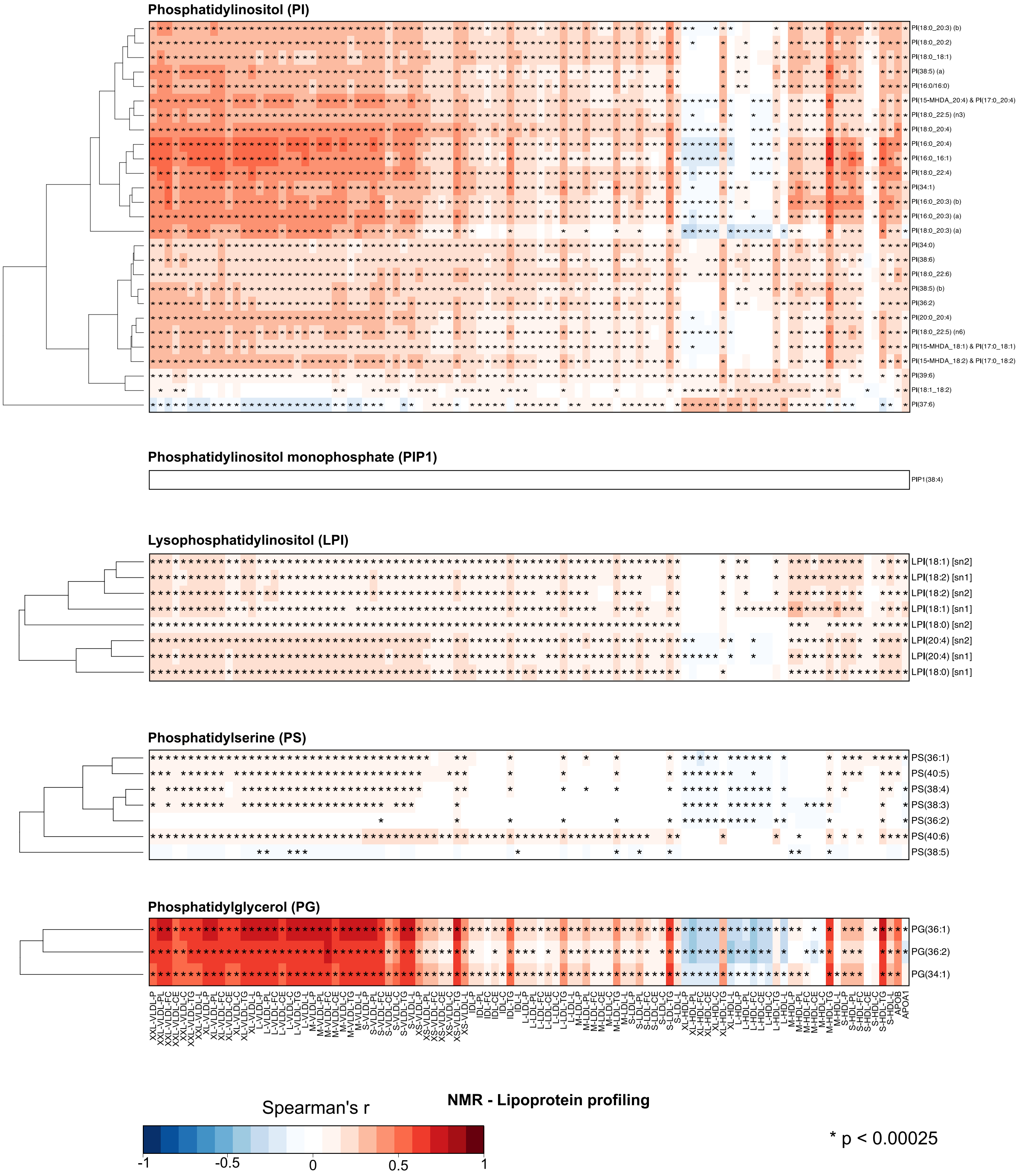
Phosphatidylinositol (PI)
Phosphatidylinositol monophosphate (PIP1)
PIP1(38:4)
Lysophosphatidylinositol (LPI)
LPI(18:1) [sn2]
LPI(18:2) [sn1]
LPI(18:2) [sn2]
LPI(18:1) [sn1]
LPI(18:0) [sn2]
LPI(20:4) [sn2]
LPI(20:4) [sn1]
LPI(18:0) [sn1]
Phosphatidylserine (PS)
PS(36:1)
PS(40:5)
PS(38:4)
PS(38:3)
PS(36:2)
PS(40:6)
PS(38:5)
Phosphatidylglycerol (PG)
PG(36:1)
PG(36:2)
PG(34:1)
Spearman's r
-1
-0.5
0
0.5
1
NMR - Lipoprotein profiling
* p < 0.00025

Figure S9.

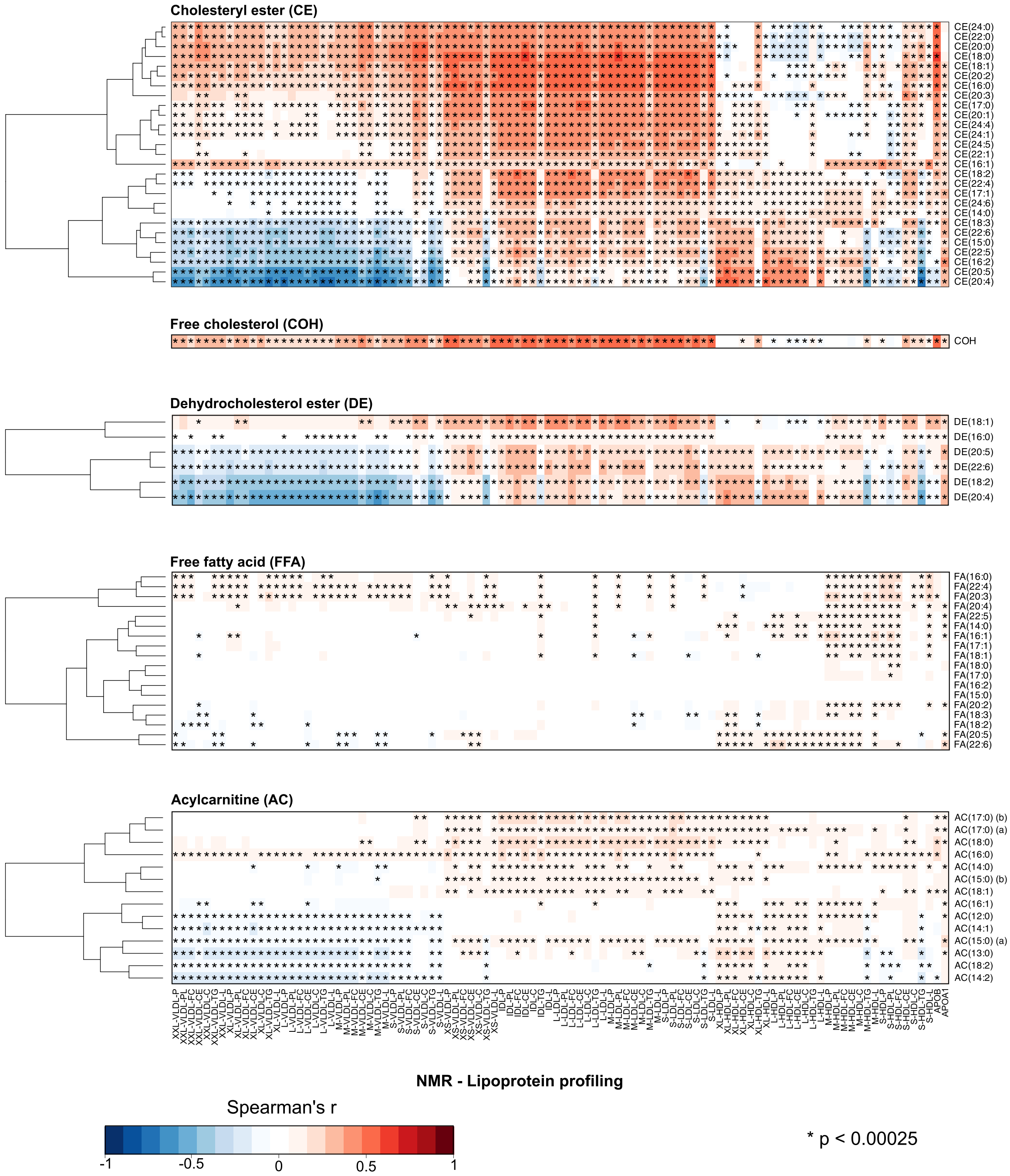
Cholesteryl ester (CE)
Free cholesterol (COH)
Dehydrocholesterol ester (DE)
Free fatty acid (FFA)
Acylcarnitine (AC)
NMR - Lipoprotein profiling
Spearman's r
-1
-0.5
0
0.5
1
* p < 0.00025

Figure S10.

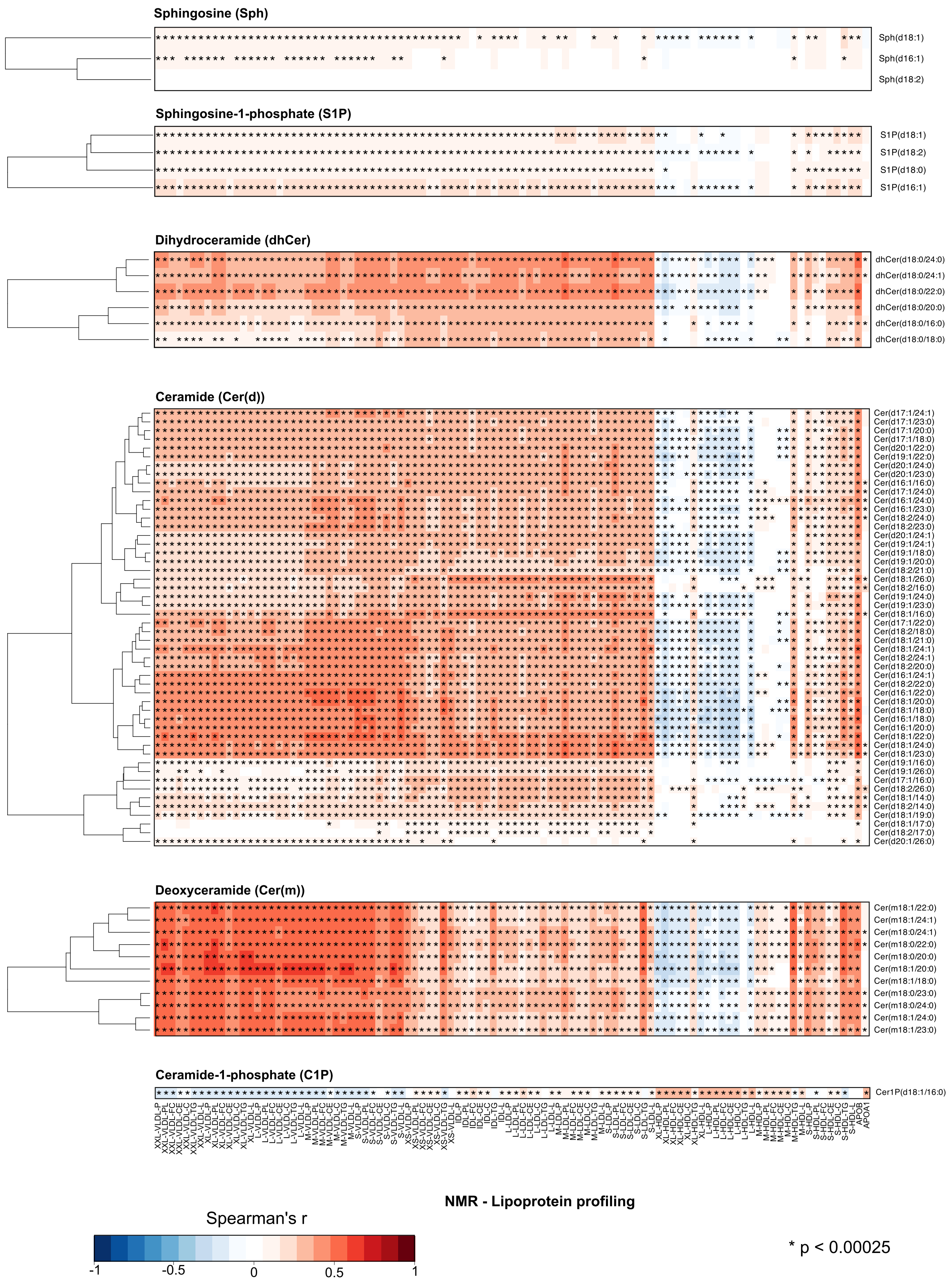
Sphingosine (Sph)
Sph(d18:1)
Sph(d16:1)
Sph(d18:2)
Sphingosine-1-phosphate (S1P)
S1P(d18:1)
S1P(d18:2)
S1P(d18:0)
S1P(d16:1)
Dihydroceramide (dhCer)
dhCer(d18:0/24:0)
dhCer(d18:0/24:1)
dhCer(d18:0/22:0)
dhCer(d18:0/20:0)
dhCer(d18:0/16:0)
dhCer(d18:0/18:0)
Ceramide (Cer(d))
Cer(d17:1/24:1)
Cer(d17:1/23:0)
Cer(d17:1/20:0)
Cer(d17:1/18:0)
Cer(d20:1/22:0)
Cer(d19:1/22:0)
Cer(d20:1/24:0)
Cer(d20:1/23:0)
Cer(d16:1/16:0)
Cer(d17:1/24:0)
Cer(d16:1/24:0)
Cer(d16:1/23:0)
Cer(d18:2/24:0)
Cer(d18:2/23:0)
Cer(d20:1/24:1)
Cer(d19:1/24:1)
Cer(d19:1/18:0)
Cer(d19:1/20:0)
Cer(d18:2/21:0)
Cer(d18:1/26:0)
Cer(d18:2/16:0)
Cer(d19:1/24:0)
Cer(d19:1/23:0)
Cer(d18:1/16:0)
Cer(d17:1/22:0)
Cer(d18:2/18:0)
Cer(d18:1/21:0)
Cer(d18:1/24:1)
Cer(d18:2/24:1)
Cer(d18:2/20:0)
Cer(d16:1/24:1)
Cer(d18:2/22:0)
Cer(d16:1/22:0)
Cer(d18:1/20:0)
Cer(d18:1/18:0)
Cer(d16:1/18:0)
Cer(d16:1/20:0)
Cer(d18:1/22:0)
Cer(d18:1/24:0)
Cer(d18:1/23:0)
Cer(d19:1/16:0)
Cer(d19:1/26:0)
Cer(d17:1/16:0)
Cer(d18:2/26:0)
Cer(d18:1/14:0)
Cer(d18:2/14:0)
Cer(d18:1/19:0)
Cer(d18:1/17:0)
Cer(d18:2/17:0)
Cer(d20:1/26:0)
Deoxyceramide (Cer(m))
Cer(m18:1/22:0)
Cer(m18:1/24:1)
Cer(m18:0/24:1)
Cer(m18:0/22:0)
Cer(m18:0/20:0)
Cer(m18:1/20:0)
Cer(m18:1/18:0)
Cer(m18:0/23:0)
Cer(m18:0/24:0)
Cer(m18:1/24:0)
Cer(m18:1/23:0)
Ceramide-1-phosphate (C1P)
Cer1P(d18:1/16:0)
NMR - Lipoprotein profiling
Spearman's r
-1
-0.5
0
0.5
1
* p < 0.00025

Figure S11.

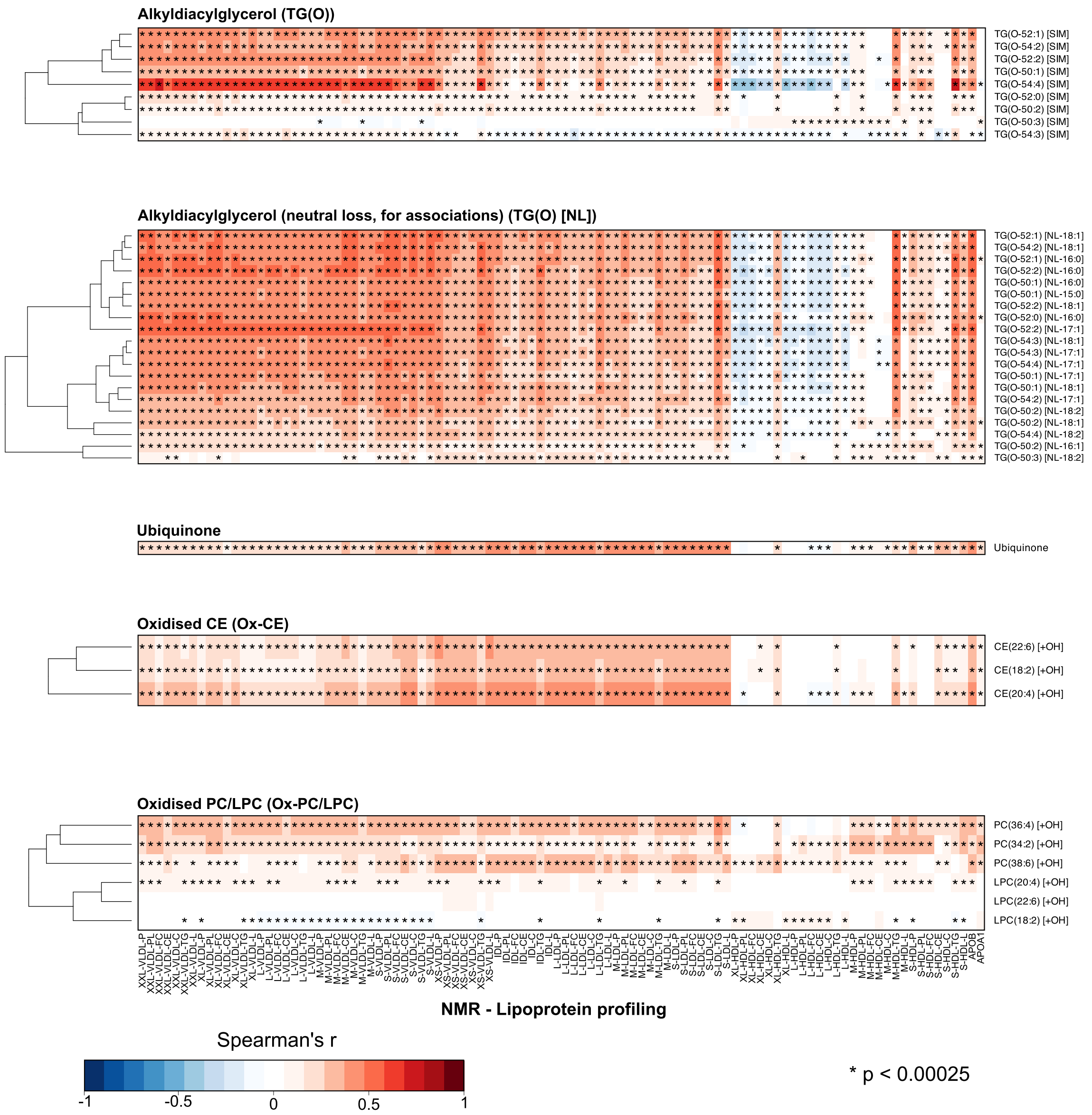

Alkyldiacylglycerol (TG(O))
TG(O-52:1) [SIM]
TG(O-54:2) [SIM]
TG(O-52:2) [SIM]
TG(O-50:1) [SIM]
TG(O-54:4) [SIM]
TG(O-52:0) [SIM]
TG(O-50:2) [SIM]
TG(O-50:3) [SIM]
TG(O-54:3) [SIM]
Alkyldiacylglycerol (neutral loss, for associations) (TG(O) [NL])
TG(O-52:1) [NL-18:1]
TG(O-54:2) [NL-18:1]
TG(O-52:1) [NL-16:0]
TG(O-52:2) [NL-16:0]
TG(O-50:1) [NL-16:0]
TG(O-50:1) [NL-15:0]
TG(O-52:2) [NL-18:1]
TG(O-52:0) [NL-16:0]
TG(O-52:2) [NL-17:1]
TG(O-54:3) [NL-18:1]
TG(O-54:3) [NL-17:1]
TG(O-54:4) [NL-17:1]
TG(O-50:1) [NL-17:1]
TG(O-50:1) [NL-18:1]
TG(O-54:2) [NL-17:1]
TG(O-50:2) [NL-18:2]
TG(O-50:2) [NL-18:1]
TG(O-54:4) [NL-18:2]
TG(O-50:2) [NL-16:1]
TG(O-50:3) [NL-18:2]
Ubiquinone
Ubiquinone
Oxidised CE (Ox-CE)
CE(22:6) [+OH]
CE(18:2) [+OH]
CE(20:4) [+OH]
Oxidised PC/LPC (Ox-PC/LPC)
PC(36:4) [+OH]
PC(34:2) [+OH]
PC(38:6) [+OH]
LPC(20:4) [+OH]
LPC(22:6) [+OH]
LPC(18:2) [+OH]
NMR - Lipoprotein profiling
Spearman's r
-1
-0.5
0
0.5
1
* p < 0.00025

Figure S12.

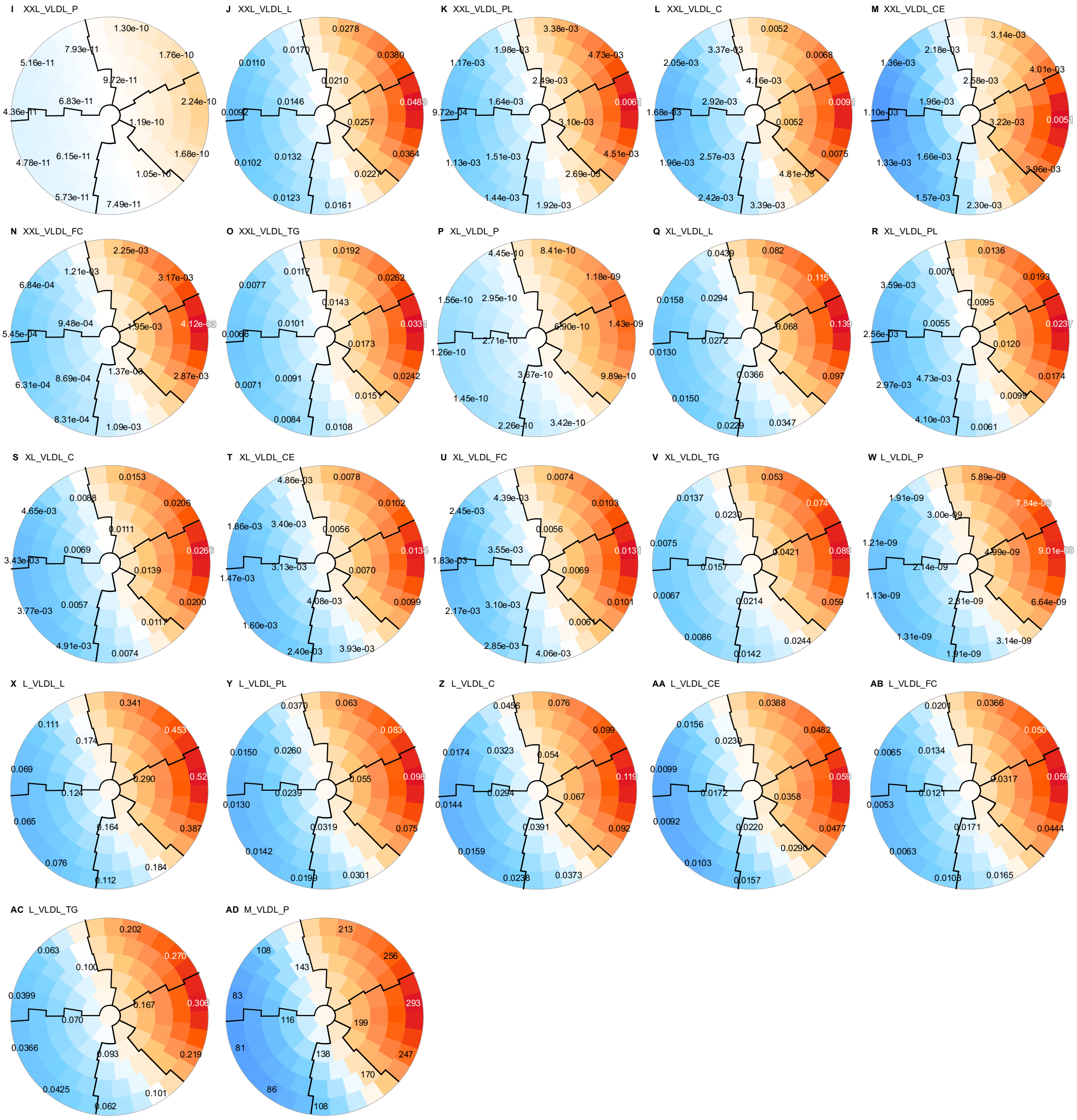
I XXL_VLDL_P
1.30e-10 7.93e-11 1.76e-10 5.16e-11 9.72e-11 2.24e-10 6.83e-11 4.36e-11 1.19e-10 1.68e-10 4.78e-11 6.15e-11 1.05e-10 5.73e-11 7.49e-11
J XXL_VLDL_L
0.0278 0.0170 0.0380 0.0110 0.0210 0.0146 0.0092 0.0257 0.0364 0.0102 0.0132 0.0227 0.0123 0.0161
K XXL_VLDL_PL
3.38e-03 1.98e-03 4.73e-03 1.17e-03 2.49e-03 1.64e-03 9.72e-04 3.10e-03 4.51e-03 1.13e-03 1.51e-03 2.69e-03 1.44e-03 1.92e-03
L XXL_VLDL_C
0.0052 3.37e-03 0.0068 2.05e-03 4.16e-03 2.92e-03 1.68e-03 0.0052 0.0075 1.96e-03 2.57e-03 4.81e-03 2.42e-03 3.39e-03
M XXL_VLDL_CE
3.14e-03 2.18e-03 4.01e-03 1.36e-03 2.58e-03 1.96e-03 1.10e-03 3.22e-03 1.33e-03 1.66e-03 3.96e-03 1.57e-03 2.30e-03
N XXL_VLDL_FC
2.25e-03 1.21e-03 3.17e-03 6.84e-04 9.48e-04 1.95e-03 5.45e-04 1.37e-03 2.87e-03 6.31e-04 8.69e-04 8.31e-04 1.09e-03
O XXL_VLDL_TG
0.0192 0.0117 0.0262 0.0077 0.0143 0.0101 0.0066 0.0173 0.0242 0.0071 0.0091 0.0151 0.0084 0.0108
P XL_VLDL_P
4.45e-10 8.41e-10 1.18e-09 1.56e-10 2.95e-10 6.90e-10 1.43e-09 2.71e-10 1.26e-10 3.67e-10 9.89e-10 1.45e-10 2.26e-10 3.42e-10
Q XL_VLDL_L
0.0439 0.082 0.115 0.0158 0.0294 0.068 0.139 0.0272 0.0130 0.0366 0.097 0.0150 0.0229 0.0347
R XL_VLDL_PL
0.0136 0.0071 0.0193 3.59e-03 0.0095 0.0055 2.56e-03 0.0120 2.97e-03 4.73e-03 0.0174 0.0099 4.10e-03 0.0061
S XL_VLDL_C
0.0153 0.0088 0.0206 4.65e-03 0.0111 0.0069 3.43e-03 0.0139 0.0200 3.77e-03 0.0057 0.0117 4.91e-03 0.0074
T XL_VLDL_CE
4.86e-03 0.0078 0.0102 1.86e-03 3.40e-03 0.0056 1.47e-03 3.13e-03 0.0070 4.08e-03 0.0099 1.60e-03 2.40e-03 3.93e-03
U XL_VLDL_FC
0.0074 4.39e-03 0.0103 2.45e-03 0.0056 3.55e-03 1.83e-03 0.0069 2.17e-03 3.10e-03 0.0101 0.0061 2.85e-03 4.06e-03
V XL_VLDL_TG
0.053 0.0137 0.074 0.0230 0.0075 0.0421 0.089 0.0157 0.0067 0.0214 0.059 0.0086 0.0244 0.0142
W L_VLDL_P
5.89e-09 1.91e-09 7.84e-09 3.00e-09 1.21e-09 4.99e-09 2.14e-09 1.13e-09 2.81e-09 6.64e-09 1.31e-09 3.14e-09 1.91e-09
X L_VLDL_L
0.341 0.111 0.453 0.174 0.069 0.290 0.124 0.065 0.164 0.387 0.076 0.184 0.112
Y L_VLDL_PL
0.0370 0.063 0.083 0.0150 0.0260 0.0239 0.055 0.0130 0.0319 0.075 0.0142 0.0199 0.0301
Z L_VLDL_C
0.0456 0.076 0.099 0.0174 0.0323 0.054 0.0294 0.067 0.0144 0.0391 0.092 0.0159 0.0238 0.0373
AA L_VLDL_CE
0.0388 0.0156 0.0482 0.0230 0.0099 0.0172 0.0358 0.0092 0.0220 0.0477 0.0296 0.0103 0.0157
AB L_VLDL_FC
0.0201 0.0366 0.050 0.0065 0.0134 0.0317 0.0121 0.0053 0.0171 0.0444 0.0063 0.0108 0.0165
AC L_VLDL_TG
0.202 0.063 0.270 0.100 0.0399 0.167 0.308 0.070 0.0366 0.093 0.219 0.0425 0.101 0.062
AD M_VLDL_P
213 108 143 256 83 116 293 199 81 138 247 170 86 108

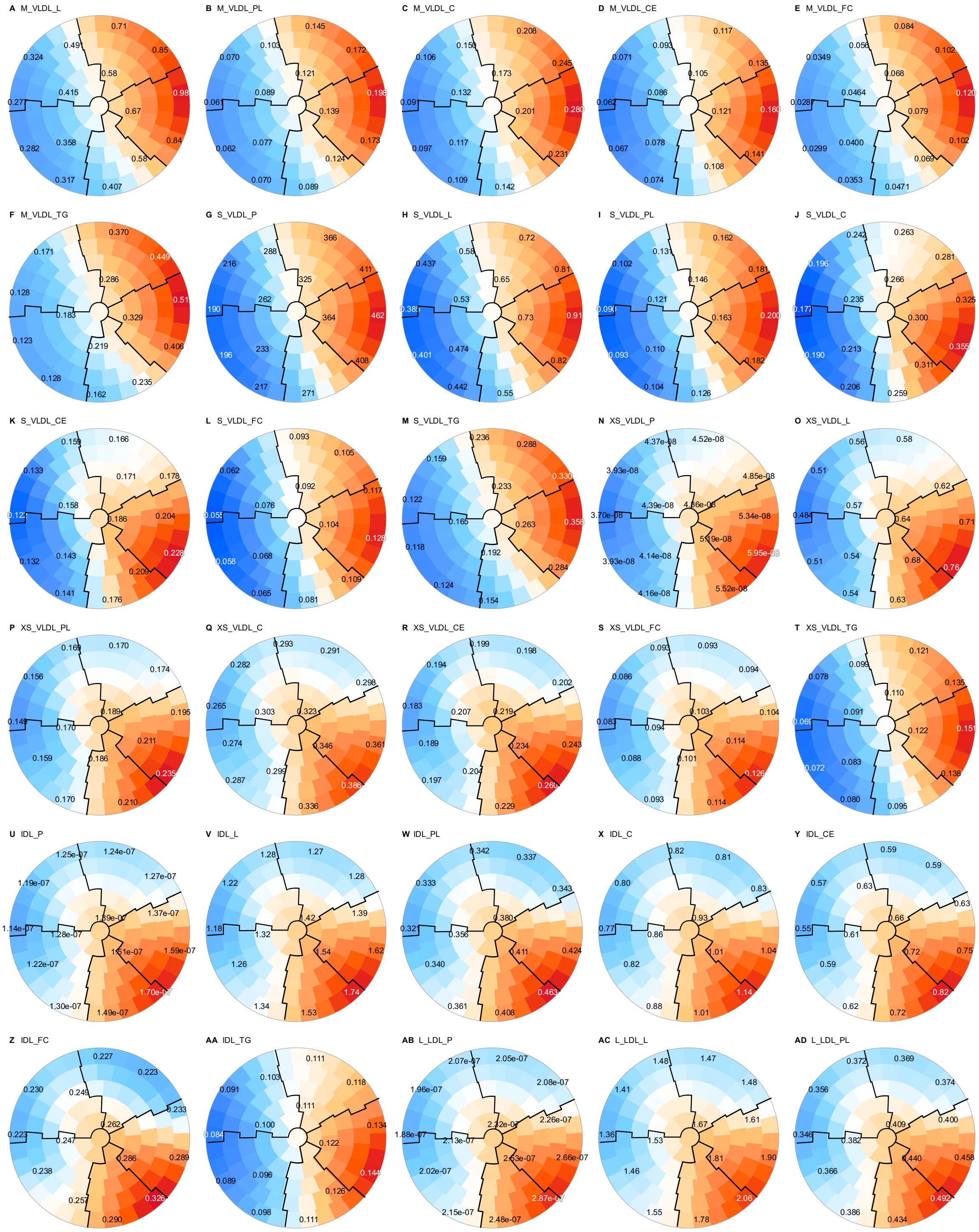
A M_VLDL_L
B M_VLDL_PL
C M_VLDL_C
D M_VLDL_CE
E M_VLDL_FC
F M_VLDL_TG
G S_VLDL_P
H S_VLDL_L
I S_VLDL_PL
J S_VLDL_C
K S_VLDL_CE
L S_VLDL_FC
M S_VLDL_TG
N XS_VLDL_P
O XS_VLDL_L
P XS_VLDL_PL
Q XS_VLDL_C
R XS_VLDL_CE
S XS_VLDL_FC
T XS_VLDL_TG
U IDL_P
V IDL_L
W IDL_PL
X IDL_C
Y IDL_CE
Z IDL_FC
AA IDL_TG
AB L_LDL_P
AC L_LDL_L
AD L_LDL_PL

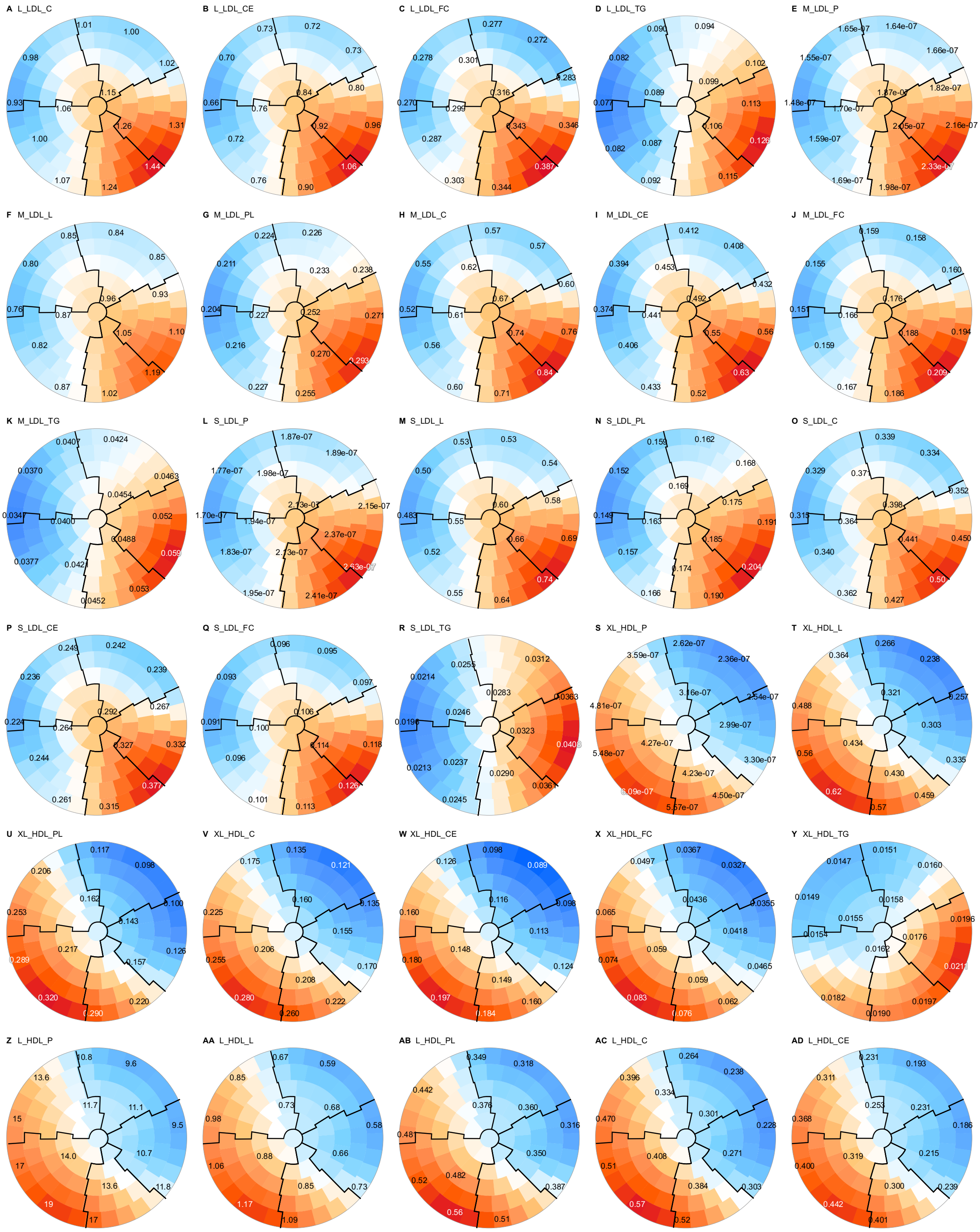
A L_LDL_C
B L_LDL_CE
C L_LDL_FC
D L_LDL_TG
E M_LDL_P
F M_LDL_L
G M_LDL_PL
H M_LDL_C
I M_LDL_CE
J M_LDL_FC
K M_LDL_TG
L S_LDL_P
M S_LDL_L
N S_LDL_PL
O S_LDL_C
P S_LDL_CE
Q S_LDL_FC
R S_LDL_TG
S XL_HDL_P
T XL_HDL_L
U XL_HDL_PL
V XL_HDL_C
W XL_HDL_CE
X XL_HDL_FC
Y XL_HDL_TG
Z L_HDL_P
AA L_HDL_L
AB L_HDL_PL
AC L_HDL_C
AD L_HDL_CE

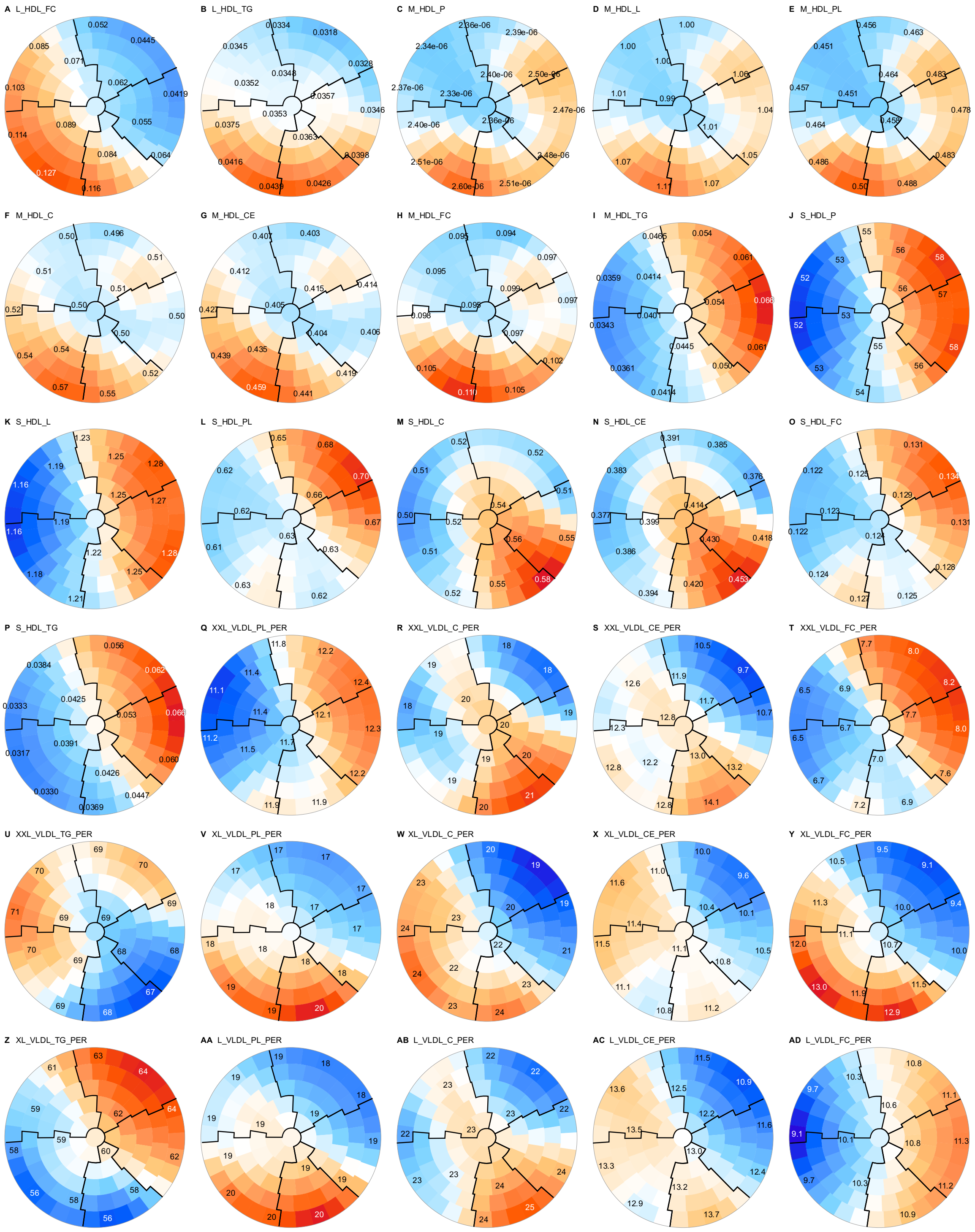

A L_HDL_FC
B L_HDL_TG
C M_HDL_P
D M_HDL_L
E M_HDL_PL
F M_HDL_C
G M_HDL_CE
H M_HDL_FC
I M_HDL_TG
J S_HDL_P
K S_HDL_L
L S_HDL_PL
M S_HDL_C
N S_HDL_CE
O S_HDL_FC
P S_HDL_TG
Q XXL_VLDL_PL_PER
R XXL_VLDL_C_PER
S XXL_VLDL_CE_PER
T XXL_VLDL_FC_PER
U XXL_VLDL_TG_PER
V XL_VLDL_PL_PER
W XL_VLDL_C_PER
X XL_VLDL_CE_PER
Y XL_VLDL_FC_PER
Z XL_VLDL_TG_PER
AA L_VLDL_PL_PER
AB L_VLDL_C_PER
AC L_VLDL_CE_PER
AD L_VLDL_FC_PER

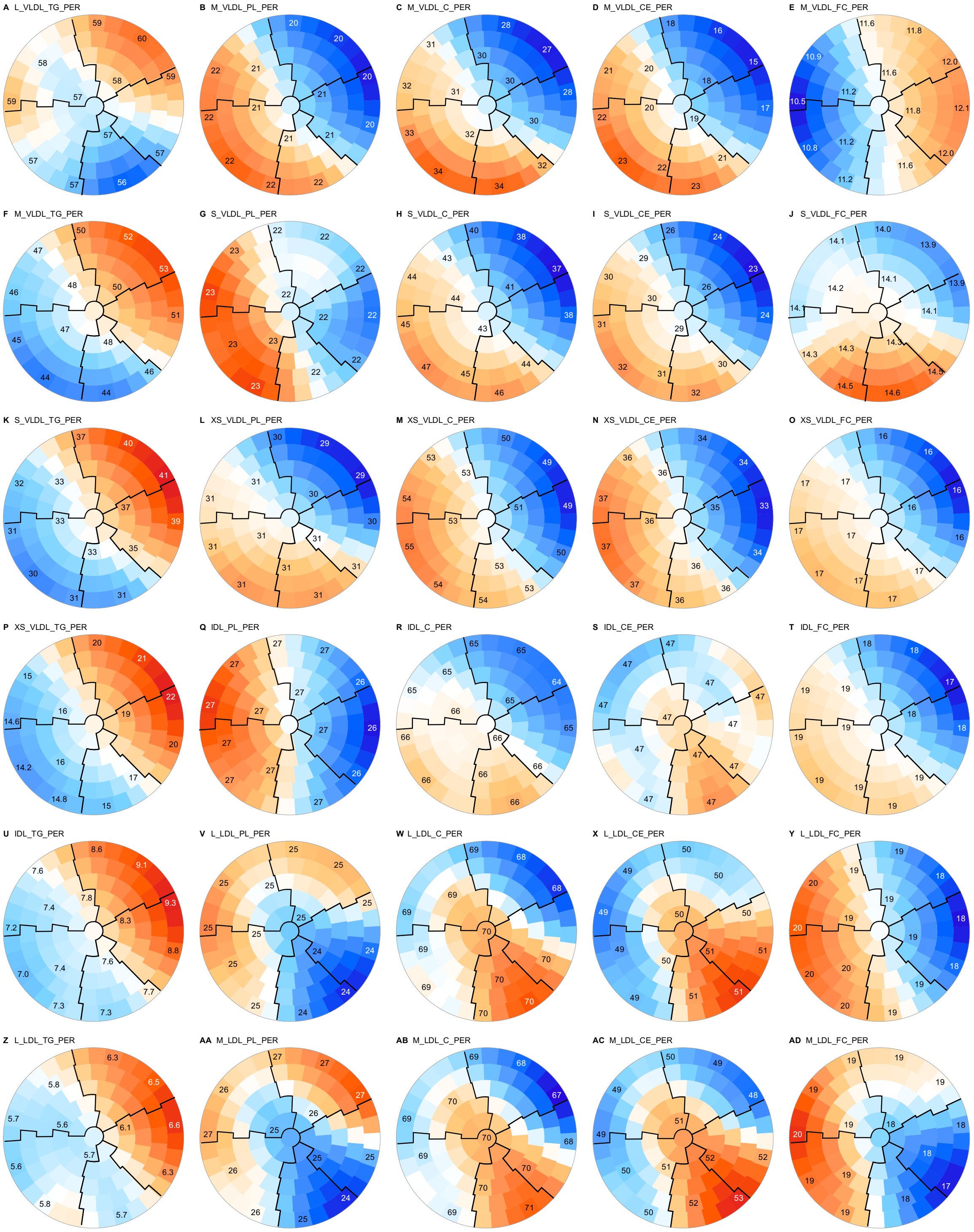
A L_VLDL_TG_PER
B M_VLDL_PL_PER
C M_VLDL_C_PER
D M_VLDL_CE_PER
E M_VLDL_FC_PER
F M_VLDL_TG_PER
G S_VLDL_PL_PER
H S_VLDL_C_PER
I S_VLDL_CE_PER
J S_VLDL_FC_PER
K S_VLDL_TG_PER
L XS_VLDL_PL_PER
M XS_VLDL_C_PER
N XS_VLDL_CE_PER
O XS_VLDL_FC_PER
P XS_VLDL_TG_PER
Q IDL_PL_PER
R IDL_C_PER
S IDL_CE_PER
T IDL_FC_PER
U IDL_TG_PER
V L_LDL_PL_PER
W L_LDL_C_PER
X L_LDL_CE_PER
Y L_LDL_FC_PER
Z L_LDL_TG_PER
AA M_LDL_PL_PER
AB M_LDL_C_PER
AC M_LDL_CE_PER
AD M_LDL_FC_PER

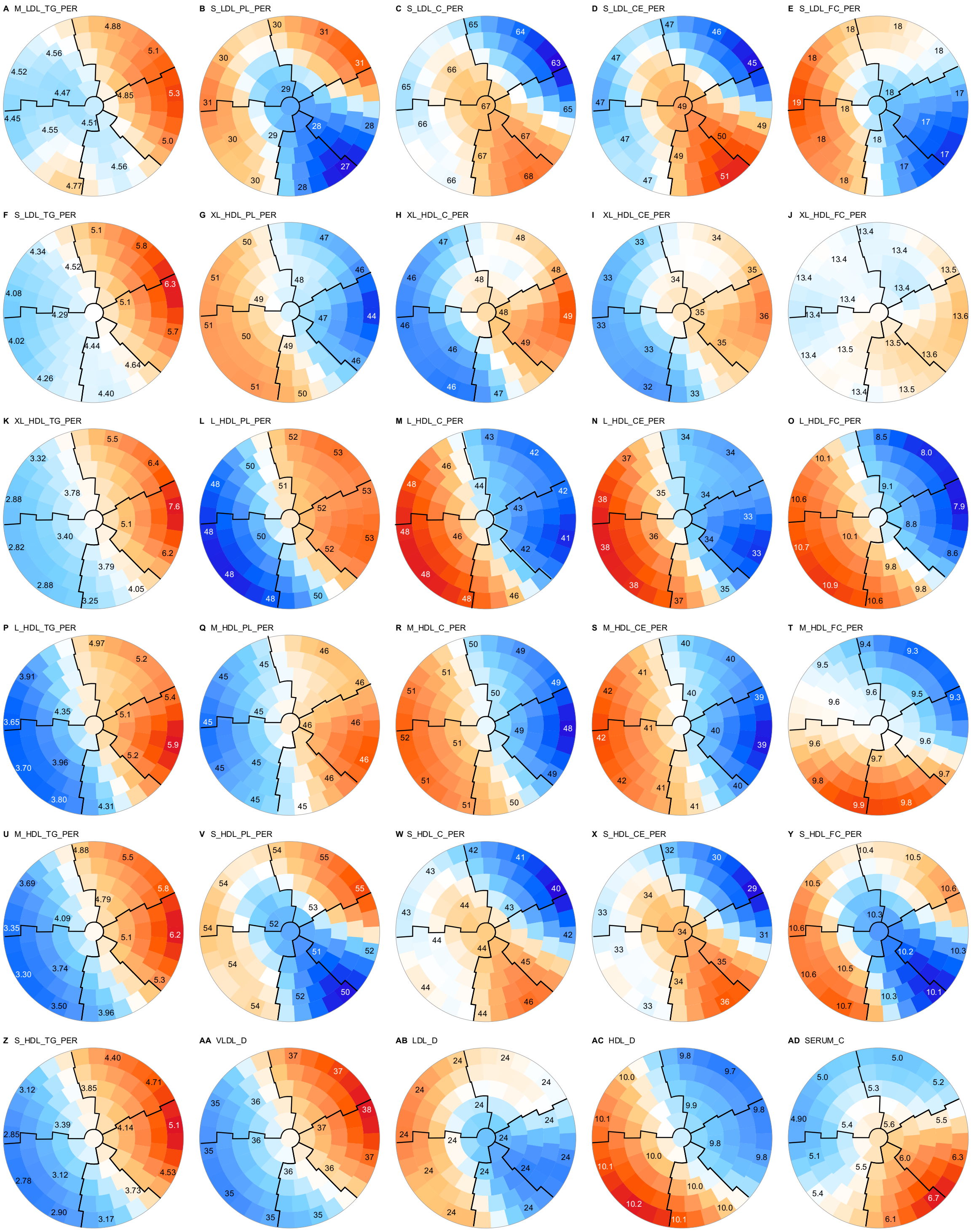

A M_LDL_TG_PER
B S_LDL_PL_PER
C S_LDL_C_PER
D S_LDL_CE_PER
E S_LDL_FC_PER
F S_LDL_TG_PER
G XL_HDL_PL_PER
H XL_HDL_C_PER
I XL_HDL_CE_PER
J XL_HDL_FC_PER
K XL_HDL_TG_PER
L L_HDL_PL_PER
M L_HDL_C_PER
N L_HDL_CE_PER
O L_HDL_FC_PER
P L_HDL_TG_PER
Q M_HDL_PL_PER
R M_HDL_C_PER
S M_HDL_CE_PER
T M_HDL_FC_PER
U M_HDL_TG_PER
V S_HDL_PL_PER
W S_HDL_C_PER
X S_HDL_CE_PER
Y S_HDL_FC_PER
Z S_HDL_TG_PER
AA VLDL_D
AB LDL_D
AC HDL_D
AD SERUM_C

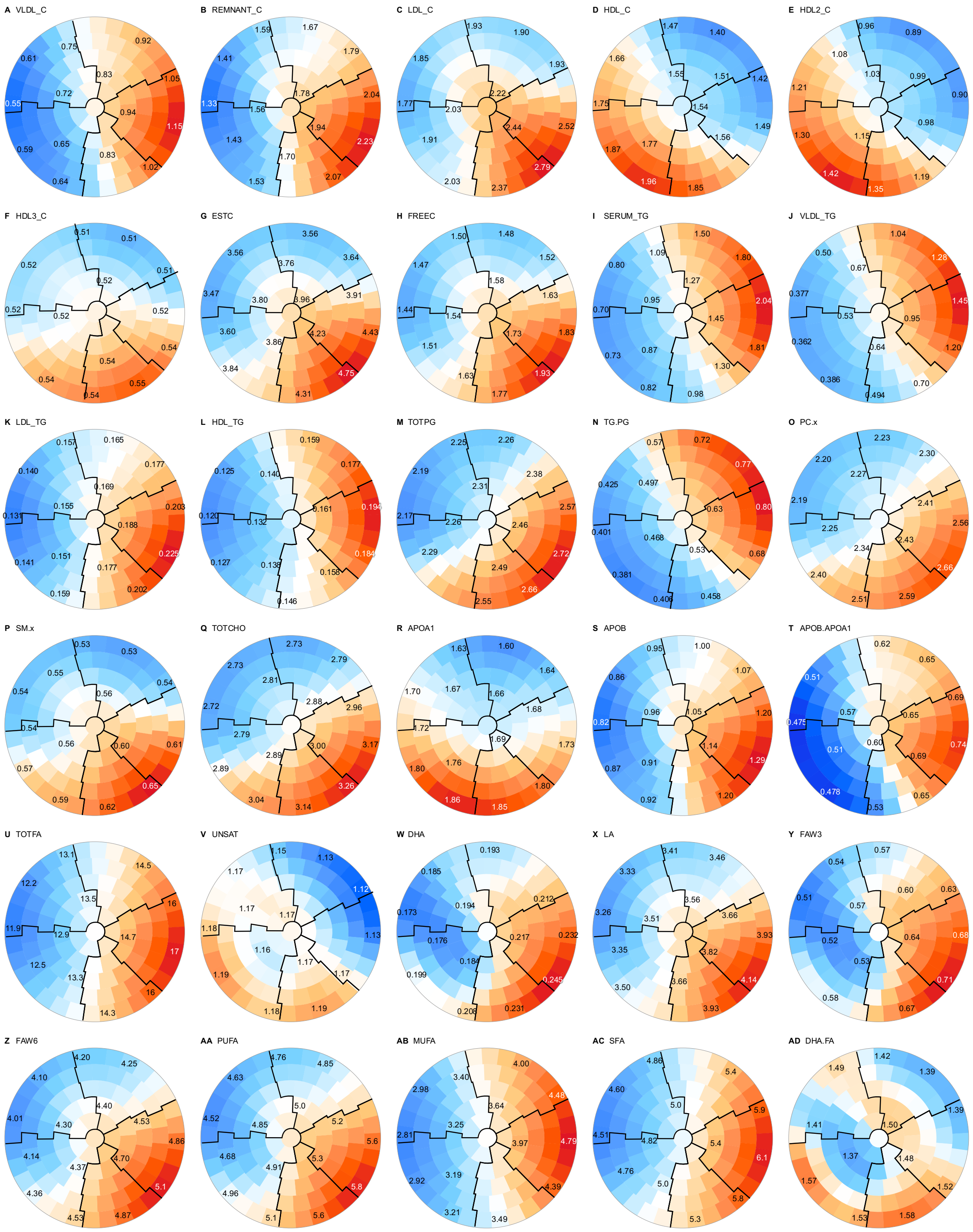
A VLDL_C
B REMNANT_C
C LDL_C
D HDL_C
E HDL2_C
F HDL3_C
G ESTC
H FREEC
I SERUM_TG
J VLDL_TG
K LDL_TG
L HDL_TG
M TOTPG
N TG.PG
O PC.x
P SM.x
Q TOTCHO
R APOA1
S APOB
T APOB.APOA1
U TOTFA
V UNSAT
W DHA
X LA
Y FAW3
Z FAW6
AA PUFA
AB MUFA
AC SFA
AD DHA.FA

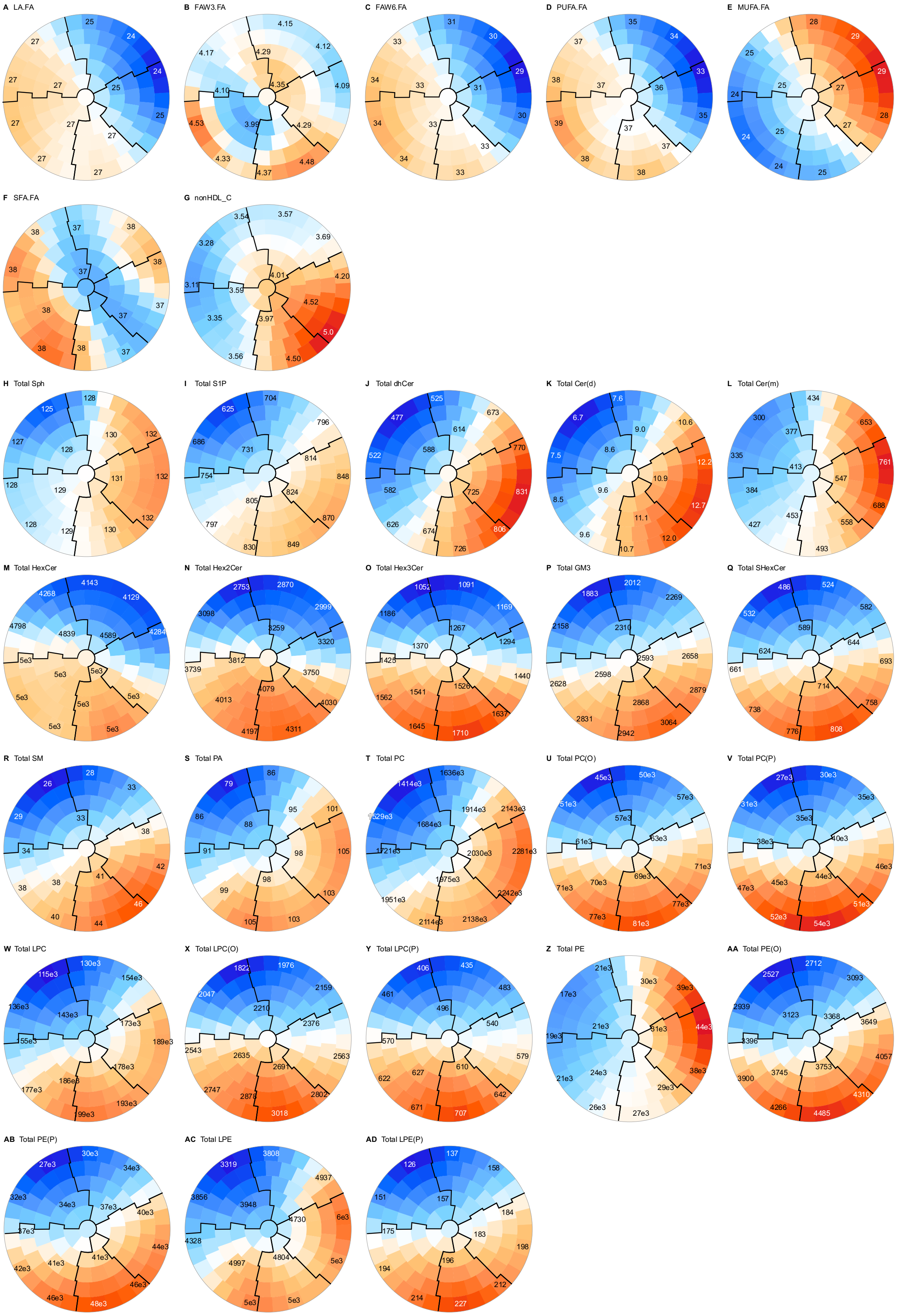
A LA.FA
B FAW3.FA
C FAW6.FA
D PUFA.FA
E MUFA.FA
F SFA.FA
G nonHDL_C
H Total Sph
I Total S1P
J Total dhCer
K Total Cer(d)
L Total Cer(m)
M Total HexCer
N Total Hex2Cer
O Total Hex3Cer
P Total GM3
Q Total SHexCer
R Total SM
S Total PA
T Total PC
U Total PC(O)
V Total PC(P)
W Total LPC
X Total LPC(O)
Y Total LPC(P)
Z Total PE
AA Total PE(O)
AB Total PE(P)
AC Total LPE
AD Total LPE(P)

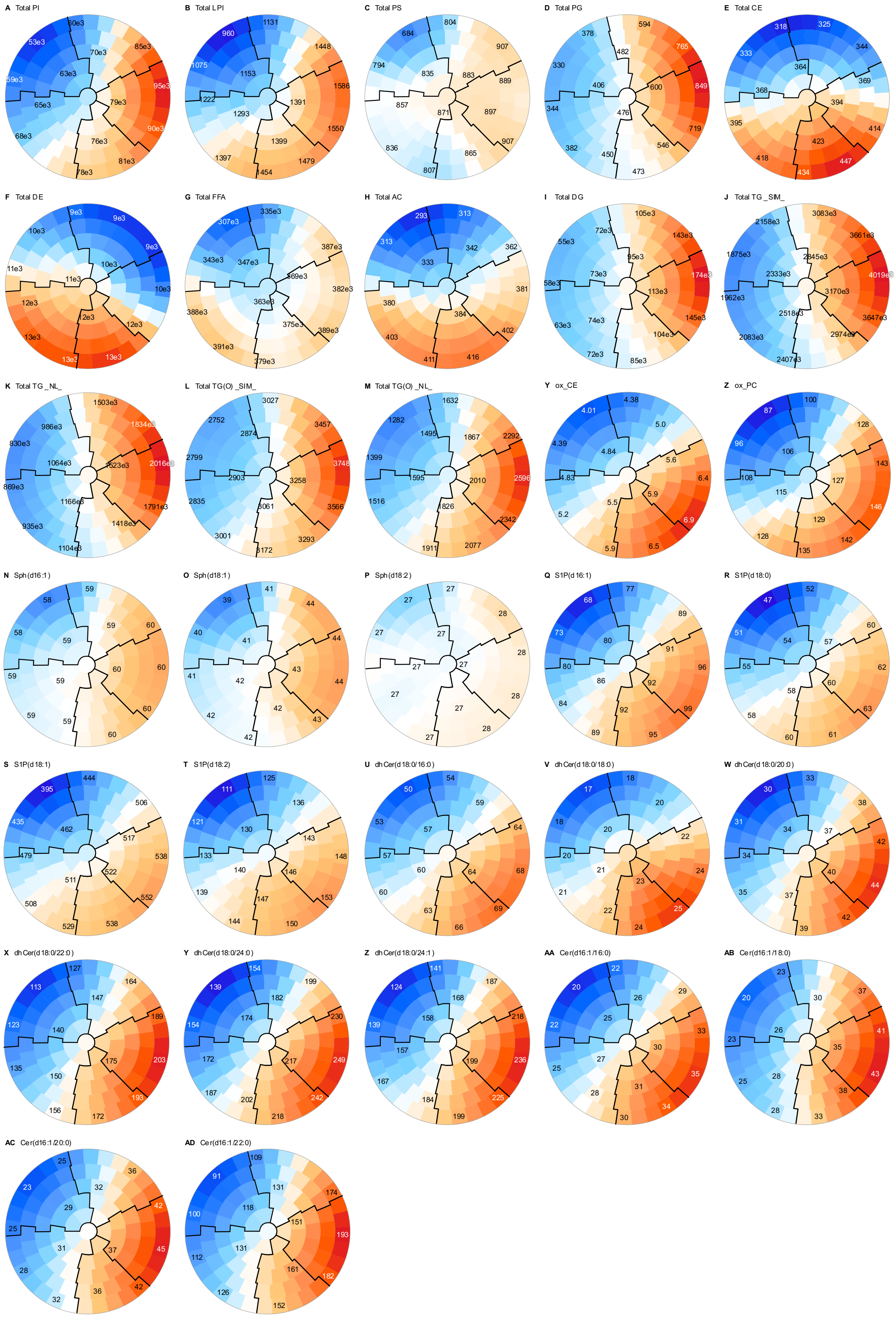
A Total PI
B Total LPI
C Total PS
D Total PG
E Total CE
F Total DE
G Total FFA
H Total AC
I Total DG
J Total TG _SIM_
K Total TG _NL_
L Total TG(O) _SIM_
M Total TG(O) _NL_
Y ox_CE
Z ox_PC
N Sph(d16:1)
O Sph(d18:1)
P Sph(d18:2)
Q S1P(d16:1)
R S1P(d18:0)
S S1P(d18:1)
T S1P(d18:2)
U dhCer(d18:0/16:0)
V dhCer(d18:0/18:0)
W dhCer(d18:0/20:0)
X dhCer(d18:0/22:0)
Y dhCer(d18:0/24:0)
Z dhCer(d18:0/24:1)
AA Cer(d16:1/16:0)
AB Cer(d16:1/18:0)
AC Cer(d16:1/20:0)
AD Cer(d16:1/22:0)

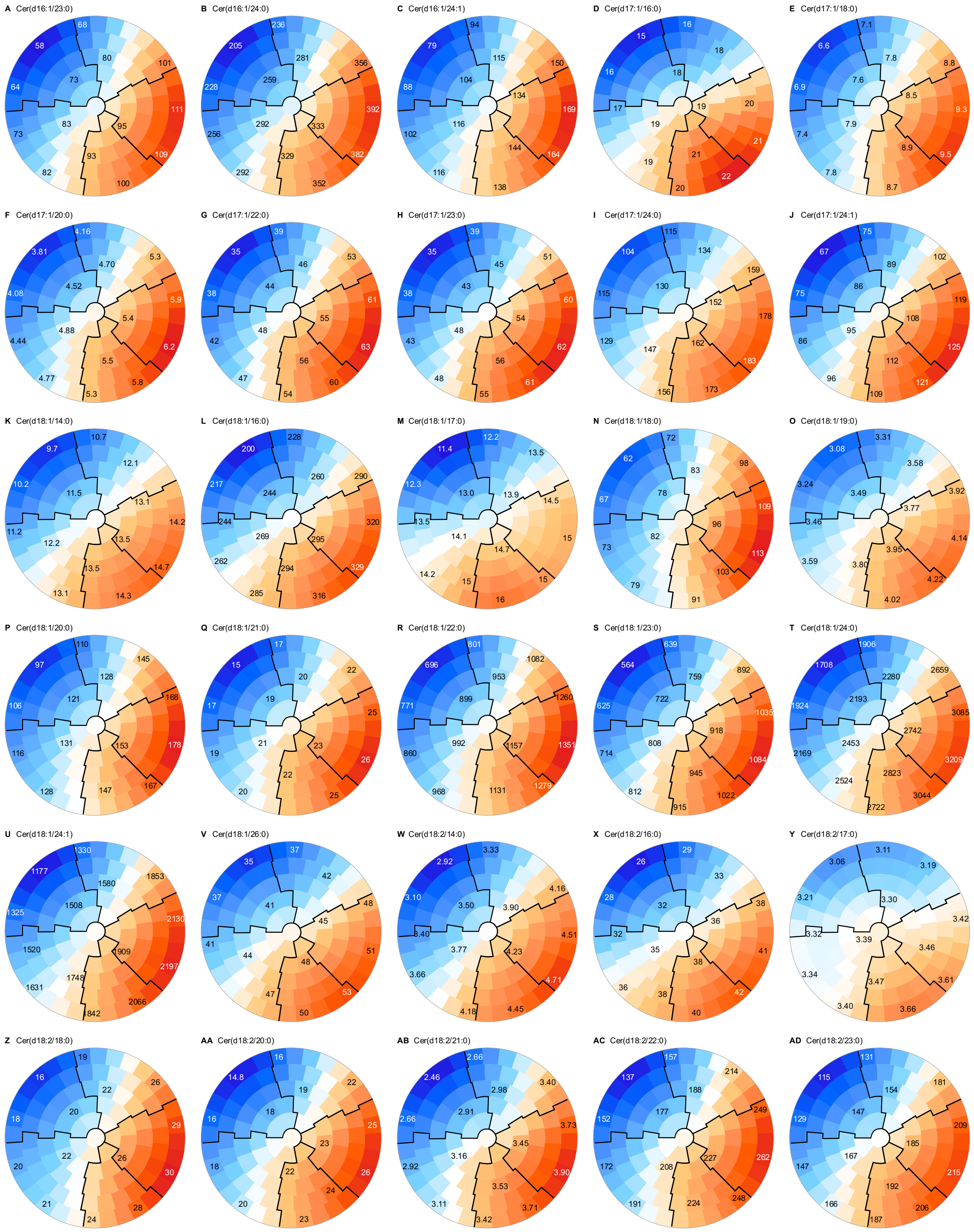
A Cer(d16:1/23:0)
B Cer(d16:1/24:0)
C Cer(d16:1/24:1)
D Cer(d17:1/16:0)
E Cer(d17:1/18:0)
F Cer(d17:1/20:0)
G Cer(d17:1/22:0)
H Cer(d17:1/23:0)
I Cer(d17:1/24:0)
J Cer(d17:1/24:1)
K Cer(d18:1/14:0)
L Cer(d18:1/16:0)
M Cer(d18:1/17:0)
N Cer(d18:1/18:0)
O Cer(d18:1/19:0)
P Cer(d18:1/20:0)
Q Cer(d18:1/21:0)
R Cer(d18:1/22:0)
S Cer(d18:1/23:0)
T Cer(d18:1/24:0)
U Cer(d18:1/24:1)
V Cer(d18:1/26:0)
W Cer(d18:2/14:0)
X Cer(d18:2/16:0)
Y Cer(d18:2/17:0)
Z Cer(d18:2/18:0)
AA Cer(d18:2/20:0)
AB Cer(d18:2/21:0)
AC Cer(d18:2/22:0)
AD Cer(d18:2/23:0)

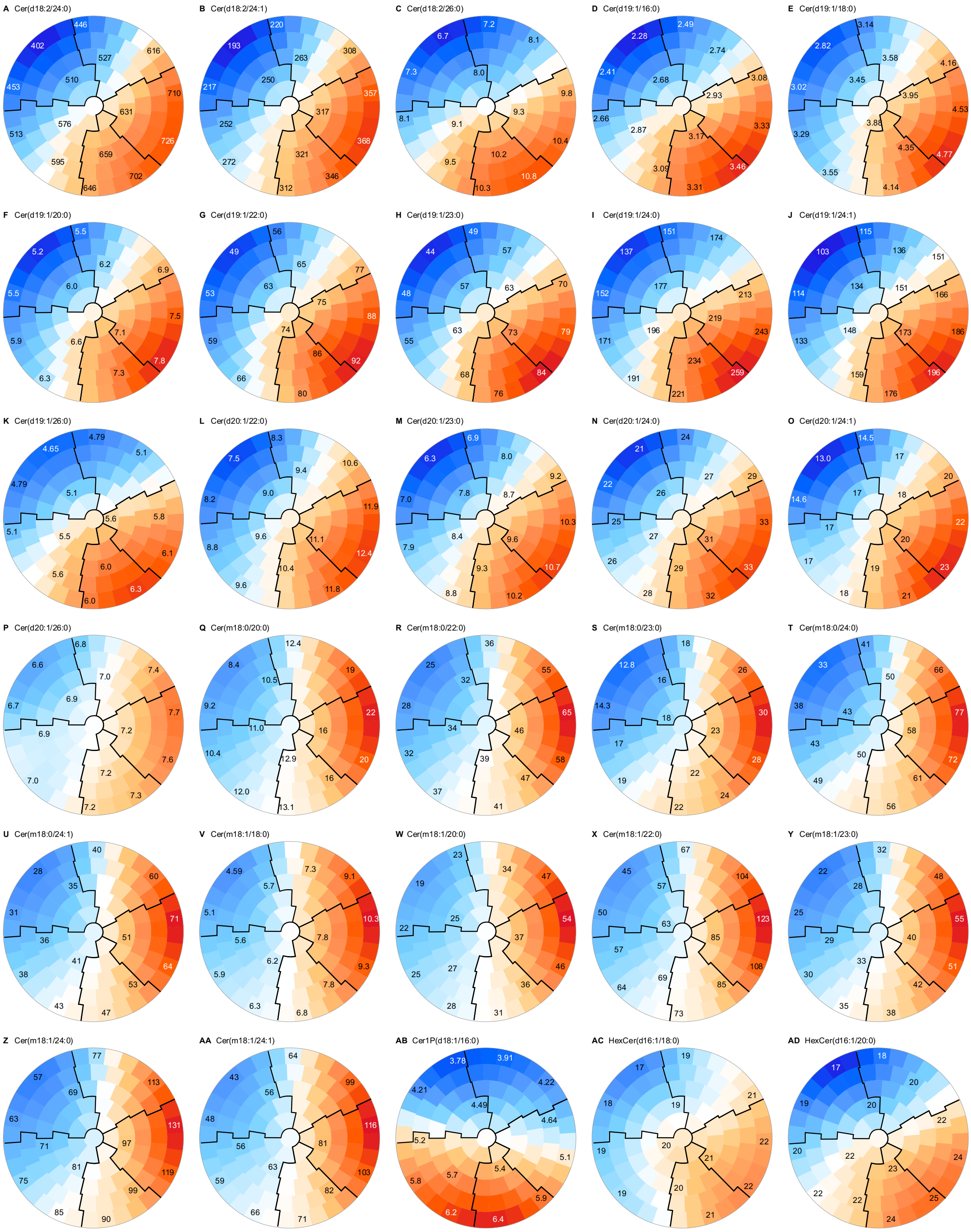

A Cer(d18:2/24:0)
B Cer(d18:2/24:1)
C Cer(d18:2/26:0)
D Cer(d19:1/16:0)
E Cer(d19:1/18:0)
F Cer(d19:1/20:0)
G Cer(d19:1/22:0)
H Cer(d19:1/23:0)
I Cer(d19:1/24:0)
J Cer(d19:1/24:1)
K Cer(d19:1/26:0)
L Cer(d20:1/22:0)
M Cer(d20:1/23:0)
N Cer(d20:1/24:0)
O Cer(d20:1/24:1)
P Cer(d20:1/26:0)
Q Cer(m18:0/20:0)
R Cer(m18:0/22:0)
S Cer(m18:0/23:0)
T Cer(m18:0/24:0)
U Cer(m18:0/24:1)
V Cer(m18:1/18:0)
W Cer(m18:1/20:0)
X Cer(m18:1/22:0)
Y Cer(m18:1/23:0)
Z Cer(m18:1/24:0)
AA Cer(m18:1/24:1)
AB Cer1P(d18:1/16:0)
AC HexCer(d16:1/18:0)
AD HexCer(d16:1/20:0)

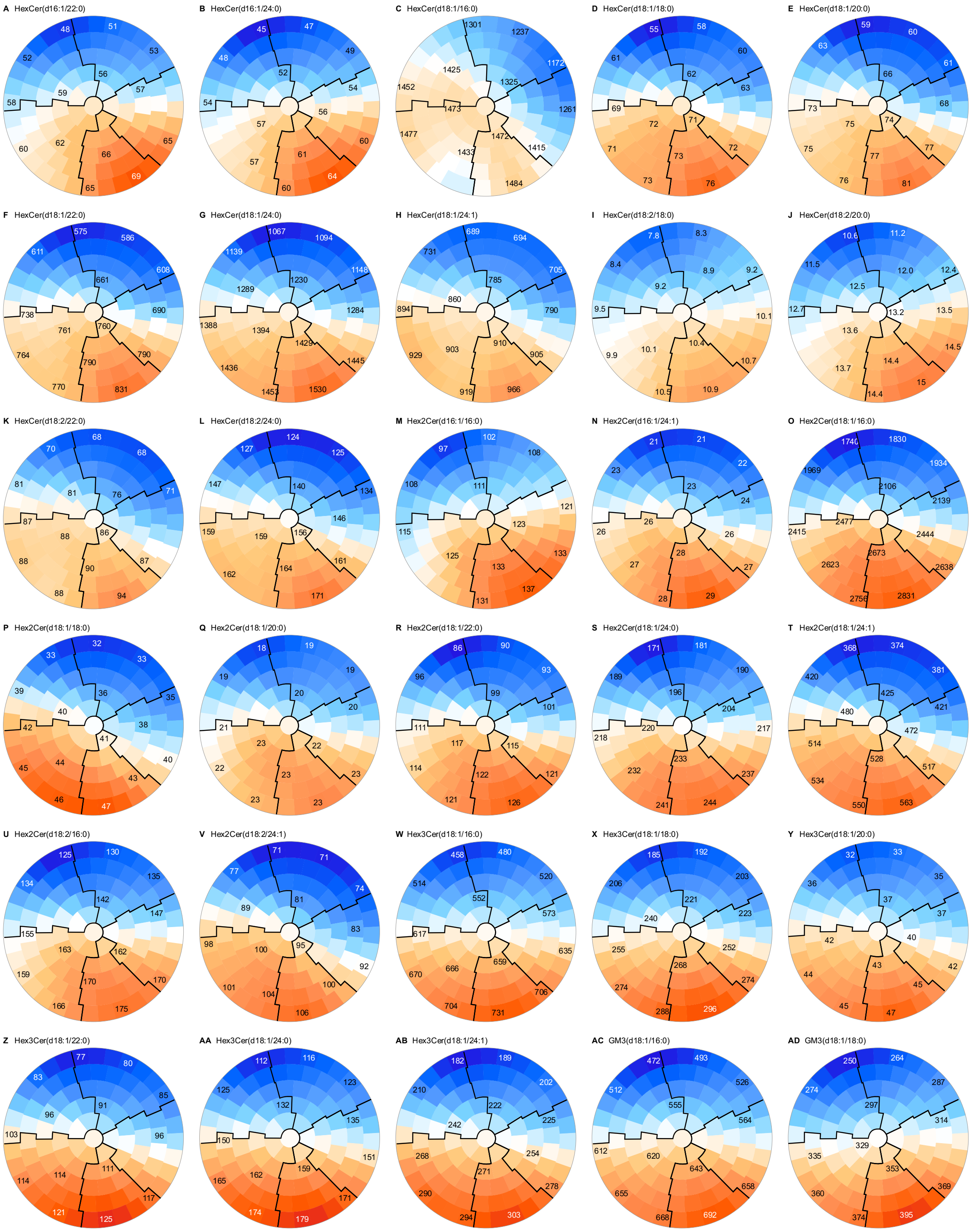
A HexCer(d16:1/22:0)
B HexCer(d16:1/24:0)
C HexCer(d18:1/16:0)
D HexCer(d18:1/18:0)
E HexCer(d18:1/20:0)
F HexCer(d18:1/22:0)
G HexCer(d18:1/24:0)
H HexCer(d18:1/24:1)
I HexCer(d18:2/18:0)
J HexCer(d18:2/20:0)
K HexCer(d18:2/22:0)
L HexCer(d18:2/24:0)
M Hex2Cer(d16:1/16:0)
N Hex2Cer(d16:1/24:1)
O Hex2Cer(d18:1/16:0)
P Hex2Cer(d18:1/18:0)
Q Hex2Cer(d18:1/20:0)
R Hex2Cer(d18:1/22:0)
S Hex2Cer(d18:1/24:0)
T Hex2Cer(d18:1/24:1)
U Hex2Cer(d18:2/16:0)
V Hex2Cer(d18:2/24:1)
W Hex3Cer(d18:1/16:0)
X Hex3Cer(d18:1/18:0)
Y Hex3Cer(d18:1/20:0)
Z Hex3Cer(d18:1/22:0)
AA Hex3Cer(d18:1/24:0)
AB Hex3Cer(d18:1/24:1)
AC GM3(d18:1/16:0)
AD GM3(d18:1/18:0)

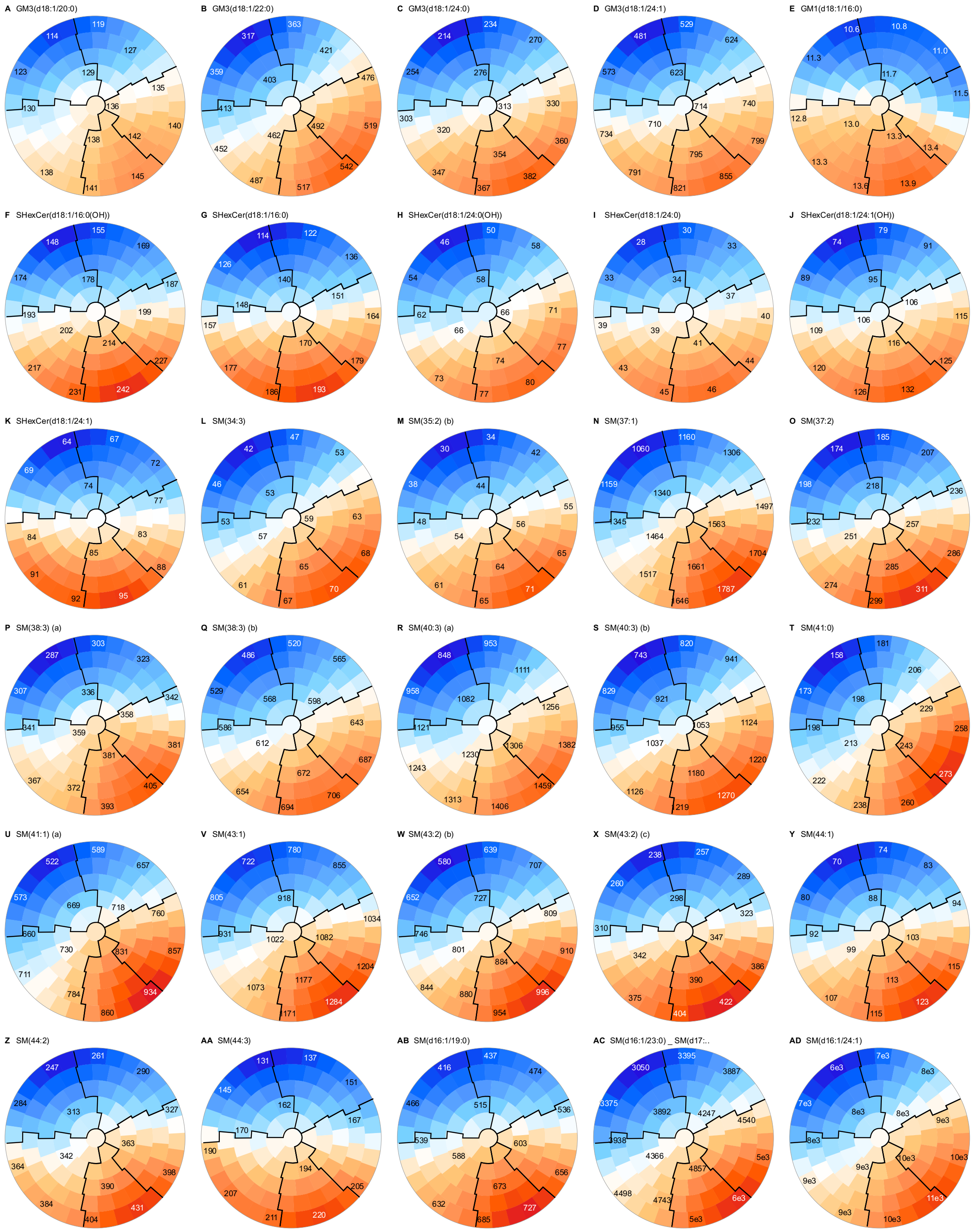
A GM3(d18:1/20:0)
B GM3(d18:1/22:0)
C GM3(d18:1/24:0)
D GM3(d18:1/24:1)
E GM1(d18:1/16:0)
F SHexCer(d18:1/16:0(OH))
G SHexCer(d18:1/16:0)
H SHexCer(d18:1/24:0(OH))
I SHexCer(d18:1/24:0)
J SHexCer(d18:1/24:1(OH))
K SHexCer(d18:1/24:1)
L SM(34:3)
M SM(35:2) (b)
N SM(37:1)
O SM(37:2)
P SM(38:3) (a)
Q SM(38:3) (b)
R SM(40:3) (a)
S SM(40:3) (b)
T SM(41:0)
U SM(41:1) (a)
V SM(43:1)
W SM(43:2) (b)
X SM(43:2) (c)
Y SM(44:1)
Z SM(44:2)
AA SM(44:3)
AB SM(d16:1/19:0)
AC SM(d16:1/23:0) _ SM(d17:..
AD SM(d16:1/24:1)

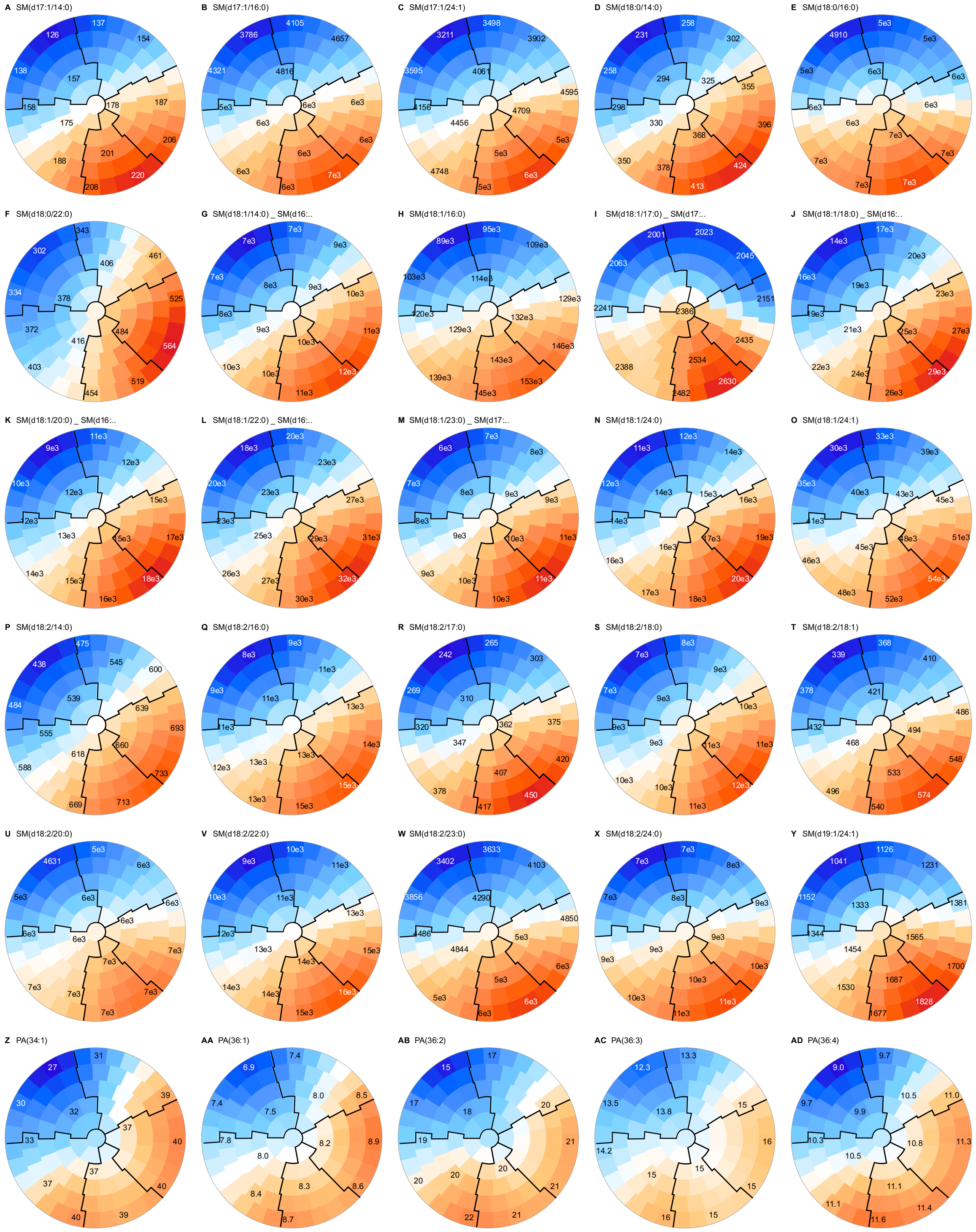

A SM(d17:1/14:0)
B SM(d17:1/16:0)
C SM(d17:1/24:1)
D SM(d18:0/14:0)
E SM(d18:0/16:0)
F SM(d18:0/22:0)
G SM(d18:1/14:0) _ SM(d16:..
H SM(d18:1/16:0)
I SM(d18:1/17:0) _ SM(d17:..
J SM(d18:1/18:0) _ SM(d16:..
K SM(d18:1/20:0) _ SM(d16:..
L SM(d18:1/22:0) _ SM(d16:..
M SM(d18:1/23:0) _ SM(d17:..
N SM(d18:1/24:0)
O SM(d18:1/24:1)
P SM(d18:2/14:0)
Q SM(d18:2/16:0)
R SM(d18:2/17:0)
S SM(d18:2/18:0)
T SM(d18:2/18:1)
U SM(d18:2/20:0)
V SM(d18:2/22:0)
W SM(d18:2/23:0)
X SM(d18:2/24:0)
Y SM(d19:1/24:1)
Z PA(34:1)
AA PA(36:1)
AB PA(36:2)
AC PA(36:3)
AD PA(36:4)

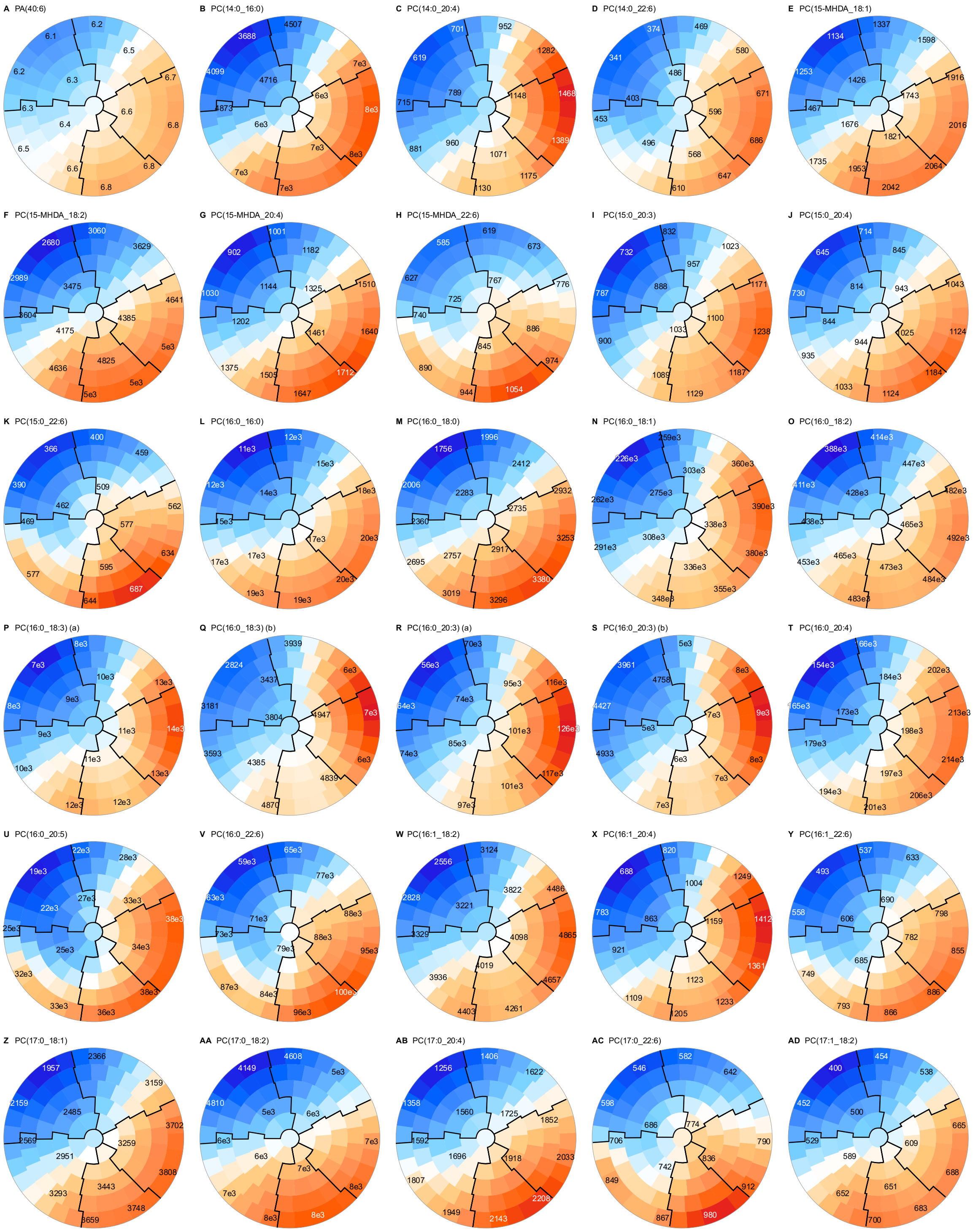
A PA(40:6)
B PC(14:0_16:0)
C PC(14:0_20:4)
D PC(14:0_22:6)
E PC(15-MHDA_18:1)
F PC(15-MHDA_18:2)
G PC(15-MHDA_20:4)
H PC(15-MHDA_22:6)
I PC(15:0_20:3)
J PC(15:0_20:4)
K PC(15:0_22:6)
L PC(16:0_16:0)
M PC(16:0_18:0)
N PC(16:0_18:1)
O PC(16:0_18:2)
P PC(16:0_18:3) (a)
Q PC(16:0_18:3) (b)
R PC(16:0_20:3) (a)
S PC(16:0_20:3) (b)
T PC(16:0_20:4)
U PC(16:0_20:5)
V PC(16:0_22:6)
W PC(16:1_18:2)
X PC(16:1_20:4)
Y PC(16:1_22:6)
Z PC(17:0_18:1)
AA PC(17:0_18:2)
AB PC(17:0_20:4)
AC PC(17:0_22:6)
AD PC(17:1_18:2)

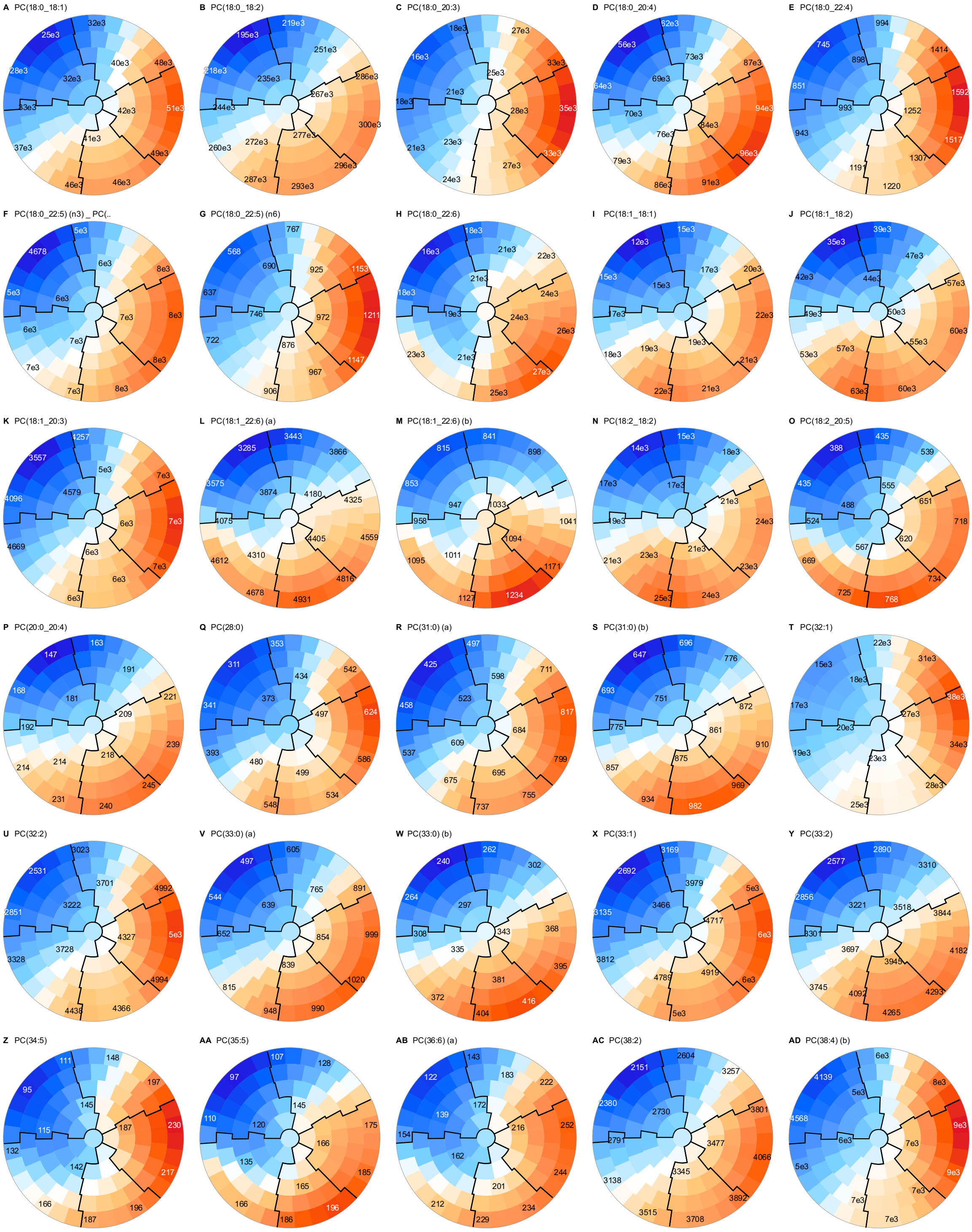
A PC(18:0_18:1)
25e3 32e3 28e3 32e3 40e3 48e3 33e3 42e3 51e3 37e3 41e3 49e3 46e3 46e3
B PC(18:0_18:2)
195e3 219e3 251e3 218e3 235e3 286e3 267e3 244e3 300e3 260e3 272e3 277e3 296e3 287e3 293e3
C PC(18:0_20:3)
18e3 27e3 16e3 25e3 33e3 18e3 21e3 28e3 35e3 21e3 23e3 33e3 27e3 24e3
D PC(18:0_20:4)
62e3 56e3 73e3 87e3 64e3 69e3 70e3 94e3 76e3 84e3 96e3 79e3 86e3 91e3
E PC(18:0_22:4)
994 745 898 1414 851 993 1252 1592 943 1517 1191 1307 1220
F PC(18:0_22:5) (n3) _ PC(..
5e3 4678 6e3 8e3 5e3 6e3 7e3 8e3 6e3 7e3 8e3 7e3 7e3 8e3
G PC(18:0_22:5) (n6)
767 568 690 925 1153 637 746 972 1211 722 876 1147 967 906
H PC(18:0_22:6)
18e3 16e3 21e3 22e3 21e3 18e3 19e3 24e3 24e3 26e3 23e3 21e3 27e3 25e3
I PC(18:1_18:1)
15e3 12e3 17e3 20e3 15e3 15e3 17e3 22e3 18e3 19e3 19e3 21e3 22e3 21e3
J PC(18:1_18:2)
39e3 35e3 47e3 42e3 44e3 57e3 50e3 49e3 60e3 53e3 57e3 55e3 63e3 60e3
K PC(18:1_20:3)
4257 3557 5e3 7e3 4096 4579 6e3 7e3 4669 6e3 7e3 6e3 6e3
L PC(18:1_22:6) (a)
3285 3443 3866 3575 3874 4180 4325 4075 4405 4559 4612 4310 4816 4678 4931
M PC(18:1_22:6) (b)
815 841 898 853 947 1033 958 1041 1094 1095 1011 1171 1127 1234
N PC(18:2_18:2)
14e3 15e3 18e3 17e3 17e3 21e3 19e3 24e3 21e3 23e3 21e3 23e3 25e3 24e3
O PC(18:2_20:5)
388 435 539 435 555 488 651 524 718 620 567 669 734 725 768
P PC(20:0_20:4)
147 163 191 168 181 221 192 209 239 214 214 218 245 231 240
Q PC(28:0)
353 311 434 542 373 624 341 497 393 480 586 499 534 548
R PC(31:0) (a)
497 425 598 711 458 523 817 684 537 609 799 675 695 755 737
S PC(31:0) (b)
647 696 776 693 751 872 775 861 910 857 875 969 934 982
T PC(32:1)
22e3 15e3 31e3 18e3 38e3 17e3 20e3 27e3 34e3 19e3 23e3 28e3 25e3
U PC(32:2)
3023 2531 3701 4992 2851 3222 4327 5e3 3328 3728 4994 4438 4366
V PC(33:0) (a)
497 605 765 891 544 639 552 854 999 839 815 1020 948 990
W PC(33:0) (b)
240 262 302 264 297 308 343 368 335 395 381 372 416 404
X PC(33:1)
3169 2692 3979 5e3 3135 3466 4717 6e3 3812 4789 4919 6e3 5e3
Y PC(33:2)
2577 2890 3310 2856 3221 3518 3844 3301 3697 3945 4182 3745 4092 4293 4265
Z PC(34:5)
111 148 95 197 145 187 230 115 132 142 217 166 196 187
AA PC(35:5)
107 97 128 145 110 120 175 166 135 185 165 166 196 186
AB PC(36:6) (a)
143 122 183 222 139 172 216 252 154 162 244 201 212 234 229
AC PC(38:2)
2151 2604 3257 2380 2730 3801 2791 3477 4066 3345 3138 3892 3515 3708
AD PC(38:4) (b)
6e3 4139 8e3 5e3 4568 6e3 9e3 7e3 5e3 9e3 7e3 7e3 7e3

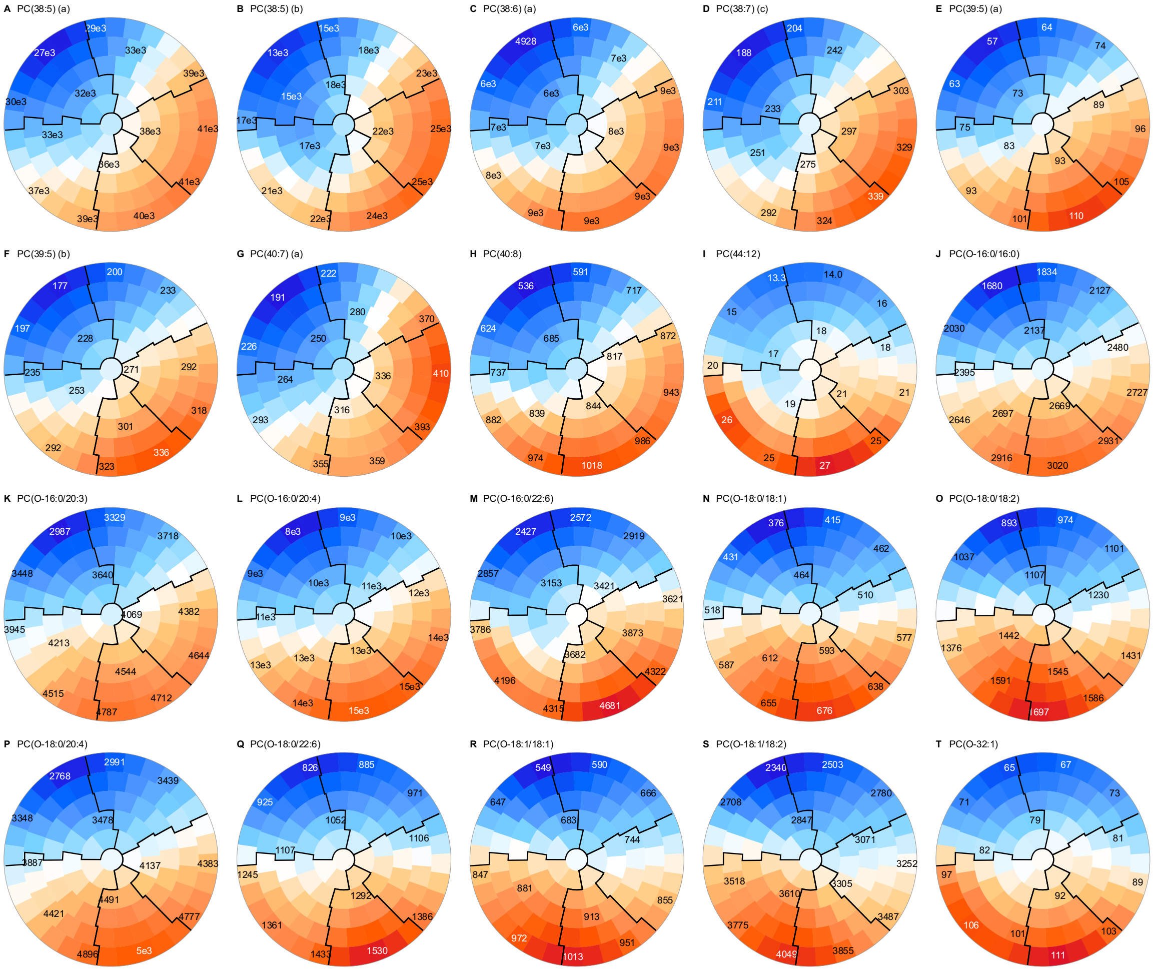
A PC(38:5) (a)
B PC(38:5) (b)
C PC(38:6) (a)
D PC(38:7) (c)
E PC(39:5) (a)
F PC(39:5) (b)
G PC(40:7) (a)
H PC(40:8)
I PC(44:12)
J PC(O-16:0/16:0)
K PC(O-16:0/20:3)
L PC(O-16:0/20:4)
M PC(O-16:0/22:6)
N PC(O-18:0/18:1)
O PC(O-18:0/18:2)
P PC(O-18:0/20:4)
Q PC(O-18:0/22:6)
R PC(O-18:1/18:1)
S PC(O-18:1/18:2)
T PC(O-32:1)

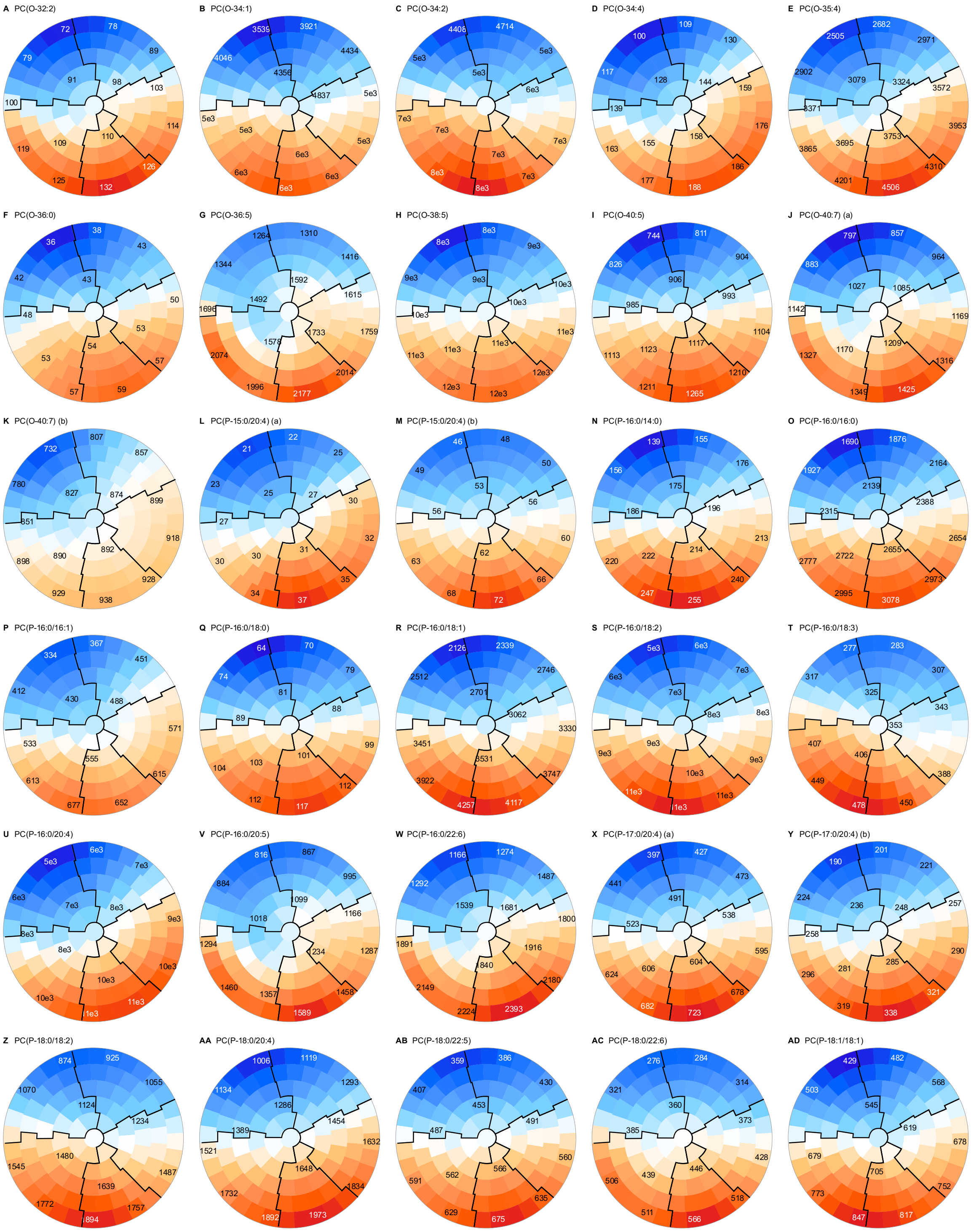
A PC(O-32:2)
B PC(O-34:1)
C PC(O-34:2)
D PC(O-34:4)
E PC(O-35:4)
F PC(O-36:0)
G PC(O-36:5)
H PC(O-38:5)
I PC(O-40:5)
J PC(O-40:7) (a)
K PC(O-40:7) (b)
L PC(P-15:0/20:4) (a)
M PC(P-15:0/20:4) (b)
N PC(P-16:0/14:0)
O PC(P-16:0/16:0)
P PC(P-16:0/16:1)
Q PC(P-16:0/18:0)
R PC(P-16:0/18:1)
S PC(P-16:0/18:2)
T PC(P-16:0/18:3)
U PC(P-16:0/20:4)
V PC(P-16:0/20:5)
W PC(P-16:0/22:6)
X PC(P-17:0/20:4) (a)
Y PC(P-17:0/20:4) (b)
Z PC(P-18:0/18:2)
AA PC(P-18:0/20:4)
AB PC(P-18:0/22:5)
AC PC(P-18:0/22:6)
AD PC(P-18:1/18:1)

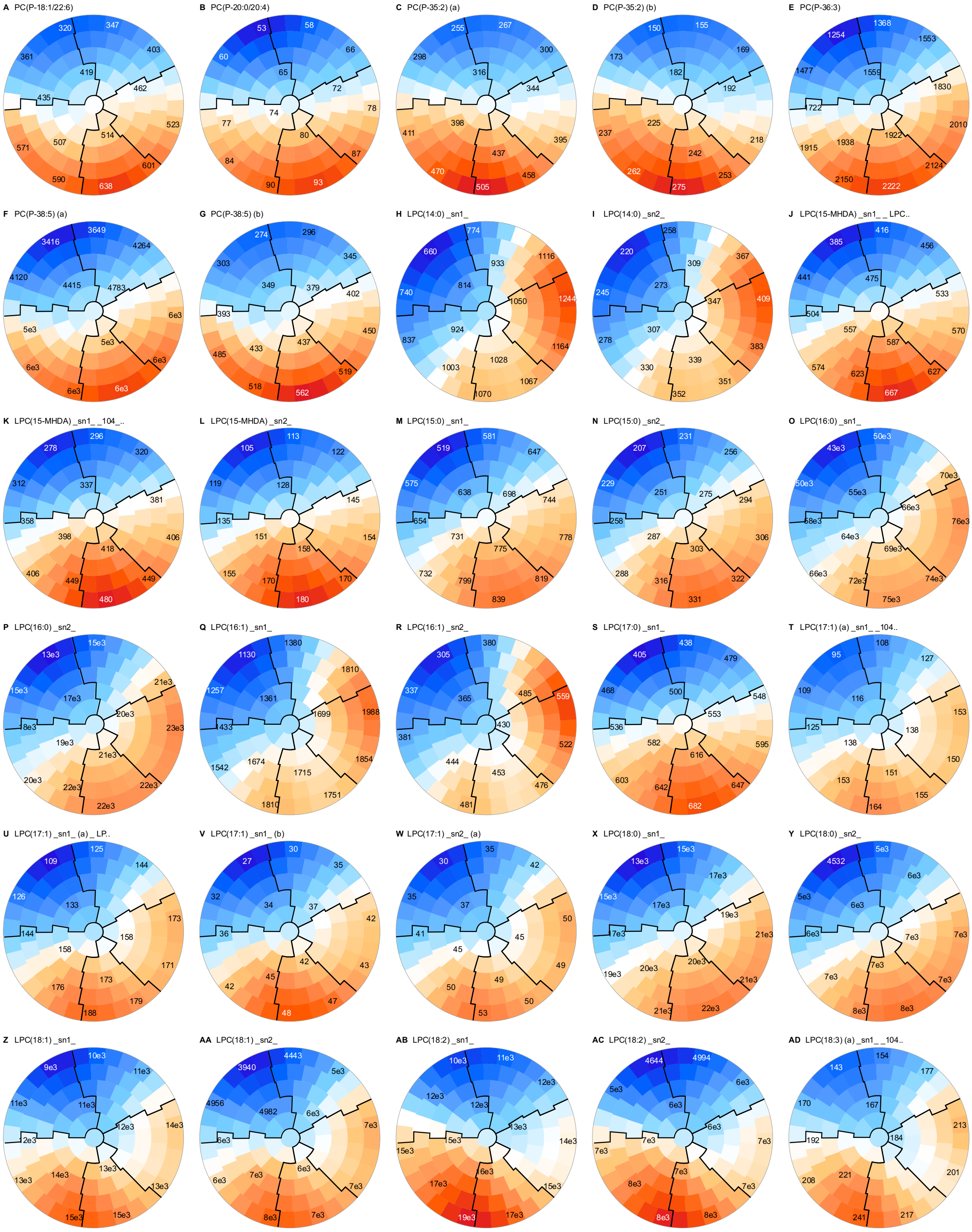
A PC(P-18:1/22:6)
B PC(P-20:0/20:4)
C PC(P-35:2) (a)
D PC(P-35:2) (b)
E PC(P-36:3)
F PC(P-38:5) (a)
G PC(P-38:5) (b)
H LPC(14:0) _sn1_
I LPC(14:0) _sn2_
J LPC(15-MHDA) _sn1_ _LPC..
K LPC(15-MHDA) _sn1_ _104_..
L LPC(15-MHDA) _sn2_
M LPC(15:0) _sn1_
N LPC(15:0) _sn2_
O LPC(16:0) _sn1_
P LPC(16:0) _sn2_
Q LPC(16:1) _sn1_
R LPC(16:1) _sn2_
S LPC(17:0) _sn1_
T LPC(17:1) (a) _sn1_ _104..
U LPC(17:1) _sn1_ (a) _ LP..
V LPC(17:1) _sn1_ (b)
W LPC(17:1) _sn2_ (a)
X LPC(18:0) _sn1_
Y LPC(18:0) _sn2_
Z LPC(18:1) _sn1_
AA LPC(18:1) _sn2_
AB LPC(18:2) _sn1_
AC LPC(18:2) _sn2_
AD LPC(18:3) (a) _sn1_ _104..

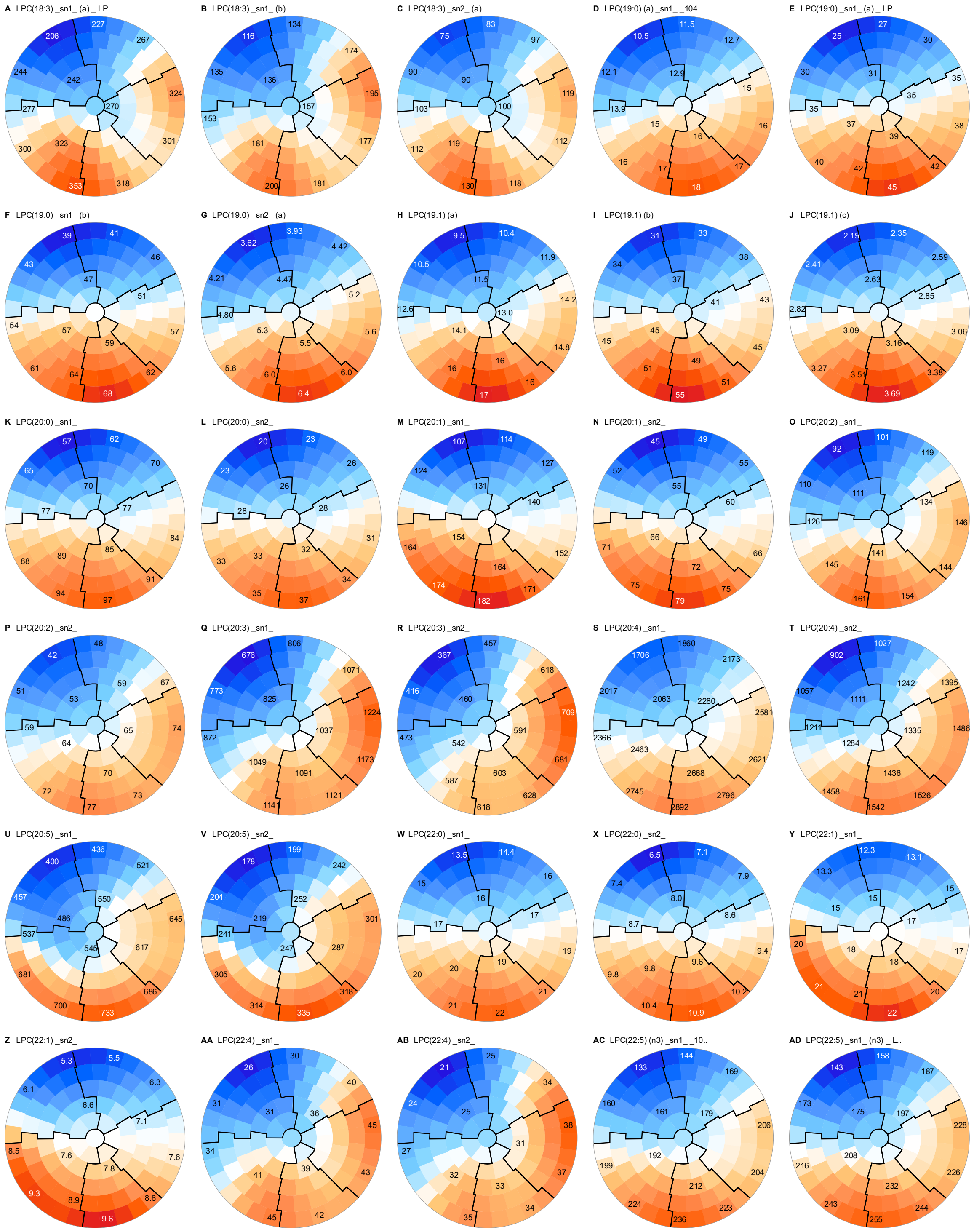
A LPC(18:3) _sn1_ (a) _ LP..
B LPC(18:3) _sn1_ (b)
C LPC(18:3) _sn2_ (a)
D LPC(19:0) (a) _sn1_ _104..
E LPC(19:0) _sn1_ (a) _ LP..
F LPC(19:0) _sn1_ (b)
G LPC(19:0) _sn2_ (a)
H LPC(19:1) (a)
I LPC(19:1) (b)
J LPC(19:1) (c)
K LPC(20:0) _sn1_
L LPC(20:0) _sn2_
M LPC(20:1) _sn1_
N LPC(20:1) _sn2_
O LPC(20:2) _sn1_
P LPC(20:2) _sn2_
Q LPC(20:3) _sn1_
R LPC(20:3) _sn2_
S LPC(20:4) _sn1_
T LPC(20:4) _sn2_
U LPC(20:5) _sn1_
V LPC(20:5) _sn2_
W LPC(22:0) _sn1_
X LPC(22:0) _sn2_
Y LPC(22:1) _sn1_
Z LPC(22:1) _sn2_
AA LPC(22:4) _sn1_
AB LPC(22:4) _sn2_
AC LPC(22:5) (n3) _sn1_ _10..
AD LPC(22:5) _sn1_ (n3) _ L..

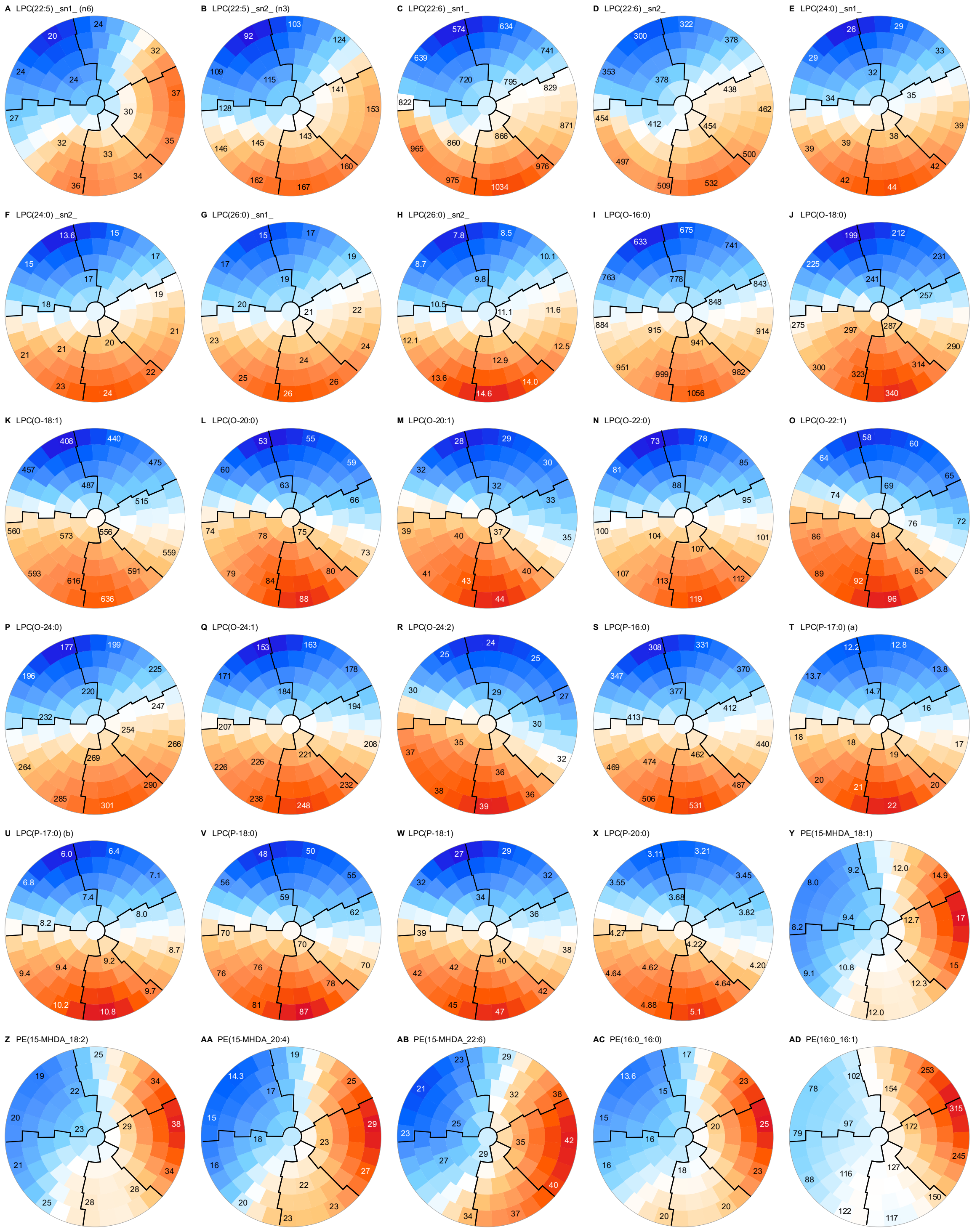
A LPC(22:5) _sn1_ (n6)
B LPC(22:5) _sn2_ (n3)
C LPC(22:6) _sn1_
D LPC(22:6) _sn2_
E LPC(24:0) _sn1_
F LPC(24:0) _sn2_
G LPC(26:0) _sn1_
H LPC(26:0) _sn2_
I LPC(O-16:0)
J LPC(O-18:0)
K LPC(O-18:1)
L LPC(O-20:0)
M LPC(O-20:1)
N LPC(O-22:0)
O LPC(O-22:1)
P LPC(O-24:0)
Q LPC(O-24:1)
R LPC(O-24:2)
S LPC(P-16:0)
T LPC(P-17:0) (a)
U LPC(P-17:0) (b)
V LPC(P-18:0)
W LPC(P-18:1)
X LPC(P-20:0)
Y PE(15-MHDA_18:1)
Z PE(15-MHDA_18:2)
AA PE(15-MHDA_20:4)
AB PE(15-MHDA_22:6)
AC PE(16:0_16:0)
AD PE(16:0_16:1)

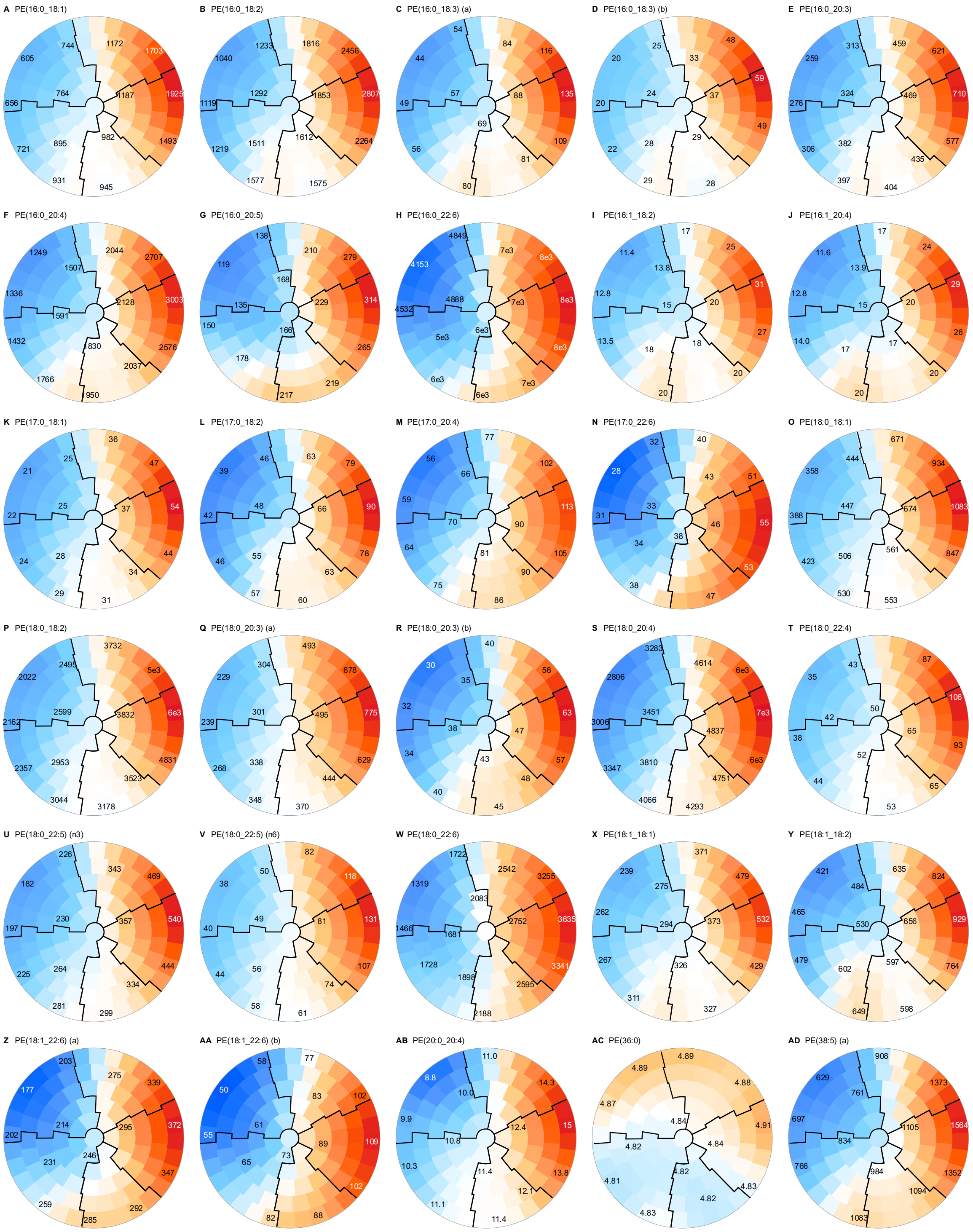
A PE(16:0_18:1)
744 1172 605 1703 764 1187 1925 656 895 982 721 1493 931 945
B PE(16:0_18:2)
1233 1816 1040 2456 1292 1853 2807 1119 1511 1612 1219 2264 1577 1575
C PE(16:0_18:3) (a)
54 84 44 116 57 88 135 49 69 56 109 81 80
D PE(16:0_18:3) (b)
25 48 20 33 59 24 37 20 49 22 28 29 29 28
E PE(16:0_20:3)
313 459 259 621 324 469 710 276 306 382 577 435 397 404
F PE(16:0_20:4)
1249 2044 2707 1507 1336 2128 3003 591 1432 830 2576 2037 1766 950
G PE(16:0_20:5)
138 210 119 279 168 229 314 135 150 166 178 265 219 217
H PE(16:0_22:6)
4849 7e3 8e3 4153 4888 7e3 8e3 4532 6e3 5e3 8e3 6e3 6e3 7e3
I PE(16:1_18:2)
17 11.4 25 13.8 31 12.8 15 20 13.5 18 18 27 20 20
J PE(16:1_20:4)
17 11.6 24 13.9 29 12.8 15 20 14.0 17 17 26 20 20
K PE(17:0_18:1)
36 25 21 47 25 37 54 22 28 24 44 34 29 31
L PE(17:0_18:2)
63 46 39 79 48 66 90 42 55 46 78 63 57 60
M PE(17:0_20:4)
77 56 66 102 59 70 90 113 64 81 105 90 75 86
N PE(17:0_22:6)
32 40 28 43 51 33 31 46 55 38 34 53 38 47
O PE(18:0_18:1)
671 444 358 934 447 674 1083 388 561 506 423 847 530 553
P PE(18:0_18:2)
3732 2495 2022 5e3 2599 3832 6e3 2162 2953 2357 4831 3523 3044 3178
Q PE(18:0_20:3) (a)
493 304 229 678 301 495 775 239 338 268 629 444 348 370
R PE(18:0_20:3) (b)
40 30 35 56 32 38 47 63 34 43 57 48 40 45
S PE(18:0_20:4)
3283 4614 6e3 2806 3451 4837 7e3 3006 3810 3347 6e3 4751 4066 4293
T PE(18:0_22:4)
43 87 35 106 50 42 65 38 52 93 44 65 53
U PE(18:0_22:5) (n3)
226 343 182 469 230 357 540 197 264 225 444 334 281 299
V PE(18:0_22:5) (n6)
82 50 38 118 49 81 131 40 56 44 107 74 58 61
W PE(18:0_22:6)
1722 2542 3255 1319 2083 2752 3635 1466 1681 1728 3341 1898 2595 2188
X PE(18:1_18:1)
371 239 275 479 262 294 373 532 267 326 429 311 327
Y PE(18:1_18:2)
635 421 484 824 465 530 656 929 479 597 602 764 649 598
Z PE(18:1_22:6) (a)
203 275 177 339 214 295 372 202 231 246 347 259 292 285
AA PE(18:1_22:6) (b)
58 77 50 83 102 61 55 89 109 73 65 102 82 88
AB PE(20:0_20:4)
11.0 8.8 14.3 10.0 9.9 12.4 15 10.8 10.3 11.4 13.8 12.1 11.1 11.4
AC PE(36:0)
4.89 4.89 4.88 4.87 4.84 4.91 4.82 4.84 4.82 4.81 4.83 4.82 4.83
AD PE(38:5) (a)
908 629 1373 761 697 1105 1564 834 766 984 1352 1094 1083

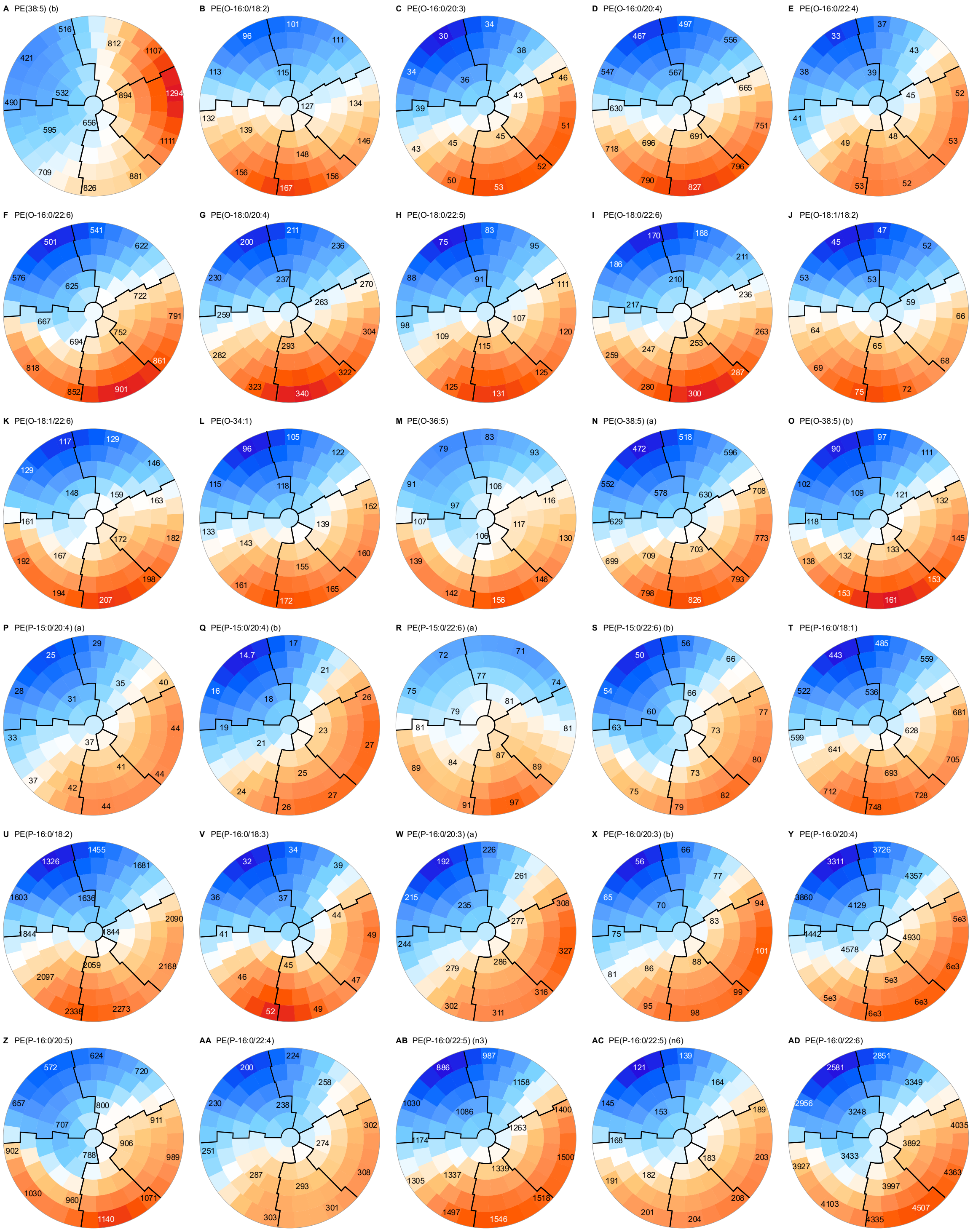
A PE(38:5) (b)
B PE(O-16:0/18:2)
C PE(O-16:0/20:3)
D PE(O-16:0/20:4)
E PE(O-16:0/22:4)
F PE(O-16:0/22:6)
G PE(O-18:0/20:4)
H PE(O-18:0/22:5)
I PE(O-18:0/22:6)
J PE(O-18:1/18:2)
K PE(O-18:1/22:6)
L PE(O-34:1)
M PE(O-36:5)
N PE(O-38:5) (a)
O PE(O-38:5) (b)
P PE(P-15:0/20:4) (a)
Q PE(P-15:0/20:4) (b)
R PE(P-15:0/22:6) (a)
S PE(P-15:0/22:6) (b)
T PE(P-16:0/18:1)
U PE(P-16:0/18:2)
V PE(P-16:0/18:3)
W PE(P-16:0/20:3) (a)
X PE(P-16:0/20:3) (b)
Y PE(P-16:0/20:4)
Z PE(P-16:0/20:5)
AA PE(P-16:0/22:4)
AB PE(P-16:0/22:5) (n3)
AC PE(P-16:0/22:5) (n6)
AD PE(P-16:0/22:6)

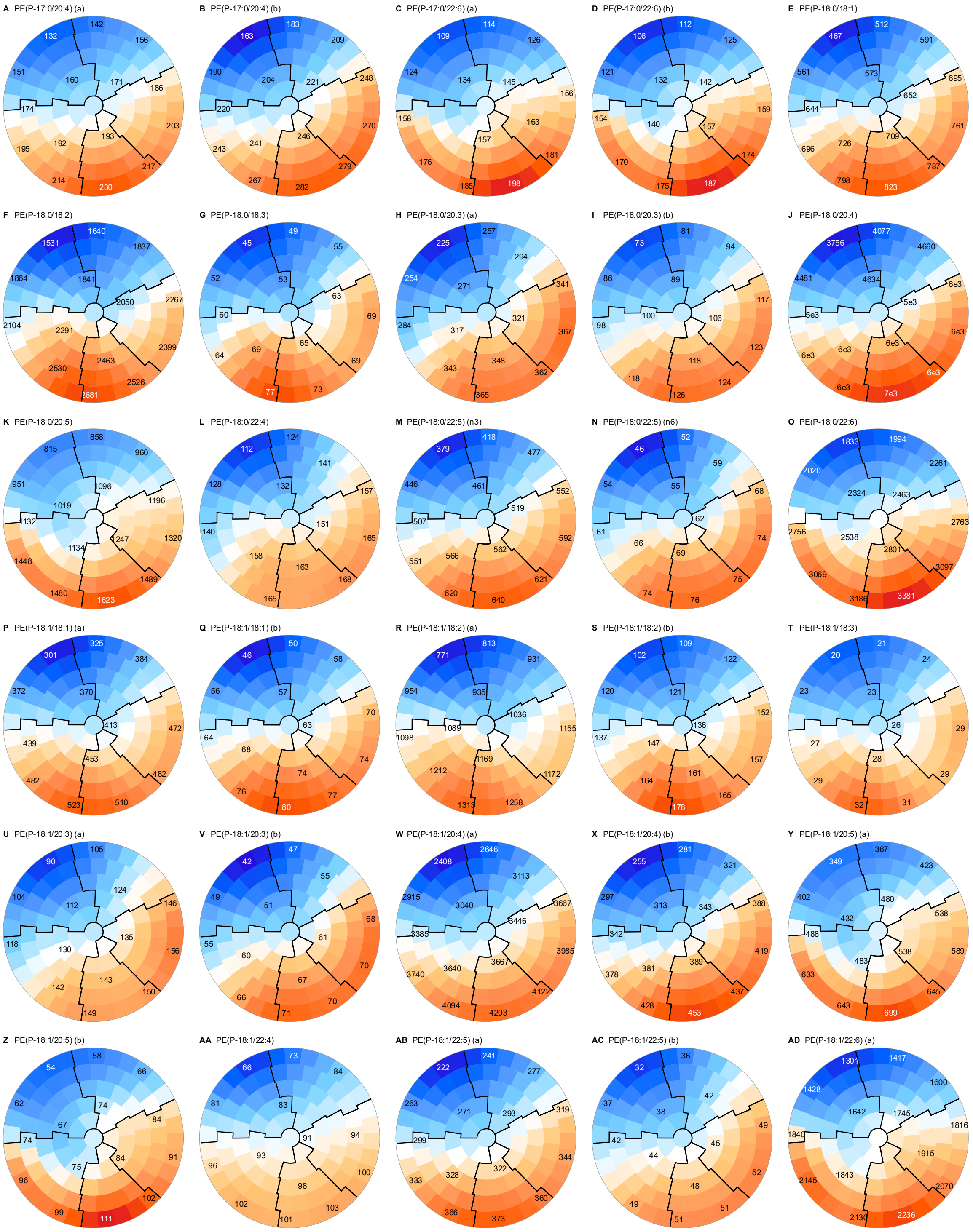
A PE(P-17:0/20:4) (a)
B PE(P-17:0/20:4) (b)
C PE(P-17:0/22:6) (a)
D PE(P-17:0/22:6) (b)
E PE(P-18:0/18:1)
F PE(P-18:0/18:2)
G PE(P-18:0/18:3)
H PE(P-18:0/20:3) (a)
I PE(P-18:0/20:3) (b)
J PE(P-18:0/20:4)
K PE(P-18:0/20:5)
L PE(P-18:0/22:4)
M PE(P-18:0/22:5) (n3)
N PE(P-18:0/22:5) (n6)
O PE(P-18:0/22:6)
P PE(P-18:1/18:1) (a)
Q PE(P-18:1/18:1) (b)
R PE(P-18:1/18:2) (a)
S PE(P-18:1/18:2) (b)
T PE(P-18:1/18:3)
U PE(P-18:1/20:3) (a)
V PE(P-18:1/20:3) (b)
W PE(P-18:1/20:4) (a)
X PE(P-18:1/20:4) (b)
Y PE(P-18:1/20:5) (a)
Z PE(P-18:1/20:5) (b)
AA PE(P-18:1/22:4)
AB PE(P-18:1/22:5) (a)
AC PE(P-18:1/22:5) (b)
AD PE(P-18:1/22:6) (a)

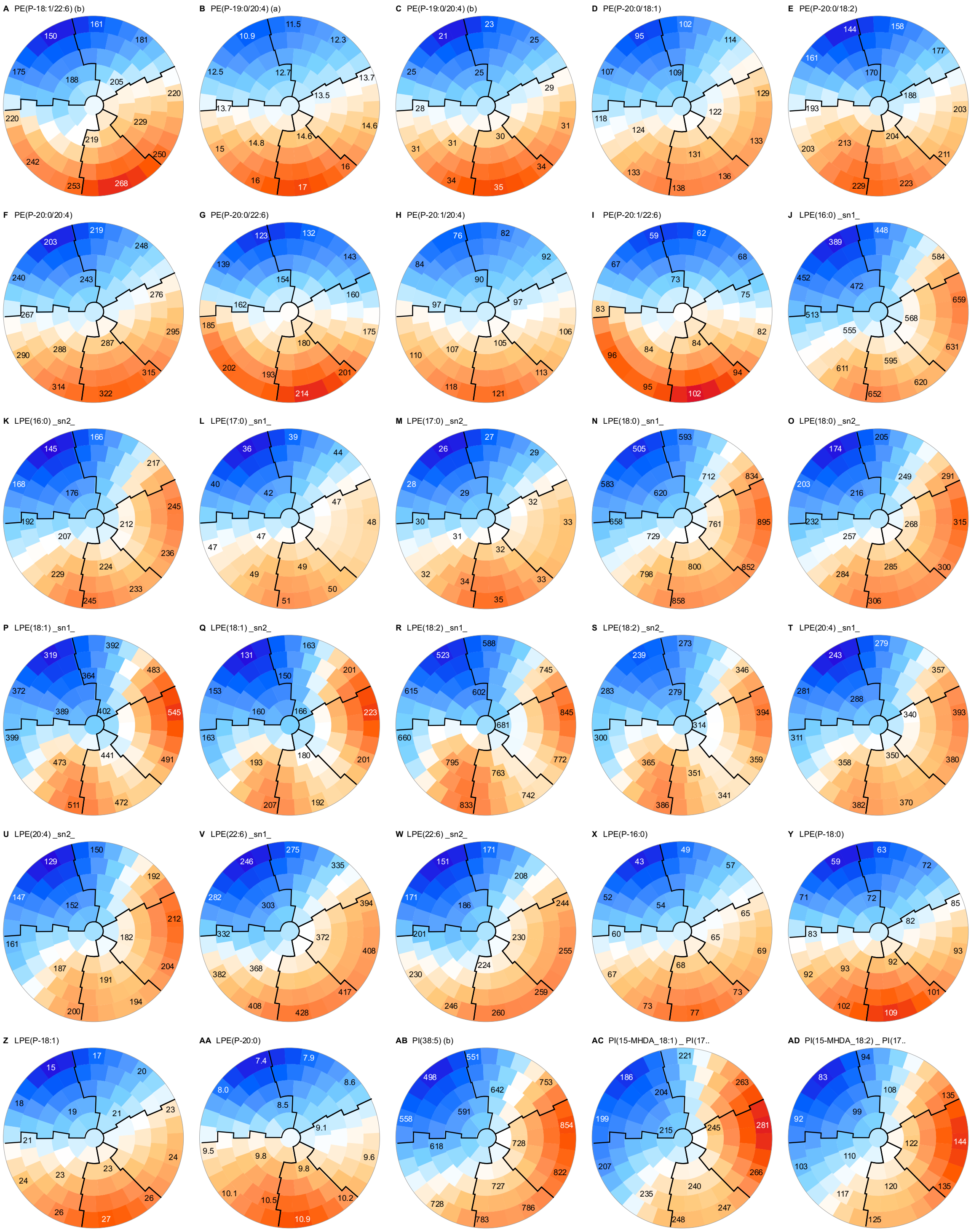
A PE(P-18:1/22:6) (b)
B PE(P-19:0/20:4) (a)
C PE(P-19:0/20:4) (b)
D PE(P-20:0/18:1)
E PE(P-20:0/18:2)
F PE(P-20:0/20:4)
G PE(P-20:0/22:6)
H PE(P-20:1/20:4)
I PE(P-20:1/22:6)
J LPE(16:0) _sn1_
K LPE(16:0) _sn2_
L LPE(17:0) _sn1_
M LPE(17:0) _sn2_
N LPE(18:0) _sn1_
O LPE(18:0) _sn2_
P LPE(18:1) _sn1_
Q LPE(18:1) _sn2_
R LPE(18:2) _sn1_
S LPE(18:2) _sn2_
T LPE(20:4) _sn1_
U LPE(20:4) _sn2_
V LPE(22:6) _sn1_
W LPE(22:6) _sn2_
X LPE(P-16:0)
Y LPE(P-18:0)
Z LPE(P-18:1)
AA LPE(P-20:0)
AB PI(38:5) (b)
AC PI(15-MHDA_18:1) _ PI(17..
AD PI(15-MHDA_18:2) _ PI(17..

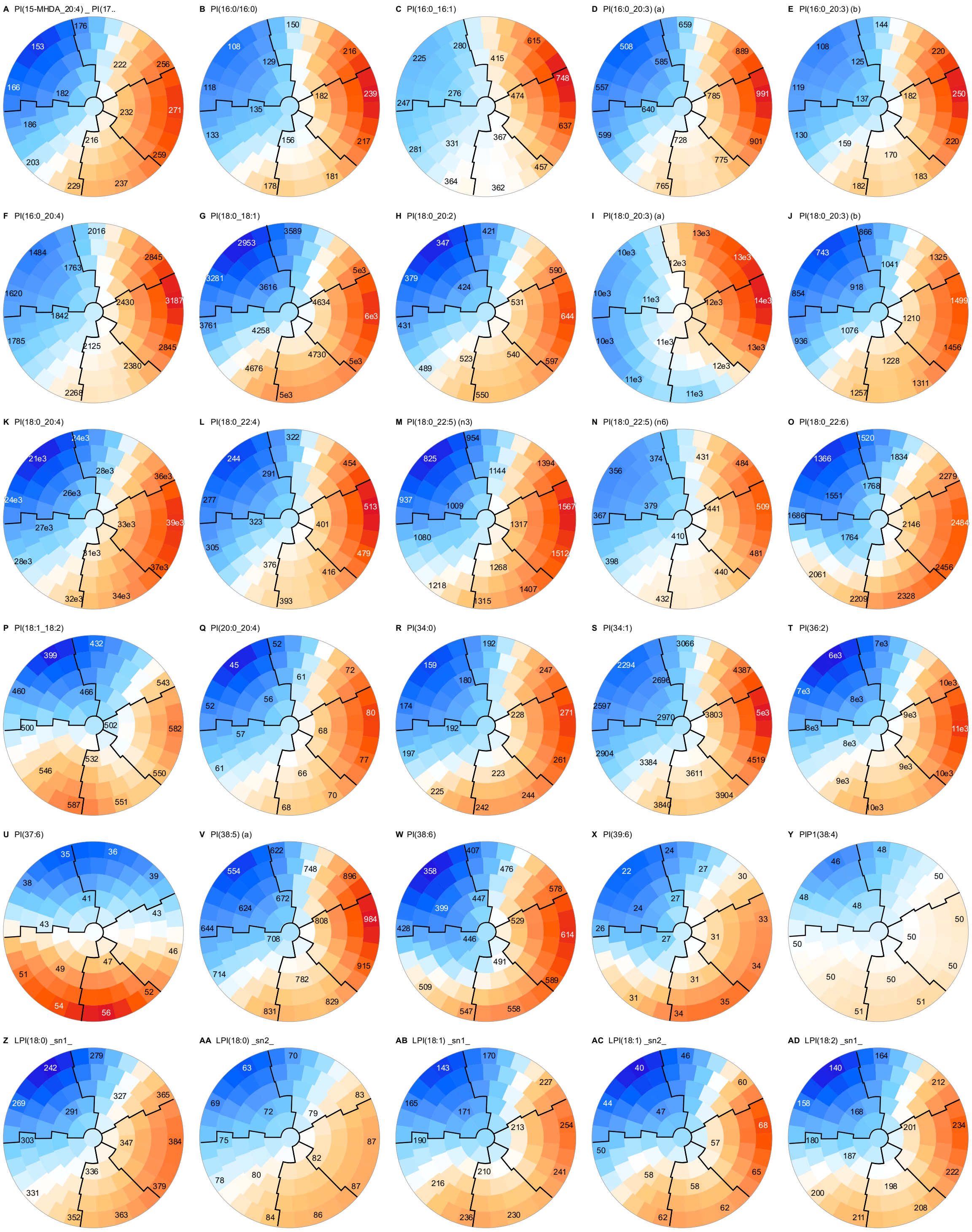
A PI(15-MHDA_20:4) _ PI(17..
B PI(16:0/16:0)
C PI(16:0_16:1)
D PI(16:0_20:3) (a)
E PI(16:0_20:3) (b)
F PI(16:0_20:4)
G PI(18:0_18:1)
H PI(18:0_20:2)
I PI(18:0_20:3) (a)
J PI(18:0_20:3) (b)
K PI(18:0_20:4)
L PI(18:0_22:4)
M PI(18:0_22:5) (n3)
N PI(18:0_22:5) (n6)
O PI(18:0_22:6)
P PI(18:1_18:2)
Q PI(20:0_20:4)
R PI(34:0)
S PI(34:1)
T PI(36:2)
U PI(37:6)
V PI(38:5) (a)
W PI(38:6)
X PI(39:6)
Y PIP1(38:4)
Z LPI(18:0) _sn1_
AA LPI(18:0) _sn2_
AB LPI(18:1) _sn1_
AC LPI(18:1) _sn2_
AD LPI(18:2) _sn1_

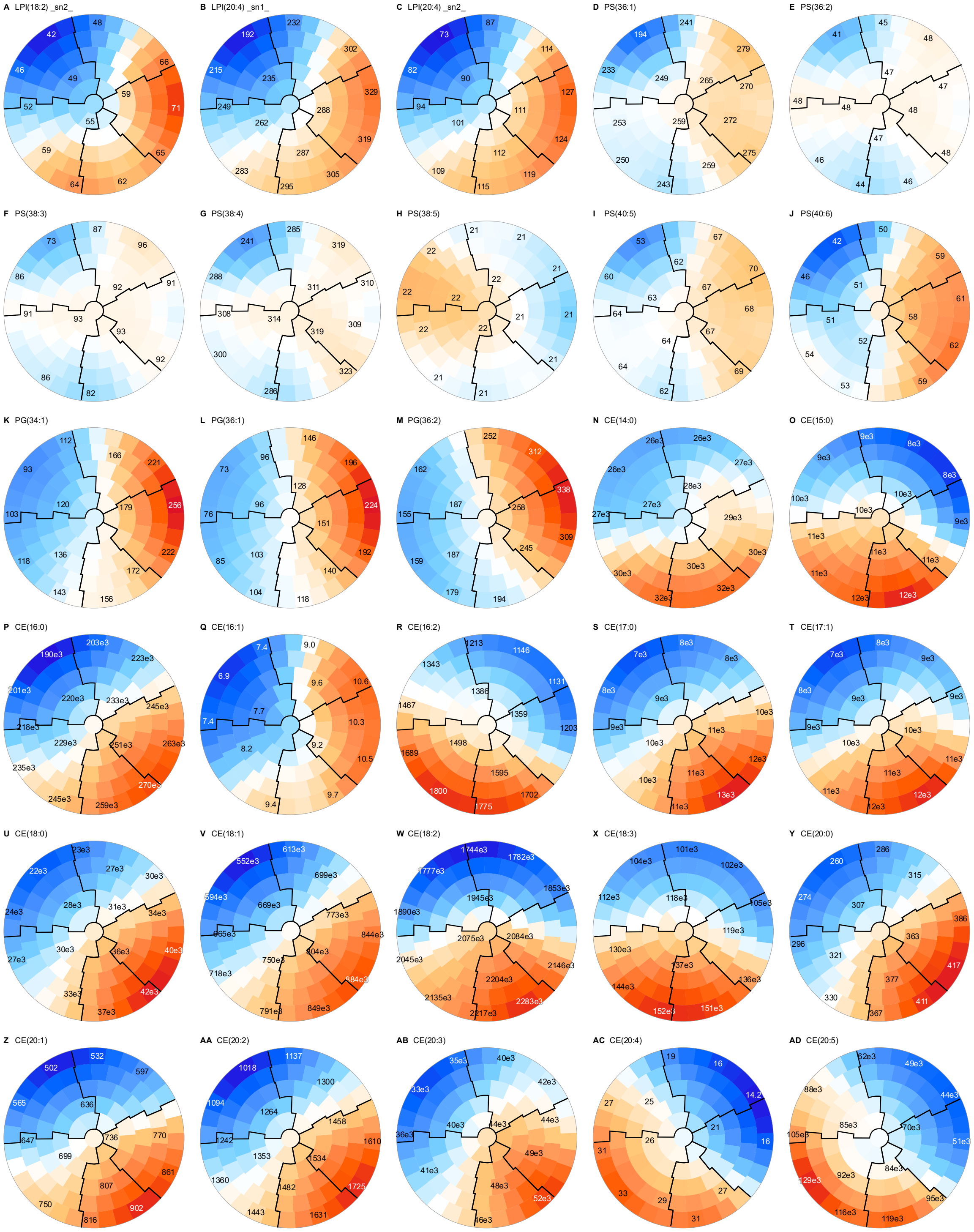
A LPI(18:2) _sn2_
B LPI(20:4) _sn1_
C LPI(20:4) _sn2_
D PS(36:1)
E PS(36:2)
F PS(38:3)
G PS(38:4)
H PS(38:5)
I PS(40:5)
J PS(40:6)
K PG(34:1)
L PG(36:1)
M PG(36:2)
N CE(14:0)
O CE(15:0)
P CE(16:0)
Q CE(16:1)
R CE(16:2)
S CE(17:0)
T CE(17:1)
U CE(18:0)
V CE(18:1)
W CE(18:2)
X CE(18:3)
Y CE(20:0)
Z CE(20:1)
AA CE(20:2)
AB CE(20:3)
AC CE(20:4)
AD CE(20:5)

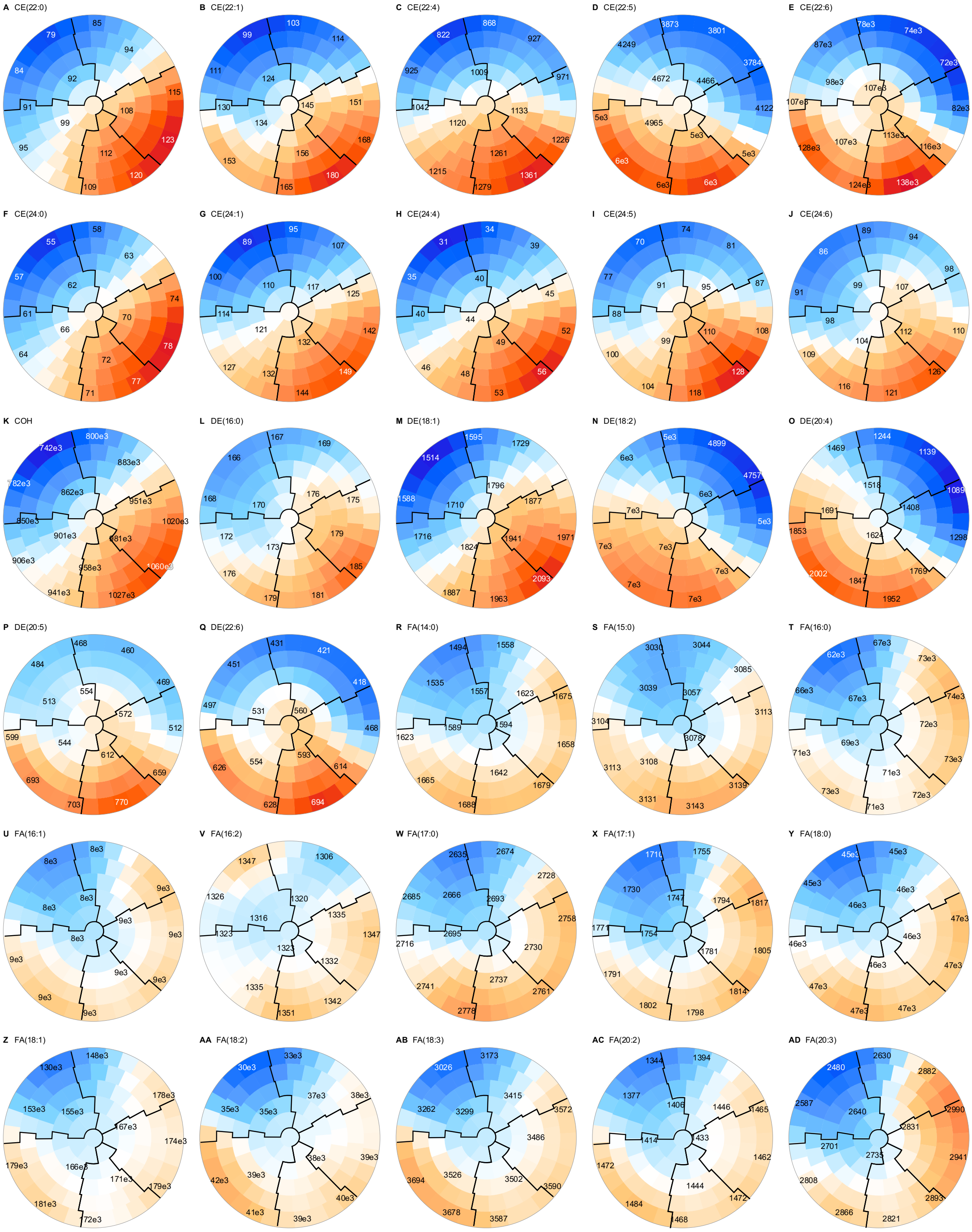
A CE(22:0)
85 79 94 84 92 115 91 108 99 123 95 112 120 109
B CE(22:1)
103 99 114 111 124 151 130 145 134 168 153 156 180 165
C CE(22:4)
868 822 927 925 1009 971 1042 1133 1120 1226 1215 1261 1361 1279
D CE(22:5)
3873 3801 4249 4672 4466 3784 4122 5e3 4965 5e3 5e3 6e3 6e3 6e3
E CE(22:6)
78e3 74e3 87e3 72e3 98e3 107e3 82e3 107e3 107e3 113e3 128e3 116e3 124e3 138e3
F CE(24:0)
58 55 63 57 62 74 61 70 66 78 64 72 77 71
G CE(24:1)
95 89 107 100 110 117 125 114 121 142 132 127 132 149 144
H CE(24:4)
34 31 39 35 40 45 40 44 52 49 46 48 56 53
I CE(24:5)
74 70 81 77 91 95 87 88 110 108 99 100 128 104 118
J CE(24:6)
89 94 86 98 99 107 91 98 112 110 104 109 126 116 121
K COH
800e3 742e3 883e3 782e3 862e3 951e3 850e3 1020e3 901e3 981e3 906e3 958e3 1060e3 941e3 1027e3
L DE(16:0)
167 169 166 176 175 168 170 172 179 173 185 176 179 181
M DE(18:1)
1595 1729 1514 1796 1877 1588 1710 1716 1824 1941 1971 2093 1887 1963
N DE(18:2)
5e3 4899 6e3 4757 6e3 7e3 5e3 7e3 7e3 7e3 7e3 7e3
O DE(20:4)
1244 1469 1139 1518 1089 1691 1408 1853 1624 1298 1769 2002 1847 1952
P DE(20:5)
468 460 484 469 554 513 572 512 599 544 612 659 693 703 770
Q DE(22:6)
431 421 451 418 497 531 560 468 554 593 614 626 628 694
R FA(14:0)
1494 1558 1535 1557 1623 1675 1589 1594 1623 1658 1642 1665 1679 1688
S FA(15:0)
3030 3044 3085 3039 3057 3113 3104 3078 3113 3108 3139 3131 3143
T FA(16:0)
67e3 62e3 73e3 66e3 67e3 74e3 72e3 69e3 71e3 73e3 71e3 73e3 72e3 71e3
U FA(16:1)
8e3 8e3 9e3 8e3 8e3 9e3 9e3 8e3 9e3 9e3 9e3 9e3 9e3
V FA(16:2)
1347 1306 1326 1320 1335 1316 1323 1347 1323 1332 1335 1342 1351
W FA(17:0)
2635 2674 2728 2685 2666 2693 2758 2695 2716 2730 2741 2737 2761 2778
X FA(17:1)
1710 1755 1730 1747 1794 1817 1771 1754 1805 1781 1791 1814 1802 1798
Y FA(18:0)
45e3 45e3 45e3 46e3 46e3 47e3 46e3 46e3 47e3 46e3 47e3 47e3 47e3
Z FA(18:1)
148e3 130e3 178e3 153e3 155e3 167e3 174e3 179e3 166e3 171e3 179e3 181e3 172e3
AA FA(18:2)
33e3 30e3 37e3 38e3 35e3 35e3 38e3 39e3 42e3 39e3 40e3 41e3 39e3
AB FA(18:3)
3173 3026 3415 3262 3299 3572 3486 3694 3526 3502 3590 3678 3587
AC FA(20:2)
1344 1394 1377 1406 1446 1465 1414 1433 1462 1472 1444 1484 1472 1468
AD FA(20:3)
2630 2480 2882 2587 2640 2990 2831 2701 2735 2941 2808 2866 2893 2821

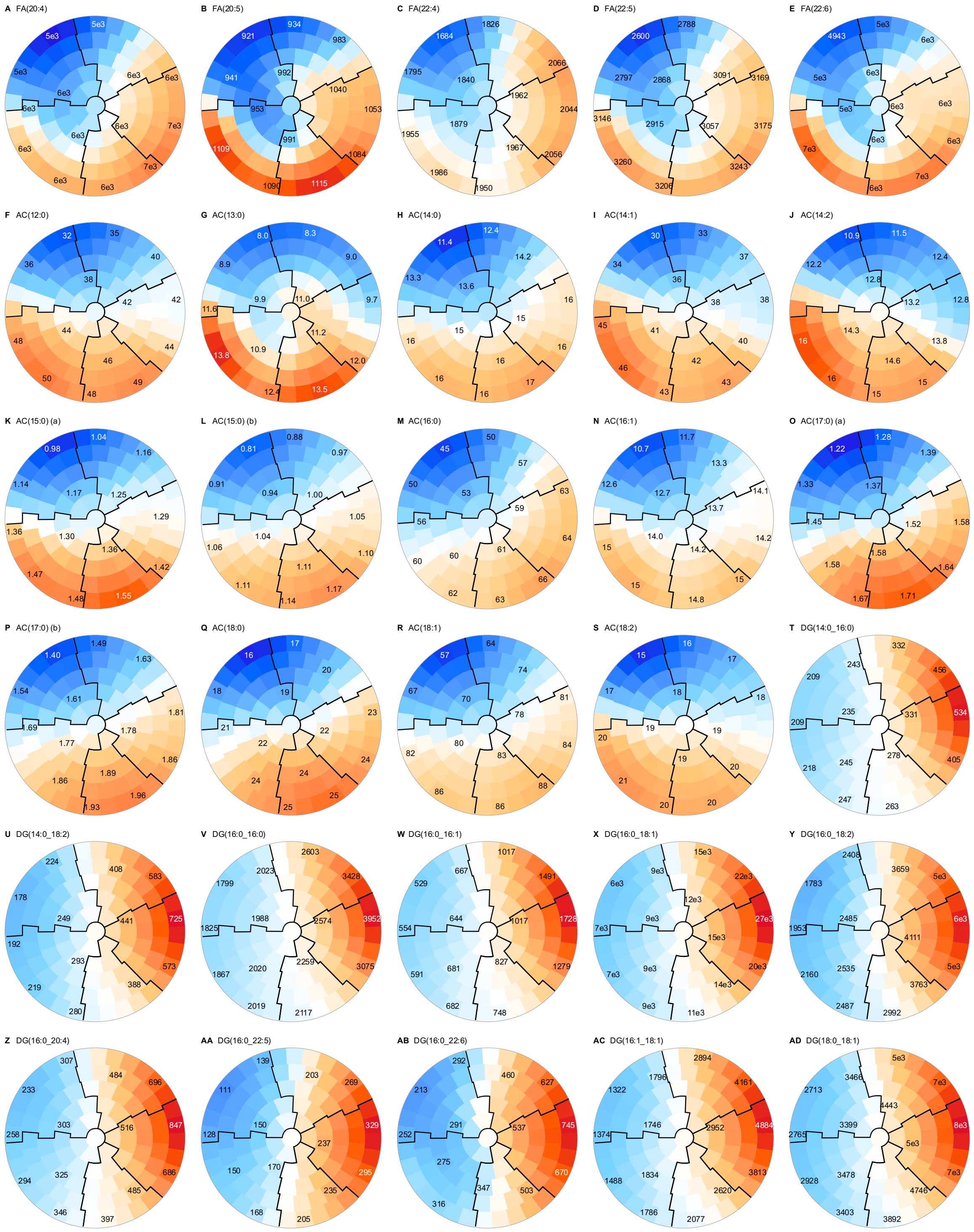

A FA(20:4)
5e3 5e3 5e3 6e3 6e3 6e3 6e3 6e3 7e3 6e3 6e3 7e3 6e3 6e3
B FA(20:5)
921 934 983 941 992 1040 953 1053 991 1109 1084 1090 1115
C FA(22:4)
1684 1826 1795 1840 2066 1962 2044 1879 1955 1967 2056 1986 1950
D FA(22:5)
2600 2788 2797 2868 3091 3169 3146 2915 3057 3175 3260 3243 3206
E FA(22:6)
4943 5e3 6e3 5e3 6e3 6e3 5e3 6e3 6e3 6e3 7e3 6e3 7e3
F AC(12:0)
32 35 36 40 38 42 42 44 48 46 44 50 49 48
G AC(13:0)
8.0 8.3 8.9 9.0 9.9 11.0 9.7 11.6 11.2 10.9 12.0 13.8 12.4 13.5
H AC(14:0)
11.4 12.4 14.2 13.3 13.6 16 15 15 16 16 16 16 17 16
I AC(14:1)
30 33 34 37 36 38 38 45 41 40 46 42 43 43
J AC(14:2)
10.9 11.5 12.2 12.4 12.8 13.2 12.8 16 14.3 13.8 14.6 16 15 15
K AC(15:0) (a)
0.98 1.04 1.16 1.14 1.17 1.25 1.29 1.36 1.30 1.36 1.42 1.47 1.48 1.55
L AC(15:0) (b)
0.81 0.88 0.97 0.91 0.94 1.00 1.05 1.06 1.04 1.10 1.11 1.11 1.17 1.14
M AC(16:0)
45 50 57 50 53 63 59 56 64 60 61 60 66 62 63
N AC(16:1)
10.7 11.7 13.3 12.6 12.7 14.1 13.7 14.0 14.2 15 14.2 15 15 14.8
O AC(17:0) (a)
1.22 1.28 1.39 1.33 1.37 1.45 1.52 1.58 1.58 1.58 1.64 1.67 1.71
P AC(17:0) (b)
1.40 1.49 1.63 1.54 1.61 1.69 1.78 1.81 1.77 1.86 1.86 1.89 1.96 1.93
Q AC(18:0)
16 17 20 18 19 21 22 23 22 24 24 24 25 25
R AC(18:1)
57 64 74 67 70 81 78 80 84 82 83 86 88 86
S AC(18:2)
15 16 17 17 18 18 19 19 20 19 20 21 20 20
T DG(14:0_16:0)
332 243 456 209 235 331 534 209 278 405 218 245 247 263
U DG(14:0_18:2)
224 408 583 178 249 441 725 192 293 573 219 388 280
V DG(16:0_16:0)
2603 2023 3428 1799 1988 2574 3952 1825 2259 3075 1867 2020 2019 2117
W DG(16:0_16:1)
1017 667 1491 529 644 1017 1728 554 827 1279 591 681 682 748
X DG(16:0_18:1)
15e3 9e3 22e3 6e3 12e3 9e3 27e3 7e3 15e3 20e3 7e3 9e3 14e3 9e3 11e3
Y DG(16:0_18:2)
2408 3659 5e3 1783 2485 6e3 1953 4111 5e3 2160 2535 3763 2487 2992
Z DG(16:0_20:4)
307 484 696 233 303 516 847 258 686 294 325 485 346 397
AA DG(16:0_22:5)
139 203 269 111 150 237 329 128 295 150 170 235 168 205
AB DG(16:0_22:6)
292 460 627 213 291 537 745 252 670 275 347 503 316
AC DG(16:1_18:1)
2894 1796 4161 1322 1746 2952 4884 1374 3813 1488 1834 2620 1786 2077
AD DG(18:0_18:1)
5e3 3466 7e3 2713 4443 3399 8e3 2765 5e3 7e3 2928 3478 4746 3403 3892

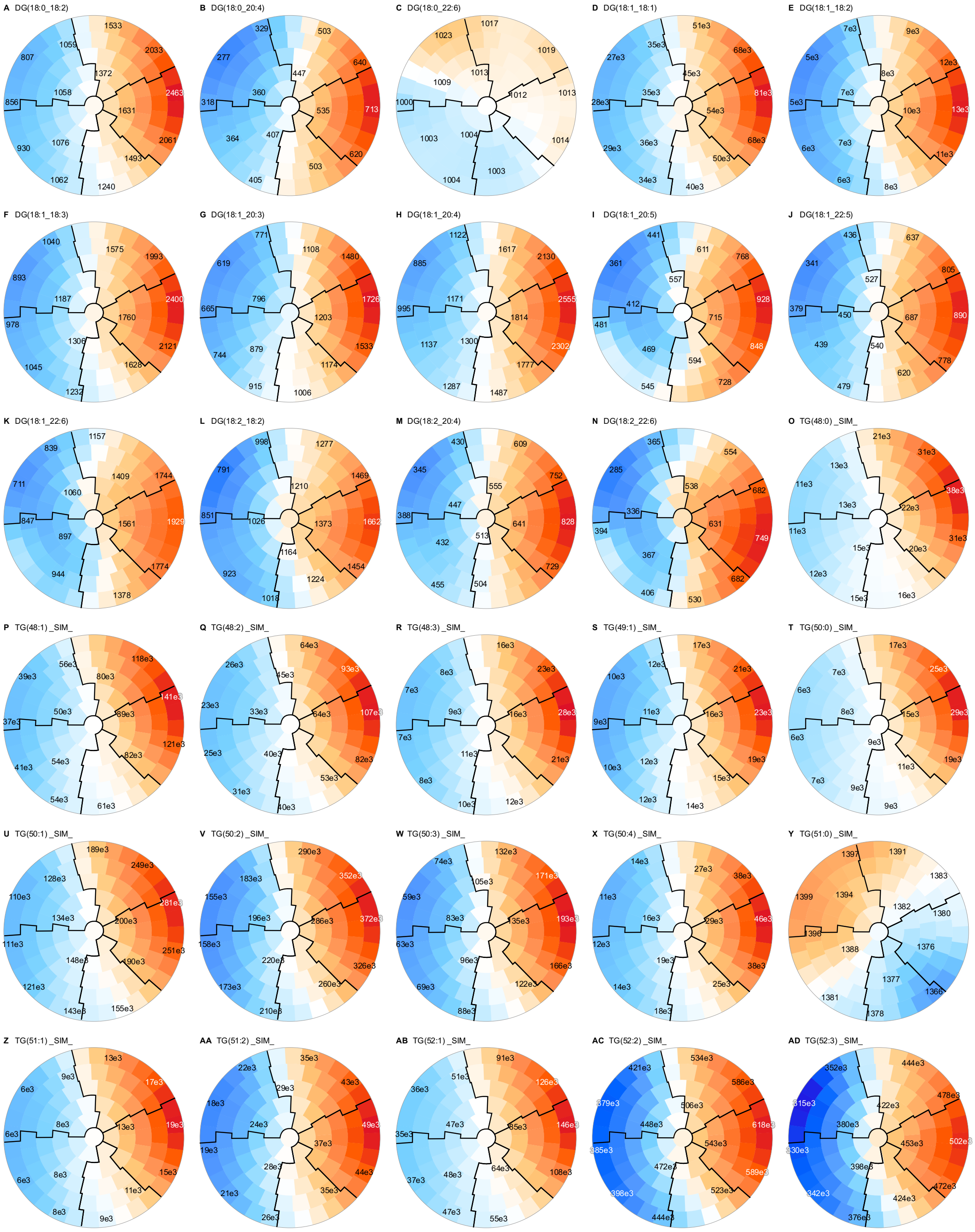
A DG(18:0_18:2)
B DG(18:0_20:4)
C DG(18:0_22:6)
D DG(18:1_18:1)
E DG(18:1_18:2)
F DG(18:1_18:3)
G DG(18:1_20:3)
H DG(18:1_20:4)
I DG(18:1_20:5)
J DG(18:1_22:5)
K DG(18:1_22:6)
L DG(18:2_18:2)
M DG(18:2_20:4)
N DG(18:2_22:6)
O TG(48:0) _SIM_
P TG(48:1) _SIM_
Q TG(48:2) _SIM_
R TG(48:3) _SIM_
S TG(49:1) _SIM_
T TG(50:0) _SIM_
U TG(50:1) _SIM_
V TG(50:2) _SIM_
W TG(50:3) _SIM_
X TG(50:4) _SIM_
Y TG(51:0) _SIM_
Z TG(51:1) _SIM_
AA TG(51:2) _SIM_
AB TG(52:1) _SIM_
AC TG(52:2) _SIM_
AD TG(52:3) _SIM_

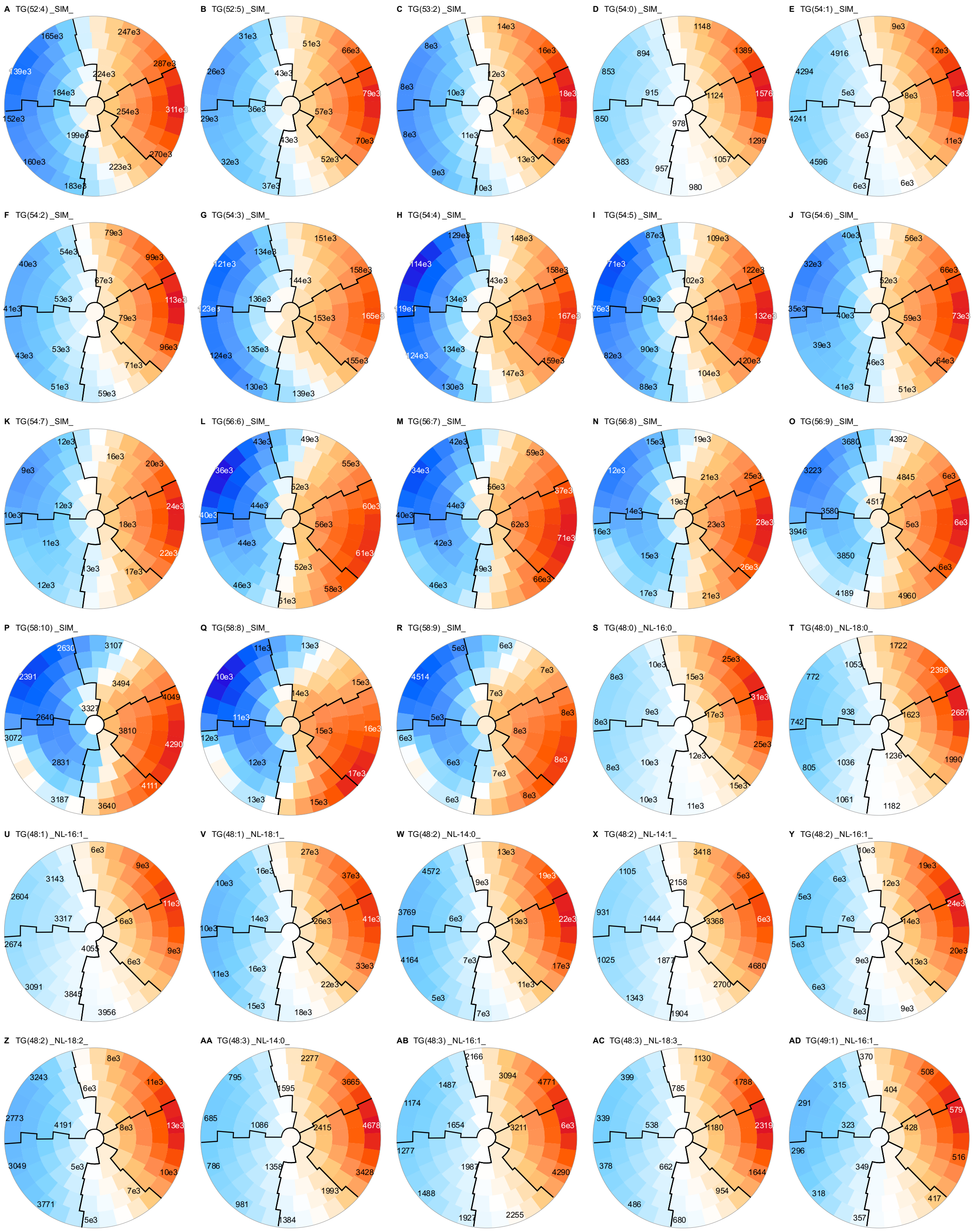

A TG(52:4) _SIM_
B TG(52:5) _SIM_
C TG(53:2) _SIM_
D TG(54:0) _SIM_
E TG(54:1) _SIM_
F TG(54:2) _SIM_
G TG(54:3) _SIM_
H TG(54:4) _SIM_
I TG(54:5) _SIM_
J TG(54:6) _SIM_
K TG(54:7) _SIM_
L TG(56:6) _SIM_
M TG(56:7) _SIM_
N TG(56:8) _SIM_
O TG(56:9) _SIM_
P TG(58:10) _SIM_
Q TG(58:8) _SIM_
R TG(58:9) _SIM_
S TG(48:0) _NL-16:0_
T TG(48:0) _NL-18:0_
U TG(48:1) _NL-16:1_
V TG(48:1) _NL-18:1_
W TG(48:2) _NL-14:0_
X TG(48:2) _NL-14:1_
Y TG(48:2) _NL-16:1_
Z TG(48:2) _NL-18:2_
AA TG(48:3) _NL-14:0_
AB TG(48:3) _NL-16:1_
AC TG(48:3) _NL-18:3_
AD TG(49:1) _NL-16:1_

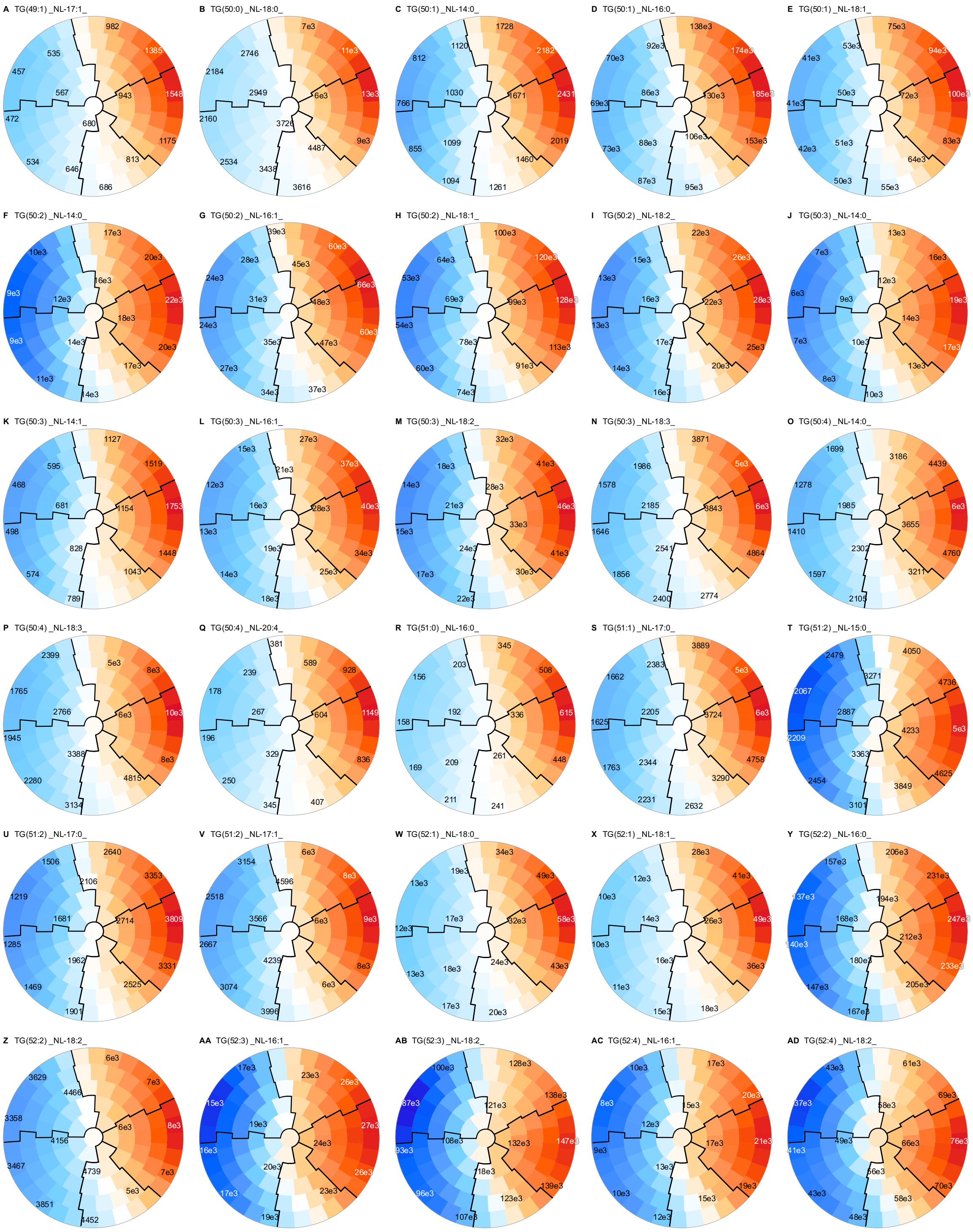
A TG(49:1) _NL-17:1_
B TG(50:0) _NL-18:0_
C TG(50:1) _NL-14:0_
D TG(50:1) _NL-16:0_
E TG(50:1) _NL-18:1_
F TG(50:2) _NL-14:0_
G TG(50:2) _NL-16:1_
H TG(50:2) _NL-18:1_
I TG(50:2) _NL-18:2_
J TG(50:3) _NL-14:0_
K TG(50:3) _NL-14:1_
L TG(50:3) _NL-16:1_
M TG(50:3) _NL-18:2_
N TG(50:3) _NL-18:3_
O TG(50:4) _NL-14:0_
P TG(50:4) _NL-18:3_
Q TG(50:4) _NL-20:4_
R TG(51:0) _NL-16:0_
S TG(51:1) _NL-17:0_
T TG(51:2) _NL-15:0_
U TG(51:2) _NL-17:0_
V TG(51:2) _NL-17:1_
W TG(52:1) _NL-18:0_
X TG(52:1) _NL-18:1_
Y TG(52:2) _NL-16:0_
Z TG(52:2) _NL-18:2_
AA TG(52:3) _NL-16:1_
AB TG(52:3) _NL-18:2_
AC TG(52:4) _NL-16:1_
AD TG(52:4) _NL-18:2_

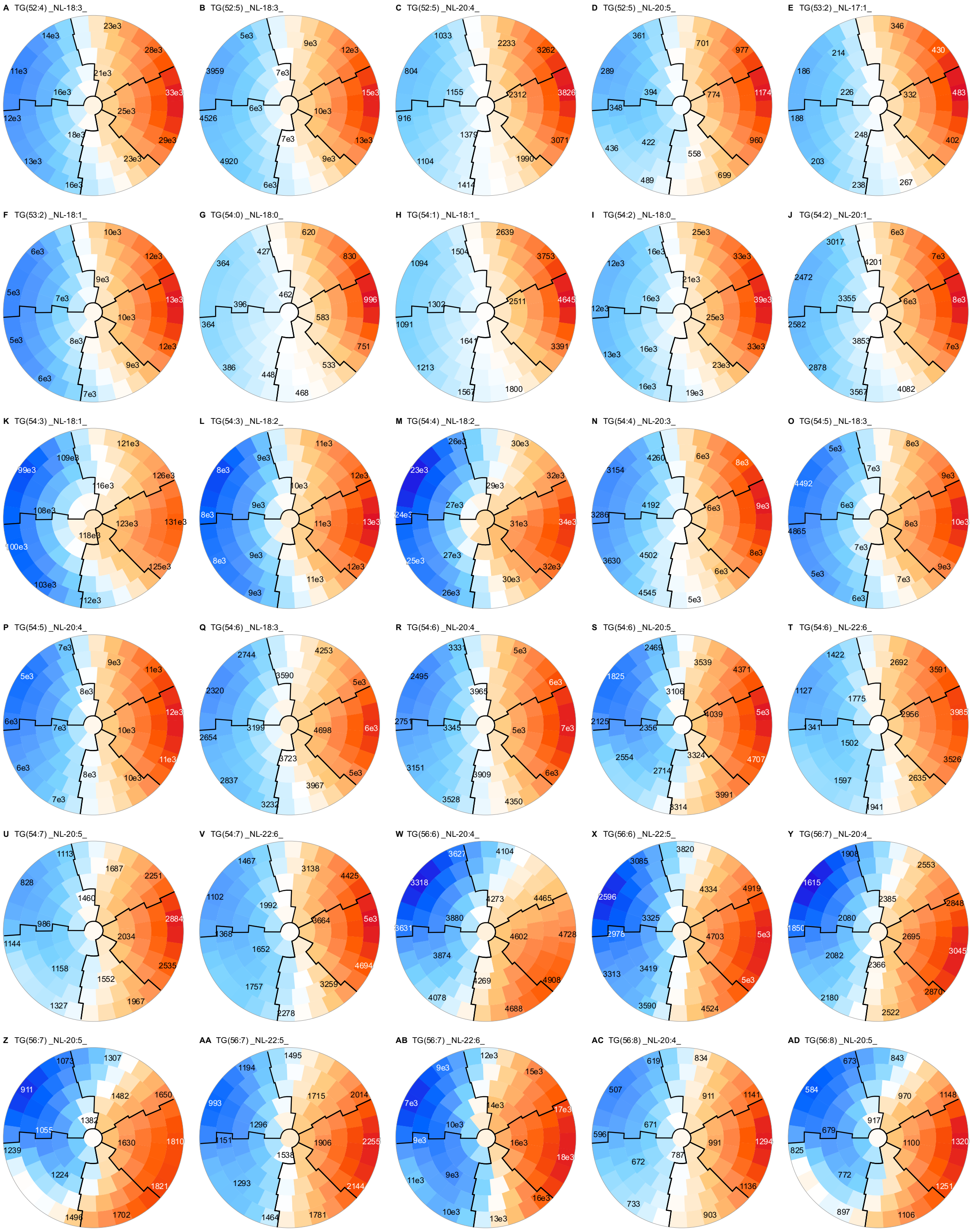
A TG(52:4) _NL-18:3_
B TG(52:5) _NL-18:3_
C TG(52:5) _NL-20:4_
D TG(52:5) _NL-20:5_
E TG(53:2) _NL-17:1_
F TG(53:2) _NL-18:1_
G TG(54:0) _NL-18:0_
H TG(54:1) _NL-18:1_
I TG(54:2) _NL-18:0_
J TG(54:2) _NL-20:1_
K TG(54:3) _NL-18:1_
L TG(54:3) _NL-18:2_
M TG(54:4) _NL-18:2_
N TG(54:4) _NL-20:3_
O TG(54:5) _NL-18:3_
P TG(54:5) _NL-20:4_
Q TG(54:6) _NL-18:3_
R TG(54:6) _NL-20:4_
S TG(54:6) _NL-20:5_
T TG(54:6) _NL-22:6_
U TG(54:7) _NL-20:5_
V TG(54:7) _NL-22:6_
W TG(56:6) _NL-20:4_
X TG(56:6) _NL-22:5_
Y TG(56:7) _NL-20:4_
Z TG(56:7) _NL-20:5_
AA TG(56:7) _NL-22:5_
AB TG(56:7) _NL-22:6_
AC TG(56:8) _NL-20:4_
AD TG(56:8) _NL-20:5_

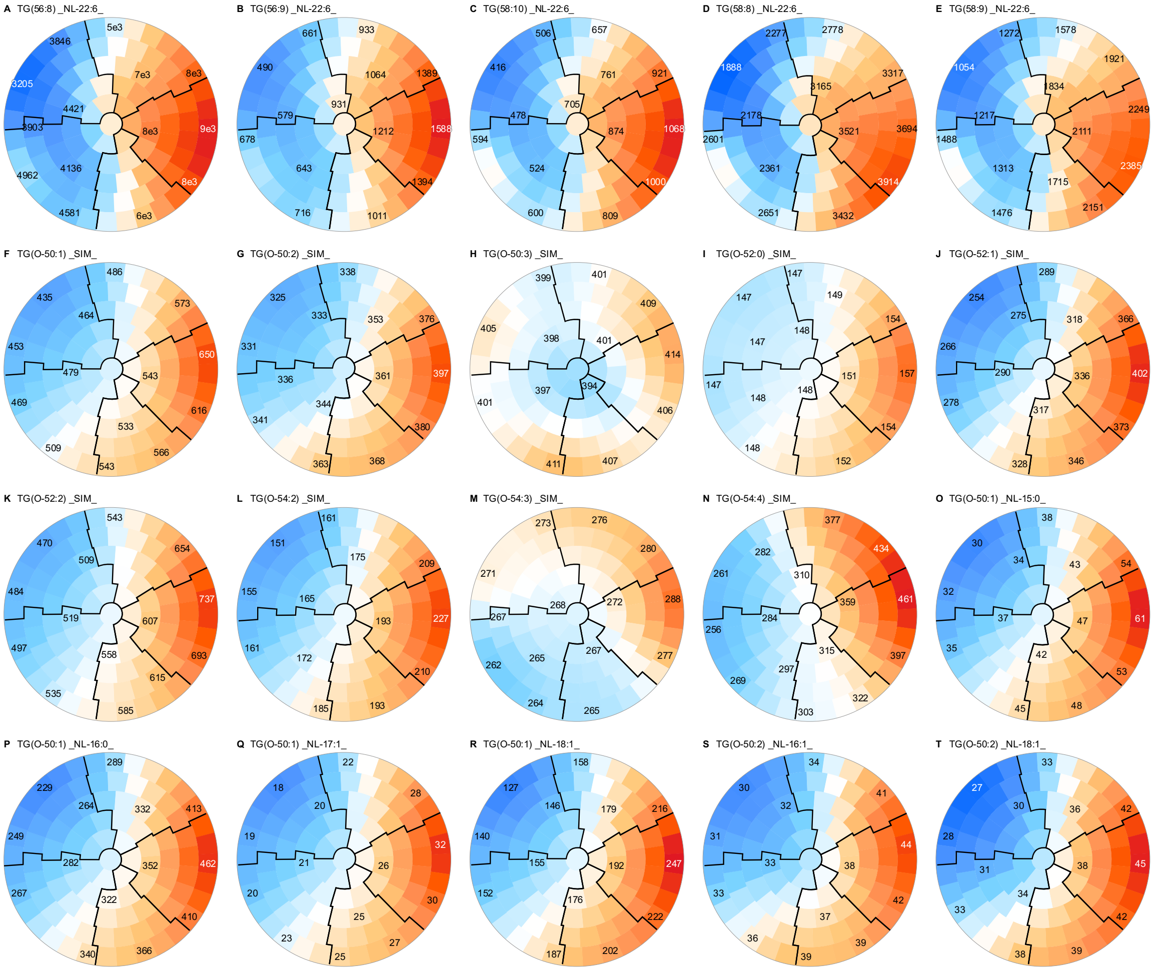
A TG(56:8) _NL-22:6_
B TG(56:9) _NL-22:6_
C TG(58:10) _NL-22:6_
D TG(58:8) _NL-22:6_
E TG(58:9) _NL-22:6_
F TG(O-50:1) _SIM_
G TG(O-50:2) _SIM_
H TG(O-50:3) _SIM_
I TG(O-52:0) _SIM_
J TG(O-52:1) _SIM_
K TG(O-52:2) _SIM_
L TG(O-54:2) _SIM_
M TG(O-54:3) _SIM_
N TG(O-54:4) _SIM_
O TG(O-50:1) _NL-15:0_
P TG(O-50:1) _NL-16:0_
Q TG(O-50:1) _NL-17:1_
R TG(O-50:1) _NL-18:1_
S TG(O-50:2) _NL-16:1_
T TG(O-50:2) _NL-18:1_

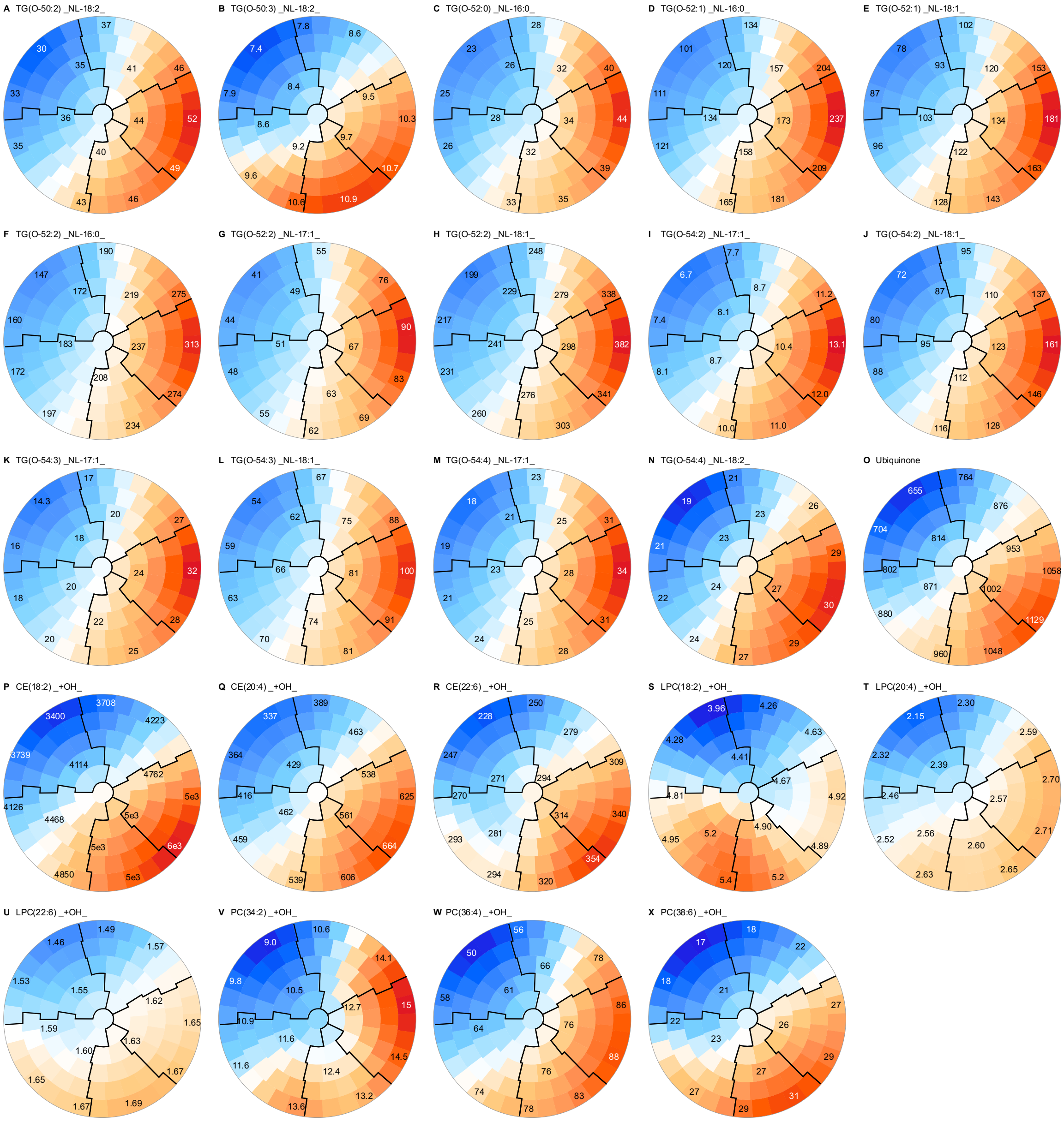
A TG(O-50:2) _NL-18:2_
37 30 35 41 46 33 36 44 52 35 40 49 43 46
B TG(O-50:3) _NL-18:2_
7.8 8.6 7.4 8.4 9.5 7.9 8.6 10.3 9.2 9.7 9.6 10.7 10.6 10.9
C TG(O-52:0) _NL-16:0_
28 23 26 32 40 25 28 34 44 26 32 39 33 35
D TG(O-52:1) _NL-16:0_
134 101 120 157 204 111 134 173 237 121 158 209 165 181
E TG(O-52:1) _NL-18:1_
102 78 93 120 153 87 103 134 181 96 122 163 128 143
F TG(O-52:2) _NL-16:0_
190 147 172 219 275 160 183 237 313 172 208 274 197 234
G TG(O-52:2) _NL-17:1_
55 41 49 76 44 90 51 67 48 83 63 55 69 62
H TG(O-52:2) _NL-18:1_
248 199 229 279 338 217 241 298 382 231 276 341 260 303
I TG(O-54:2) _NL-17:1_
7.7 6.7 8.7 11.2 8.1 7.4 10.4 13.1 8.7 8.1 12.0 10.0 11.0
J TG(O-54:2) _NL-18:1_
95 72 87 110 137 80 95 123 161 88 112 146 116 128
K TG(O-54:3) _NL-17:1_
17 14.3 20 27 18 16 24 32 20 18 22 28 20 25
L TG(O-54:3) _NL-18:1_
67 54 62 75 88 59 66 81 100 63 74 91 70 81
M TG(O-54:4) _NL-17:1_
23 18 21 25 31 19 23 28 34 21 25 31 24 28
N TG(O-54:4) _NL-18:2_
21 19 23 26 23 21 29 24 27 22 30 24 29 27
O Ubiquinone
764 655 876 704 814 953 802 1058 871 1002 880 1129 960 1048
P CE(18:2) _+OH_
3708 3400 4223 3739 4114 4762 5e3 4126 5e3 4468 5e3 6e3 4850 5e3
Q CE(20:4) _+OH_
389 337 463 364 429 538 625 416 462 561 459 664 539 606
R CE(22:6) _+OH_
250 228 279 247 309 271 294 270 340 314 281 293 354 294 320
S LPC(18:2) _+OH_
3.96 4.26 4.63 4.28 4.41 4.67 4.92 4.81 4.90 4.95 5.2 4.89 5.4 5.2
T LPC(20:4) _+OH_
2.30 2.15 2.59 2.32 2.39 2.70 2.46 2.57 2.71 2.52 2.56 2.60 2.63 2.65
U LPC(22:6) _+OH_
1.49 1.46 1.57 1.53 1.55 1.62 1.65 1.59 1.63 1.60 1.67 1.65 1.67 1.69
V PC(34:2) _+OH_
10.6 9.0 14.1 9.8 10.5 12.7 15 10.9 11.6 14.5 11.6 12.4 13.2 13.6
W PC(36:4) _+OH_
56 50 78 66 86 58 61 76 64 88 76 74 83 78
X PC(38:6) _+OH_
18 17 22 18 21 27 22 26 23 29 27 27 31 29
