# Supplementary material for: Correction to “Personalized Profiling of Lipoprotein and Lipid Metabolism Based on 1018 Measures from Combined Quantitative NMR and LC-MS/MS Platforms”
Source: Anal Chem. 2025 Apr 1;97(14):8130. doi: 10.1021/acs.analchem.5c01621 (PMC12004337; doi:10.1021/acs.analchem.5c01621)
Supplement: Supplementary file 1 — ac5c01621_si_001.pdf [file ac5c01621_si_001.pdf]

Supporting Information

Personalized Profiling of Lipoprotein and Lipid Metabolism Based on 1018 Measures from Combined Quantitative NMR and LC-MS/MS Platforms

Siyu Zhao,[1,2,3] Corey Giles,[4,5] Kevin Huynh,[4,5] Johannes Kettunen,[1,2,3,6] Marjo-Riitta Järvelin,[2,7,8] Mika Kähönen,[9] Jorma Viikari,[10,11] Terho Lehtimäki,[12] Olli T. Raitakari,[13,14,15,16] Peter J. Meikle,[4,5,17] Ville-Petteri Mäkinen,[1,2,3] and Mika Ala-Korpela[1,2,3,17,18,*]

[1]Systems Epidemiology, Faculty of Medicine, University of Oulu, 90014 Oulu, Finland; [2]Research Unit of Population Health, Faculty of Medicine, University of Oulu, 90014 Oulu, Finland; [3]Biocenter Oulu, 90014 Oulu, Finland; [4]Baker Heart and Diabetes Institute, Melbourne 3004, Australia; [5]Baker Department of Cardiometabolic Health, University of Melbourne, Melbourne 3004, Australia; [6]Department of Public Health and Welfare, Finnish Institute for Health and Welfare, 00271 Helsinki, Finland; [7]Department of Epidemiology and Biostatistics, MRC Centre for Environment and Health, School of Public Health, Imperial College London, London W12 0BZ, UK; [8]Department of Life Sciences, College of Health and Life Sciences, Brunel University London, UB8 3PH, UK; [9]Department of Clinical Physiology, Tampere University Hospital, and Finnish Cardiovascular Research Center Tampere, Faculty of Medicine and Health Technology, Tampere University, 33270 Tampere, Finland; [10]Department of Medicine, University of Turku, 20014 Turku, Finland; [11]Division of Medicine, Turku University Hospital, 20014 Turku, Finland; [12]Department of Clinical Chemistry, Fimlab Laboratories, and Finnish Cardiovascular Research Center Tampere, Faculty of Medicine and Health Technology, Tampere University, 33270Tampere, Finland; [13]Research Centre of Applied and Preventive Cardiovascular Medicine, University of Turku, 20014 Turku, Finland; [14]Centre for Population Health Research, University of Turku and Turku University Hospital, 20014 Turku, Finland; [15]Department of Clinical Physiology and Nuclear Medicine, Turku University Hospital, 20014 Turku, Finland; [16]InFLAMES Research Flagship, University of Turku, 20014 Turku, Finland; [17]Monash University, Melbourne 3004, Australia; [18]NMR Metabolomics Laboratory, School of Pharmacy, University of Eastern Finland, 70210 Kuopio, Finland.

Table of Content

Supplement Note

Study Populations **S4**

LC-MS/MS Mass Spectrometry Analysis for lipidomics **S4**

Self-Organizing Maps **S5**

Characteristics of Integrated Data **S6**

Results and Discussion **S6**

References **S8**

Funding **S9**

Abbreviations **S10**

Supplement Tables

Table S1. Clinical characteristics in NFBC66 and YFS. **S13**

Table S2. NMR-based lipoprotein measures in NFBC66 and YFS. **S14**

Table S3. LC-MS/MS lipidomics measures for the total concentrations of the circulating lipid classes in NFBC66 and YFS. **S21**

Table S4. (Excel) LC-MS/MS lipidomics measures for the concentrations of the circulating individual lipid species in NFBC66 and YFS.

Table S5. Comparison of the relative abundances (mol-%) of the most common serum lipid classes in lipidomics studies. **S23**

Table S6. (Excel) A. Spearman's rank correlations (adjusted for sex) for the 209 lipoprotein and 809 lipidomics measures in NFBC66. **B**. Spearman's rank correlations (adjusted for sex) for the 209 lipoprotein and 809 lipidomics measures in YFS. **C.** Meta-analyzed Spearman's rank correlations (adjusted for sex) for the 209 lipoprotein and 809 lipidomics measures in NFBC66 and YFS.

Supplement Figures

Figure S1. Individual variation in the relative abundances of the four most abundant serum lipid classes in NFBC66 and YFS based on the LC-MS/MS data. **S24**

Figure S2. Associations between the concentrations of 25 individual DG species, 34 individual TG[SIM], and 79 TG[NL] species (LC-MS/MS) and the particle and lipid concentrations of 14 lipoprotein subclasses (NMR) as indicated by partial Spearman's rank correlations. **S25**

Figure S3. Associations between the concentrations of 3 individual Sph, 4 individual S1P, 6 individual dhCer, 50 individual Cer(d), 11individual Cer(m), and one C1P species (LC-MS/MS) and the particle and lipid concentrations of 14 lipoprotein subclasses (NMR) as indicated by partial Spearman's rank correlations. **S26**

Figure S4. Associations between the concentrations of 14 individual HexCer, 10 Hex2Cer, 6 Hex3Cer, 6 individual GM3, one GM1, 6 ShexCer, and 44 individual SM species (LC-MS/MS) and the particle and lipid concentrations of 14 lipoprotein subclasses (NMR) as indicated by partial Spearman's rank correlations. **S27**

Figure S5. Associations between the concentrations of 6 individual PA species and 68 individual PC species (LC-MS/MS) and the particle and lipid concentrations of 14 lipoprotein subclasses (NMR) as indicated by partial Spearman's rank correlations. **S28**

Figure S6. Associations between the concentrations of 61 individual LPC and 37 individual PE species (LC-MS/MS) and the particle and lipid concentrations of 14 lipoprotein subclasses (NMR) as indicated by partial Spearman's rank correlations. **S29**

Figure S7. Associations between the concentrations of 22 individual PC(O), 26 individual PC(P), 10 individual LPC(O), 6 individual LPC(P), 14 individual PE(O), 14 LPE, and 4 LPE(P) species (LC-MS/MS) and the particle and lipid concentrations of 14 lipoprotein subclasses (NMR) as indicated by partial Spearman's rank correlations. **S30**

Figure S8. Associations between the concentrations of 54 individual PE(P) species (LC-MS/MS) and the particle and lipid concentrations of 14 lipoprotein subclasses (NMR) as indicated by partial Spearman's rank correlations. **S31**

Figure S9. Associations between the concentrations of 38 individual PI, one PIP1, 8 individual LPI, 7 PS, and 3 PG species (LC-MS/MS) and the particle and lipid concentrations of 14 lipoprotein subclasses (NMR) as indicated by partial Spearman's rank correlations. **S32**

Figure S10. Associations between the concentrations of 27 individual CE, one Free C, 6 individual DE, 18 individual FFA, and 14 AC species (LC-MS/MS) and the particle and lipid concentrations of 14 lipoprotein subclasses (NMR) as indicated by partial Spearman's rank correlations. **S33**

Figure S11. Associations between the concentrations of 9 individual TG(O)[SIM], 20 TG(O)[NL], one individual ubiquinone, 3 oxidized CE, and 6 oxidized PC/LPC species (LC-MS/MS) and the particle and lipid concentrations of 14 lipoprotein subclasses (NMR) as indicated by partial Spearman's rank correlations. **S34**

Figure S12. Colored SOM component planes for all the 1,018 molecular inputs. **S35**

Supplement Note

Study Populations

Northern Finland Birth Cohorts 1966

The Northern Finland Birth Cohort (NFBC) studies are two longitudinal birth cohorts established to study factors affecting preterm birth and consequent morbidity in the two northernmost provinces of Finland, Oulu and Lapland. The NFBC66 includes 12,058 live births (12,231 children) covering 96% of all eligible births in this region during January – December 1966. Data collection conducted in 2012 at their age of 46 years, including clinical examination and questionnaires.[1] The serum samples (n=5,657) in this study used for NMR spectroscopy and MS analysis were taken after overnight fasting. The research protocols were approved by the Ethics Committee of University of Oulu and the Ethics Committee of Northern Ostrobotnia Hospital District, Finland. More information on the cohort and the data collection can be found at http://www.oulu.fi/nfbc.

Cardiovascular Risk in Young Finns Study

The Cardiovascular Risk in Young Finns Study (YFS) is a population based prospective cohort study conducted with the aim of studying the levels of cardiovascular risk factors in children and adolescents in different parts of the country.[2] The first cross-sectional survey was conducted in 1980. Total sample size was 4,320 boys and girls in 6 age cohorts (aged 3, 6, 9, 12, 15 and 18), these subjects were randomly chosen from the national register. After that, several follow-up studies of this cohort have been conducted. A total of 7,349 participants attended these three generational field studies. Examinations have included comprehensive data collection using questionnaires, physical measurements, and blood tests. The follow-up during 2007 used in this study comprise 2,173 subjects. All serum samples were taken after overnight fasting. All participants gave written informed consent, and the study was approved by the local ethics committees and conducted in accordance with the Declaration of Helsinki. More information on the cohort can be found at https://youngfinnsstudy.utu.fi/index.html.

LC-MS/MS Mass Spectrometry Analysis for lipidomics

The lipid analyses were performed using triple quadruple mass spectrometer (Agilent 6490 QQQ) with an Agilent 1290 series HPLC system and a ZORBAX eclipse plus C18 column (2.1 × 100 mm × 1.8 µm). Solvent A consisted of 50% H2O/ 30% acetonitrile / 20% isopropanol with 10mM ammonium formate and 5µM medronic acid, while solvent B consisted of 1% H2O/ 9% acetonitrile / 90% isopropanol with 10mM ammonium formate. Mass spectrometry analysis was performed in a positive ion mode with dynamic

scheduled multiple reaction monitoring (MRM). We modified the methodology to include a dual column setup (in which one of the columns is set to equilibrate while the other is running a sample). The temperature within the column compartment was set to 45 °C with the chromatographic conditions as follows: starting at 15% solvent B and increasing to 50% B over 2.5 min, then quickly ramping to 57% B for 0.1 min. For 6.4 min, %B was increased to 70%, then increased to 93% over 0.1 min and increased to 96% over 1.9 min. The gradient was quickly ramped up to 100% B for 0.1 min and held at 100% B for a further 0.9 min. This was a total run time of 12 min. The column was then brought back down to 15% B for 0.2 min and held for another 0.7 min prior to switching to the alternate column for running the next sample. The column that was being equilibrated was run as follows: 0.9 min of 15% B, 0.1 min increase to 100% B and held for 5 min, decreasing back to 15% B over 0.1 min and held until it was switched for the next sample. We used a 1 µL injection per sample and the following mass spectrometer conditions were used: gas temperature, 150 °C; gas flow rate, 17 L/min; nebulizer, 20 psi; sheath gas temperature, 200 °C; capillary voltage, 3500 V and sheath gas flow, 10 L/min.

The Baker lipidomics platform has been under development for over 15 years. It represents a state of the art, high-throughput lipidomic methodology using reverse-phase liquid chromatography coupled tandem mass spectrometry. Lipid species are measured using polarity switching and dynamic multiple reaction monitoring. Detailed information on the characterization of lipid species is available in Huynh et al.[3] A detailed description of the methodology is highlighted in the Baker Institute-Agilent Application Note.[4] Extensive details of each lipid species, including chromatograms and integration bounds are available in our web portal.[5] We have provided transition, retention times, and collision energy for all measured species and internal standards.

Self-Organizing Maps

All individuals from both cohorts were analyzed together. Inputs were rank transformed to remove scale differences and to mitigate outliers. This was done separately for the cohorts to also remove potential cohort-specific biases. The impact of each lipid variable on the structure of the map was calculated using permutation analysis. Visually, the statistical impact is represented via the intensity of the map colors (Figure 3). Here various key measures from both data domains were considered when assessing the subgroup boundaries. These included, for example, apoB, TG, and lipoprotein subclass particle concentrations from NMR as well as, for example, concentration data for various lipid classes and some individual CE species from LC-MS/MS.

Characteristics of Integrated Data

Firstly, the lipoprotein analysis with NMR is performed directly from the intact serum sample without any sample pretreatments. The LC-MS/MS analysis, however, calls for a lipid extraction procedure, i.e., all the individual lipid molecules in the serum sample need to be isolated into a homogeneous lipid-soluble mixture (Figure 1). Along this process all the lipoprotein-related heterogeneity and information in the original sample is lost and thereby the LC-MS/MS lipid measures (both classes and species) represent total concentrations in all the circulating lipoprotein particles (Figure 2).

Secondly, almost the entire molecular transport in the bloodstream is regulated via a lipoprotein-centered metabolic cascade in which a complicated interplay with various enzymes, lipid transfer proteins and cell surface receptors result in, e.g., the delivery of TG in the VLDL particles, synthesized in the liver, to the adipose tissue. The resultant particles are further modified to LDL particles that, in unfavorable circumstances, can accumulate in the intimal spaces of arteries and cause atherosclerosis. The HDL particles – that do not contain apoB – interact with and are important players in the continuous conversions of the apoB particles. They also have other biological roles, for example, in relation to inflammation.

The path from the site of synthesis to the site of delivery or action for the individual lipid molecules can be extremely tangled with lipoprotein particles. Only a few lipid classes are transported by albumin, e.g., free fatty acids, acylcarnitines, and a portion of lipid species with only one fatty acid chain.[6,7] Thus, almost every metabolic pathway related to lipids in the systemic circulation is mediated by lipoprotein particles. This accentuates the importance of comprehensive lipoprotein data for proper analyses and interpretations of lipidomics data, accomplished here for the first time.

Results and Discussion

Associations between the Lipoprotein Subclasses and Lipid Classes

The entire lipoprotein cascade, as well as the individual particles, are metabolically and biophysically constrained with strong innate correlations.[8] The entirety of systemic lipoprotein metabolism can thus have wide-ranging effects even for correlations between a single lipoprotein subclass and a single lipidomics measure. This makes specific molecular interpretations challenging.

Associations between the Lipoprotein Subclasses and Lipid Species

This kind of opposite behaviour with various CE clusters bears resemblance to total cholesterol. It sums up all the circulating cholesterol with two main components, LDL-C and HDL-C, inherently representing opposing metabolic roles. This has led to the routine use of both LDL-C and HDL-C as key parts of the clinical lipid measurements.[9,10]

Self-Organizing Maps and Population Stratification

We have demonstrated previously that the SOM analysis is a prime method in solving complicated biomedical challenges, for example, in assessing risk for type 1 diabetes complications based on clinical and biochemical data[11] and in conformational analyses of molecular dynamic simulations in relation to the structural features of biological membranes.[12] The key distinction between the SOM analysis and many common unsupervised chemometric techniques, e.g., principal component analysis, is the means to handle multi-dimensional inputs and transform them to a 2-dimensional (human interpretable) map that is fully and directly connected with the unaltered input data (Figure 3).[13] In SOM analyses neither vast numbers of inputs nor individuals pose particular obstacles to the statistical algorithm.

In Figure 6 red color means higher and blue color lower values in relative to the population mean in each map district. The subgroup boundaries are based on the key characteristics of both data domains, including multiple lipoprotein-related measures (NMR) together with some of the lipid classes and species (LC-MS/MS). They should not be taken too strictly but to provide a guide to the human eye to understand and interpret the overall metabolic population characteristics. This is the strength of the SOM analysis.[13]

The compositional information on the lipoprotein subclass particles (L, M, and N) reveals that high circulating triglycerides (A) are associated with TG-enrichment of all circulating lipoprotein particles, including the HDL particles. In fact, compositional TG-enrichment of HDL particles has been indicated to result in higher risk of coronary heart disease, independently of total cholesterol and triglycerides.[14] This is an intriguing finding, also supported by the current results, linking the molecular details of lipoprotein particles to the risk of atherosclerosis. Nonetheless, it is our supposition that the TG-enrichment of the lipoprotein particles is not the primary metabolic cause of the disease risk, but the increased concentrations of circulating total triglycerides.

References

(1) Nordström, T.; Miettunen, J.; Auvinen, J.; Ala-Mursula, L.; Keinänen-Kiukaanniemi, S.; Veijola, J.; Järvelin, M.-R.; Sebert, S.; Männikkö, M. Cohort Profile: 46 Years of Follow-up of the Northern Finland Birth Cohort 1966 (NFBC1966). *Int. J. Epidemiol.* **2022**, *50*, 1786–1787j.
(2) Raitakari, O. T.; Juonala, M.; Rönnemaa, T.; Keltikangas-Järvinen, L.; Räsänen, L.; Pietikäinen, M.; Hutri-Kähönen, N.; Taittonen, L.; Jokinen, E.; Marniemi, J.; Jula, A.; Telama, R.; Kähönen, M.; Lehtimäki, T.; Akerblom, H. K.; Viikari, J. S. A. Cohort Profile: The Cardiovascular Risk in Young Finns Study. *Int. J. Epidemiol.* **2008**, *37*, 1220–1226.
(3) Huynh, K.; Barlow, C. K.; Jayawardana, K. S.; Weir, J. M.; Mellett, N. A.; Cinel, M.; Magliano, D. J.; Shaw, J. E.; Drew, B. G.; Meikle, P. J. High-Throughput Plasma Lipidomics: Detailed Mapping of the Associations with Cardiometabolic Risk Factors. *Cell Chem. Biol.* **2019**, *26*, 71-84.e4.
(4) Huynh, K.; Mellett, N. A.; Duong, T.; Nguyen, A.; Meikle, T. G.; Giles, C.; Meikle, P. J. A Comprehensive, Curated, High-Throughput Method for the Detailed Analysis of the Plasma Lipidome. *Application Note - Agilent.* **2021**, 1-45. https://www.agilent.com/cs/library/applications/an-plasma-lipidomics-6495-lc-ms-ms-5994-3747en-agilent.pdf
(5) https://metabolomics.baker.edu.au/method/
(6) Wiesner, P.; Leidl, K.; Boettcher, A.; Schmitz, G.; Liebisch, G. Lipid Profiling of FPLC-Separated Lipoprotein Fractions by Electrospray Ionization Tandem Mass Spectrometry. *J. Lipid Res.* **2009**, *50*, 574–585.
(7) Dambrova, M.; Makrecka-Kuka, M.; Kuka, J.; Vilskersts, R.; Nordberg, D.; Attwood, M. M.; Smesny, S.; Sen, Z. D.; Guo, A. C.; Oler, E.; Tian, S.; Zheng, J.; Wishart, D. S.; Liepinsh, E.; Schiöth, H. B. Acylcarnitines: Nomenclature, Biomarkers, Therapeutic Potential, Drug Targets, and Clinical Trials. *Pharmacol. Rev.* **2022**, *74*, 506–551.
(8) Ala-Korpela, M.; Zhao, S.; Järvelin, M.-R.; Mäkinen, V.-P.; Ohukainen, P. Apt Interpretation of Comprehensive Lipoprotein Data in Large-Scale Epidemiology: Disclosure of Fundamental Structural and Metabolic Relationships. *Int. J. Epidemiol.* **2022**, *51*, 996–1011.
(9) Goldstein, J. L.; Brown, M. S. A Century of Cholesterol and Coronaries: From Plaques to Genes to Statins. *Cell* **2015**, *161*, 161–172.
(10) Schmidt, A. F.; Joshi, R.; Gordillo-Marañón, M.; Drenos, F.; Charoen, P.; Giambartolomei, C.; Bis, J. C.; Gaunt, T. R.; Hughes, A. D.; Lawlor, D. A.; Wong, A.; Price, J. F.; Chaturvedi, N.; Wannamethee, G.; Franceschini, N.; Kivimaki, M.; Hingorani, A. D.; Finan, C. Biomedical Consequences of Elevated Cholesterol-Containing Lipoproteins and Apolipoproteins on Cardiovascular and Non-Cardiovascular Outcomes. *Commun. Med.* **2023**, *3*, 9.
(11) Mäkinen, V.-P.; Forsblom, C.; Thorn, L. M.; Wadén, J.; Gordin, D.; Heikkilä, O.; Hietala, K.; Kyllönen, L.; Kytö, J.; Rosengård-Bärlund, M.; Saraheimo, M.; Tolonen, N.; Parkkonen, M.; Kaski, K.; Ala-Korpela, M.; Groop, P.-H.; FinnDiane Study Group. Metabolic Phenotypes, Vascular Complications, and Premature Deaths in a Population of 4,197 Patients with Type 1 Diabetes. *Diabetes* **2008**, *57*, 2480–2487.
(12) Hyvönen, M. T.; Hiltunen, Y.; El-Deredy, W.; Ojala, T.; Vaara, J.; Kovanen, P. T.; Ala-Korpela, M. Application of Self-Organizing Maps in Conformational Analysis of Lipids. *J. Am. Chem. Soc.* **2001**, *123*, 810–816.
(13) Gao, S.; Mutter, S.; Casey, A.; Mäkinen, V.-P. Numero: A Statistical Framework to Define Multivariable Subgroups in Complex Population-Based Datasets. *Int. J. Epidemiol.* **2019**, *48*, 369–374.
(14) Kettunen, J.; Holmes, M. V.; Allara, E.; Anufrieva, O.; Ohukainen, P.; Oliver-Williams, C.; Wang, Q.; Tillin, T.; Hughes, A. D.; Kähönen, M.; Lehtimäki, T.; Viikari, J.; Rai-takari, O. T.; Salomaa, V.; Järvelin, M.-R.; Perola, M.; Smith, G. D.; Chaturvedi, N.; Danesh, J.; Angelantonio, E. D.; Butter-worth, A. S.; Ala-Korpela, M. Lipoprotein Signatures of Cho-lesteryl Ester Transfer Protein and HMG-CoA Reductase Inhibition. PLOS Biol. 2019, 17, e3000572.

Funding

The NFBC has been supported, for instance, by EU, Academy of Finland, PREcisE, Joint Programming Initiative a Healthy Diet for a Healthy Life (no. 655), UK Medical Research Council, Biotechnology and Biological Sciences Research Council (MR/S03658X/1), and European Regional Development Fund Grant no. 539/2010 A31592. The Young Finns Study has been financially supported by the Academy of Finland: grants 356405, 322098, 286284, 134309 (Eye), 126925, 121584, 124282, 129378 (Salve), 117797 (Gendi), and 141071 (Skidi); the Social Insurance Institution of Finland; Competitive State Research Financing of the Expert Responsibility area of Kuopio, Tampere and Turku University Hospitals (grant X51001); Juho Vainio Foundation; Paavo Nurmi Foundation; Finnish Foundation for Cardiovascular Research; Finnish Cultural Foundation; The Sigrid Juselius Foundation; Tampere Tuberculosis Foundation; Emil Aaltonen Foundation; Yrjö Jahnsson Foundation; Signe and Ane Gyllenberg Foundation; Diabetes Research Foundation of Finnish Diabetes Association; EU Horizon 2020 (grant 755320 for TAXINOMISIS and grant 848146 for To Aition); European Research Council (grant 742927 for MULTIEPIGEN project); Tampere University Hospital Supporting Foundation; Finnish Society of Clinical Chemistry; the Cancer Foundation Finland; pBETTER4U_EU (Preventing obesity through Biologically and bEhaviorally Tailored inTERventions for you; project number: 101080117); CVDLink (EU grant nro.) and the Jane and Aatos Erkko Foundation. C.G., K.H., and P.J.M. were supported by Investigator grants (2027256, 1197190, and 2009965, respectively) from the National Health and Medical Research Council of Australia. In addition, their work was supported by the Victorian Government's Operational Infrastructure Support Program. M.A.-K. was supported by a research grant from the Sigrid Juselius Foundation, the Finnish Foundation for Cardiovascular Research, and the Research Council of Finland (grant no. 357183).

Abbreviations

AC, Acylcarnitines
ALT, Alanine aminotransferase
APOA1, Apolipoprotein A-I
APOB, Apolipoprotein B
APOB/APOA1, Ratio of apolipoprotein B to apolipoprotein A-I
BMI, Body mass index
C, Cholesterol
C1P, Ceramide-1-phosphates
CE, Cholesteryl esters
Cer(d), Ceramides
Cer(m), Deoxyceramides
cIMT, Carotid artery intima-media thickness
CKD, Chronic kidney disease
CRP, C-reactive protein
DE, Dehydrocholesterol esters (refers to all cholesterol precursors with an additional double bond; see Table S4 for a note)
DG, Diacylglycerols
DHA, Docosahexaenoic acid (22:6)
DHA/FA, Ratio of docosahexaenoic acid to total fatty acids
dhCer, Dihydroceramides
ESTC, Esterified cholesterol
FAW3, Omega-3 fatty acids
FAW3/FA, Ratio of omega-3 fatty acids to total fatty acids
FAW6, Omega-6 fatty acids
FAW6/FA, Ratio of omega-6 fatty acids to total fatty acids
FC, Free cholesterol
FFA, Free fatty acids
GM1, GM1 gangliosides
GM3, GM3 gangliosides
HDL, High-density lipoprotein
HDL-C, Total cholesterol in HDL particles
HDL-TG, Triglycerides in HDL particles
HDL2-C, Total cholesterol in HDL2 particles
HDL3-C, Total cholesterol in HDL3 particles
Hex2Cer, Dihexosylceramides
Hex3Cer, Trihexosylceramides
HexCer, Monohexosylceramides
IDL, Intermediate-density lipoprotein
L, Large (in relation to lipoprotein subclass particle size)

L, Total lipid concentration (in relation to lipoprotein subclasses)
LA, Linoleic acid (18:2)
LA/FA, Ratio of linoleic acid to total fatty acids
LC, Liquid chromatography
LDL, Low-density lipoprotein
LDL-C, Total cholesterol in LDL particles
LDL-TG, Triglycerides in LDL particles
Lp(a), Lipoprotein (a)
LPC, Lysophosphatidylcholines
LPC(O), Lysoalkylphosphatidylcholines
LPC(P), Lysoalkenylphosphatidylcholines (plasmalogens)
LPE, Lysophosphatidylethanolamines
LPE(P), Lysoalkenylphosphatidylethanolamines (plasmalogens)
LPI, Lysophosphatidylinositols
M, Medium (in relation to lipoprotein subclass particle size)
MS, Mass spectrometry
MUFA, Monounsaturated fatty acids
MUFA/FA, Ratio of monounsaturated fatty acids to total fatty acids
NMR, Nuclear magnetic resonance
NFBC66, Northern Finland Birth Cohort 1966
Ox-CE, Oxidised cholesteryl esters
Ox-PC/LPC, Oxidised Phosphatidylcholines and lysophosphatidylcholines
PA, Phosphatidic acids
PC, Phosphatidylcholine and other cholines
PC, Phosphatidylcholines
PC(O), Alkylphosphatidylcholines
PC(P), Alkenylphosphatidylcholines (plasmalogens)
PE, Phosphatidylethanolamines
PE(O), Alkylphosphatidylethanolamines
PE(P), Alkenylphosphatidylethanolamines (plasmalogens)
PG, Phosphatidylglycerols
PI, Phosphatidylinositols
PIP1, Phosphatidylinositol monophosphates
PL, Phospholipids
PS, Phosphatidylserines
PUFA, Polyunsaturated fatty acids
PUFA/FA, Ratio of polyunsaturated fatty acids to total fatty acids
REMNANT-C, Remnant cholesterol, i.e., (non-HDL, non-LDL)-cholesterol
S, Small (in relation to lipoprotein subclass particle size)
S1P, Sphingosine-1-phosphates
SERUM-C, Serum total cholesterol

SERUM-TG, Serum total triglycerides
SFA, Saturated fatty acids
SFA/FA, Ratio of saturated fatty acids to total fatty acids
SHexCer, Sulfatides
SM, Sphingomyelins
SM, Sphingomyelins
SOM, Self-organizing map
Sph, Sphingosines
TG, Triglycerides (NMR)
TG [NL], Triglycerides (LC-MS/MS)
TG [SIM], Triglycerides (LC-MS/MS)
TG(O) [SIM], Alkyldiacylglycerols
TG/PG, Ratio of triglycerides to phosphoglycerides
FA, Total fatty acids
TOTPG, Total phosphoglycerides
UNSAT, Estimated degree of unsaturation
VLDL, very-low-density lipoprotein
VLDL-C, Total cholesterol in VLDL particles
VLDL-TG, Triglycerides in VLDL particles
XL, Very large (in relation to lipoprotein subclass particle size)
XS, Very small (in relation to lipoprotein subclass particle size)
XXL, Extremely large (in relation to lipoprotein subclass particle size)
YFS, Cardiovascular Risk in Young Finns Study

Supplement Tables

Table S1.

Clinical characteristics in NFBC66 and YFS. The values are median [Q1 – Q3].

Clinical characteristic	NFBC66	YFS
Number of participants	5,657	2,173
Number of females (%)	3,168 (56.0 %)	1,190 (54.8 %)
Age (year)	46.6 [46.2 - 47.1]	39.0 [33.0 - 42.0]
BMI (kg/m2)	26.1 [23.5 - 29.3]	25.8 [23.1 - 29.1]
C-reactive protein [CRP] (mg/L)	0.83 [0.45 - 1.70]	0.88 [0.41 - 1.91]
CKD Chien risk	6.55 [4.94 - 9.00]	3.50 [2.2 - 5.23]
Alanine aminotransferase [ALT] (U/L)	25.0 [18.0 -36.0]	14.0 [10.0 - 22.0]
Lipoprotein (a) [Lp(a)] (mg/L)	NA	75.0 [33.0 - 188.0]
Carotid artery intima-media thickness [cIMT] (mm)	NA	0.62 [0.56 – 0.68]

NA refers to not available.

Table S2.

NMR-based lipoprotein measures in NFBC66 and YFS. The values are median [Q1 – Q3].

	NFBC66	YFS
**Lipoprotein subclass concentration measures**		
**Extremely large very-low-density lipoprotein subclass (XXL-VLDL)**		
XXL-VLDL-P (mol/L)	9.14E-11 [5.6E-11 - 1.6E-11]	7.4E-11 [3.7E-11 - 1.5E-10]
XXL-VLDL-L (mmol/L)	0.02 [0.01 - 0.03]	0.016 [0.0079 - 0.0325]
XXL-VLDL-PL (mmol/L)	0.0023 [0.0014 - 0.0042]	0.0018 [0.0008 - 0.0039]
XXL-VLDL-C (mmol/L)	0.0039 [0.0022 - 0.0066]	0.0033 [0.0015 - 0.0067]
XXL-VLDL-CE (mmol/L)	0.0025 [0.0014 - 0.0041]	0.0021 [0.0009 - 0.0041]
XXL-VLDL-FC (mmol/L)	0.0014 [0.0008 - 0.0026]	0.0012 [0.0005 - 0.0027]
XXL-VLDL-TG (mmol/L)	0.0134 [0.0083 - 0.0233]	0.0108 [0.0056 - 0.022]
**Very large very-low-density lipoprotein subclass (XL-VLDL)**		
XL-VLDL-P (mol/L)	4.5E-10 [2.0E-10 - 9.4E-10]	3.7E-10 [1.3E-10 - 8.6E-10]
XL-VLDL-L (mmol/L)	0.04 [0.02 - 0.09]	0.036 [0.013 - 0.085]
XL-VLDL-PL (mmol/L)	0.0083 [0.0041 - 0.016]	0.007 [0.002 - 0.015]
XL-VLDL-C (mmol/L)	0.0095 [0.0049 - 0.0182]	0.009 [0.003 - 0.019]
XL-VLDL-CE (mmol/L)	0.0046 [0.0021 - 0.0093]	0.005 [0.001 - 0.01]
XL-VLDL-FC (mmol/L)	0.0049 [0.0028 - 0.009]	0.004 [0.002 - 0.009]
XL-VLDL-TG (mmol/L)	0.0268 [0.011 - 0.0576]	0.021 [0.007 - 0.052]
**Large very-low-density lipoprotein subclass (L-VLDL)**		
L-VLDL-P (mol/L)	3.3E-9 [1.6E-9 - 6.4E-9]	2.8E-9 [1.3E-9 - 5.8E-9]
L-VLDL-L (mmol/L)	0.19 [0.093 - 0.371]	0.17 [0.08 - 0.34]
L-VLDL-PL (mmol/L)	0.04 [0.02 - 0.07]	0.03 [0.02 - 0.06]
L-VLDL-C (mmol/L)	0.04 [0.02 - 0.08]	0.04 [0.02 - 0.08]
L-VLDL-CE (mmol/L)	0.02 [0.01 - 0.04]	0.02 [0.01 - 0.05]
L-VLDL-FC (mmol/L)	0.02 [0.01 - 0.04]	0.02 [0.01 - 0.04]
L-VLDL-TG (mmol/L)	0.11 [0.05 - 0.22]	0.09 [0.04 - 0.19]
**Medium very-low-density lipoprotein subclass (M-VLDL)**		
M-VLDL-P (mol/L)	1.5E-8 [9.7E-9 - 2.3E-8]	1.4E-8 [9.2E-9 - 2.2E-8]
M-VLDL-L (mmol/L)	0.5 [0.33 - 0.79]	0.48 [0.32 - 0.76]
M-VLDL-PL (mmol/L)	0.11 [0.07 - 0.16]	0.1 [0.07 - 0.15]
M-VLDL-C (mmol/L)	0.16 [0.11 - 0.23]	0.15 [0.11 - 0.23]
M-VLDL-CE (mmol/L)	0.1 [0.07 - 0.13]	0.1 [0.07 - 0.13]
M-VLDL-FC (mmol/L)	0.06 [0.04 - 0.09]	0.06 [0.04 - 0.09]
M-VLDL-TG (mmol/L)	0.24 [0.15 - 0.4]	0.22 [0.14 - 0.38]

**Small very-low-density lipoprotein subclass (S-VLDL)**		
S-VLDL-P (mol/L)	3.0E-8 [2.2E-8 - 4.0E-8]	2.9E-8 [2.2E-8 - 3.9E-8]
S-VLDL-L (mmol/L)	0.6 [0.44 - 0.79]	0.59 [0.44 - 0.78]
S-VLDL-PL (mmol/L)	0.14 [0.1 - 0.18]	0.13 [0.1 - 0.17]
S-VLDL-C (mmol/L)	0.26 [0.2 - 0.32]	0.25 [0.19 - 0.31]
S-VLDL-CE (mmol/L)	0.17 [0.13 - 0.21]	0.16 [0.12 - 0.2]
S-VLDL-FC (mmol/L)	0.09 [0.06 - 0.11]	0.09 [0.06 - 0.11]
S-VLDL-TG (mmol/L)	0.2 [0.14 - 0.29]	0.21 [0.14 - 0.3]
**Very small very-low-density lipoprotein subclass (XS-VLDL)**		
XS-VLDL-P (mol/L)	4.7E-8 [3.9E-8 - 5.5E-8]	4.5E-8 [3.7E-8 - 5.3E-8]
XS-VLDL-L (mmol/L)	0.6 [0.51 - 0.71]	0.57 [0.48 - 0.68]
XS-VLDL-PL (mmol/L)	0.18 [0.16 - 0.22]	0.17 [0.15 - 0.2]
XS-VLDL-C (mmol/L)	0.32 [0.27 - 0.37]	0.29 [0.24 - 0.34]
XS-VLDL-CE (mmol/L)	0.22 [0.18 - 0.25]	0.2 [0.16 - 0.23]
XS-VLDL-FC (mmol/L)	0.1 [0.09 - 0.12]	0.09 [0.08 - 0.11]
XS-VLDL-TG (mmol/L)	0.1 [0.08 - 0.13]	0.11 [0.08 - 0.14]
**Intermediate-density lipoprotein subclass (IDL)**		
IDL-P (mol/L)	1.4E-7 [1.2E-7 - 1.6E-7]	1.3E-7 [1.1E-7 - 1.5E-7]
IDL-L (mmol/L)	1.4 [1.2 - 1.63]	1.3 [1.11 - 1.52]
IDL-PL (mmol/L)	0.37 [0.32 - 0.43]	0.35 [0.3 - 0.41]
IDL-C (mmol/L)	0.92 [0.78 - 1.08]	0.83 [0.7 - 0.97]
IDL-CE (mmol/L)	0.66 [0.57 - 0.77]	0.59 [0.5 - 0.69]
IDL-FC (mmol/L)	0.26 [0.22 - 0.3]	0.24 [0.2 - 0.28]
IDL-TG (mmol/L)	0.11 [0.09 - 0.13]	0.11 [0.09 - 0.15]
**Large low-density lipoprotein subclass (L-LDL)**		
L-LDL-P (mol/L)	2.3E-7 [2.0E-7 - 2.7E-7]	2.1E-7 [1.8E-7 - 2.5E-7]
L-LDL-L (mmol/L)	1.63 [1.39 - 1.92]	1.5 [1.27 - 1.78]
L-LDL-PL (mmol/L)	0.4 [0.35 - 0.46]	0.37 [0.33 - 0.43]
L-LDL-C (mmol/L)	1.13 [0.95 - 1.35]	1.02 [0.85 - 1.22]
L-LDL-CE (mmol/L)	0.82 [0.68 - 0.99]	0.73 [0.6 - 0.88]
L-LDL-FC (mmol/L)	0.31 [0.27 - 0.36]	0.29 [0.24 - 0.34]
L-LDL-TG (mmol/L)	0.09 [0.08 - 0.11]	0.1 [0.08 - 0.13]
**Medium low-density lipoprotein subclass (M-LDL)**		
M-LDL-P (mol/L)	1.8E-7 [1.5E-7 - 2.2E-7]	1.6E-7 [1.4E-7 - 2.0E-7]
M-LDL-L (mmol/L)	0.94 [0.78 - 1.13]	0.84 [0.7 - 1.02]
M-LDL-PL (mmol/L)	0.24 [0.21 - 0.28]	0.23 [0.2 - 0.26]
M-LDL-C (mmol/L)	0.66 [0.54 - 0.8]	0.57 [0.46 - 0.7]
M-LDL-CE (mmol/L)	0.48 [0.38 - 0.6]	0.41 [0.32 - 0.52]
M-LDL-FC (mmol/L)	0.17 [0.15 - 0.2]	0.16 [0.14 - 0.18]
M-LDL-TG (mmol/L)	0.04 [0.03 - 0.05]	0.05 [0.04 - 0.06]

**Small low-density lipoprotein subclass (S-LDL)**		
S-LDL-P (mol/L)	2.1E-7 [1.8E-7 - 2.5E-7]	1.9E-7 [1.6E-7 - 2.3E-7]
S-LDL-L (mmol/L)	0.59 [0.5 - 0.71]	0.53 [0.44 - 0.64]
S-LDL-PL (mmol/L)	0.17 [0.15 - 0.2]	0.16 [0.14 - 0.18]
S-LDL-C (mmol/L)	0.39 [0.32 - 0.48]	0.34 [0.27 - 0.42]
S-LDL-CE (mmol/L)	0.29 [0.23 - 0.36]	0.24 [0.19 - 0.31]
S-LDL-FC (mmol/L)	0.11 [0.09 - 0.12]	0.1 [0.08 - 0.11]
S-LDL-TG (mmol/L)	0.027 [0.021 - 0.035]	0.03 [0.02 - 0.04]
**Very large high-density lipoprotein subclass (XL-HDL)**		
XL-HDL-P (mol/L)	2.9E-7 [2.4E-7 - 5.9E-7]	3.7E-7 [2.3E-7 - 5.6E-7]
XL-HDL-L (mmol/L)	0.39 [0.24 - 0.6]	0.37 [0.23 - 0.57]
XL-HDL-PL (mmol/L)	0.18 [0.09 - 0.3]	0.19 [0.1 - 0.3]
XL-HDL-C (mmol/L)	0.19 [0.13 - 0.28]	0.17 [0.11 - 0.25]
XL-HDL-CE (mmol/L)	0.14 [0.09 - 0.2]	0.12 [0.07 - 0.18]
XL-HDL-FC (mmol/L)	0.05 [0.03 - 0.08]	0.05 [0.03 - 0.08]
XL-HDL-TG (mmol/L)	0.02 [0.01 - 0.02]	0.02 [0.01 - 0.02]
**Large high-density lipoprotein subclass (L-HDL)**		
L-HDL-P	1.3E-6 [3.2E-7 - 1.8E-6]	1.3E-6 [8.8E-7 - 1.8E-6]
L-HDL-L (mmol/L)	0.81 [0.52 - 1.12]	0.82 [0.54 - 1.14]
L-HDL-PL (mmol/L)	0.41 [0.28 - 0.54]	0.41 [0.29 - 0.55]
L-HDL-C (mmol/L)	0.36 [0.2 - 0.53]	0.38 [0.23 - 0.55]
L-HDL-CE (mmol/L)	0.28 [0.16 - 0.41]	0.29 [0.18 - 0.42]
L-HDL-FC (mmol/L)	0.08 [0.04 - 0.12]	0.08 [0.04 - 0.12]
L-HDL-TG (mmol/L)	0.04 [0.03 - 0.05]	0.03 [0.02 - 0.05]
**Medium high-density lipoprotein subclass (M-HDL)**		
M-HDL-P (mol/L)	2.4E-6 [2.1E-6 - 2.7E-6]	2.5E-6 [2.2E-6 - 2.8E-6]
M-HDL-L (mmol/L)	1.03 [0.91 - 1.16]	1.04 [0.92 - 1.21]
M-HDL-PL (mmol/L)	0.47 [0.42 - 0.52]	0.47 [0.42 - 0.55]
M-HDL-C (mmol/L)	0.52 [0.45 - 0.59]	0.52 [0.45 - 0.61]
M-HDL-CE (mmol/L)	0.42 [0.36 - 0.48]	0.42 [0.36 - 0.49]
M-HDL-FC (mmol/L)	0.1 [0.08 - 0.11]	0.1 [0.08 - 0.12]
M-HDL-TG (mmol/L)	0.05 [0.04 - 0.06]	0.05 [0.04 - 0.06]
**Small high-density lipoprotein subclass (S-HDL)**		
S-HDL-P (mol/L)	5.5E-6 [5.1E-6 - 5.9E-6]	5.4E-6 [5.0E-6 -5.9E-6]
S-HDL-L (mmol/L)	1.23 [1.14 - 1.32]	1.2 [1.1 - 1.31]
S-HDL-PL (mmol/L)	0.64 [0.59 - 0.7]	0.64 [0.59 - 0.71]
S-HDL-C (mmol/L)	0.54 [0.49 - 0.59]	0.51 [0.45 - 0.57]
S-HDL-CE (mmol/L)	0.41 [0.36 - 0.46]	0.38 [0.33 - 0.44]
S-HDL-FC (mmol/L)	0.13 [0.12 - 0.14]	0.13 [0.12 - 0.14]
S-HDL-TG (mmol/L)	0.05 [0.04 - 0.06]	0.04 [0.03 - 0.06]

**Lipoprotein subclass lipid compositions**		
**Lipid percentages of extremely large very-low-density lipoprotein subclass (XXL-VLDL)**		
XXL-VLDL-PL %	12.02 [11.03 - 12.74]	11.28 [9.83 - 12.18]
XXL-VLDL-C %	19.06 [16.72 - 21.48]	20.08 [17.52 - 22.69]
XXL-VLDL-CE %	12.11 [9.49 - 14.62]	12.49 [9.66 - 15.39]
XXL-VLDL-FC %	7.21 [6.1 - 8.02]	7.7 [6.41 - 8.47]
XXL-VLDL-TG %	69.02 [66.73 - 71.37]	68.74 [66.18 - 71.59]
**Lipid percentage of very large very-low-density lipoprotein subclass (XL-VLDL)**		
XL-VLDL-PL %	17.84 [16.72 - 19.86]	17.36 [16.49 - 19.24]
XL-VLDL-C %	21.01 [18.18 - 24.85]	23.41 [20.42 - 26.94]
XL-VLDL-CE %	10.37 [8.43 - 12.4]	12.07 [9.87 - 14.29]
XL-VLDL-FC %	10.48 [9.14 - 12.87]	10.99 [9.79 - 13.16]
XL-VLDL-TG %	61.14 [55.64 - 64.66]	59.16 [54.43 - 62.37]
**Lipid percentage of large very-low-density lipoprotein subclass (L-VLDL)**		
L-VLDL-PL %	18.96 [18.37 - 19.96]	18.8 [18.24 - 19.87]
L-VLDL-C %	22.51 [20.62 - 24.75]	24.73 [22.66 - 27.07]
L-VLDL-CE %	12.18 [10.37 - 14.38]	14.3 [12.05 - 16.75]
L-VLDL-FC %	10.54 [9.61 - 11.19]	10.75 [9.22 - 11.68]
L-VLDL-TG %	58.39 [55.61 - 60.55]	56.3 [53.46 - 58.61]
**Lipid percentage of medium very-low-density lipoprotein subclass (M-VLDL)**		
M-VLDL-PL %	21.08 [20.35 - 21.91]	21.03 [20.33 - 21.85]
M-VLDL-C %	30.48 [27.73 - 33.94]	31.78 [28.95 - 35.08]
M-VLDL-CE %	19.15 [16.12 - 22.89]	20.17 [17.16 - 23.75]
M-VLDL-FC %	11.48 [10.88 - 11.88]	11.68 [11.01 - 12.17]
M-VLDL-TG %	48.4 [44.19 - 51.87]	47.13 [43.04 - 50.55]
**Lipid percentage of small very-low-density lipoprotein subclass (S-VLDL)**		
S-VLDL-PL %	22.63 [21.88 - 23.49]	22.44 [21.69 - 23.44]
S-VLDL-C %	42.91 [39.19 - 46.8]	41.39 [37.86 - 45.63]
S-VLDL-CE %	28.64 [25.07 - 32.39]	27.02 [23.73 - 31.11]
S-VLDL-FC %	14.18 [13.73 - 14.62]	14.24 [13.77 - 14.8]
S-VLDL-TG %	34.13 [30.1 - 38.31]	35.73 [31.52 - 39.8]
**Lipid percentage of very small very-low-density lipoprotein subclass (XS-VLDL)**		
XS-VLDL-PL %	30.56 [29.43 - 31.62]	30.47 [28.92 - 32.08]
XS-VLDL-C %	52.85 [50.39 - 55.04]	50.39 [47.31 - 53.17]
XS-VLDL-CE %	36.04 [33.77 - 38.15]	34.13 [31.3 - 36.82]
XS-VLDL-FC %	16.77 [16.16 - 17.32]	16.27 [15.62 - 16.84]
XS-VLDL-TG %	16.48 [14.12 - 19.32]	18.97 [15.99 - 22.32]
**Lipid percentage of intermediate-density lipoprotein subclass (IDL)**		
IDL-PL %	26.75 [26.34 - 27.16]	27.01 [26.54 - 27.55]

IDL-C %	65.76 [64.57 - 66.69]	63.95 [62.33 - 65.21]
IDL-CE %	47.18 [46.05 - 48.19]	45.44 [44.03 - 46.64]
IDL-FC %	18.62 [18 - 19.05]	18.57 [17.92 - 19.05]
IDL-TG %	7.48 [6.53 - 8.73]	8.96 [7.69 - 10.5]
**Lipid percentage of large low-density lipoprotein subclass (L-LDL)**		
L-LDL-PL %	24.69 [24.05 - 25.44]	25.02 [24.26 - 25.86]
L-LDL-C %	69.56 [68.32 - 70.5]	68.07 [66.48 - 69.34]
L-LDL-CE %	50.45 [49.02 - 51.63]	48.87 [47.17 - 50.15]
L-LDL-FC %	19.2 [18.64 - 19.68]	19.29 [18.65 - 19.82]
L-LDL-TG %	5.67 [4.97 - 6.55]	6.72 [5.84 - 8.03]
**Lipid percentage of medium low-density lipoprotein subclass (M-LDL)**		
M-LDL-PL %	25.68 [24.55 - 27.1]	26.75 [25.29 - 28.32]
M-LDL-C %	69.79 [67.96 - 71.11]	67.63 [65.24 - 69.47]
M-LDL-CE %	51.21 [48.67 - 53.17]	48.66 [45.54 - 51.21]
M-LDL-FC %	18.55 [17.71 - 19.52]	18.84 [17.96 - 19.99]
M-LDL-TG %	4.48 [3.85 - 5.27]	5.51 [4.63 - 6.8]
**Lipid percentage of small low-density lipoprotein subclass (S-LDL)**		
S-LDL-PL %	29.25 [27.73 - 31.22]	29.72 [27.82 - 31.96]
S-LDL-C %	66.19 [63.9 - 67.8]	64.78 [61.82 - 66.92]
S-LDL-CE %	48.34 [45.37 - 50.5]	46.6 [43.08 - 49.48]
S-LDL-FC %	17.81 [17.07 - 18.69]	18.0 [17.18 - 19.02]
S-LDL-TG %	4.49 [3.85 - 5.37]	5.39 [4.42 - 6.75]
**Lipid percentage of very large high-density lipoprotein subclass (XL-HDL)**		
XL-HDL-PL %	48.05 [40.45 - 51.79]	51.15 [44.61 - 55.44]
XL-HDL-C %	47.92 [44.52 - 53.42]	45.23 [40.98 - 50.27]
XL-HDL-CE %	34.69 [31.31 - 40.03]	31.27 [27.37 - 35.6]
XL-HDL-FC %	13.31 [12.54 - 14.07]	14.04 [12.94 - 15.22]
XL-HDL-TG %	3.97 [2.97 - 6.32]	3.71 [2.64 - 6]
**Lipid percentage of large high-density lipoprotein subclass (L-HDL)**		
L-HDL-PL %	50.44 [47.76 - 54.01]	50.25 [47.71 - 53.69]
L-HDL-C %	45.06 [40.16 - 48.17]	45.76 [41.72 - 48.42]
L-HDL-CE %	35.43 [32.32 - 37.51]	36.05 [33.48 - 37.85]
L-HDL-FC %	9.69 [7.96 - 10.74]	9.74 [8.16 - 10.73]
L-HDL-TG %	4.65 [3.75 - 6.08]	4.06 [3.19 - 5.23]
**Lipid percentage of medium high-density lipoprotein subclass (M-HDL)**		
M-HDL-PL %	45.41 [44.79 - 46.15]	45.5 [44.82 - 46.22]
M-HDL-C %	50.16 [48.45 - 51.5]	49.91 [48.24 - 51.24]
M-HDL-CE %	40.62 [39.1 - 41.81]	40.28 [38.78 - 41.58]
M-HDL-FC %	9.59 [9.23 - 9.87]	9.6 [9.25 - 9.96]
M-HDL-TG %	4.39 [3.46 - 5.58]	4.58 [3.72 - 5.7]

**Lipid percentage of small high-density lipoprotein subclass (S-HDL)**		
S-HDL-PL %	52.67 [50.1 - 55.06]	54.05 [51.32 - 56.69]
S-HDL-C %	43.69 [41.26 - 46.16]	42.17 [39.42 - 45.04]
S-HDL-CE %	33.25 [30.61 - 36.08]	31.65 [28.51 - 34.66]
S-HDL-FC %	10.33 [9.91 - 10.76]	10.6 [10.19 - 11.04]
S-HDL-TG %	3.65 [2.92 - 4.52]	3.64 [2.88 - 4.59]
**Lipoprotein particle size (nm)**		
VLDL-D	36.02 [35.15 - 37.09]	35.96 [35.09 - 37.04]
LDL-D	23.57 [23.51 - 23.65]	23.63 [23.55 - 23.71]
HDL-D	9.93 [9.72 - 10.16]	9.95 [9.76 - 10.16]
**Cholesterol (mmol/L)**		
SERUM-C	5.62 [4.97 - 6.35]	5.16 [4.56 - 5.92]
VLDL-C	0.8 [0.63 - 1.02]	0.76 [0.59 - 0.98]
REMNANT-C	1.74 [1.45 - 2.09]	1.6 [1.32 - 1.94]
LDL-C	2.18 [1.81 - 2.63]	1.93 [1.58 - 2.33]
Non-HDL cholesterol	3.93 [3.3 - 4.69]	3.53 [2.92 - 4.24]
HDL-C	1.63 [1.36 - 1.92]	1.6 [1.34 - 1.9]
HDL2-C	1.1 [0.84 - 1.37]	1.08 [0.83 - 1.36]
HDL3-C	0.53 [0.51 - 0.55]	0.52 [0.5 - 0.55]
Esterified cholesterol	4.01 [3.53 - 4.54]	3.58 [3.13 - 4.13]
Free cholesterol	1.61 [1.43 - 1.82]	1.59 [1.42 - 1.8]
**Glycerides and phospholipids (mmol/L)**		
SERUM-TG	1.13 [0.83 - 1.61]	1.11 [0.81 - 1.61]
VLDL-TG	0.69 [0.44 - 1.13]	0.66 [0.43 - 1.08]
LDL-TG	0.16 [0.13 - 0.2]	0.18 [0.14 - 0.23]
HDL-TG	0.15 [0.13 - 0.18]	0.14 [0.12 - 0.18]
Total phosphoglycerides	2.38 [2.12 - 2.65]	2.42 [2.15 - 2.76]
Ratio of triglycerides to phosphoglycerides	0.55 [0.42 - 0.72]	0.49 [0.4 - 0.64]
Phosphatidylcholine and other cholines	2.41 [2.17 - 2.67]	2.24 [2.01 - 2.57]
Sphingomyelins	0.58 [0.53 - 0.64]	0.54 [0.48 - 0.6]
Total cholines	2.97 [2.69 - 3.25]	2.75 [2.49 - 3.09]
**Apolipoprotein**		
Apolipoprotein A-I (g/L)	1.73 [1.59 - 1.89]	1.66 [1.52 - 1.84]
Apolipoprotein B (g/L)	1.03 [0.88 - 1.21]	0.96 [0.82 - 1.13]
APOB/APOA-I	0.6 [0.49 - 0.72]	0.57 [0.48 - 0.69]

**Total fatty acids and saturation measures**		
Total fatty acids (mmol/L)	13.95 [12.59 - 15.63]	13.17 [11.55 - 15.31]
Estimated degree of unsaturation	1.17 [1.13 - 1.21]	1.15 [1.11 - 1.18]
**Fatty acids**		
DHA (mmol/L)	0.19 [0.16 - 0.23]	0.23 [0.19 - 0.28]
LA (mmol/L)	3.68 [3.33 - 4.07]	3.33 [2.96 - 3.77]
**Fatty acids by saturation measures**		
Omega-3 fatty acids (mmol/L)	0.6 [0.5 - 0.72]	0.56 [0.46 - 0.7]
Omega-6 fatty acids (mmol/L)	4.54 [4.11 - 5]	4.11 [3.67 - 4.67]
PUFA (mmol/L)	5.17 [4.66 - 5.69]	4.69 [4.17 - 5.34]
MUFA (mmol/L)	3.55 [3.04 - 4.24]	3.5 [2.97 - 4.33]
SFA (mmol/L)	5.22 [4.71 - 5.84]	4.93 [4.3 - 5.75]
**Fatty acids (%)**		
DHA/FA	1.37 [1.16 - 1.63]	1.74 [1.52 - 2.01]
LA/FA	26.46 [24.88 - 27.9]	25.28 [23.15 - 27.22]
FAW3/FA	4.24 [3.66 - 4.99]	4.23 [3.65 - 4.91]
FAW6/FA	32.62 [30.8 - 34.16]	31.38 [29.13 - 33.27]
PUFA/FA	36.85 [34.82 - 38.83]	35.66 [33.31 - 37.79]
MUFA/FA	25.7 [23.67 - 27.86]	26.89 [24.99 - 28.85]
SFA/FA	37.43 [36.09 - 38.75]	37.46 [36.23 - 38.72]

Table S3.

LC-MS/MS lipidomics measures for the total concentrations of the circulating lipid classes (µmol/L) in NFBC66 and YFS. The values are median [Q1 – Q3]. The clusters refer to Figure 4 in the main text.

	**NFBC66**	**YFS**
**Cluster no.1 (mol %)**	**1.1 %**	**1.1 %**
Deoxyceramide [Cer(m)]	0.49 [0.36 - 0.69]	0.41 [0.29 - 0.60]
Phosphatidylinositol [PI]	73.06 [61.12 - 87.26]	70.22 [56.31 - 85.44]
Phosphatidylethanolamine [PE]	26.12 [19.75 - 35.19]	26.12 [18.27 - 38.06]
**Cluster no.2 (mol %)**	**0.11 %**	**0.11 %**
Ceramide [Cer(d)]	9.95 [8.21 - 11.9]	8.96 [6.77 - 11.18]
Dihydroceramide [dhCer]	0.67 [0.55 - 0.81]	0.59 [0.46 - 0.75]
**Cluster no.3 (mol %)**	**21.1 %**	**20.2 %**
Phosphatidylcholine [PC]	1901.35 [1667.36 - 2133.32]	1964.87 [1656.30 - 2210.23]
**Cluster no.4 (mol %)**	**0.05 %**	**0.05 %**
Oxidised PC/LPC [Ox-PC/LPC]	0.12 [0.10 - 0.14]	0.11 [0.09 - 0.14]
Lysophosphatidylinositol [LPI]	1.31 [1.08 - 1.57]	1.33 [1.02 - 1.66]
Alkyldiacylglycerol [TG(O) [SIM]]	3.12 [2.69 - 3.65]	3.06 [2.63 - 3.63]
**Cluster no.5 (mol %)**	**16.3 %**	**15.1 %**
Diacylglycerol [DG]	88.72 [66.67 - 124.30]	84.87 [58.30 - 129.23]
Triacylglycerol [TG [NL]]	1232.4 [938.6 – 1624.2]	1299.8 [1028.1 – 1644.1]
Phosphatidylglycerol [PG]	0.49 [0.36 - 0.67]	0.51 [0.38 - 0.71]
**Cluster no.6 (mol %)**	**13.1 %**	**14.0 %**
Ubiquinone	1.00 [0.74 - 1.18]	0.77 [0.54 - 1.03]
Oxidised CE [Ox-CE]	5.45 [4.27 - 7.26]	4.93 [3.76 - 6.42]
Free cholesterol [Free C]	933.79 [828.36 - 1044.57]	856.67 [725.81 - 979.82]
Sphingomyelin [SM]	372.52 [317.91 - 428.45]	342.5 [270.27 - 400.84]
**Cluster no.7 (mol %)**	**1.8 %**	**1.9 %**
Lysophosphatidylethanolamine [LPE]	4.53 [3.76 - 5.48]	4.82 [3.65 - 6.11]
Lysophosphatidylcholine [LPC]	166.35 [136.85 - 200.61]	156.44 [117.17 - 195.57]
Phosphatidic acid [PA]	0.095 [0.083 - 0.11]	0.095 [0.074 - 0.12]
**Cluster no.8 (mol %)**	**0.01 %**	**0.02 %**
Sphingosine [Sph]	0.13 [0.12 - 0.14]	0.12 [0.11 - 0.13]
Sphingosine-1-phosphate [S1P]	0.79 [0.70 - 0.89]	0.73 [0.57 - 0.90]
Phosphatidylserine [PS]	1.04 [0.79 - 1.42]	0.26 [0.21 - 0.32]
**Cluster no.9 (mol %)**	**3.7 %**	**4.4 %**
Lysoalkenylphosphatidylethanolamine (plasmalogen) [LPE(P)]	0.18 [0.15 - 0.22]	0.17 [0.13 - 0.23]
Acylcarnitine [AC]	0.33 [0.27 - 0.41]	0.50 [0.37 - 0.67]

Free fatty acid [FFA]	389.05 [316.51 - 479.84]	306.59 [249.89 - 384.32]
Phosphatidylinositol monophosphate [PIP1]	0.052 [0.043 - 0.059]	0.047 [0.037 - 0.056]
**Cluster no.10 (mol %)**	**1.2 %**	**1.2 %**
Alkenylphosphatidylethanolamine (plasmalogen) [PE(P)]	39.42 [32.16 - 47.76]	35.79 [27.04 - 45.51]
Alkylphosphatidylethanolamine [PE(O)]	3.59 [2.86 - 4.56]	3.26 [2.45 - 4.21]
Sulfatide [ShexCer]	0.68 [0.56 - 0.81]	0.58 [0.45 - 0.71]
Alkylphosphatidylcholine [PC(O)]	63.87 [54.12 - 75.20]	63.29 [50.47 - 76.66]
GM3 ganglioside [GM3]	2.58 [2.16 - 3.05]	2.40 [1.93 - 2.89]
**Cluster no.11 (mol %)**	**41.0 %**	**41.0 %**
Cholesteryl ester [CE]	3860.76 [3423.71 - 4329.89]	3661.83 [3198.54 - 4111.50]
**Cluster no.12 (mol %)**	**0.45 %**	**0.45 %**
Alkenylphosphatidylcholine (plasmalogen) [PC(P)]	40.54 [33.26 - 48.97]	40.03 [30.82 - 49.90]
Trihexosylcermide [Hex3Cer]	1.39 [1.16 - 1.66]	1.37 [1.09 - 1.65]
Ceramide-1-phosphate [C1P]	0.005 [0.004 - 0.007]	0.005 [0.004 - 0.006]
**Cluster no.13 (mol %)**	**0.12 %**	**0.13 %**
Dihexosylceramide[Hex2Cer]	3.54 [2.88 - 4.35]	3.65 [2.83 - 4.52]
Monohexosylceramide [HexCer]	4.91 [4.06 - 5.92]	4.62 [3.77 - 5.59]
Lysoalkylphosphatidylcholine [LPC(O)]	2.51 [2.10 - 2.96]	2.24 [1.69 - 2.78]
Lysoalkenylphosphatidylcholine (plasmalogen) [LPC(P)]	0.56 [0.46 - 0.68]	0.53 [0. 39 - 0.68]
GM1 ganglioside [GM1]	0.012 [0.010 - 0.015]	0.012 [0.010 - 0.015]
**Cluster no.14 (mol %)**	**0.13 %**	**0.12 %**
Dehydrocholesterol ester [DE]	10.96 [9.18 - 13.06]	11.27 [8.52 - 14.37]

Table S5.

Comparison of the relative abundances (mol-%) of the most common serum lipid classes in lipidomics studies.

Lipid species	(1)	(2)	(3)	(4)	(5)	(6)	(7)	(8)	(9)	NFBC66 LC-MS/MS	YFS LC-MS/MS
**CE**	29.41%	78.13%	37.58%	52%	30.00%	55.50%	50.40%	36.85%	39.40%	**41.20%**	**40.90%**
**PC**	27.31%	12.09%	35.49%	29%	15.80%	9.30%	13.10%	24.62%	30.90%	**20.20%**	**21.10%**
**TG**	12.61%	3.80%	6.54%	9%	39.70%	7.60%	8.00%	13.20%	15.50%	**14.00%**	**15.20%**
**Free C**						9.60%	12.70%	10.30%		**10%**	**9.40%**
**FFA**	21.01%				3.17%			2.67%	13.00%	**4.40%**	**3.70%**
**SM**	3.99%	4.63%	2.09%	8%	3.17%	4.80%	5.00%	3.79%	8.00%	**4.00%**	**3.60%**
**LPC**	2.10%	0.82%	10.44%	1.90%	3.57%	3.70%	2.60%	1.29%	3.80%	**1.80%**	**1.70%**
**DG**	0.29%	0.06%	1.39%	0.70%	1.43%	1.90%	0.40%	0.65%	0.50%	**1.10%**	**1.10%**
**PI**	0.34%		0.11%	0.60%	0.67%	1.00%	0.70%	0.39%		**0.80%**	**0.80%**
**PC(O)**		0.27%	5.43%		0.64%	0.90%	1.30%			**0.70%**	**0.70%**
**PC(P)**	0.84%				0.47%	1.00%	0.50%			**0.40%**	**0.40%**
**PE(P)**					0.54%	4.00%	4.20%			**0.40%**	**0.40%**
**PE**	1.68%	0.19%	0.15%	0.60%	0.23%	0.40%	0.60%	5.43%	1.70%	**0.3%**	**0.3%**
**DE**					0.08%					**0.11%**	**0.10%**
**Cer(d)**	0.04%		0.14%		0.09%	0.20%	0.10%	0.15%	0.10%	**0.09%**	**0.09%**
**Others**	0.38%	0.02%	0.64%	0.11%	0.41%	0.10%	0.20%	0.68%	0.10%	**0.36%**	**0.45%**

The NFBC66 and YFS LC-MS/MS results correspond to those given in Figure 2B. The numbers in the table refer to the following studies:

(1) Eichelmann F., et al. Deep Lipidomics in Human Plasma: Cardiometabolic Disease Risk and Effect of Dietary Fat Modulation. Circulation. 2022;146:21-35

(2) Fernandez C., et al. Plasma lipid composition and risk of developing cardiovascular disease. PLoS One. 2013;8:e71846

(3) Aristizabal-Henao J. J., et al. Nontargeted lipidomics of novel human plasma reference materials: hypertriglyceridemic, diabetic, and African-American. Anal Bioanal Chem. 2020;412:7373-7380

(4) Nilsson A. K., et al. Lipid profiling of suction blister fluid: comparison of lipids in interstitial fluid and plasma. Lipids Health Dis. 2019;18:164

(5) Yap C. X., et al. Interactions between the lipidome and genetic and environmental factors in autism. Nat Med. 2023;29:936-949

(6) Huynh K., et al. High-Throughput Plasma Lipidomics: Detailed Mapping of the Associations with Cardiometabolic Risk Factors. Cell Chem Biol. 2019;26:71-84.e4

(7) Bowden J. A., et al. Harmonizing lipidomics: NIST interlaboratory comparison exercise for lipidomics using SRM 1950-Metabolites in Frozen Human Plasma. J Lipid Res. 2017;58:2275-2288

(8) Quehenberger O., et al. Lipidomics reveals a remarkable diversity of lipids in human plasma. J Lipid Res. 2010;51:3299-305

(9) Ghorasaini M., et al. Cross-Laboratory Standardization of Preclinical Lipidomics Using Differential Mobility Spectrometry and Multiple Reaction Monitoring. Anal Chem. 2021;93:16369-16378

Supplement Figures

Figure S1.

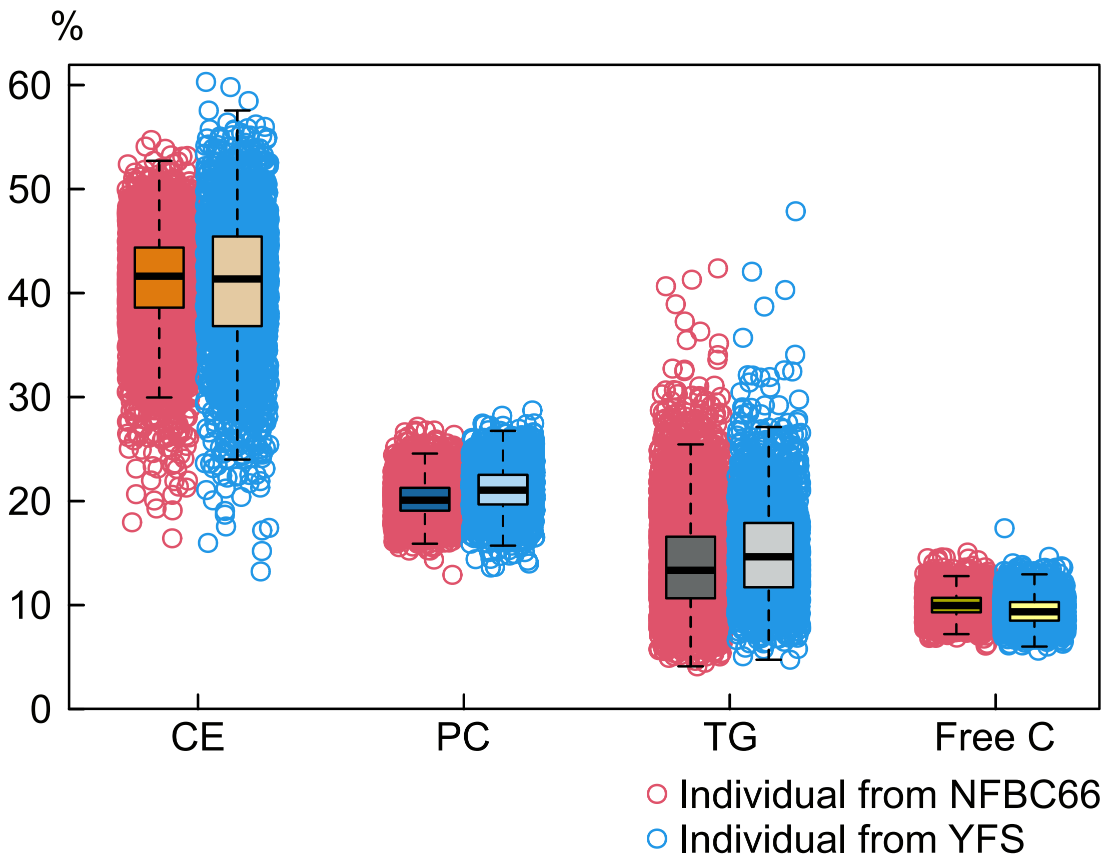

Figure S2.

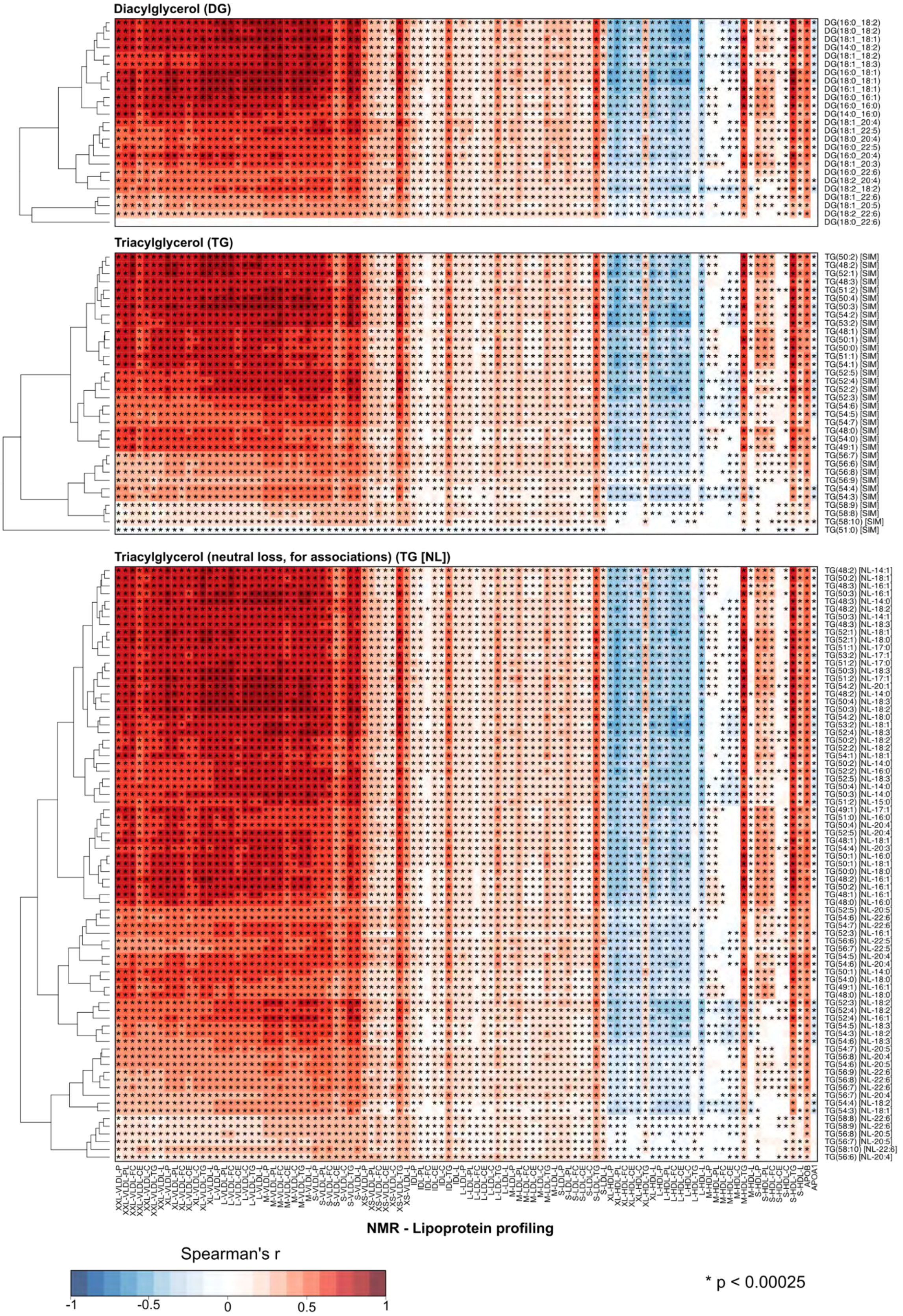
Diacylglycerol (DG)
Triacylglycerol (TG)
Triacylglycerol (neutral loss, for associations) (TG [NL])
NMR - Lipoprotein profiling
Spearman's r
-1
-0.5
0
0.5
1
* p < 0.00025

Figure S3.

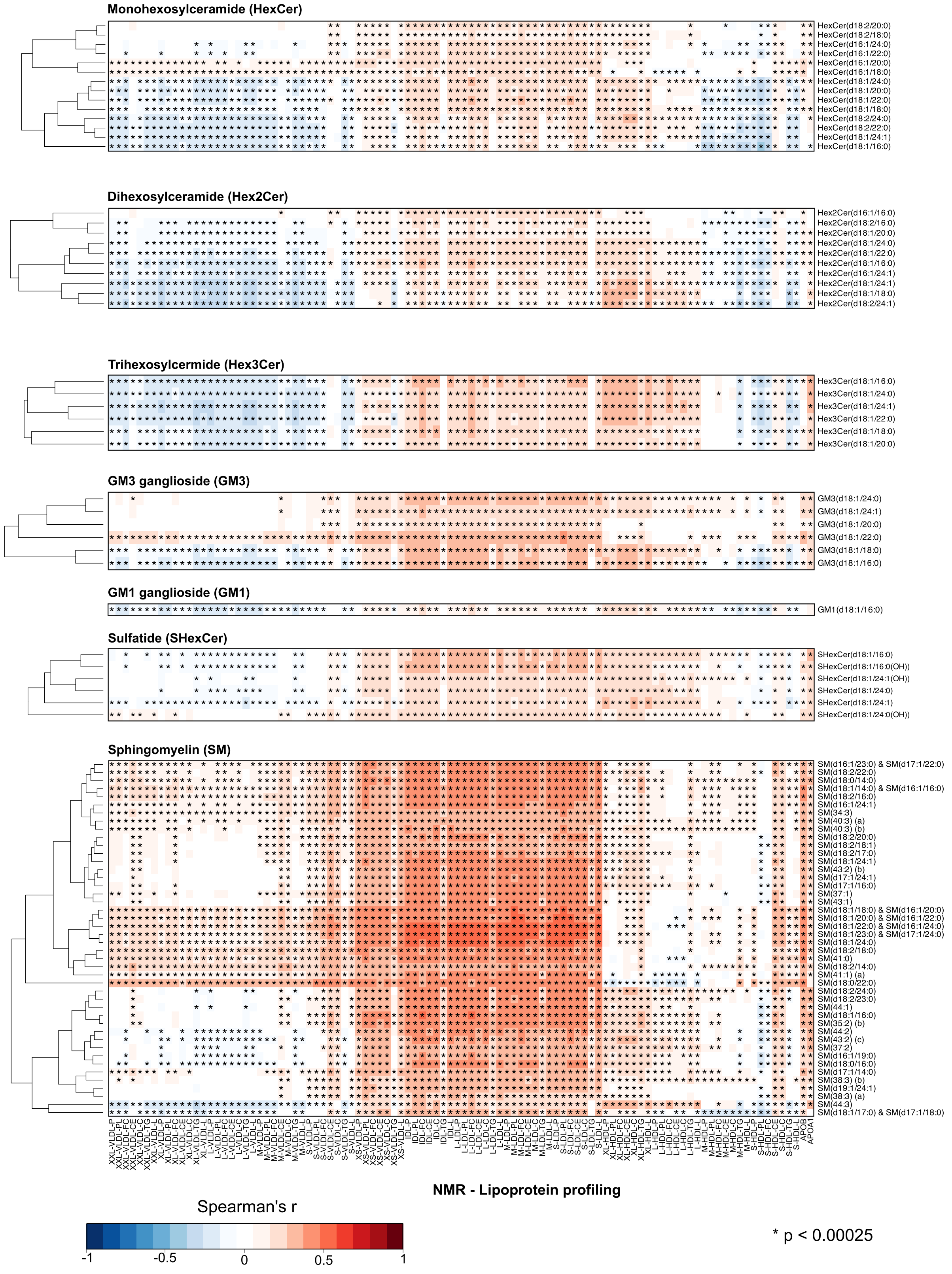
Monohexosylceramide (HexCer)
HexCer(d18:2/20:0)
HexCer(d18:2/18:0)
HexCer(d16:1/24:0)
HexCer(d16:1/22:0)
HexCer(d16:1/20:0)
HexCer(d16:1/18:0)
HexCer(d18:1/24:0)
HexCer(d18:1/20:0)
HexCer(d18:1/22:0)
HexCer(d18:1/18:0)
HexCer(d18:2/24:0)
HexCer(d18:2/22:0)
HexCer(d18:1/24:1)
HexCer(d18:1/16:0)
Dihexosylceramide (Hex2Cer)
Hex2Cer(d16:1/16:0)
Hex2Cer(d18:2/16:0)
Hex2Cer(d18:1/20:0)
Hex2Cer(d18:1/24:0)
Hex2Cer(d18:1/22:0)
Hex2Cer(d18:1/16:0)
Hex2Cer(d16:1/24:1)
Hex2Cer(d18:1/24:1)
Hex2Cer(d18:1/18:0)
Hex2Cer(d18:2/24:1)
Trihexosylcermide (Hex3Cer)
Hex3Cer(d18:1/16:0)
Hex3Cer(d18:1/24:0)
Hex3Cer(d18:1/24:1)
Hex3Cer(d18:1/22:0)
Hex3Cer(d18:1/18:0)
Hex3Cer(d18:1/20:0)
GM3 ganglioside (GM3)
GM3(d18:1/24:0)
GM3(d18:1/24:1)
GM3(d18:1/20:0)
GM3(d18:1/22:0)
GM3(d18:1/18:0)
GM3(d18:1/16:0)
GM1 ganglioside (GM1)
GM1(d18:1/16:0)
Sulfatide (SHexCer)
SHexCer(d18:1/16:0)
SHexCer(d18:1/16:0(OH))
SHexCer(d18:1/24:1(OH))
SHexCer(d18:1/24:0)
SHexCer(d18:1/24:1)
SHexCer(d18:1/24:0(OH))
Sphingomyelin (SM)
SM(d16:1/23:0) & SM(d17:1/22:0)
SM(d18:2/22:0)
SM(d18:0/14:0)
SM(d18:1/14:0) & SM(d16:1/16:0)
SM(d18:2/16:0)
SM(d16:1/24:1)
SM(34:3)
SM(40:3) (a)
SM(40:3) (b)
SM(d18:2/20:0)
SM(d18:2/18:1)
SM(d18:2/17:0)
SM(d18:1/24:1)
SM(43:2) (b)
SM(d17:1/24:1)
SM(d17:1/16:0)
SM(37:1)
SM(43:1)
SM(d18:1/18:0) & SM(d16:1/20:0)
SM(d18:1/20:0) & SM(d16:1/22:0)
SM(d18:1/22:0) & SM(d16:1/24:0)
SM(d18:1/23:0) & SM(d17:1/24:0)
SM(d18:1/24:0)
SM(d18:2/18:0)
SM(41:0)
SM(d18:2/14:0)
SM(41:1) (a)
SM(d18:0/22:0)
SM(d18:2/24:0)
SM(d18:2/23:0)
SM(44:1)
SM(d18:1/16:0)
SM(35:2) (b)
SM(44:2)
SM(43:2) (c)
SM(37:2)
SM(d16:1/19:0)
SM(d18:0/16:0)
SM(d17:1/14:0)
SM(38:3) (b)
SM(d19:1/24:1)
SM(38:3) (a)
SM(44:3)
SM(d18:1/17:0) & SM(d17:1/18:0)
NMR - Lipoprotein profiling
Spearman's r
-1
-0.5
0
0.5
1
* p < 0.00025

Figure S4.

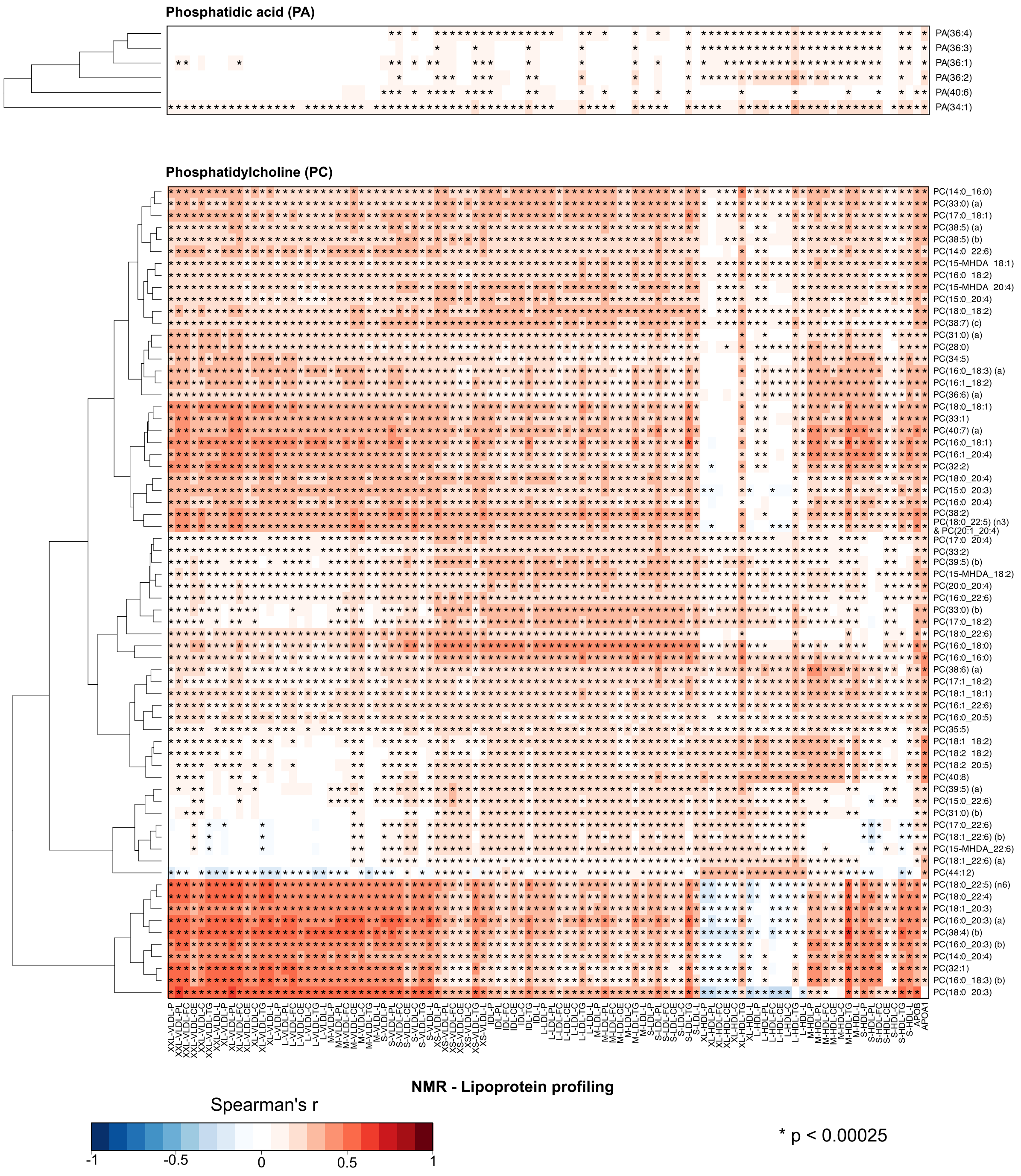
Phosphatidic acid (PA)
Phosphatidylcholine (PC)
NMR - Lipoprotein profiling
Spearman's r
-1
-0.5
0
0.5
1
* p < 0.00025

Figure S5.

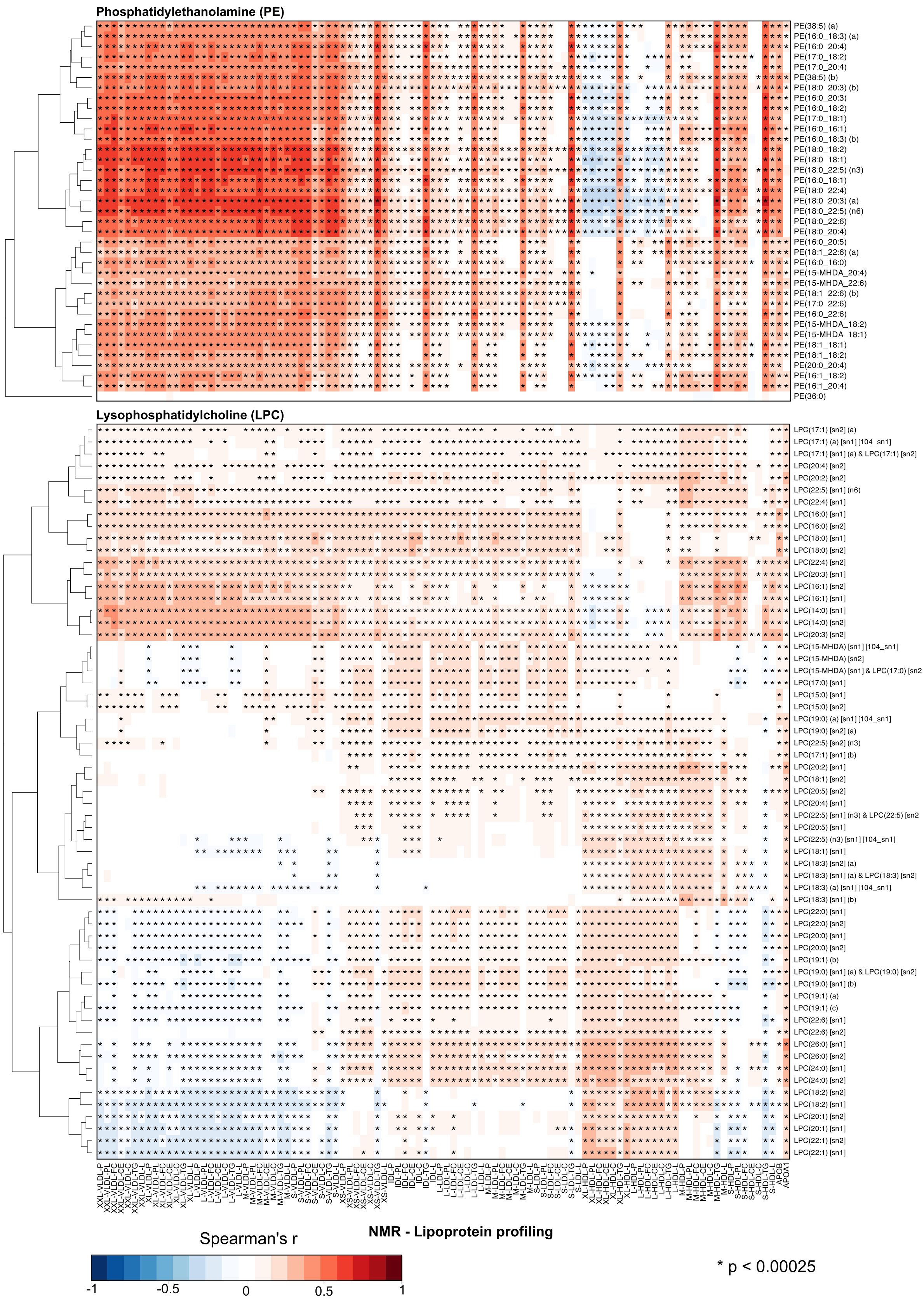

Phosphatidylethanolamine (PE)
Lysophosphatidylcholine (LPC)
NMR - Lipoprotein profiling
Spearman's r
-1
-0.5
0
0.5
1
* p < 0.00025

Figure S6.

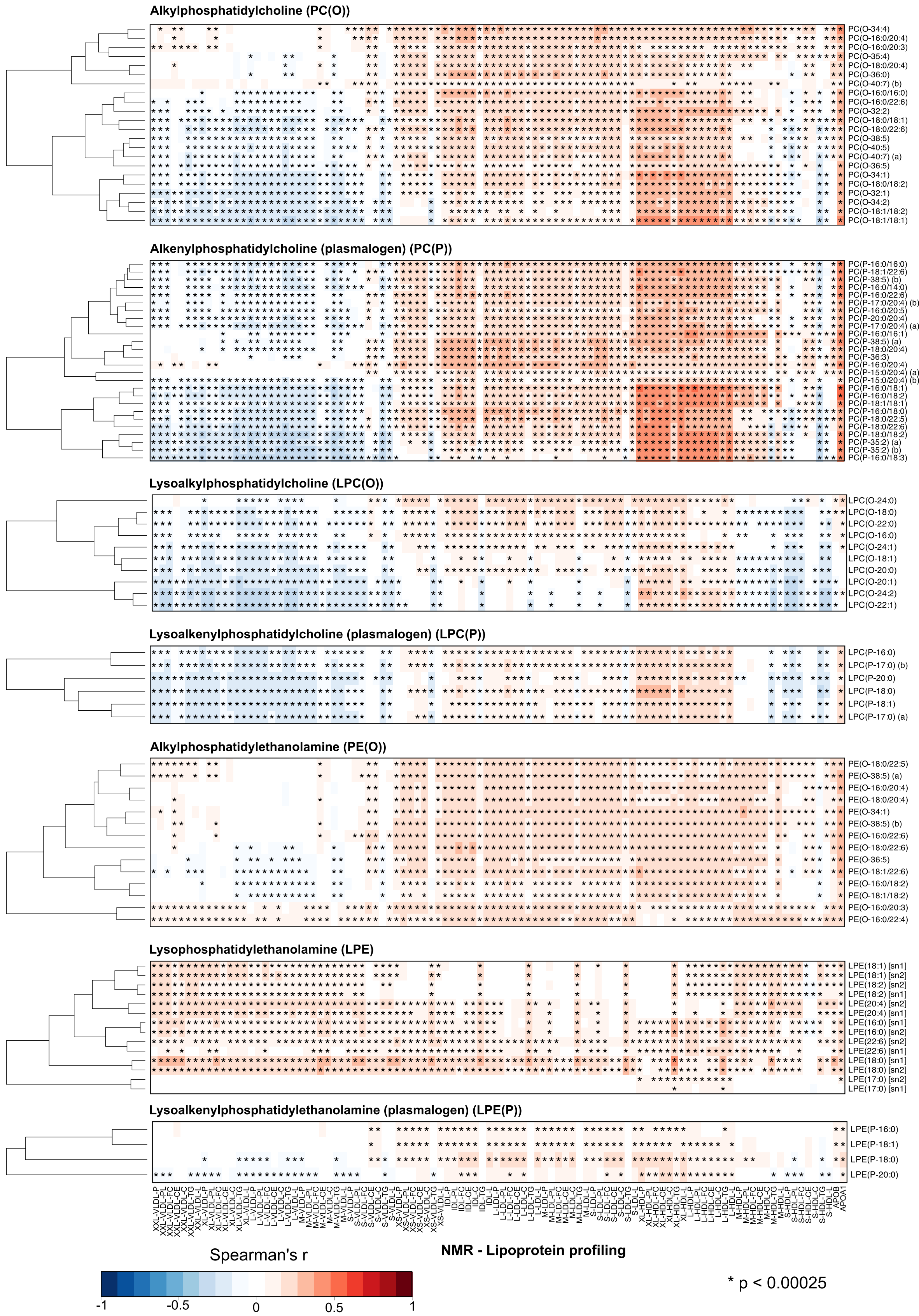
Alkylphosphatidylcholine (PC(O))
Alkenylphosphatidylcholine (plasmalogen) (PC(P))
Lysoalkylphosphatidylcholine (LPC(O))
Lysoalkenylphosphatidylcholine (plasmalogen) (LPC(P))
Alkylphosphatidylethanolamine (PE(O))
Lysophosphatidylethanolamine (LPE)
Lysoalkenylphosphatidylethanolamine (plasmalogen) (LPE(P))
Spearman's r
-1
-0.5
0
0.5
1
NMR - Lipoprotein profiling
* p < 0.00025

Figure S7.

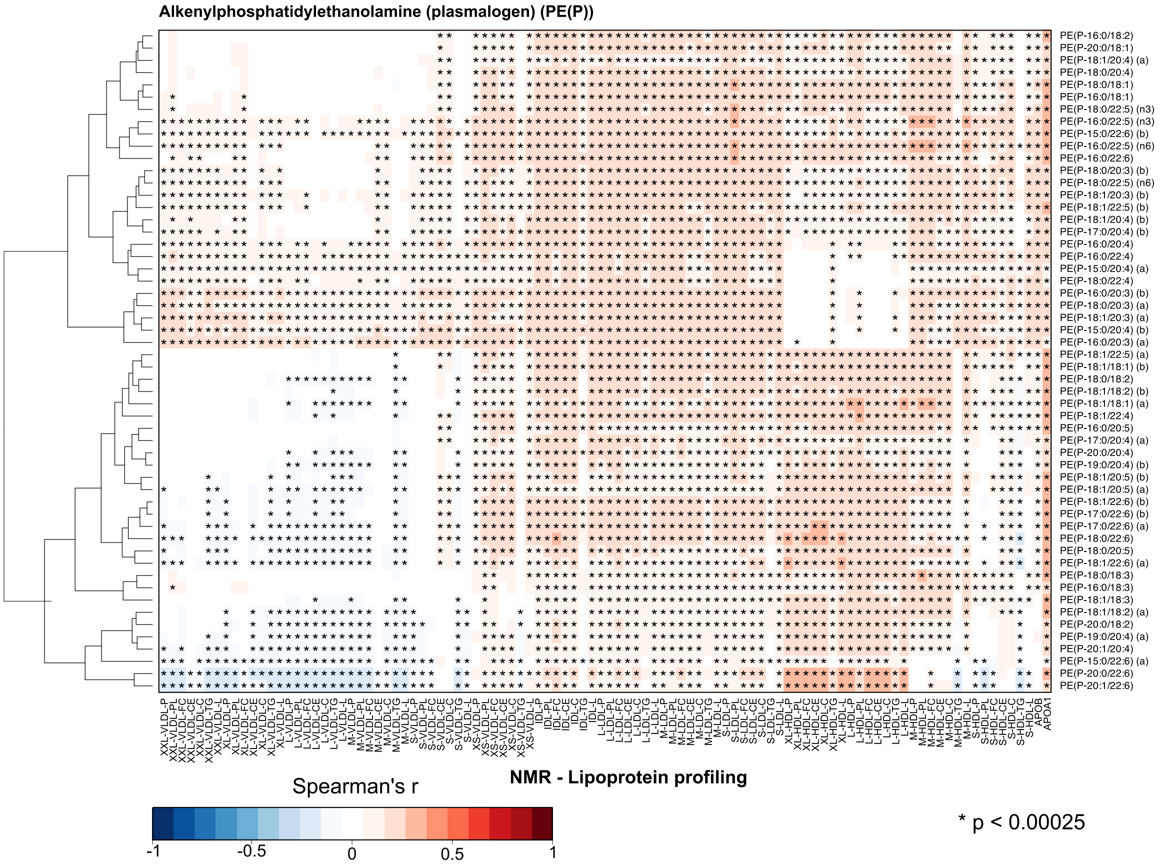
Alkenylphosphatidylethanolamine (plasmalogen) (PE(P))
PE(P-16:0/18:2)
PE(P-20:0/18:1)
PE(P-18:1/20:4) (a)
PE(P-18:0/20:4)
PE(P-18:0/18:1)
PE(P-16:0/18:1)
PE(P-18:0/22:5) (n3)
PE(P-16:0/22:5) (n3)
PE(P-15:0/22:6) (b)
PE(P-16:0/22:5) (n6)
PE(P-16:0/22:6)
PE(P-18:0/20:3) (b)
PE(P-18:0/22:5) (n6)
PE(P-18:1/20:3) (b)
PE(P-18:1/22:5) (b)
PE(P-18:1/20:4) (b)
PE(P-17:0/20:4) (b)
PE(P-16:0/20:4)
PE(P-16:0/22:4)
PE(P-15:0/20:4) (a)
PE(P-18:0/22:4)
PE(P-16:0/20:3) (b)
PE(P-18:0/20:3) (a)
PE(P-18:1/20:3) (a)
PE(P-15:0/20:4) (b)
PE(P-16:0/20:3) (a)
PE(P-18:1/22:5) (a)
PE(P-18:1/18:1) (b)
PE(P-18:0/18:2)
PE(P-18:1/18:2) (b)
PE(P-18:1/18:1) (a)
PE(P-18:1/22:4)
PE(P-16:0/20:5)
PE(P-17:0/20:4) (a)
PE(P-20:0/20:4)
PE(P-19:0/20:4) (b)
PE(P-18:1/20:5) (b)
PE(P-18:1/20:5) (a)
PE(P-18:1/22:6) (b)
PE(P-17:0/22:6) (b)
PE(P-17:0/22:6) (a)
PE(P-18:0/22:6)
PE(P-18:0/20:5)
PE(P-18:1/22:6) (a)
PE(P-18:0/18:3)
PE(P-16:0/18:3)
PE(P-18:1/18:3)
PE(P-18:1/18:2) (a)
PE(P-20:0/18:2)
PE(P-19:0/20:4) (a)
PE(P-20:1/20:4)
PE(P-15:0/22:6) (a)
PE(P-20:0/22:6)
PE(P-20:1/22:6)
XXL-VLDL-P
XXL-VLDL-PL
XXL-VLDL-FC
XXL-VLDL-CE
XXL-VLDL-C
XXL-VLDL-TG
XXL-VLDL-L
XL-VLDL-P
XL-VLDL-PL
XL-VLDL-FC
XL-VLDL-CE
XL-VLDL-C
XL-VLDL-TG
XL-VLDL-L
L-VLDL-P
L-VLDL-PL
L-VLDL-FC
L-VLDL-CE
L-VLDL-C
L-VLDL-TG
L-VLDL-L
M-VLDL-P
M-VLDL-PL
M-VLDL-FC
M-VLDL-CE
M-VLDL-C
M-VLDL-TG
M-VLDL-L
S-VLDL-P
S-VLDL-PL
S-VLDL-FC
S-VLDL-CE
S-VLDL-C
S-VLDL-TG
S-VLDL-L
XS-VLDL-P
XS-VLDL-PL
XS-VLDL-FC
XS-VLDL-CE
XS-VLDL-C
XS-VLDL-TG
XS-VLDL-L
IDL-P
IDL-PL
IDL-FC
IDL-CE
IDL-C
IDL-TG
IDL-L
L-LDL-P
L-LDL-PL
L-LDL-FC
L-LDL-CE
L-LDL-C
L-LDL-TG
L-LDL-L
M-LDL-P
M-LDL-PL
M-LDL-FC
M-LDL-CE
M-LDL-C
M-LDL-TG
M-LDL-L
S-LDL-P
S-LDL-PL
S-LDL-FC
S-LDL-CE
S-LDL-C
S-LDL-TG
S-LDL-L
XL-HDL-P
XL-HDL-PL
XL-HDL-FC
XL-HDL-CE
XL-HDL-C
XL-HDL-TG
XL-HDL-L
L-HDL-P
L-HDL-PL
L-HDL-FC
L-HDL-CE
L-HDL-C
L-HDL-TG
L-HDL-L
M-HDL-P
M-HDL-PL
M-HDL-FC
M-HDL-CE
M-HDL-C
M-HDL-TG
M-HDL-L
S-HDL-P
S-HDL-PL
S-HDL-FC
S-HDL-CE
S-HDL-C
S-HDL-TG
S-HDL-L
APOB
APOA1
NMR - Lipoprotein profiling
Spearman's r
-1
-0.5
0
0.5
1
* p < 0.00025

Figure S8.

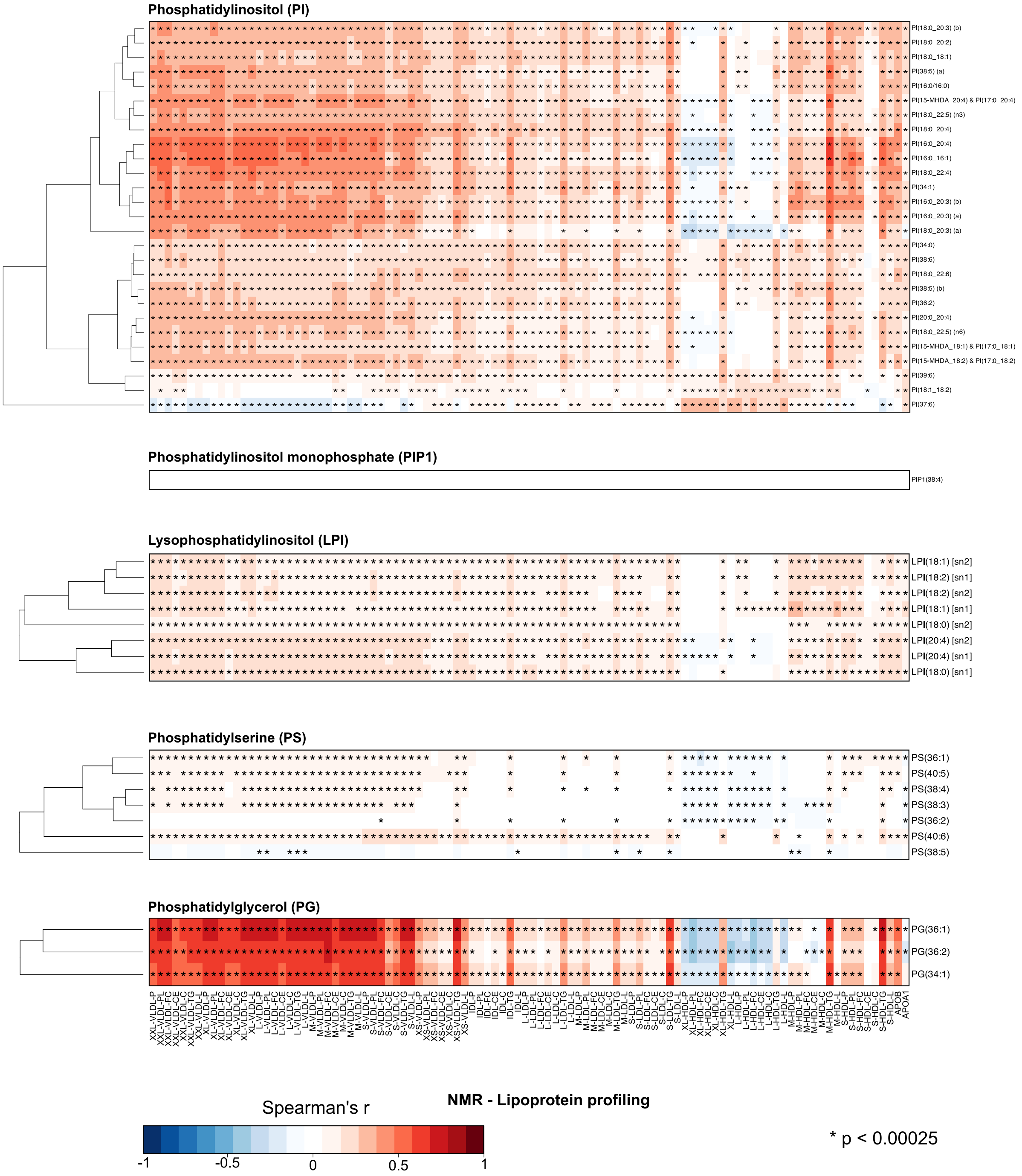
Phosphatidylinositol (PI)
Phosphatidylinositol monophosphate (PIP1)
Lysophosphatidylinositol (LPI)
Phosphatidylserine (PS)
Phosphatidylglycerol (PG)
LPI(18:1) [sn2]
LPI(18:2) [sn1]
LPI(18:2) [sn2]
LPI(18:1) [sn1]
LPI(18:0) [sn2]
LPI(20:4) [sn2]
LPI(20:4) [sn1]
LPI(18:0) [sn1]
PS(36:1)
PS(40:5)
PS(38:4)
PS(38:3)
PS(36:2)
PS(40:6)
PS(38:5)
PG(36:1)
PG(36:2)
PG(34:1)
Spearman's r
-1
-0.5
0
0.5
1
NMR - Lipoprotein profiling
* p < 0.00025

Figure S9.

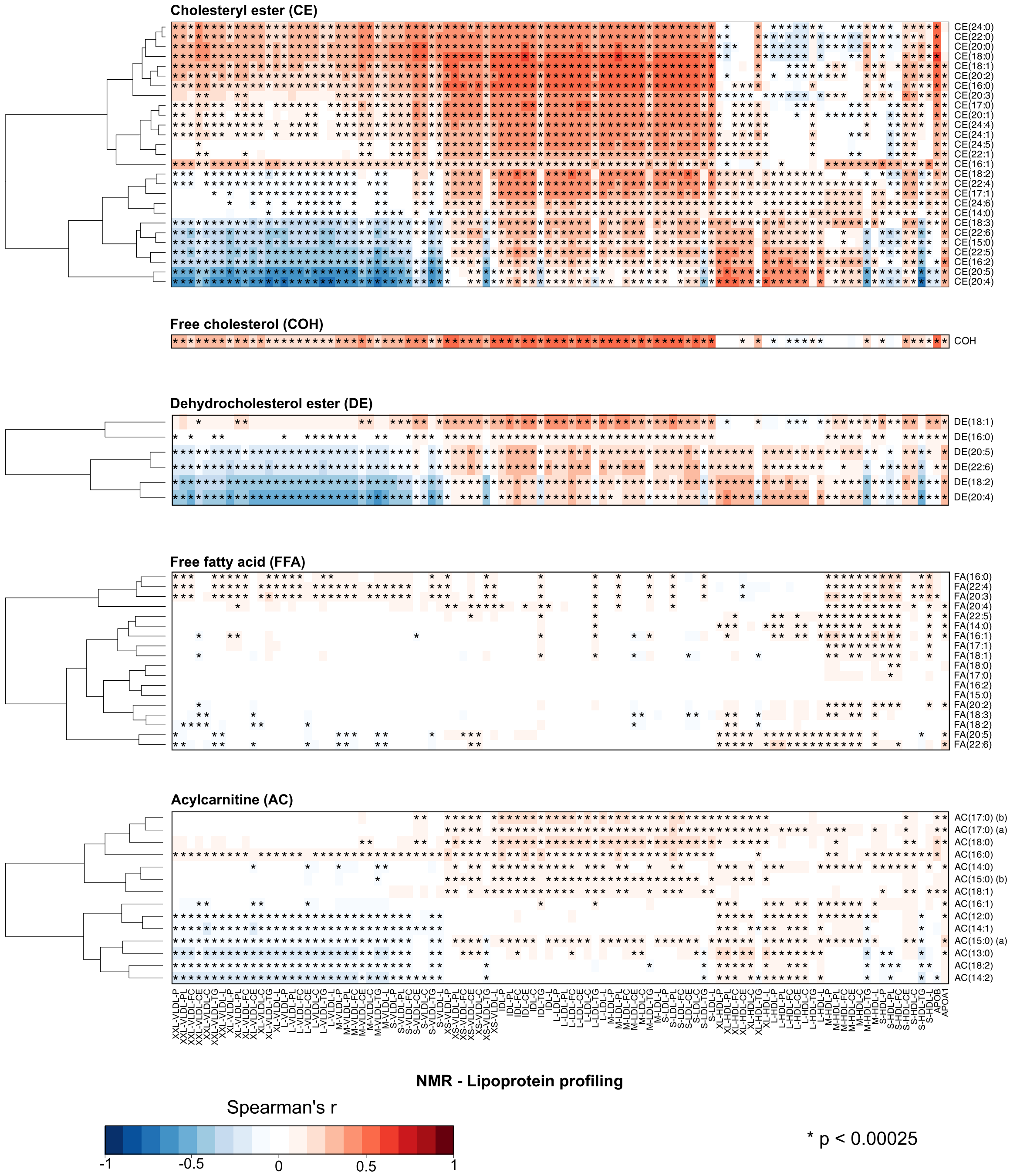
Cholesteryl ester (CE)
CE(24:0)
CE(22:0)
CE(20:0)
CE(18:0)
CE(18:1)
CE(20:2)
CE(16:0)
CE(20:3)
CE(17:0)
CE(20:1)
CE(24:4)
CE(24:1)
CE(24:5)
CE(22:1)
CE(16:1)
CE(18:2)
CE(22:4)
CE(17:1)
CE(24:6)
CE(14:0)
CE(18:3)
CE(22:6)
CE(15:0)
CE(22:5)
CE(16:2)
CE(20:5)
CE(20:4)
Free cholesterol (COH)
COH
Dehydrocholesterol ester (DE)
DE(18:1)
DE(16:0)
DE(20:5)
DE(22:6)
DE(18:2)
DE(20:4)
Free fatty acid (FFA)
FA(16:0)
FA(22:4)
FA(20:3)
FA(20:4)
FA(22:5)
FA(14:0)
FA(16:1)
FA(17:1)
FA(18:1)
FA(18:0)
FA(17:0)
FA(16:2)
FA(15:0)
FA(20:2)
FA(18:3)
FA(18:2)
FA(20:5)
FA(22:6)
Acylcarnitine (AC)
AC(17:0) (b)
AC(17:0) (a)
AC(18:0)
AC(16:0)
AC(14:0)
AC(15:0) (b)
AC(18:1)
AC(16:1)
AC(12:0)
AC(14:1)
AC(15:0) (a)
AC(13:0)
AC(18:2)
AC(14:2)
NMR - Lipoprotein profiling
Spearman's r
-1
-0.5
0
0.5
1
* p < 0.00025

Figure S10.

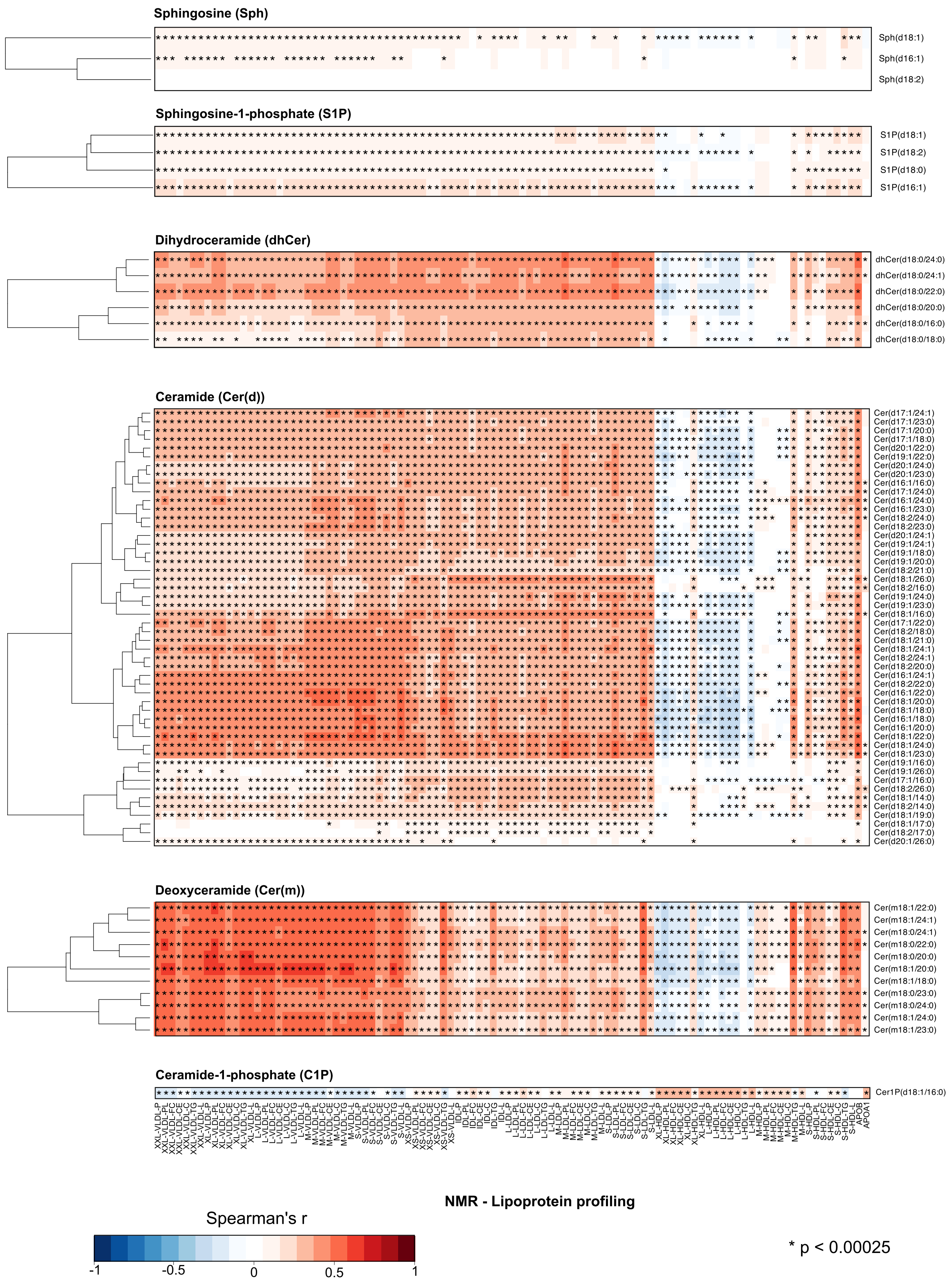

Sphingosine (Sph)
Sph(d18:1)
Sph(d16:1)
Sph(d18:2)
Sphingosine-1-phosphate (S1P)
S1P(d18:1)
S1P(d18:2)
S1P(d18:0)
S1P(d16:1)
Dihydroceramide (dhCer)
dhCer(d18:0/24:0)
dhCer(d18:0/24:1)
dhCer(d18:0/22:0)
dhCer(d18:0/20:0)
dhCer(d18:0/16:0)
dhCer(d18:0/18:0)
Ceramide (Cer(d))
Deoxyceramide (Cer(m))
Cer(m18:1/22:0)
Cer(m18:1/24:1)
Cer(m18:0/24:1)
Cer(m18:0/22:0)
Cer(m18:0/20:0)
Cer(m18:1/20:0)
Cer(m18:1/18:0)
Cer(m18:0/23:0)
Cer(m18:0/24:0)
Cer(m18:1/24:0)
Cer(m18:1/23:0)
Ceramide-1-phosphate (C1P)
Cer1P(d18:1/16:0)
NMR - Lipoprotein profiling
Spearman's r
-1
-0.5
0
0.5
1
* p < 0.00025

Figure S11.

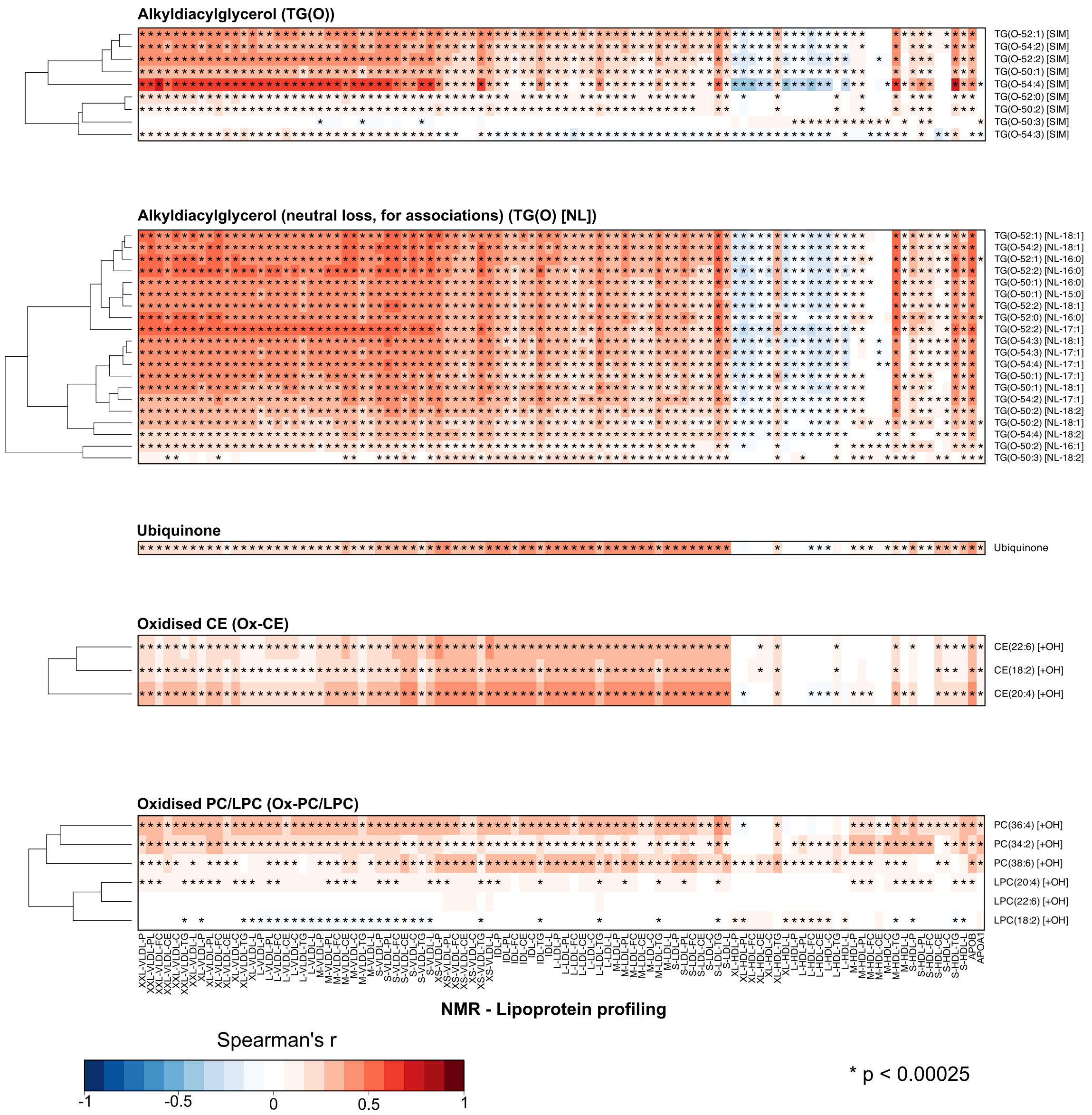
Alkyldiacylglycerol (TG(O))
TG(O-52:1) [SIM]
TG(O-54:2) [SIM]
TG(O-52:2) [SIM]
TG(O-50:1) [SIM]
TG(O-54:4) [SIM]
TG(O-52:0) [SIM]
TG(O-50:2) [SIM]
TG(O-50:3) [SIM]
TG(O-54:3) [SIM]
Alkyldiacylglycerol (neutral loss, for associations) (TG(O) [NL])
TG(O-52:1) [NL-18:1]
TG(O-54:2) [NL-18:1]
TG(O-52:1) [NL-16:0]
TG(O-52:2) [NL-16:0]
TG(O-50:1) [NL-16:0]
TG(O-50:1) [NL-15:0]
TG(O-52:2) [NL-18:1]
TG(O-52:0) [NL-16:0]
TG(O-52:2) [NL-17:1]
TG(O-54:3) [NL-18:1]
TG(O-54:3) [NL-17:1]
TG(O-54:4) [NL-17:1]
TG(O-50:1) [NL-17:1]
TG(O-50:1) [NL-18:1]
TG(O-54:2) [NL-17:1]
TG(O-50:2) [NL-18:2]
TG(O-50:2) [NL-18:1]
TG(O-54:4) [NL-18:2]
TG(O-50:2) [NL-16:1]
TG(O-50:3) [NL-18:2]
Ubiquinone
Ubiquinone
Oxidised CE (Ox-CE)
CE(22:6) [+OH]
CE(18:2) [+OH]
CE(20:4) [+OH]
Oxidised PC/LPC (Ox-PC/LPC)
PC(36:4) [+OH]
PC(34:2) [+OH]
PC(38:6) [+OH]
LPC(20:4) [+OH]
LPC(22:6) [+OH]
LPC(18:2) [+OH]
NMR - Lipoprotein profiling
Spearman's r
-1
-0.5
0
0.5
1
* p < 0.00025

Figure S12.

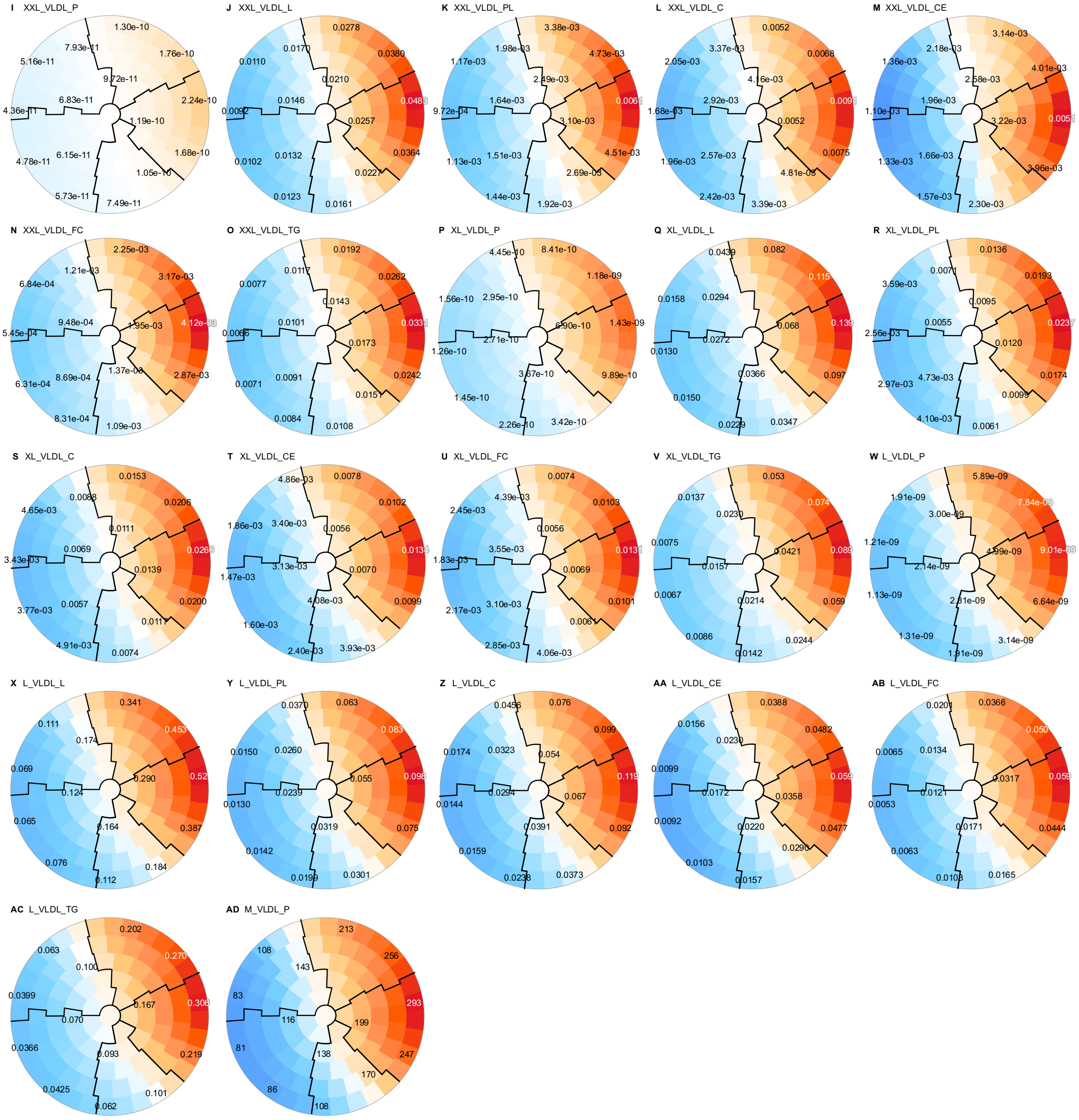
I XXL_VLDL_P
J XXL_VLDL_L
K XXL_VLDL_PL
L XXL_VLDL_C
M XXL_VLDL_CE
N XXL_VLDL_FC
O XXL_VLDL_TG
P XL_VLDL_P
Q XL_VLDL_L
R XL_VLDL_PL
S XL_VLDL_C
T XL_VLDL_CE
U XL_VLDL_FC
V XL_VLDL_TG
W L_VLDL_P
X L_VLDL_L
Y L_VLDL_PL
Z L_VLDL_C
AA L_VLDL_CE
AB L_VLDL_FC
AC L_VLDL_TG
AD M_VLDL_P

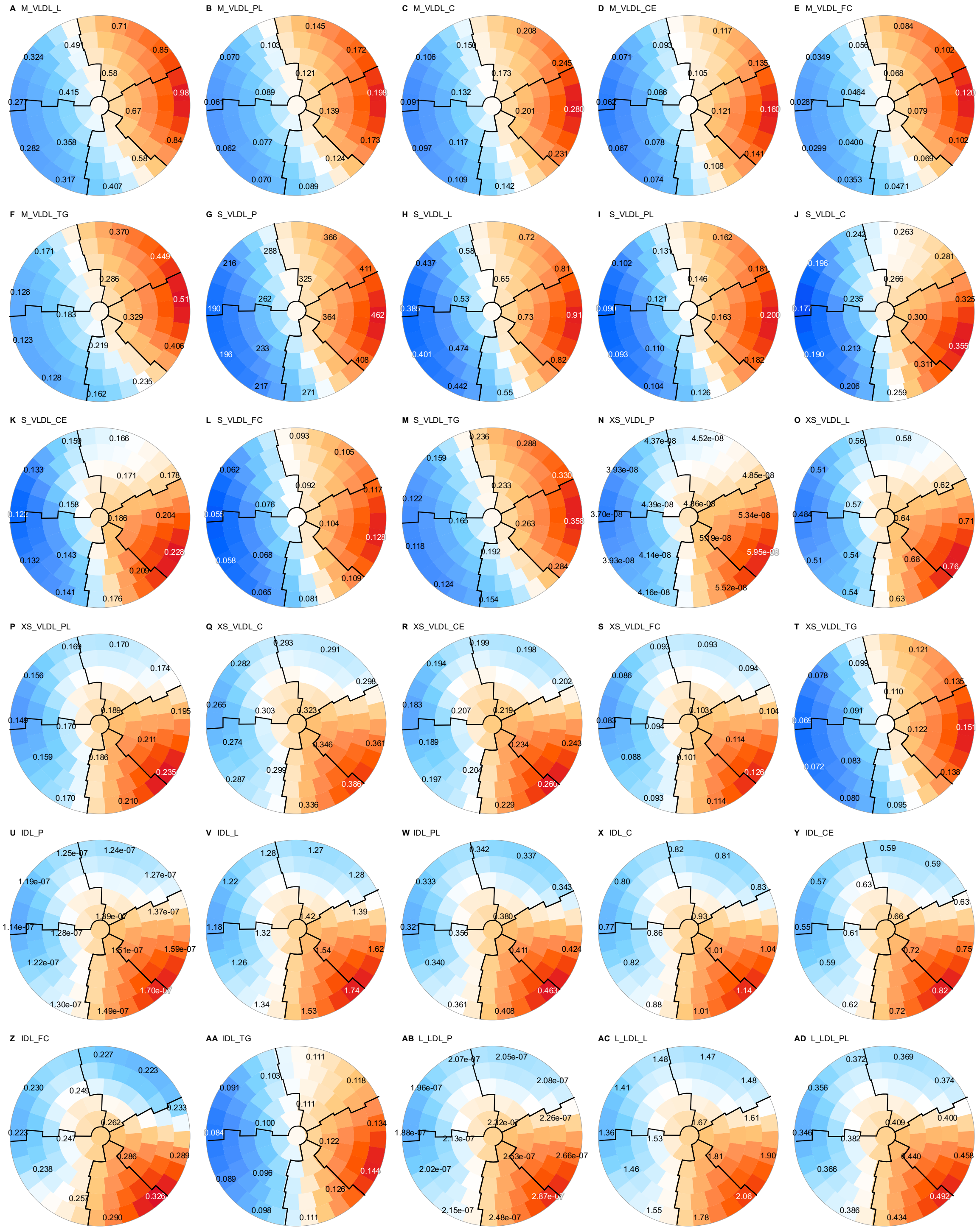
A M_VLDL_L
0.71
0.49
0.85
0.324
0.58
0.98
0.415
0.277
0.67
0.358
0.84
0.282
0.58
0.317
0.407
B M_VLDL_PL
0.145
0.103
0.172
0.070
0.121
0.198
0.089
0.061
0.139
0.077
0.173
0.062
0.124
0.070
0.089
C M_VLDL_C
0.208
0.150
0.245
0.106
0.173
0.280
0.132
0.091
0.201
0.117
0.231
0.097
0.109
0.142
D M_VLDL_CE
0.117
0.093
0.135
0.071
0.105
0.160
0.086
0.062
0.121
0.078
0.141
0.067
0.108
0.074
E M_VLDL_FC
0.084
0.056
0.102
0.0349
0.068
0.120
0.0464
0.028
0.079
0.0400
0.102
0.0299
0.069
0.0353
0.0471
F M_VLDL_TG
0.370
0.171
0.449
0.286
0.128
0.51
0.183
0.329
0.123
0.219
0.406
0.128
0.235
0.162
G S_VLDL_P
366
288
411
216
325
262
190
462
364
233
196
408
217
271
H S_VLDL_L
0.72
0.58
0.81
0.437
0.65
0.53
0.385
0.91
0.73
0.474
0.401
0.82
0.442
0.55
I S_VLDL_PL
0.162
0.131
0.181
0.102
0.146
0.121
0.090
0.200
0.163
0.110
0.093
0.182
0.104
0.126
J S_VLDL_C
0.263
0.242
0.281
0.196
0.266
0.235
0.177
0.325
0.300
0.213
0.190
0.355
0.311
0.206
0.259
K S_VLDL_CE
0.166
0.159
0.133
0.171
0.178
0.158
0.122
0.186
0.204
0.143
0.132
0.228
0.209
0.141
0.176
L S_VLDL_FC
0.093
0.105
0.062
0.092
0.117
0.076
0.055
0.104
0.128
0.068
0.058
0.109
0.065
0.081
M S_VLDL_TG
0.236
0.288
0.159
0.233
0.330
0.122
0.165
0.263
0.358
0.118
0.192
0.284
0.124
0.154
N XS_VLDL_P
4.37e-08
4.52e-08
3.93e-08
4.85e-08
4.39e-08
4.86e-08
3.70e-08
5.34e-08
5.19e-08
3.93e-08
4.14e-08
5.95e-08
4.16e-08
5.52e-08
O XS_VLDL_L
0.56
0.58
0.51
0.62
0.57
0.484
0.64
0.71
0.51
0.54
0.68
0.76
0.54
0.63
P XS_VLDL_PL
0.170
0.169
0.156
0.174
0.189
0.195
0.149
0.170
0.211
0.159
0.186
0.235
0.170
0.210
Q XS_VLDL_C
0.293
0.291
0.282
0.298
0.265
0.303
0.323
0.274
0.346
0.361
0.299
0.287
0.386
0.336
R XS_VLDL_CE
0.199
0.198
0.194
0.202
0.183
0.207
0.219
0.189
0.234
0.243
0.204
0.197
0.260
0.229
S XS_VLDL_FC
0.093
0.093
0.086
0.094
0.083
0.103
0.104
0.094
0.114
0.088
0.101
0.126
0.093
0.114
T XS_VLDL_TG
0.121
0.099
0.078
0.135
0.110
0.091
0.069
0.151
0.122
0.072
0.083
0.138
0.080
0.095
U IDL_P
1.25e-07
1.24e-07
1.19e-07
1.27e-07
1.39e-07
1.37e-07
1.14e-07
1.28e-07
1.51e-07
1.59e-07
1.22e-07
1.70e-07
1.30e-07
1.49e-07
V IDL_L
1.28
1.27
1.22
1.28
1.42
1.39
1.18
1.32
1.54
1.62
1.26
1.74
1.34
1.53
W IDL_PL
0.342
0.337
0.333
0.343
0.380
0.321
0.356
0.411
0.424
0.340
0.463
0.361
0.408
X IDL_C
0.82
0.81
0.80
0.83
0.93
0.77
0.86
1.01
1.04
0.82
1.14
0.88
1.01
Y IDL_CE
0.59
0.59
0.57
0.63
0.63
0.66
0.55
0.61
0.72
0.75
0.59
0.82
0.62
0.72
Z IDL_FC
0.227
0.223
0.230
0.249
0.233
0.262
0.223
0.247
0.286
0.289
0.238
0.257
0.326
0.290
AA IDL_TG
0.111
0.103
0.118
0.091
0.111
0.100
0.134
0.084
0.122
0.096
0.144
0.089
0.126
0.098
0.111
AB L_LDL_P
2.07e-07
2.05e-07
1.96e-07
2.08e-07
2.32e-07
2.26e-07
1.88e-07
2.13e-07
2.63e-07
2.66e-07
2.02e-07
2.87e-07
2.15e-07
2.48e-07
AC L_LDL_L
1.48
1.47
1.41
1.48
1.67
1.61
1.36
1.53
1.81
1.90
1.46
2.06
1.55
1.78
AD L_LDL_PL
0.372
0.369
0.356
0.374
0.409
0.400
0.346
0.382
0.440
0.458
0.366
0.492
0.386
0.434

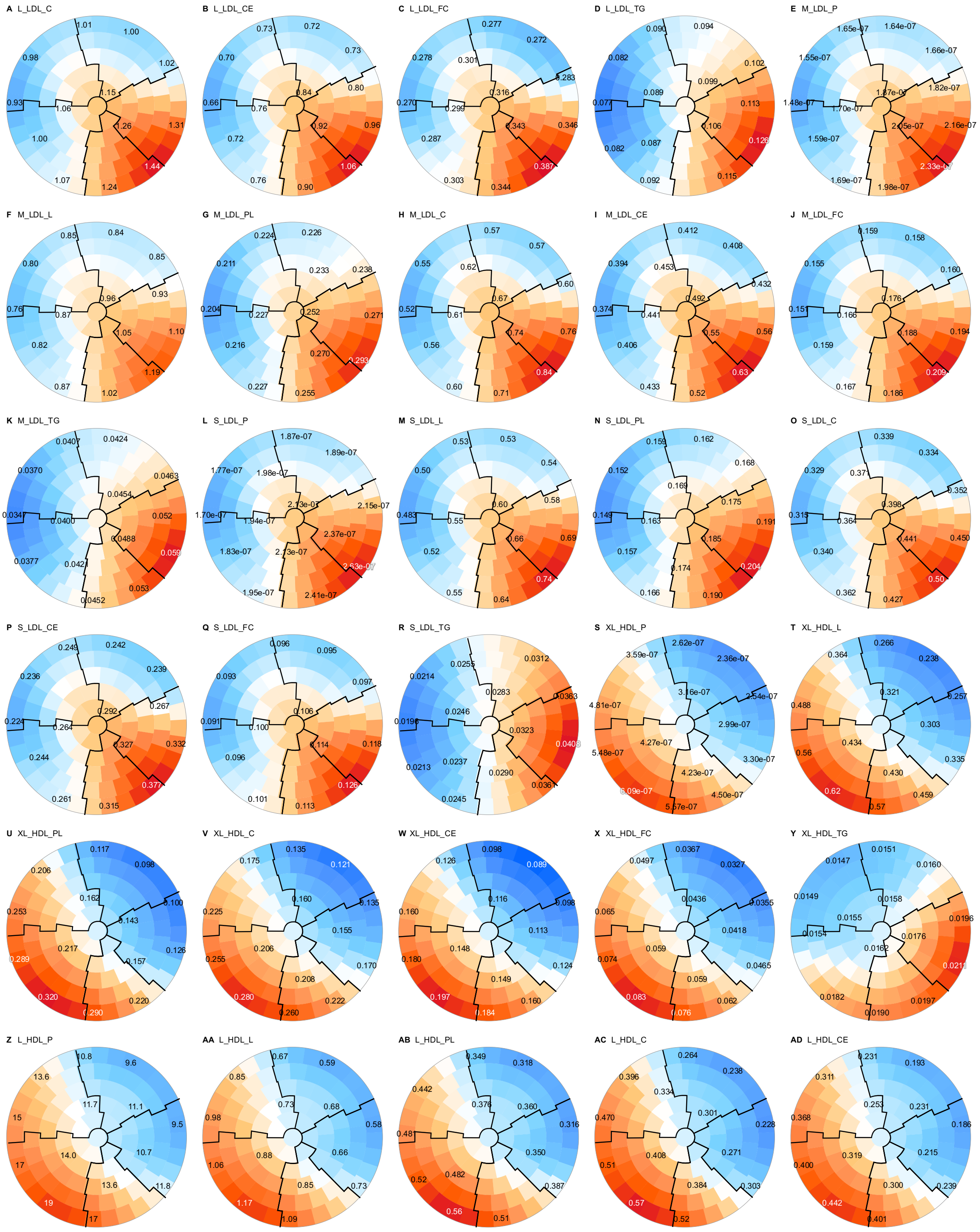
A L_LDL_C
B L_LDL_CE
C L_LDL_FC
D L_LDL_TG
E M_LDL_P
F M_LDL_L
G M_LDL_PL
H M_LDL_C
I M_LDL_CE
J M_LDL_FC
K M_LDL_TG
L S_LDL_P
M S_LDL_L
N S_LDL_PL
O S_LDL_C
P S_LDL_CE
Q S_LDL_FC
R S_LDL_TG
S XL_HDL_P
T XL_HDL_L
U XL_HDL_PL
V XL_HDL_C
W XL_HDL_CE
X XL_HDL_FC
Y XL_HDL_TG
Z L_HDL_P
AA L_HDL_L
AB L_HDL_PL
AC L_HDL_C
AD L_HDL_CE

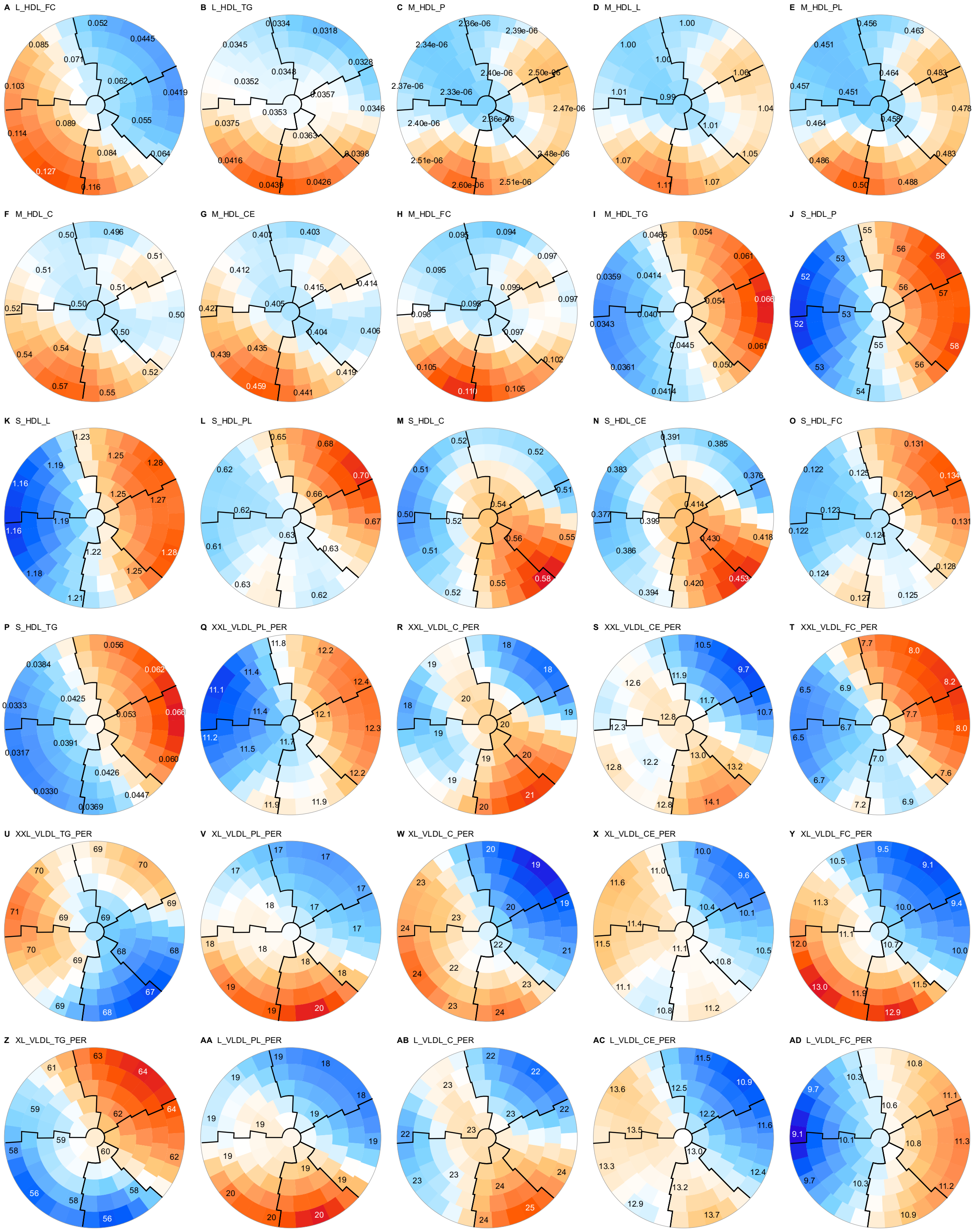
A L_HDL_FC
B L_HDL_TG
C M_HDL_P
D M_HDL_L
E M_HDL_PL
F M_HDL_C
G M_HDL_CE
H M_HDL_FC
I M_HDL_TG
J S_HDL_P
K S_HDL_L
L S_HDL_PL
M S_HDL_C
N S_HDL_CE
O S_HDL_FC
P S_HDL_TG
Q XXL_VLDL_PL_PER
R XXL_VLDL_C_PER
S XXL_VLDL_CE_PER
T XXL_VLDL_FC_PER
U XXL_VLDL_TG_PER
V XL_VLDL_PL_PER
W XL_VLDL_C_PER
X XL_VLDL_CE_PER
Y XL_VLDL_FC_PER
Z XL_VLDL_TG_PER
AA L_VLDL_PL_PER
AB L_VLDL_C_PER
AC L_VLDL_CE_PER
AD L_VLDL_FC_PER

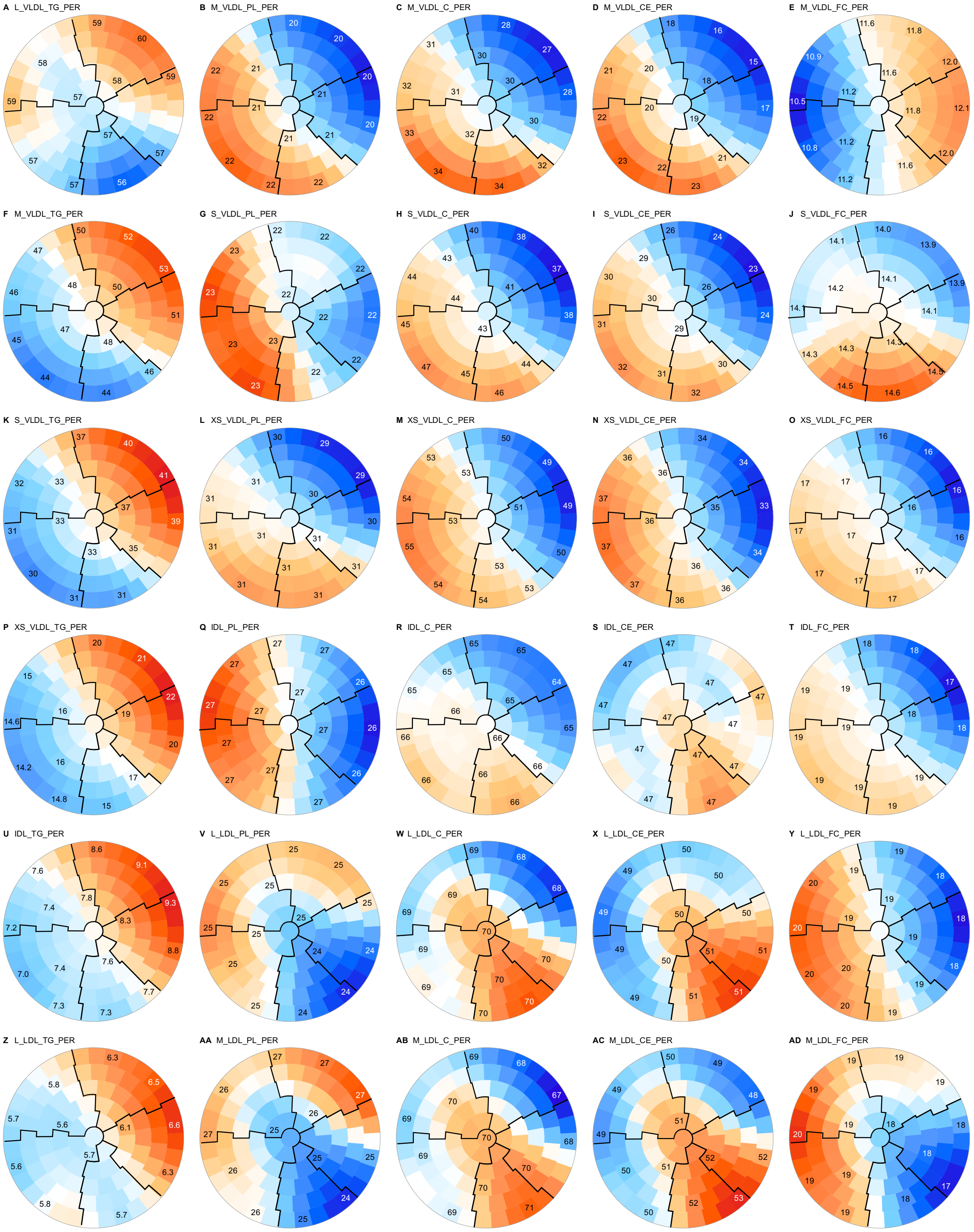
A L_VLDL_TG_PER
B M_VLDL_PL_PER
C M_VLDL_C_PER
D M_VLDL_CE_PER
E M_VLDL_FC_PER
F M_VLDL_TG_PER
G S_VLDL_PL_PER
H S_VLDL_C_PER
I S_VLDL_CE_PER
J S_VLDL_FC_PER
K S_VLDL_TG_PER
L XS_VLDL_PL_PER
M XS_VLDL_C_PER
N XS_VLDL_CE_PER
O XS_VLDL_FC_PER
P XS_VLDL_TG_PER
Q IDL_PL_PER
R IDL_C_PER
S IDL_CE_PER
T IDL_FC_PER
U IDL_TG_PER
V L_LDL_PL_PER
W L_LDL_C_PER
X L_LDL_CE_PER
Y L_LDL_FC_PER
Z L_LDL_TG_PER
AA M_LDL_PL_PER
AB M_LDL_C_PER
AC M_LDL_CE_PER
AD M_LDL_FC_PER

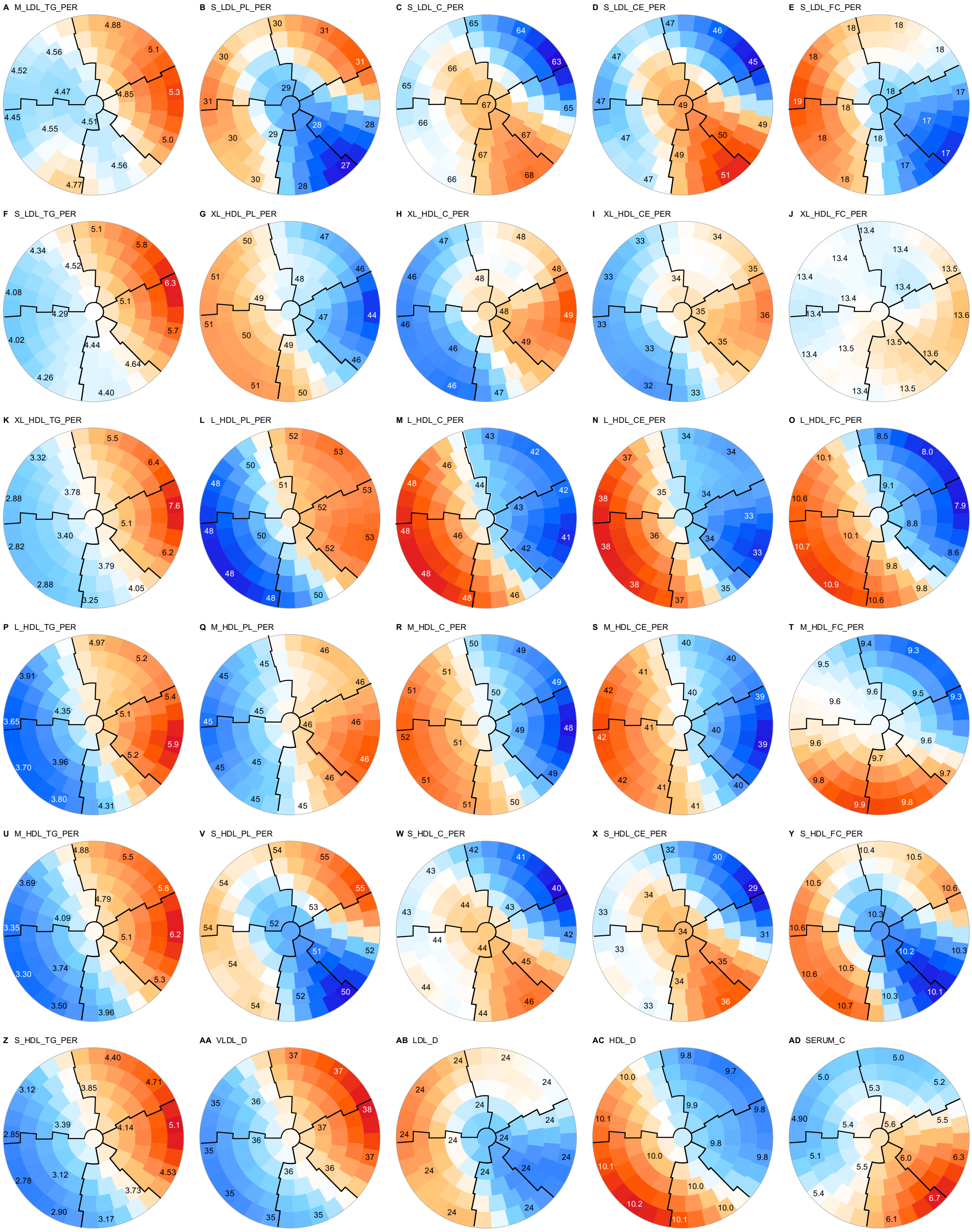
A M_LDL_TG_PER
B S_LDL_PL_PER
C S_LDL_C_PER
D S_LDL_CE_PER
E S_LDL_FC_PER
F S_LDL_TG_PER
G XL_HDL_PL_PER
H XL_HDL_C_PER
I XL_HDL_CE_PER
J XL_HDL_FC_PER
K XL_HDL_TG_PER
L L_HDL_PL_PER
M L_HDL_C_PER
N L_HDL_CE_PER
O L_HDL_FC_PER
P L_HDL_TG_PER
Q M_HDL_PL_PER
R M_HDL_C_PER
S M_HDL_CE_PER
T M_HDL_FC_PER
U M_HDL_TG_PER
V S_HDL_PL_PER
W S_HDL_C_PER
X S_HDL_CE_PER
Y S_HDL_FC_PER
Z S_HDL_TG_PER
AA VLDL_D
AB LDL_D
AC HDL_D
AD SERUM_C

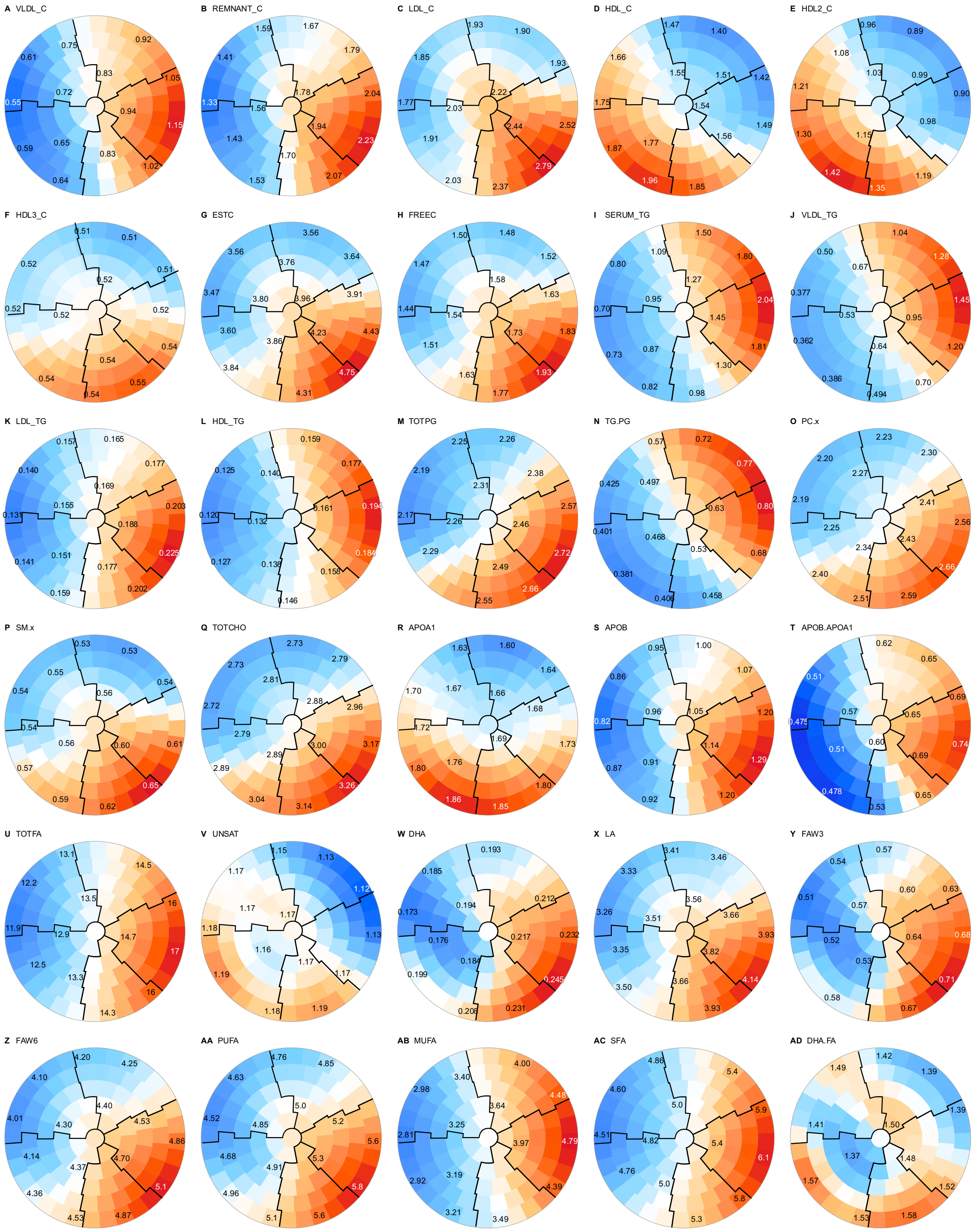
A VLDL_C
B REMNANT_C
C LDL_C
D HDL_C
E HDL2_C
F HDL3_C
G ESTC
H FREEC
I SERUM_TG
J VLDL_TG
K LDL_TG
L HDL_TG
M TOTPG
N TG.PG
O PC.x
P SM.x
Q TOTCHO
R APOA1
S APOB
T APOB.APOA1
U TOTFA
V UNSAT
W DHA
X LA
Y FAW3
Z FAW6
AA PUFA
AB MUFA
AC SFA
AD DHA.FA

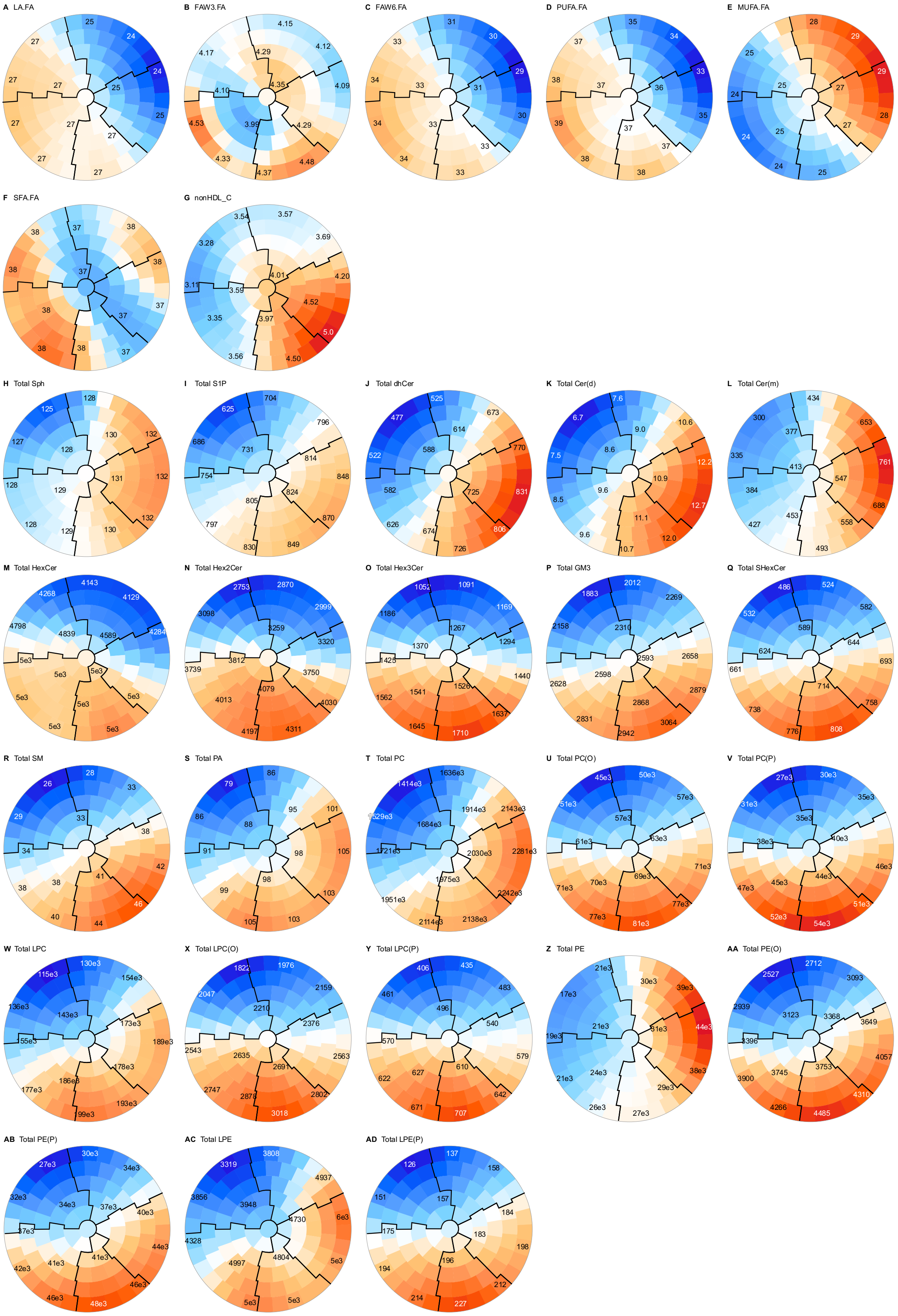
A LA.FA
B FAW3.FA
C FAW6.FA
D PUFA.FA
E MUFA.FA
F SFA.FA
G nonHDL_C
H Total Sph
I Total S1P
J Total dhCer
K Total Cer(d)
L Total Cer(m)
M Total HexCer
N Total Hex2Cer
O Total Hex3Cer
P Total GM3
Q Total SHexCer
R Total SM
S Total PA
T Total PC
U Total PC(O)
V Total PC(P)
W Total LPC
X Total LPC(O)
Y Total LPC(P)
Z Total PE
AA Total PE(O)
AB Total PE(P)
AC Total LPE
AD Total LPE(P)

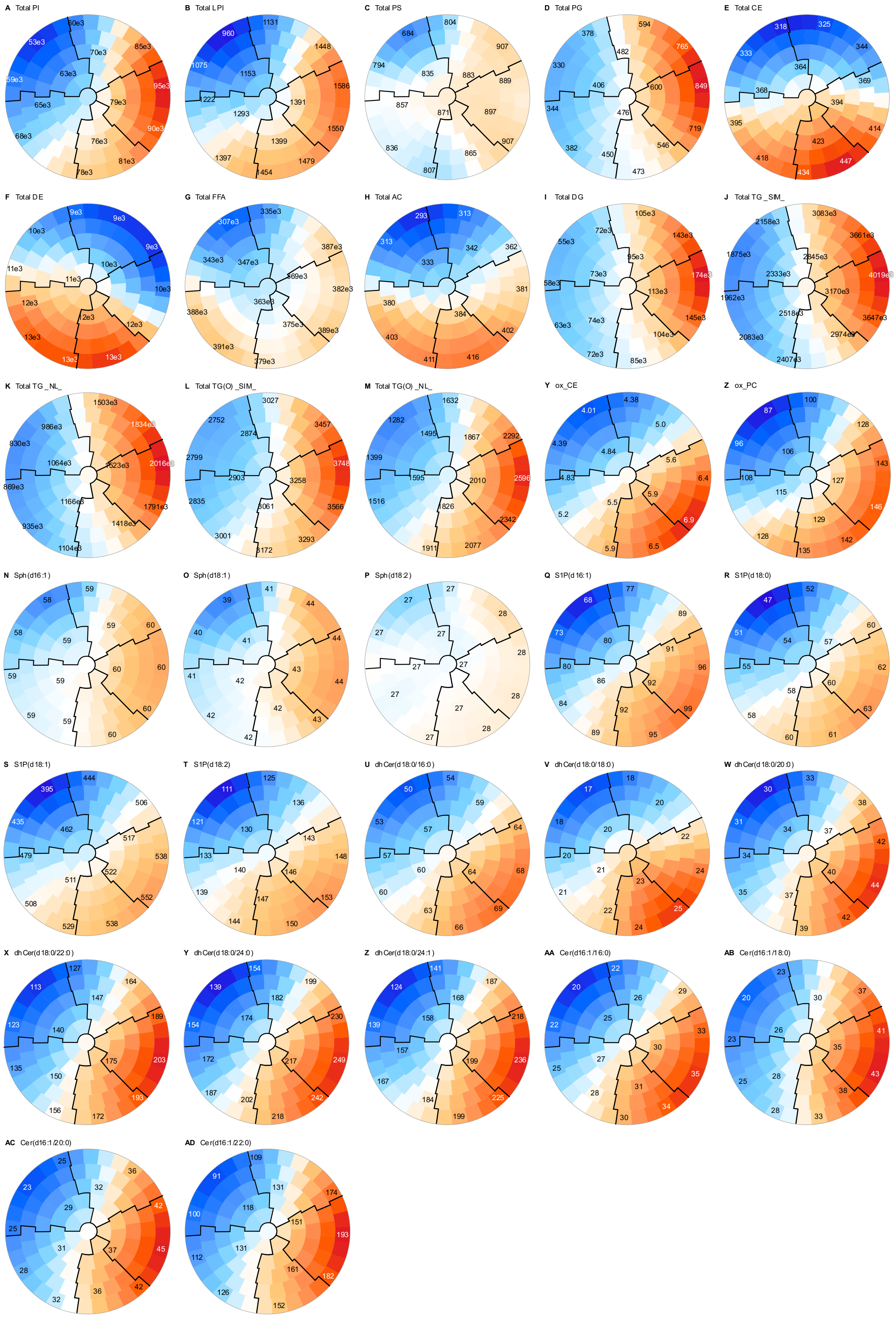
A Total PI
B Total LPI
C Total PS
D Total PG
E Total CE
F Total DE
G Total FFA
H Total AC
I Total DG
J Total TG _SIM_
K Total TG _NL_
L Total TG(O) _SIM_
M Total TG(O) _NL_
Y ox_CE
Z ox_PC
N Sph(d16:1)
O Sph(d18:1)
P Sph(d18:2)
Q S1P(d16:1)
R S1P(d18:0)
S S1P(d18:1)
T S1P(d18:2)
U dhCer(d18:0/16:0)
V dhCer(d18:0/18:0)
W dhCer(d18:0/20:0)
X dhCer(d18:0/22:0)
Y dhCer(d18:0/24:0)
Z dhCer(d18:0/24:1)
AA Cer(d16:1/16:0)
AB Cer(d16:1/18:0)
AC Cer(d16:1/20:0)
AD Cer(d16:1/22:0)

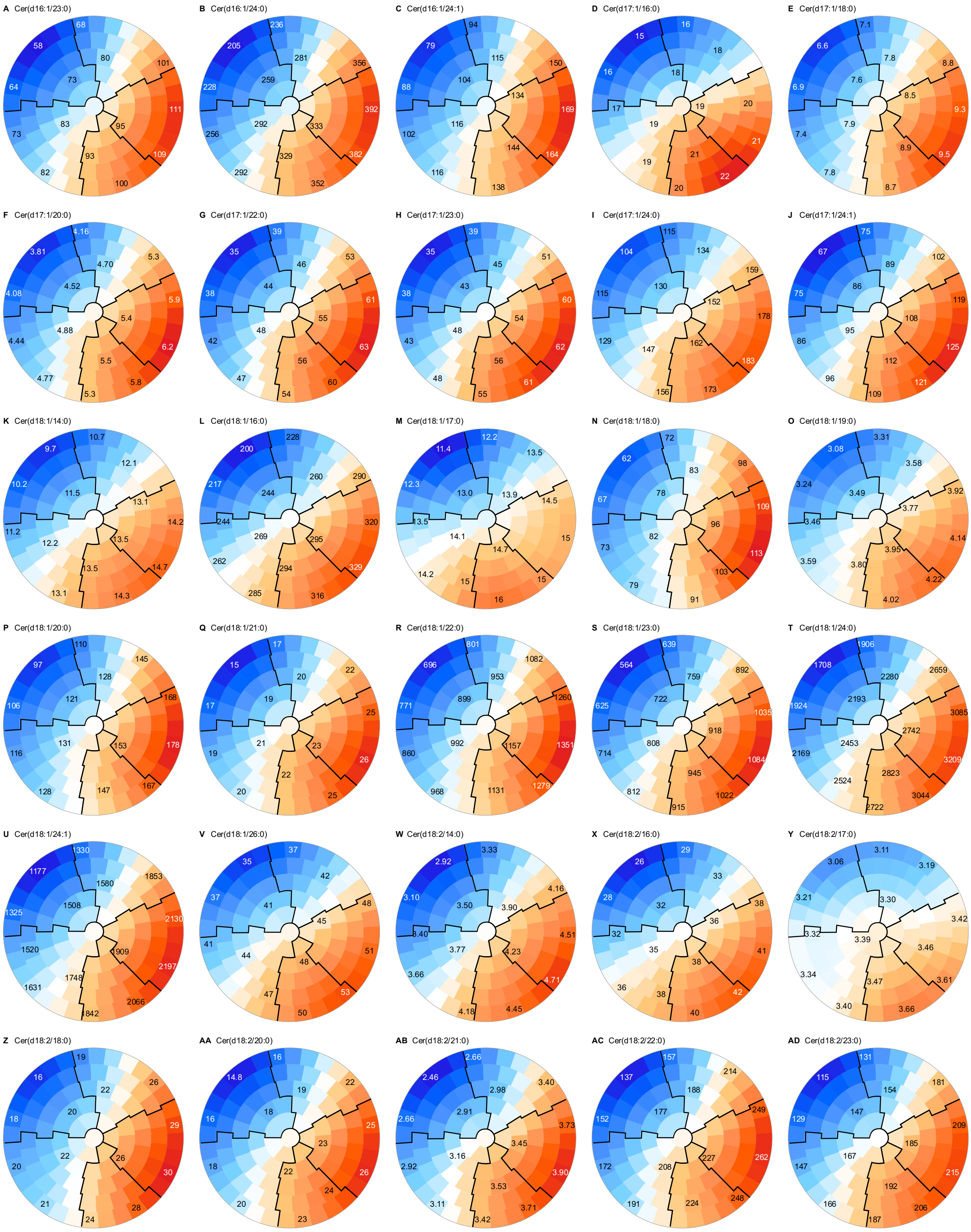
A Cer(d16:1/23:0)
68 58 80 101 73 64 111 83 95 73 109 93 82 100
B Cer(d16:1/24:0)
236 205 281 356 259 228 392 292 333 256 382 329 292 352
C Cer(d16:1/24:1)
94 79 115 150 104 134 88 169 116 102 144 164 116 138
D Cer(d17:1/16:0)
16 15 18 18 16 20 17 19 19 21 21 19 22 20
E Cer(d17:1/18:0)
7.1 6.6 7.8 8.8 7.6 8.5 6.9 9.3 7.9 7.4 8.9 9.5 7.8 8.7
F Cer(d17:1/20:0)
4.16 3.81 4.70 5.3 4.52 4.08 5.9 5.4 4.88 4.44 6.2 5.5 4.77 5.8 5.3
G Cer(d17:1/22:0)
39 35 46 53 44 38 61 55 48 42 63 56 47 60 54
H Cer(d17:1/23:0)
39 35 45 51 43 38 60 54 48 43 62 56 48 61 55
I Cer(d17:1/24:0)
115 104 134 159 130 115 152 178 129 147 162 183 156 173
J Cer(d17:1/24:1)
75 67 89 102 86 75 119 108 95 86 125 112 96 121 109
K Cer(d18:1/14:0)
10.7 9.7 12.1 10.2 11.5 13.1 14.2 11.2 12.2 13.5 13.5 14.7 13.1 14.3
L Cer(d18:1/16:0)
228 200 260 290 217 244 244 320 269 295 262 294 329 285 316
M Cer(d18:1/17:0)
12.2 11.4 13.5 12.3 13.0 13.9 14.5 13.5 14.1 15 14.7 14.2 15 15 16
N Cer(d18:1/18:0)
72 62 83 98 78 67 109 96 82 73 113 103 79 91
O Cer(d18:1/19:0)
3.31 3.08 3.58 3.24 3.49 3.92 3.77 3.46 4.14 3.95 3.59 3.80 4.22 4.02
P Cer(d18:1/20:0)
110 97 145 128 121 168 106 131 153 178 116 167 128 147
Q Cer(d18:1/21:0)
17 15 20 22 19 17 25 21 23 19 26 22 20 25
R Cer(d18:1/22:0)
801 696 1082 953 899 1260 771 992 1157 1351 860 1279 968 1131
S Cer(d18:1/23:0)
639 564 759 892 722 625 1035 918 808 714 1084 945 812 1022 915
T Cer(d18:1/24:0)
1906 1708 2280 2659 2193 1924 3085 2742 2453 2169 3209 2823 2524 3044 2722
U Cer(d18:1/24:1)
1330 1177 1580 1853 1508 1325 2130 1909 1520 2197 1748 1631 2066 1842
V Cer(d18:1/26:0)
37 35 42 48 37 41 45 41 44 51 48 53 47 50
W Cer(d18:2/14:0)
3.33 2.92 4.16 3.10 3.50 3.90 3.40 4.51 3.77 4.23 3.66 4.71 4.18 4.45
X Cer(d18:2/16:0)
29 26 33 28 32 38 36 32 35 41 38 36 42 38 40
Y Cer(d18:2/17:0)
3.11 3.06 3.19 3.21 3.30 3.42 3.32 3.39 3.46 3.34 3.47 3.61 3.40 3.66
Z Cer(d18:2/18:0)
19 16 22 26 20 18 29 22 26 20 30 21 28 24
AA Cer(d18:2/20:0)
16 14.8 19 22 18 16 25 23 22 18 26 24 20 23
AB Cer(d18:2/21:0)
2.66 2.46 2.98 3.40 2.91 2.66 3.73 3.45 3.16 2.92 3.90 3.53 3.11 3.71 3.42
AC Cer(d18:2/22:0)
157 137 214 188 177 249 152 227 208 262 172 248 191 224
AD Cer(d18:2/23:0)
131 115 154 181 147 129 209 185 167 147 215 192 166 206 187

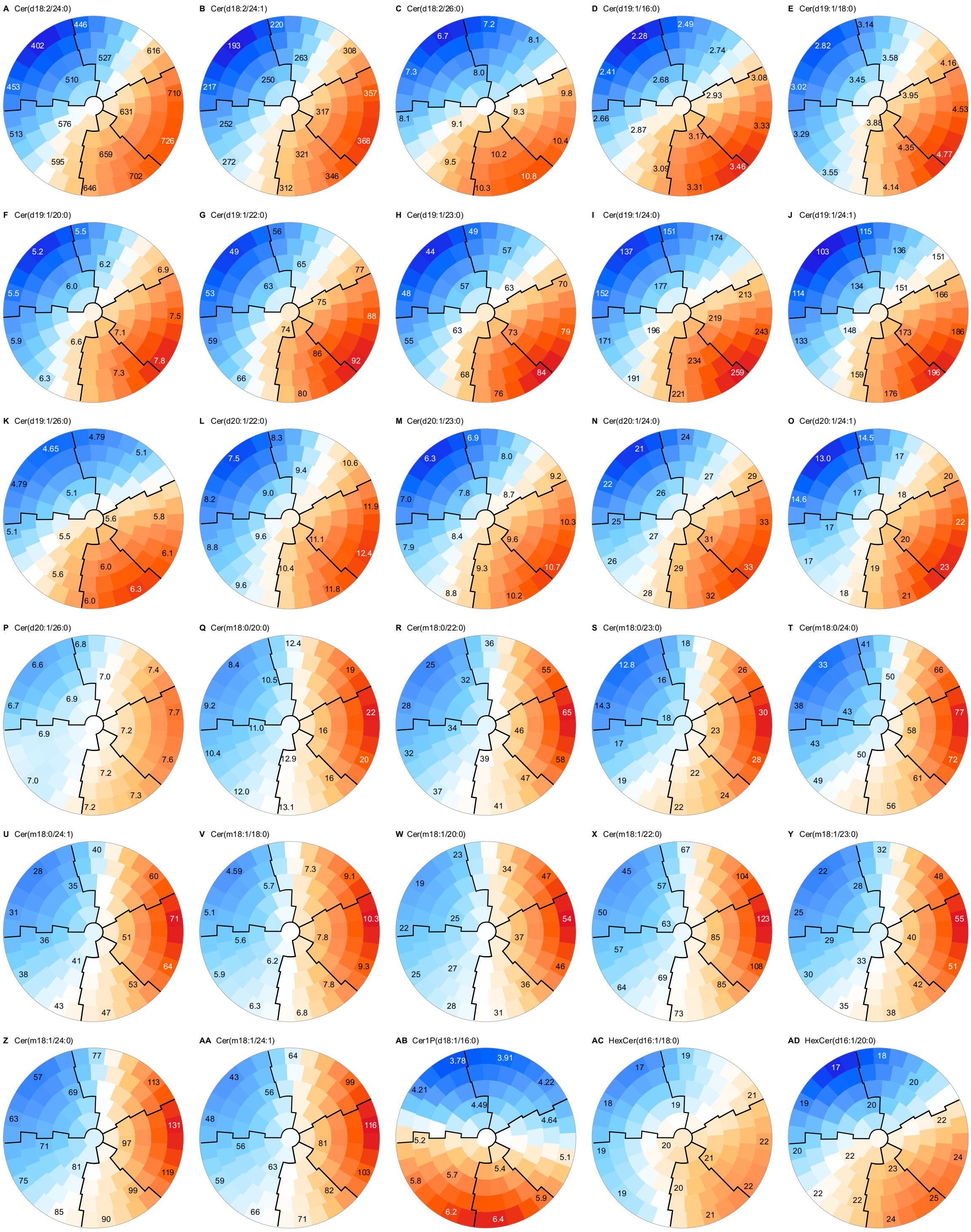
A Cer(d18:2/24:0)
B Cer(d18:2/24:1)
C Cer(d18:2/26:0)
D Cer(d19:1/16:0)
E Cer(d19:1/18:0)
F Cer(d19:1/20:0)
G Cer(d19:1/22:0)
H Cer(d19:1/23:0)
I Cer(d19:1/24:0)
J Cer(d19:1/24:1)
K Cer(d19:1/26:0)
L Cer(d20:1/22:0)
M Cer(d20:1/23:0)
N Cer(d20:1/24:0)
O Cer(d20:1/24:1)
P Cer(d20:1/26:0)
Q Cer(m18:0/20:0)
R Cer(m18:0/22:0)
S Cer(m18:0/23:0)
T Cer(m18:0/24:0)
U Cer(m18:0/24:1)
V Cer(m18:1/18:0)
W Cer(m18:1/20:0)
X Cer(m18:1/22:0)
Y Cer(m18:1/23:0)
Z Cer(m18:1/24:0)
AA Cer(m18:1/24:1)
AB Cer1P(d18:1/16:0)
AC HexCer(d16:1/18:0)
AD HexCer(d16:1/20:0)

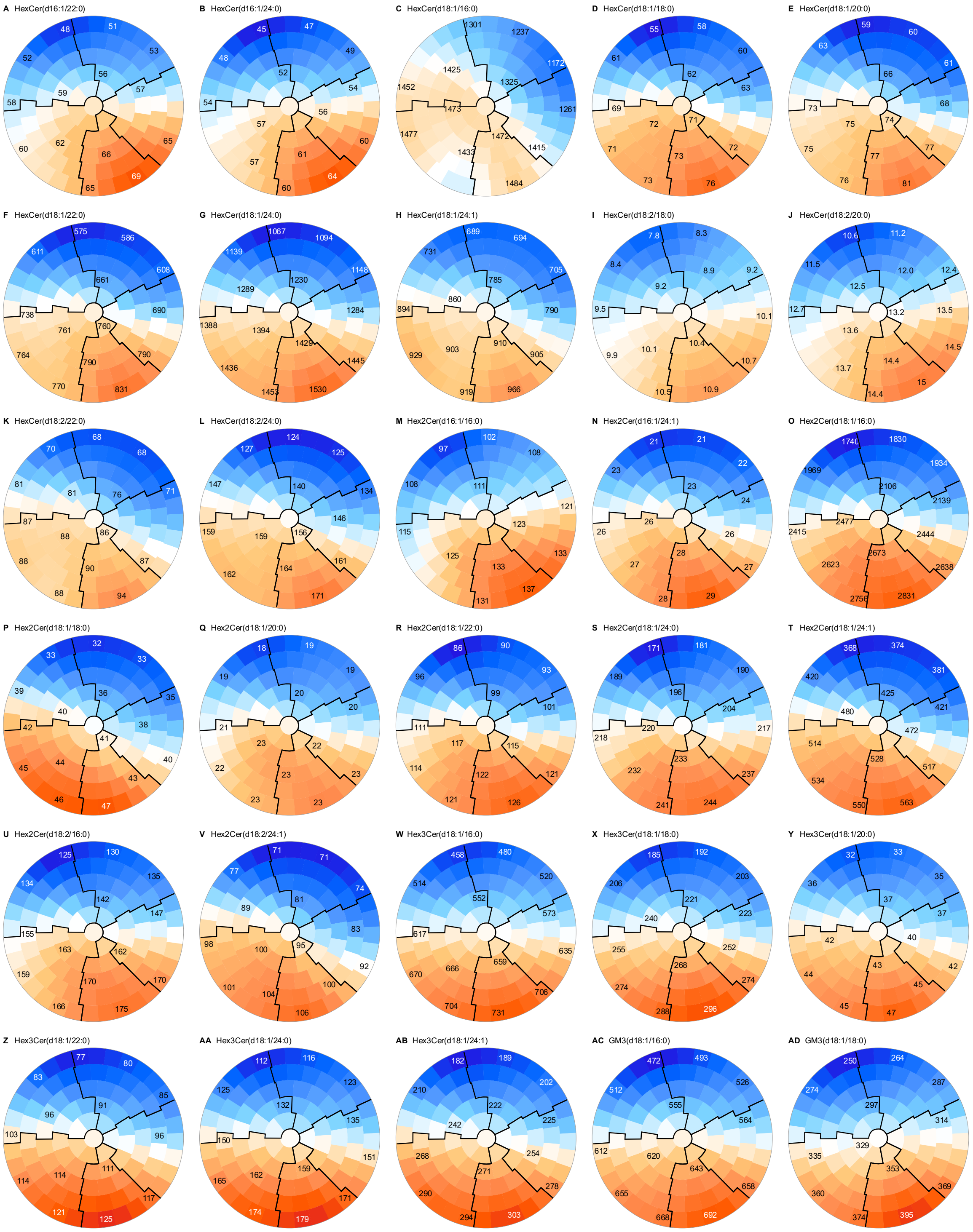
A HexCer(d16:1/22:0)
B HexCer(d16:1/24:0)
C HexCer(d18:1/16:0)
D HexCer(d18:1/18:0)
E HexCer(d18:1/20:0)
F HexCer(d18:1/22:0)
G HexCer(d18:1/24:0)
H HexCer(d18:1/24:1)
I HexCer(d18:2/18:0)
J HexCer(d18:2/20:0)
K HexCer(d18:2/22:0)
L HexCer(d18:2/24:0)
M Hex2Cer(d16:1/16:0)
N Hex2Cer(d16:1/24:1)
O Hex2Cer(d18:1/16:0)
P Hex2Cer(d18:1/18:0)
Q Hex2Cer(d18:1/20:0)
R Hex2Cer(d18:1/22:0)
S Hex2Cer(d18:1/24:0)
T Hex2Cer(d18:1/24:1)
U Hex2Cer(d18:2/16:0)
V Hex2Cer(d18:2/24:1)
W Hex3Cer(d18:1/16:0)
X Hex3Cer(d18:1/18:0)
Y Hex3Cer(d18:1/20:0)
Z Hex3Cer(d18:1/22:0)
AA Hex3Cer(d18:1/24:0)
AB Hex3Cer(d18:1/24:1)
AC GM3(d18:1/16:0)
AD GM3(d18:1/18:0)

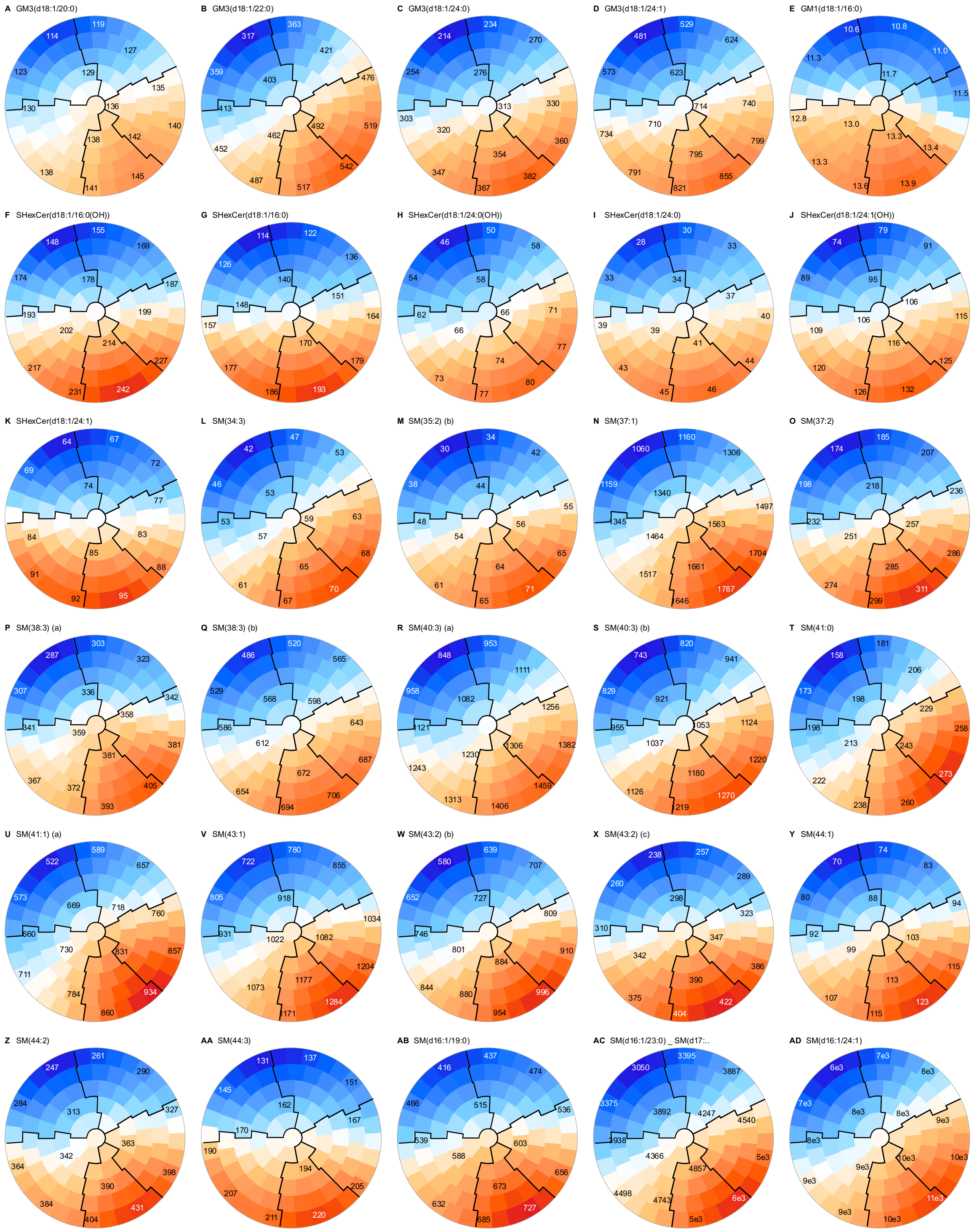
A GM3(d18:1/20:0)
B GM3(d18:1/22:0)
C GM3(d18:1/24:0)
D GM3(d18:1/24:1)
E GM1(d18:1/16:0)
F SHexCer(d18:1/16:0(OH))
G SHexCer(d18:1/16:0)
H SHexCer(d18:1/24:0(OH))
I SHexCer(d18:1/24:0)
J SHexCer(d18:1/24:1(OH))
K SHexCer(d18:1/24:1)
L SM(34:3)
M SM(35:2) (b)
N SM(37:1)
O SM(37:2)
P SM(38:3) (a)
Q SM(38:3) (b)
R SM(40:3) (a)
S SM(40:3) (b)
T SM(41:0)
U SM(41:1) (a)
V SM(43:1)
W SM(43:2) (b)
X SM(43:2) (c)
Y SM(44:1)
Z SM(44:2)
AA SM(44:3)
AB SM(d16:1/19:0)
AC SM(d16:1/23:0) _ SM(d17:..
AD SM(d16:1/24:1)

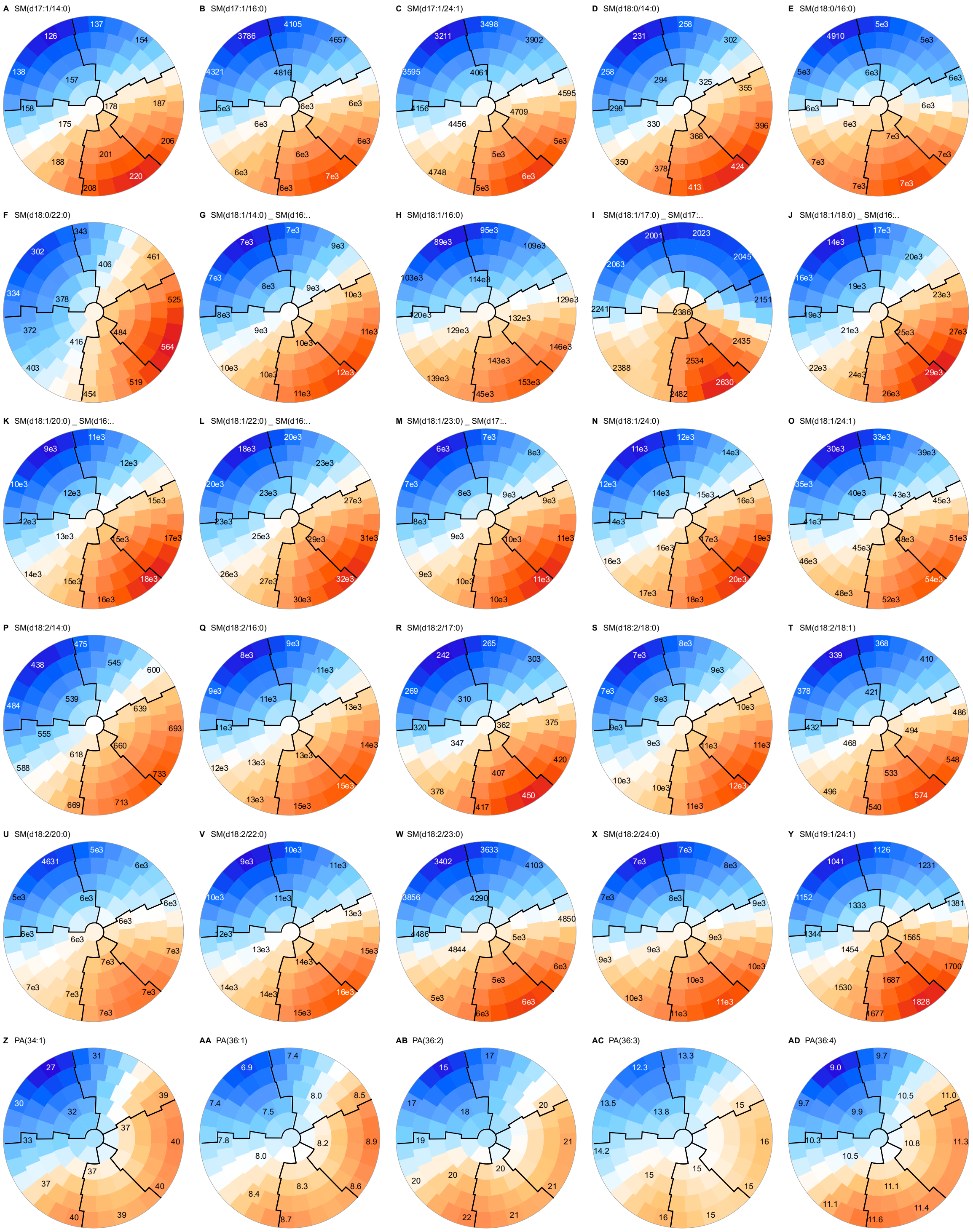
A SM(d17:1/14:0)
B SM(d17:1/16:0)
C SM(d17:1/24:1)
D SM(d18:0/14:0)
E SM(d18:0/16:0)
F SM(d18:0/22:0)
G SM(d18:1/14:0) _ SM(d16:..
H SM(d18:1/16:0)
I SM(d18:1/17:0) _ SM(d17:..
J SM(d18:1/18:0) _ SM(d16:..
K SM(d18:1/20:0) _ SM(d16:..
L SM(d18:1/22:0) _ SM(d16:..
M SM(d18:1/23:0) _ SM(d17:..
N SM(d18:1/24:0)
O SM(d18:1/24:1)
P SM(d18:2/14:0)
Q SM(d18:2/16:0)
R SM(d18:2/17:0)
S SM(d18:2/18:0)
T SM(d18:2/18:1)
U SM(d18:2/20:0)
V SM(d18:2/22:0)
W SM(d18:2/23:0)
X SM(d18:2/24:0)
Y SM(d19:1/24:1)
Z PA(34:1)
AA PA(36:1)
AB PA(36:2)
AC PA(36:3)
AD PA(36:4)

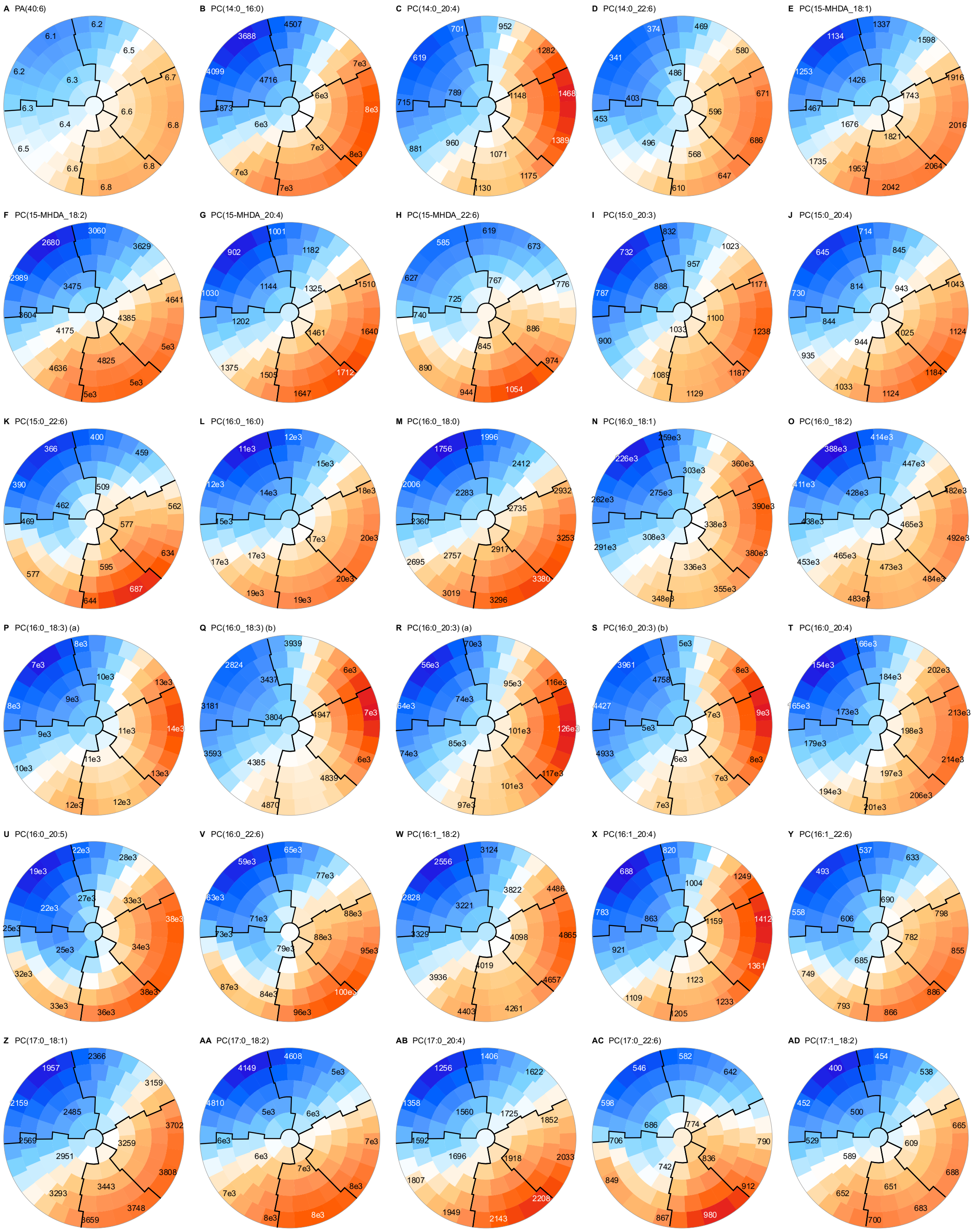
A PA(40:6)
B PC(14:0_16:0)
C PC(14:0_20:4)
D PC(14:0_22:6)
E PC(15-MHDA_18:1)
F PC(15-MHDA_18:2)
G PC(15-MHDA_20:4)
H PC(15-MHDA_22:6)
I PC(15:0_20:3)
J PC(15:0_20:4)
K PC(15:0_22:6)
L PC(16:0_16:0)
M PC(16:0_18:0)
N PC(16:0_18:1)
O PC(16:0_18:2)
P PC(16:0_18:3) (a)
Q PC(16:0_18:3) (b)
R PC(16:0_20:3) (a)
S PC(16:0_20:3) (b)
T PC(16:0_20:4)
U PC(16:0_20:5)
V PC(16:0_22:6)
W PC(16:1_18:2)
X PC(16:1_20:4)
Y PC(16:1_22:6)
Z PC(17:0_18:1)
AA PC(17:0_18:2)
AB PC(17:0_20:4)
AC PC(17:0_22:6)
AD PC(17:1_18:2)

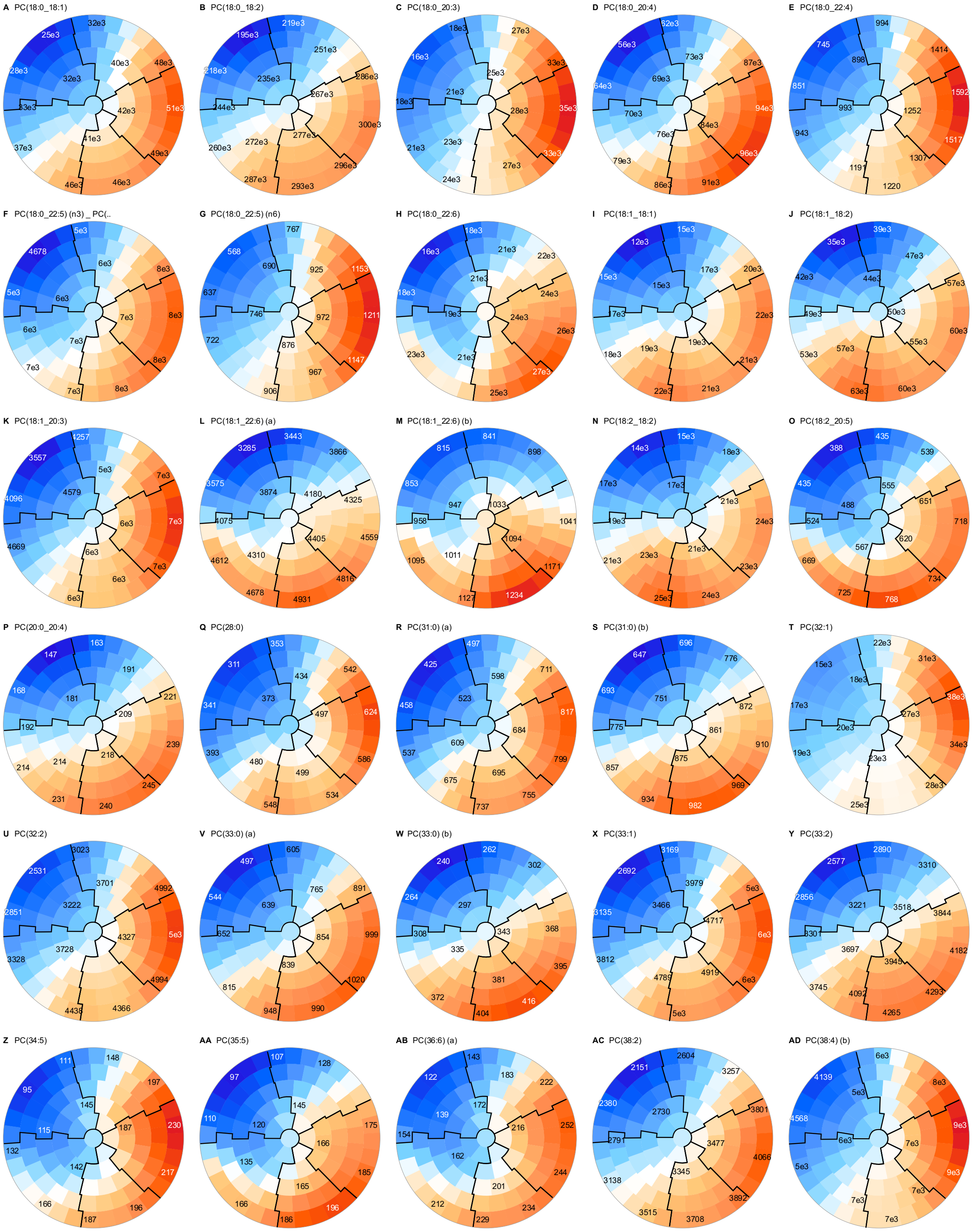
A PC(18:0_18:1)
25e3 32e3 40e3 48e3 28e3 32e3 51e3 42e3 33e3 41e3 37e3 49e3 46e3 46e3
B PC(18:0_18:2)
195e3 219e3 251e3 218e3 235e3 286e3 267e3 244e3 300e3 277e3 260e3 272e3 296e3 287e3 293e3
C PC(18:0_20:3)
18e3 27e3 16e3 33e3 25e3 21e3 18e3 28e3 35e3 21e3 23e3 33e3 27e3 24e3
D PC(18:0_20:4)
56e3 62e3 73e3 69e3 87e3 64e3 70e3 94e3 76e3 84e3 79e3 96e3 86e3 91e3
E PC(18:0_22:4)
745 994 898 1414 851 993 1252 1592 943 1517 1191 1307 1220
F PC(18:0_22:5) (n3) _ PC(..
4678 5e3 6e3 8e3 5e3 6e3 7e3 8e3 6e3 7e3 7e3 8e3 7e3 8e3
G PC(18:0_22:5) (n6)
568 767 690 925 1153 637 746 972 1211 722 876 1147 967 906
H PC(18:0_22:6)
16e3 18e3 21e3 22e3 21e3 18e3 24e3 19e3 24e3 26e3 23e3 21e3 27e3 25e3
I PC(18:1_18:1)
12e3 15e3 17e3 20e3 15e3 15e3 22e3 17e3 19e3 18e3 19e3 21e3 22e3 21e3
J PC(18:1_18:2)
35e3 39e3 47e3 42e3 44e3 57e3 50e3 49e3 60e3 55e3 53e3 57e3 63e3 60e3
K PC(18:1_20:3)
3557 4257 5e3 7e3 4096 4579 7e3 6e3 6e3 4669 7e3 6e3 6e3
L PC(18:1_22:6) (a)
3285 3443 3866 3575 3874 4180 4325 4075 4405 4559 4612 4310 4816 4678 4931
M PC(18:1_22:6) (b)
815 841 898 853 947 1033 958 1041 1094 1095 1011 1171 1127 1234
N PC(18:2_18:2)
14e3 15e3 18e3 17e3 17e3 21e3 19e3 24e3 21e3 23e3 21e3 23e3 25e3 24e3
O PC(18:2_20:5)
388 435 539 435 555 488 651 524 718 620 567 669 734 725 768
P PC(20:0_20:4)
147 163 191 168 181 221 209 192 239 218 214 214 245 231 240
Q PC(28:0)
311 353 434 542 341 373 497 624 393 480 586 499 548 534
R PC(31:0) (a)
425 497 598 711 458 523 817 684 537 609 799 675 695 755 737
S PC(31:0) (b)
647 696 776 693 751 872 775 861 910 857 875 969 934 982
T PC(32:1)
15e3 22e3 31e3 18e3 17e3 38e3 27e3 20e3 34e3 19e3 23e3 28e3 25e3
U PC(32:2)
2531 3023 3701 4992 2851 3222 5e3 4327 3328 3728 4994 4438 4366
V PC(33:0) (a)
497 605 765 891 544 639 652 854 999 839 815 1020 948 990
W PC(33:0) (b)
240 262 302 264 297 308 343 368 335 395 381 372 416 404
X PC(33:1)
2692 3169 3979 5e3 3135 3466 4717 6e3 3812 4789 4919 6e3 5e3
Y PC(33:2)
2577 2890 3310 2856 3221 3518 3844 3301 3697 3945 4182 3745 4092 4293 4265
Z PC(34:5)
111 148 95 197 145 187 230 115 132 142 217 166 196 187
AA PC(35:5)
97 107 128 145 110 120 175 166 135 185 165 166 196 186
AB PC(36:6) (a)
122 143 183 222 139 172 216 252 154 162 244 201 212 234 229
AC PC(38:2)
2151 2604 3257 2380 2730 3801 2791 3477 4066 3345 3138 3892 3515 3708
AD PC(38:4) (b)
4139 6e3 8e3 5e3 4568 6e3 7e3 9e3 5e3 9e3 7e3 7e3 7e3

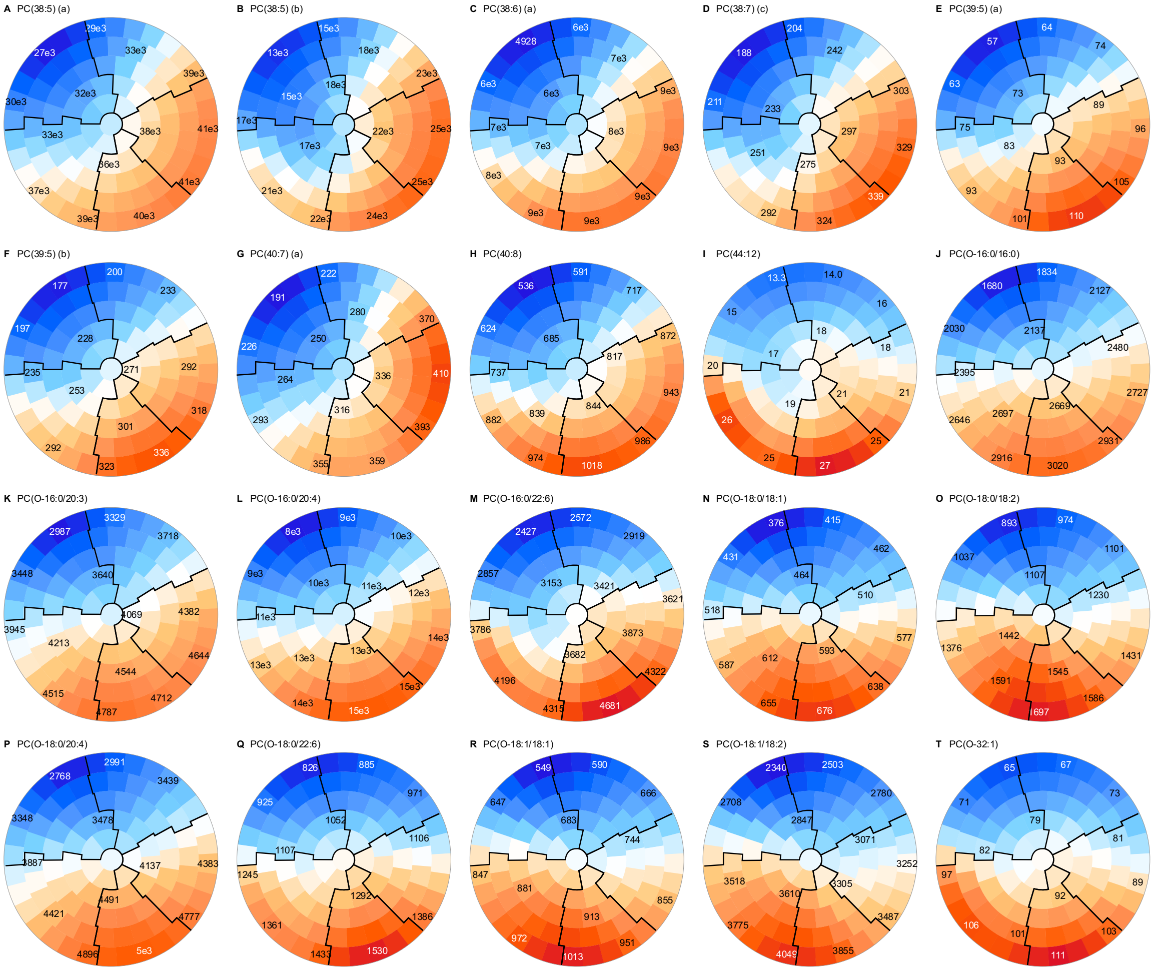
A PC(38:5) (a)
B PC(38:5) (b)
C PC(38:6) (a)
D PC(38:7) (c)
E PC(39:5) (a)
F PC(39:5) (b)
G PC(40:7) (a)
H PC(40:8)
I PC(44:12)
J PC(O-16:0/16:0)
K PC(O-16:0/20:3)
L PC(O-16:0/20:4)
M PC(O-16:0/22:6)
N PC(O-18:0/18:1)
O PC(O-18:0/18:2)
P PC(O-18:0/20:4)
Q PC(O-18:0/22:6)
R PC(O-18:1/18:1)
S PC(O-18:1/18:2)
T PC(O-32:1)

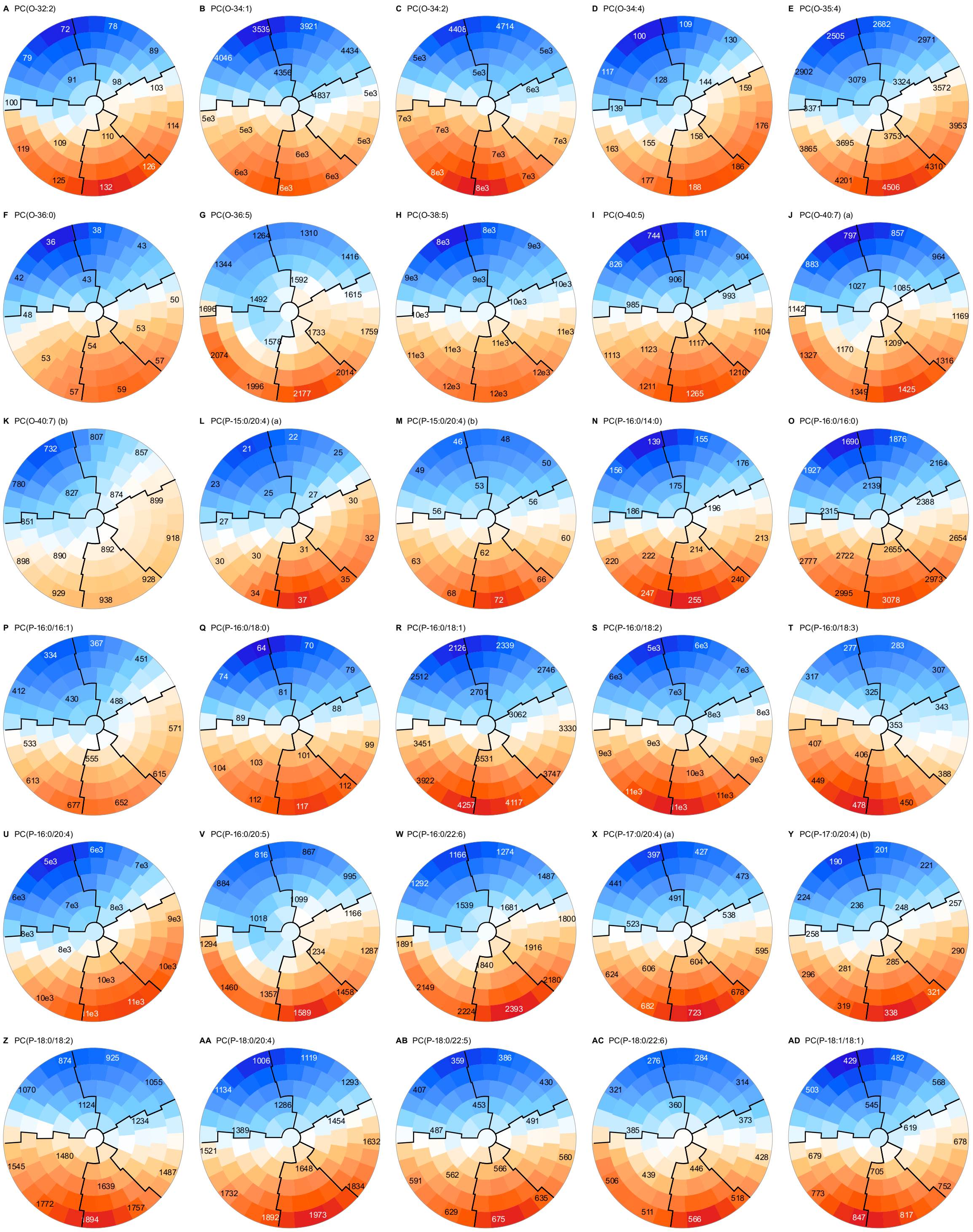
A PC(O-32:2)
B PC(O-34:1)
C PC(O-34:2)
D PC(O-34:4)
E PC(O-35:4)
F PC(O-36:0)
G PC(O-36:5)
H PC(O-38:5)
I PC(O-40:5)
J PC(O-40:7) (a)
K PC(O-40:7) (b)
L PC(P-15:0/20:4) (a)
M PC(P-15:0/20:4) (b)
N PC(P-16:0/14:0)
O PC(P-16:0/16:0)
P PC(P-16:0/16:1)
Q PC(P-16:0/18:0)
R PC(P-16:0/18:1)
S PC(P-16:0/18:2)
T PC(P-16:0/18:3)
U PC(P-16:0/20:4)
V PC(P-16:0/20:5)
W PC(P-16:0/22:6)
X PC(P-17:0/20:4) (a)
Y PC(P-17:0/20:4) (b)
Z PC(P-18:0/18:2)
AA PC(P-18:0/20:4)
AB PC(P-18:0/22:5)
AC PC(P-18:0/22:6)
AD PC(P-18:1/18:1)

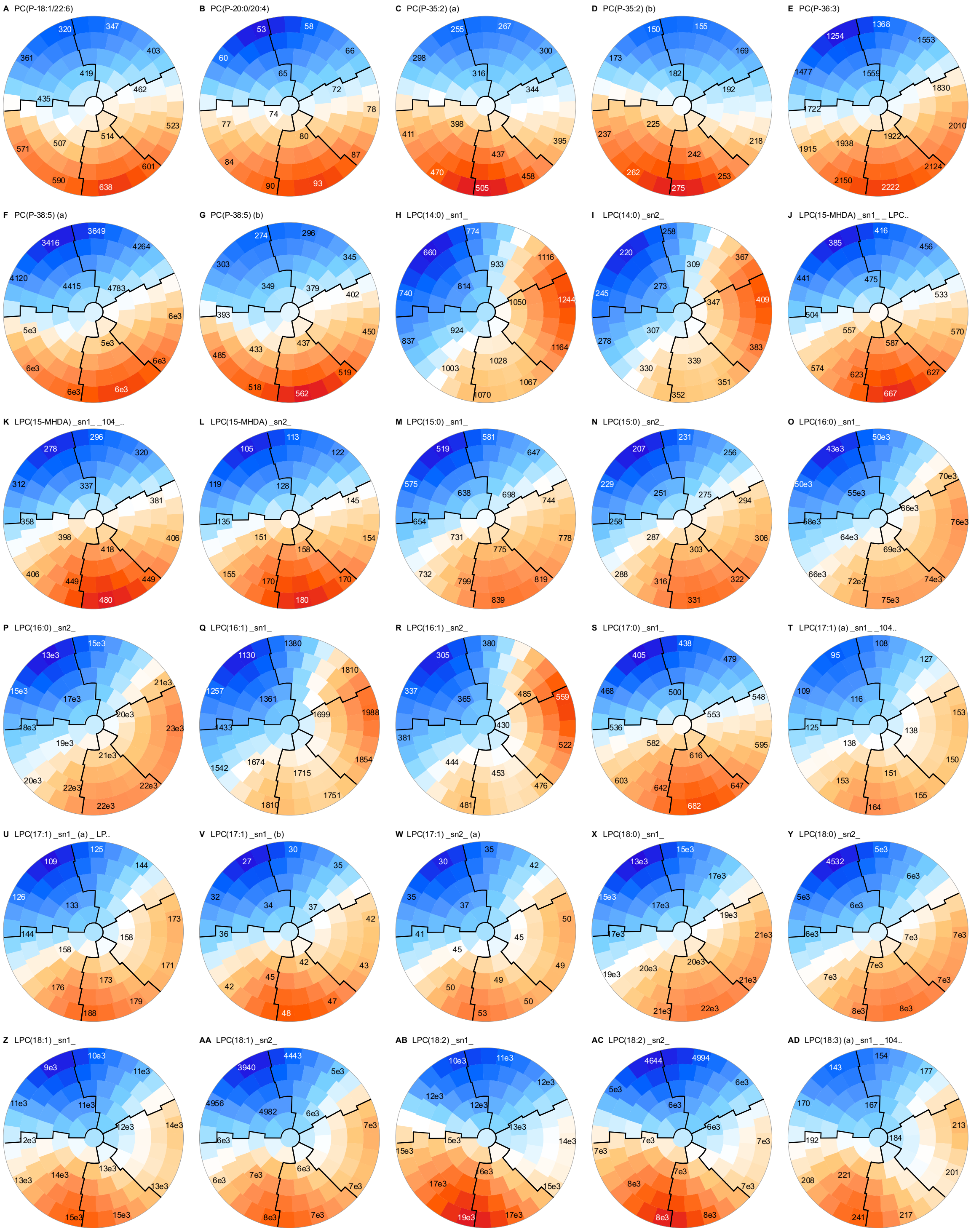
A PC(P-18:1/22:6)
320 347 361 403 419 462 435 523 507 514 571 601 590 638
B PC(P-20:0/20:4)
53 58 60 66 65 72 78 77 74 80 84 87 90 93
C PC(P-35:2) (a)
255 267 298 300 316 344 398 411 395 437 470 458 505
D PC(P-35:2) (b)
150 155 173 169 182 192 225 237 218 242 262 253 275
E PC(P-36:3)
1254 1368 1553 1477 1559 1830 1722 2010 1915 1938 1922 2124 2150 2222
F PC(P-38:5) (a)
3649 3416 4264 4120 4415 4783 6e3 5e3 5e3 6e3 6e3 6e3 6e3
G PC(P-38:5) (b)
274 296 303 345 349 379 402 393 450 437 485 433 519 518 562
H LPC(14:0) _sn1_
774 660 1116 933 814 740 1050 1244 924 837 1164 1003 1028 1067 1070
I LPC(14:0) _sn2_
258 220 367 309 273 245 347 409 278 307 383 330 339 351 352
J LPC(15-MHDA) _sn1_ _LPC..
416 385 456 441 475 533 504 570 557 587 574 623 627 667
K LPC(15-MHDA) _sn1_ _104_..
296 278 320 312 337 381 358 398 406 418 406 449 449 480
L LPC(15-MHDA) _sn2_
105 113 122 119 128 145 135 151 154 158 155 170 170 180
M LPC(15:0) _sn1_
519 581 647 575 638 698 744 654 731 778 775 732 799 819 839
N LPC(15:0) _sn2_
207 231 256 229 251 275 294 258 287 306 303 288 316 322 331
O LPC(16:0) _sn1_
43e3 50e3 70e3 50e3 55e3 66e3 58e3 76e3 64e3 69e3 66e3 72e3 74e3 75e3
P LPC(16:0) _sn2_
13e3 15e3 21e3 15e3 17e3 20e3 8e3 23e3 19e3 21e3 20e3 22e3 22e3 22e3
Q LPC(16:1) _sn1_
1130 1380 1810 1257 1361 1699 1988 1433 1674 1542 1715 1854 1751 1810
R LPC(16:1) _sn2_
305 380 485 559 337 365 430 381 522 444 453 476 481
S LPC(17:0) _sn1_
405 438 479 468 500 548 553 536 582 616 595 603 642 647 682
T LPC(17:1) (a) _sn1_ _104..
95 108 127 109 116 153 125 138 138 150 153 151 155 164
U LPC(17:1) _sn1_ (a) _ LP..
109 125 144 126 133 173 144 158 158 171 176 173 179 188
V LPC(17:1) _sn1_ (b)
27 30 35 32 34 37 42 36 42 43 42 45 47 48
W LPC(17:1) _sn2_ (a)
30 35 42 35 37 50 41 45 45 49 50 49 50 53
X LPC(18:0) _sn1_
13e3 15e3 17e3 15e3 17e3 19e3 7e3 21e3 20e3 19e3 20e3 21e3 21e3 22e3
Y LPC(18:0) _sn2_
4532 5e3 6e3 5e3 6e3 7e3 6e3 7e3 7e3 7e3 7e3 8e3 8e3
Z LPC(18:1) _sn1_
9e3 10e3 11e3 11e3 11e3 12e3 14e3 12e3 13e3 14e3 13e3 13e3 15e3 15e3
AA LPC(18:1) _sn2_
3940 4443 5e3 4956 4982 6e3 7e3 6e3 7e3 6e3 6e3 7e3 8e3 7e3
AB LPC(18:2) _sn1_
10e3 11e3 12e3 12e3 12e3 13e3 14e3 5e3 15e3 6e3 17e3 15e3 19e3 17e3
AC LPC(18:2) _sn2_
4644 4994 5e3 6e3 6e3 6e3 7e3 7e3 7e3 7e3 8e3 7e3 8e3 8e3
AD LPC(18:3) (a) _sn1_ _104..
143 154 177 170 167 213 192 184 208 221 201 241 217

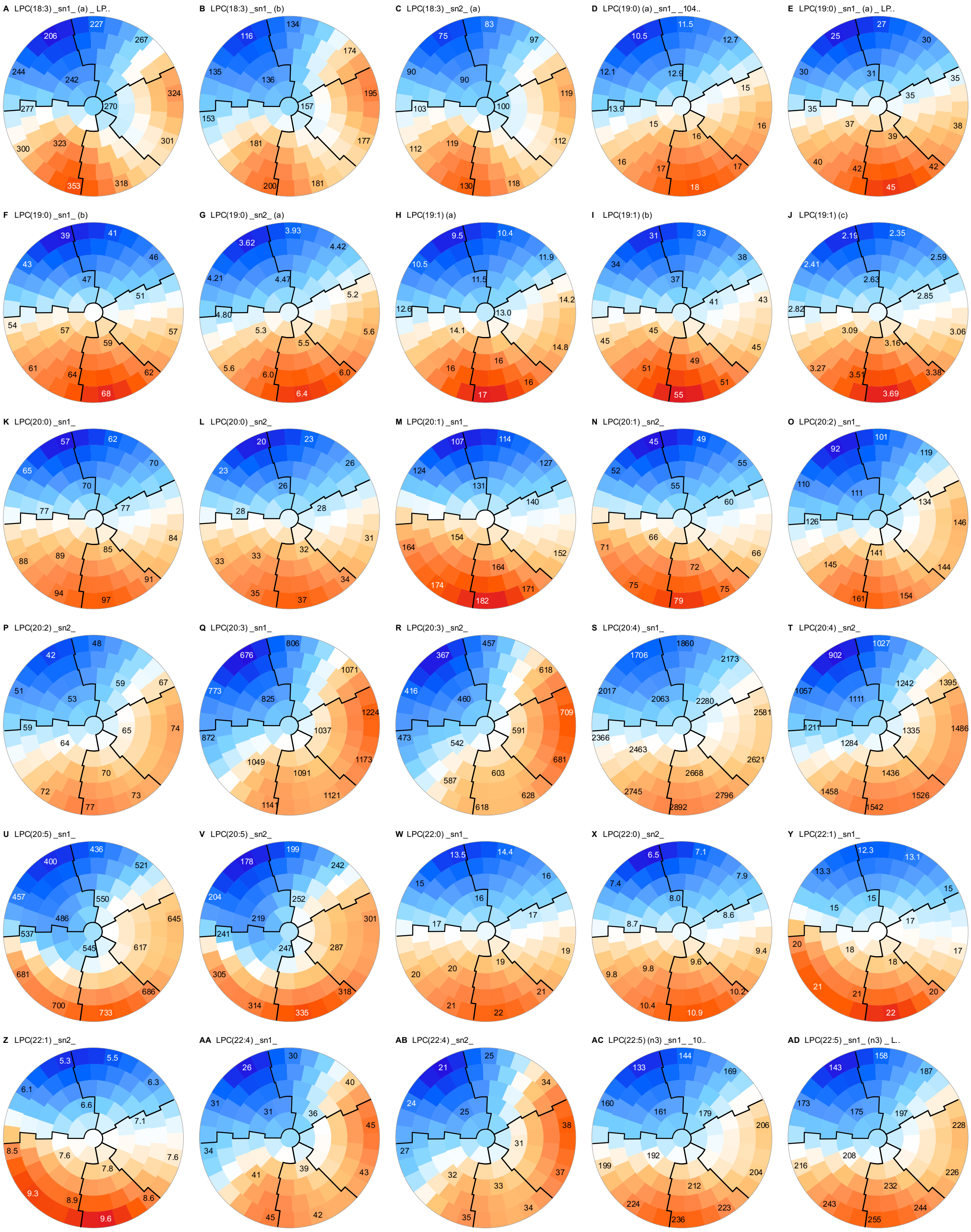
A LPC(18:3) _sn1_ (a) _ LP..
B LPC(18:3) _sn1_ (b)
C LPC(18:3) _sn2_ (a)
D LPC(19:0) (a) _sn1_ _104..
E LPC(19:0) _sn1_ (a) _ LP..
F LPC(19:0) _sn1_ (b)
G LPC(19:0) _sn2_ (a)
H LPC(19:1) (a)
I LPC(19:1) (b)
J LPC(19:1) (c)
K LPC(20:0) _sn1_
L LPC(20:0) _sn2_
M LPC(20:1) _sn1_
N LPC(20:1) _sn2_
O LPC(20:2) _sn1_
P LPC(20:2) _sn2_
Q LPC(20:3) _sn1_
R LPC(20:3) _sn2_
S LPC(20:4) _sn1_
T LPC(20:4) _sn2_
U LPC(20:5) _sn1_
V LPC(20:5) _sn2_
W LPC(22:0) _sn1_
X LPC(22:0) _sn2_
Y LPC(22:1) _sn1_
Z LPC(22:1) _sn2_
AA LPC(22:4) _sn1_
AB LPC(22:4) _sn2_
AC LPC(22:5) (n3) _sn1_ _10..
AD LPC(22:5) _sn1_ (n3) _ L..

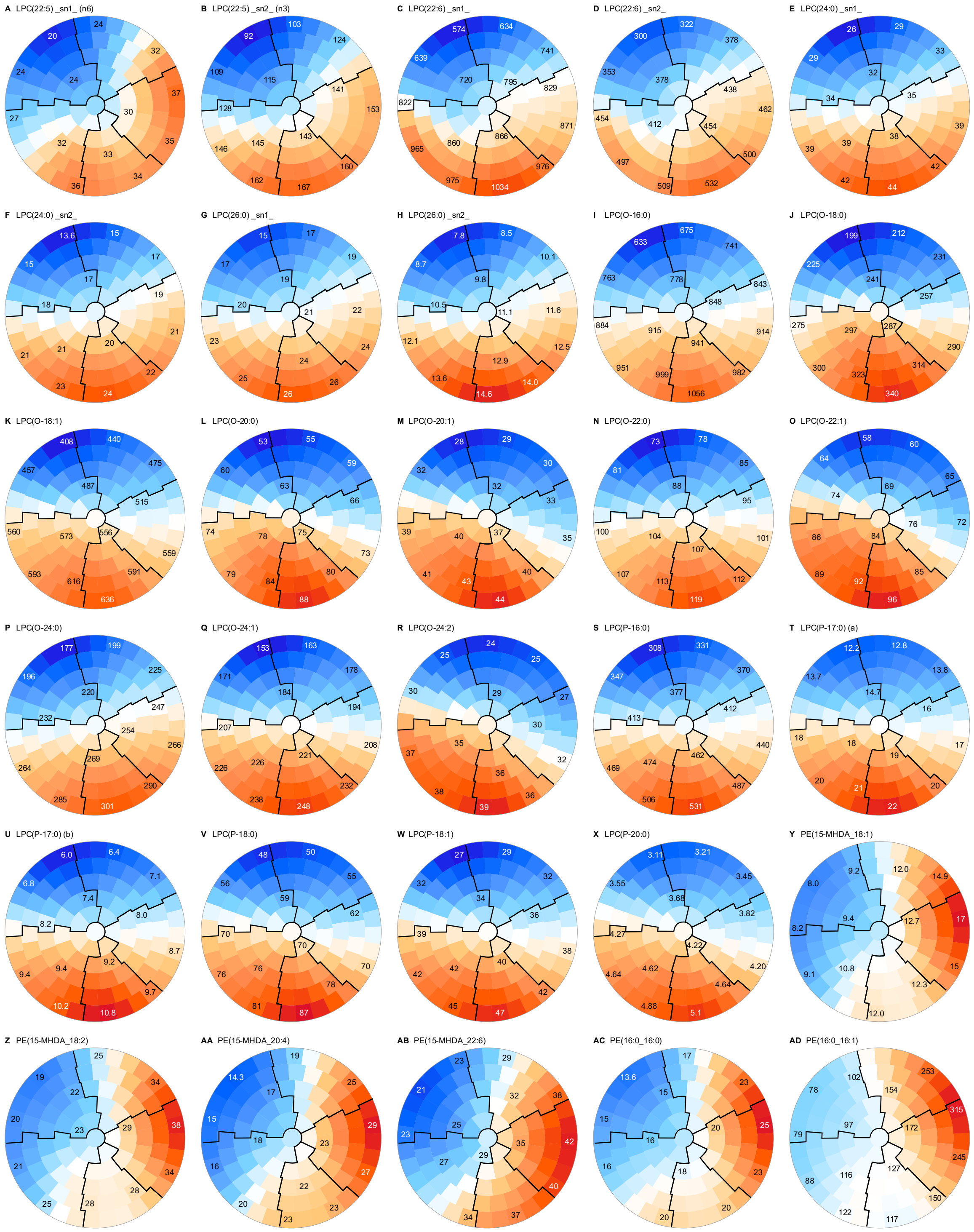
A LPC(22:5) _sn1_ (n6)
B LPC(22:5) _sn2_ (n3)
C LPC(22:6) _sn1_
D LPC(22:6) _sn2_
E LPC(24:0) _sn1_
F LPC(24:0) _sn2_
G LPC(26:0) _sn1_
H LPC(26:0) _sn2_
I LPC(O-16:0)
J LPC(O-18:0)
K LPC(O-18:1)
L LPC(O-20:0)
M LPC(O-20:1)
N LPC(O-22:0)
O LPC(O-22:1)
P LPC(O-24:0)
Q LPC(O-24:1)
R LPC(O-24:2)
S LPC(P-16:0)
T LPC(P-17:0) (a)
U LPC(P-17:0) (b)
V LPC(P-18:0)
W LPC(P-18:1)
X LPC(P-20:0)
Y PE(15-MHDA_18:1)
Z PE(15-MHDA_18:2)
AA PE(15-MHDA_20:4)
AB PE(15-MHDA_22:6)
AC PE(16:0_16:0)
AD PE(16:0_16:1)

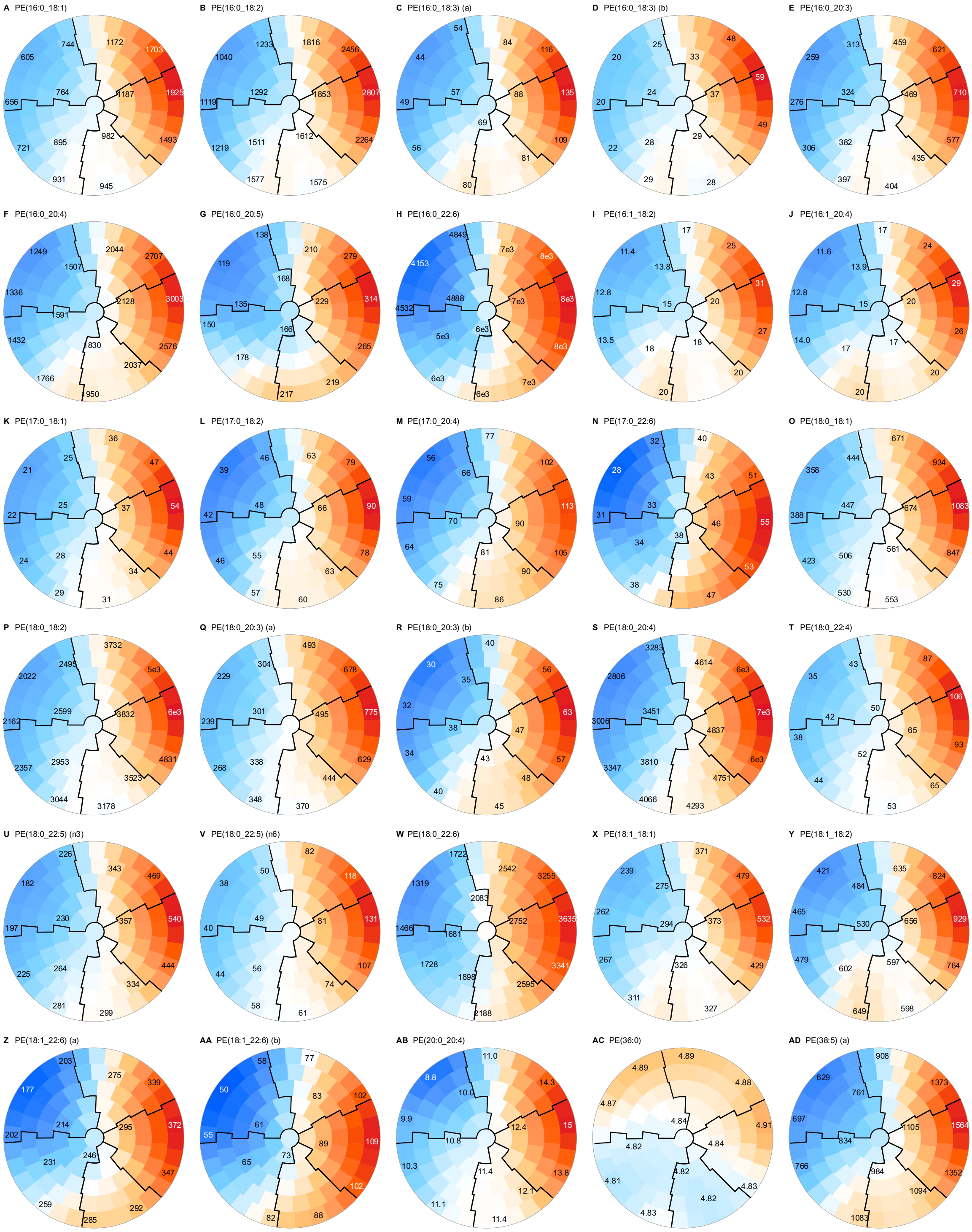
A PE(16:0_18:1)
B PE(16:0_18:2)
C PE(16:0_18:3) (a)
D PE(16:0_18:3) (b)
E PE(16:0_20:3)
F PE(16:0_20:4)
G PE(16:0_20:5)
H PE(16:0_22:6)
I PE(16:1_18:2)
J PE(16:1_20:4)
K PE(17:0_18:1)
L PE(17:0_18:2)
M PE(17:0_20:4)
N PE(17:0_22:6)
O PE(18:0_18:1)
P PE(18:0_18:2)
Q PE(18:0_20:3) (a)
R PE(18:0_20:3) (b)
S PE(18:0_20:4)
T PE(18:0_22:4)
U PE(18:0_22:5) (n3)
V PE(18:0_22:5) (n6)
W PE(18:0_22:6)
X PE(18:1_18:1)
Y PE(18:1_18:2)
Z PE(18:1_22:6) (a)
AA PE(18:1_22:6) (b)
AB PE(20:0_20:4)
AC PE(36:0)
AD PE(38:5) (a)

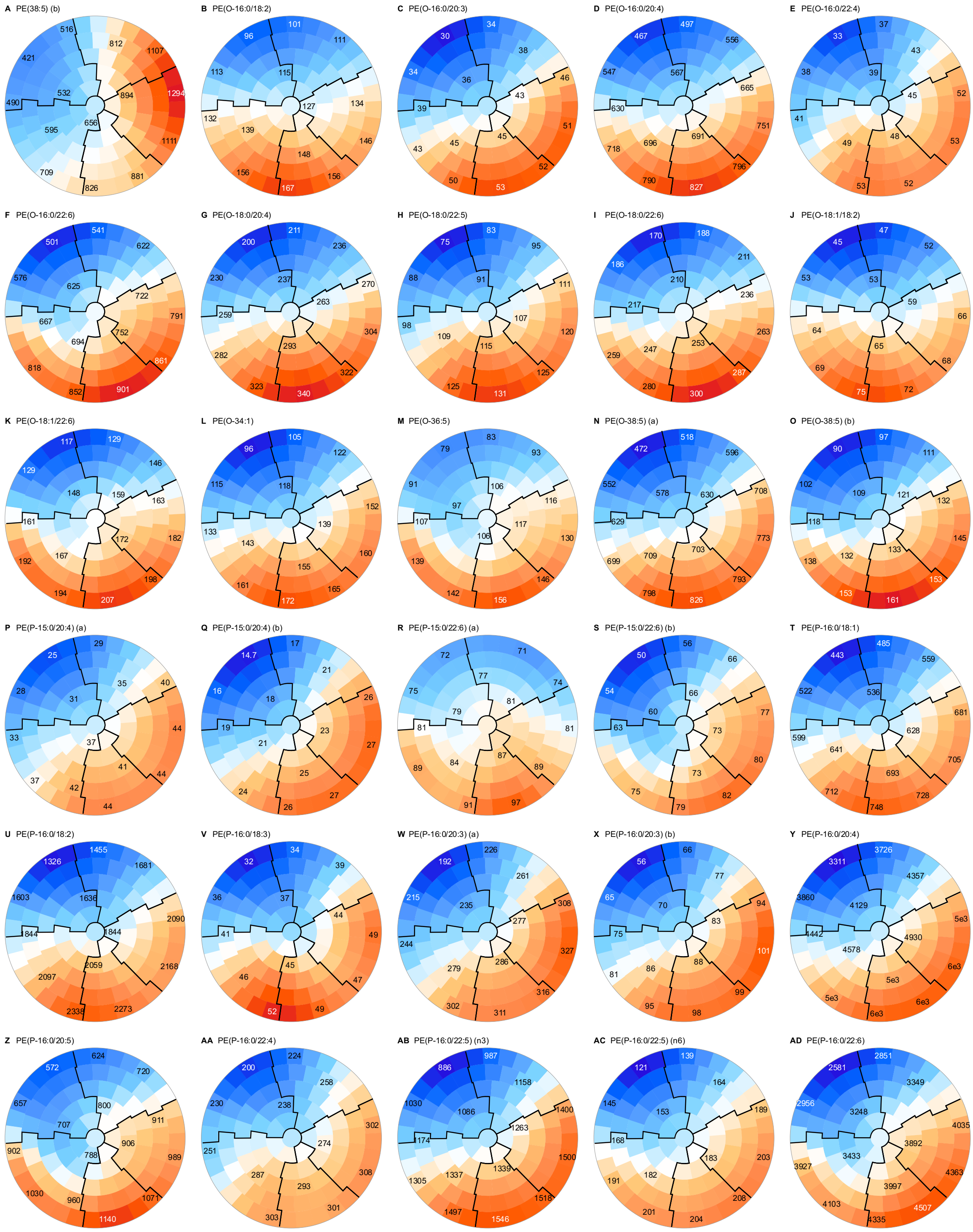
A PE(38:5) (b)
B PE(O-16:0/18:2)
C PE(O-16:0/20:3)
D PE(O-16:0/20:4)
E PE(O-16:0/22:4)
F PE(O-16:0/22:6)
G PE(O-18:0/20:4)
H PE(O-18:0/22:5)
I PE(O-18:0/22:6)
J PE(O-18:1/18:2)
K PE(O-18:1/22:6)
L PE(O-34:1)
M PE(O-36:5)
N PE(O-38:5) (a)
O PE(O-38:5) (b)
P PE(P-15:0/20:4) (a)
Q PE(P-15:0/20:4) (b)
R PE(P-15:0/22:6) (a)
S PE(P-15:0/22:6) (b)
T PE(P-16:0/18:1)
U PE(P-16:0/18:2)
V PE(P-16:0/18:3)
W PE(P-16:0/20:3) (a)
X PE(P-16:0/20:3) (b)
Y PE(P-16:0/20:4)
Z PE(P-16:0/20:5)
AA PE(P-16:0/22:4)
AB PE(P-16:0/22:5) (n3)
AC PE(P-16:0/22:5) (n6)
AD PE(P-16:0/22:6)

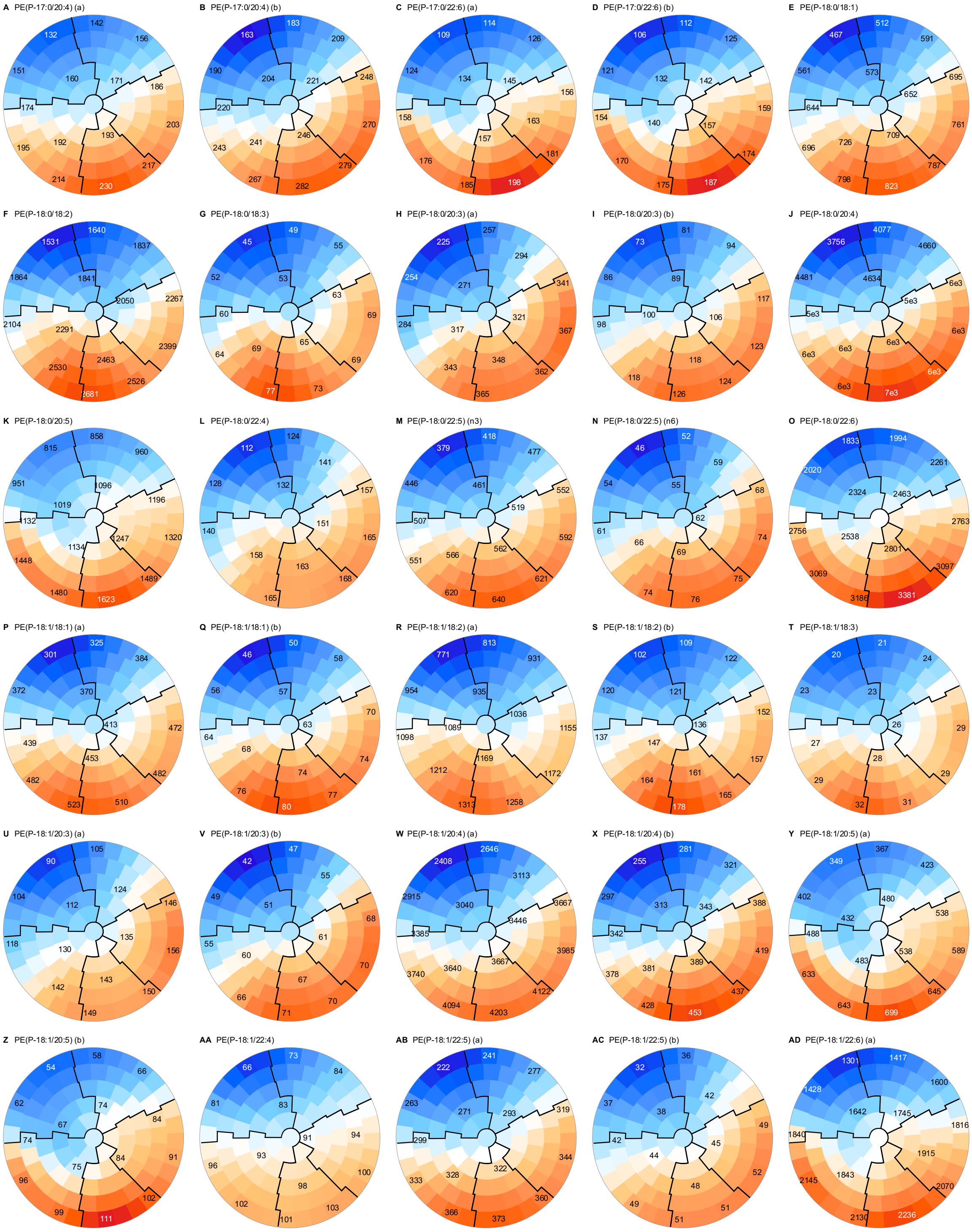

A PE(P-17:0/20:4) (a)
132 142 156 151 160 171 186 174 203 195 192 193 217 214 230
B PE(P-17:0/20:4) (b)
163 183 209 190 204 221 248 220 270 243 241 246 279 267 282
C PE(P-17:0/22:6) (a)
109 114 126 124 134 145 156 158 163 157 181 176 185 198
D PE(P-17:0/22:6) (b)
106 112 125 121 132 142 154 140 157 159 174 170 175 187
E PE(P-18:0/18:1)
467 512 591 561 573 652 695 644 709 761 696 726 787 798 823
F PE(P-18:0/18:2)
1531 1640 1837 1864 1841 2050 2267 2104 2291 2399 2463 2530 2526 2681
G PE(P-18:0/18:3)
45 49 55 52 53 63 69 60 65 64 69 69 77 73
H PE(P-18:0/20:3) (a)
225 257 294 254 271 341 321 367 284 317 343 348 362 365
I PE(P-18:0/20:3) (b)
73 81 94 86 89 117 98 100 106 123 118 118 124 126
J PE(P-18:0/20:4)
3756 4077 4660 4481 4634 6e3 5e3 5e3 6e3 6e3 6e3 6e3 6e3 6e3 7e3
K PE(P-18:0/20:5)
815 858 960 951 1096 1196 1019 1132 1247 1320 1134 1448 1489 1480 1623
L PE(P-18:0/22:4)
112 124 141 128 132 157 151 140 165 158 163 168 165
M PE(P-18:0/22:5) (n3)
379 418 477 446 461 552 519 507 592 551 566 562 621 620 640
N PE(P-18:0/22:5) (n6)
46 52 59 54 55 68 62 61 66 74 69 75 74 76
O PE(P-18:0/22:6)
1833 1994 2261 2020 2324 2463 2763 2756 2538 2801 3097 3069 3186 3381
P PE(P-18:1/18:1) (a)
301 325 384 372 370 413 472 439 453 482 482 523 510
Q PE(P-18:1/18:1) (b)
46 50 58 56 57 63 70 64 68 74 74 76 77 80
R PE(P-18:1/18:2) (a)
771 813 931 954 935 1036 1155 1098 1089 1169 1212 1172 1313 1258
S PE(P-18:1/18:2) (b)
102 109 122 120 121 136 152 137 147 157 161 164 165 178
T PE(P-18:1/18:3)
20 21 24 23 23 26 29 27 28 29 29 32 31
U PE(P-18:1/20:3) (a)
90 105 124 104 112 146 135 118 130 156 143 142 150 149
V PE(P-18:1/20:3) (b)
42 47 55 49 51 68 61 55 60 70 67 66 70 71
W PE(P-18:1/20:4) (a)
2408 2646 3113 2915 3040 3667 3446 3385 3985 3640 3667 3740 4122 4094 4203
X PE(P-18:1/20:4) (b)
255 281 321 297 313 343 388 342 389 419 381 378 437 428 453
Y PE(P-18:1/20:5) (a)
349 367 423 402 480 432 538 488 483 538 589 633 645 643 699
Z PE(P-18:1/20:5) (b)
54 58 66 62 74 67 84 74 75 84 91 96 102 99 111
AA PE(P-18:1/22:4)
66 73 84 81 83 94 91 96 93 98 100 102 103 101
AB PE(P-18:1/22:5) (a)
222 241 277 263 271 293 319 299 322 344 333 328 360 366 373
AC PE(P-18:1/22:5) (b)
32 36 42 37 38 49 42 45 44 48 52 49 51 51
AD PE(P-18:1/22:6) (a)
1301 1417 1428 1600 1642 1745 1816 1840 1915 1843 2145 2070 2130 2236

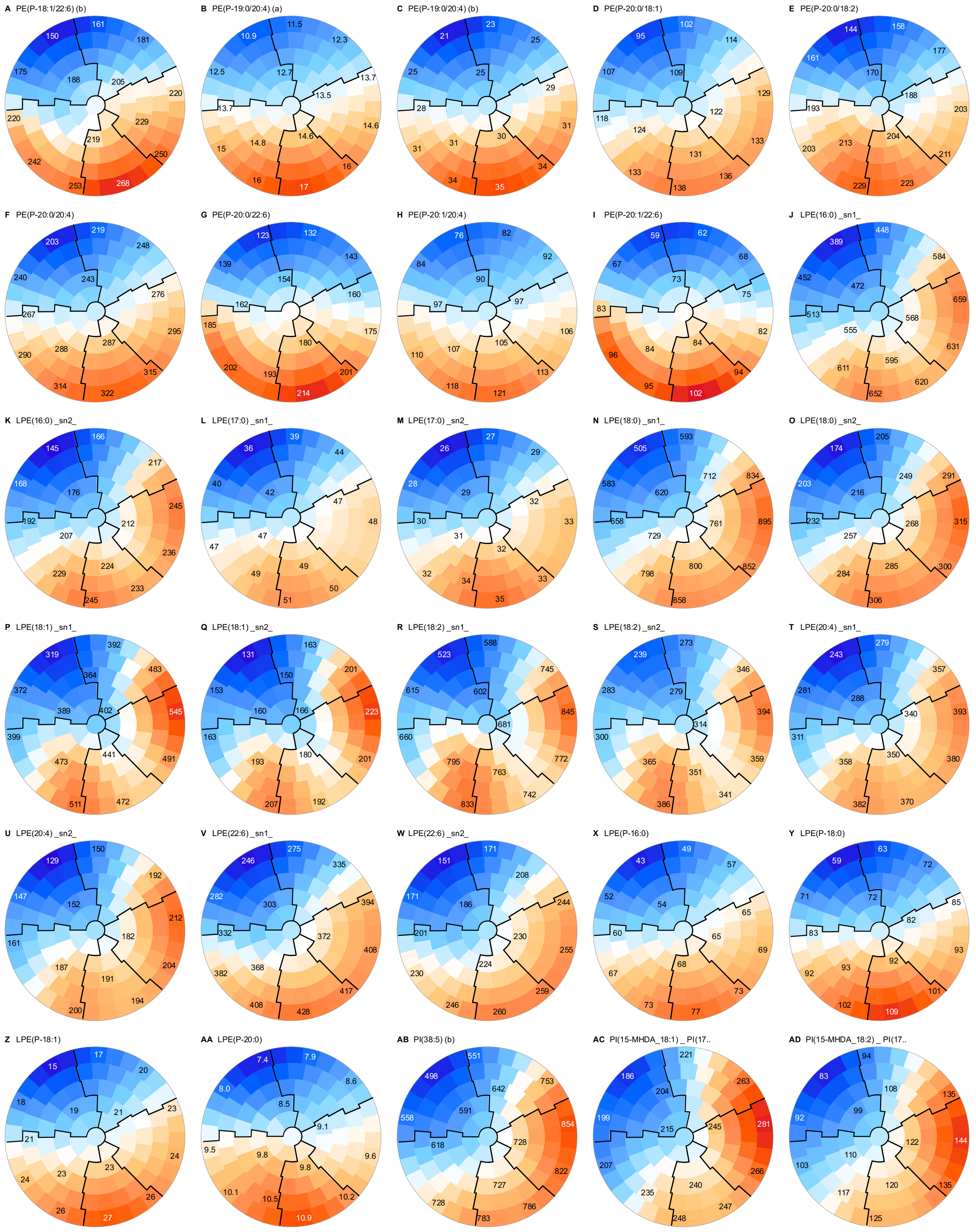
A PE(P-18:1/22:6) (b)
B PE(P-19:0/20:4) (a)
C PE(P-19:0/20:4) (b)
D PE(P-20:0/18:1)
E PE(P-20:0/18:2)
F PE(P-20:0/20:4)
G PE(P-20:0/22:6)
H PE(P-20:1/20:4)
I PE(P-20:1/22:6)
J LPE(16:0) _sn1_
K LPE(16:0) _sn2_
L LPE(17:0) _sn1_
M LPE(17:0) _sn2_
N LPE(18:0) _sn1_
O LPE(18:0) _sn2_
P LPE(18:1) _sn1_
Q LPE(18:1) _sn2_
R LPE(18:2) _sn1_
S LPE(18:2) _sn2_
T LPE(20:4) _sn1_
U LPE(20:4) _sn2_
V LPE(22:6) _sn1_
W LPE(22:6) _sn2_
X LPE(P-16:0)
Y LPE(P-18:0)
Z LPE(P-18:1)
AA LPE(P-20:0)
AB PI(38:5) (b)
AC PI(15-MHDA_18:1)_ PI(17..
AD PI(15-MHDA_18:2)_ PI(17..

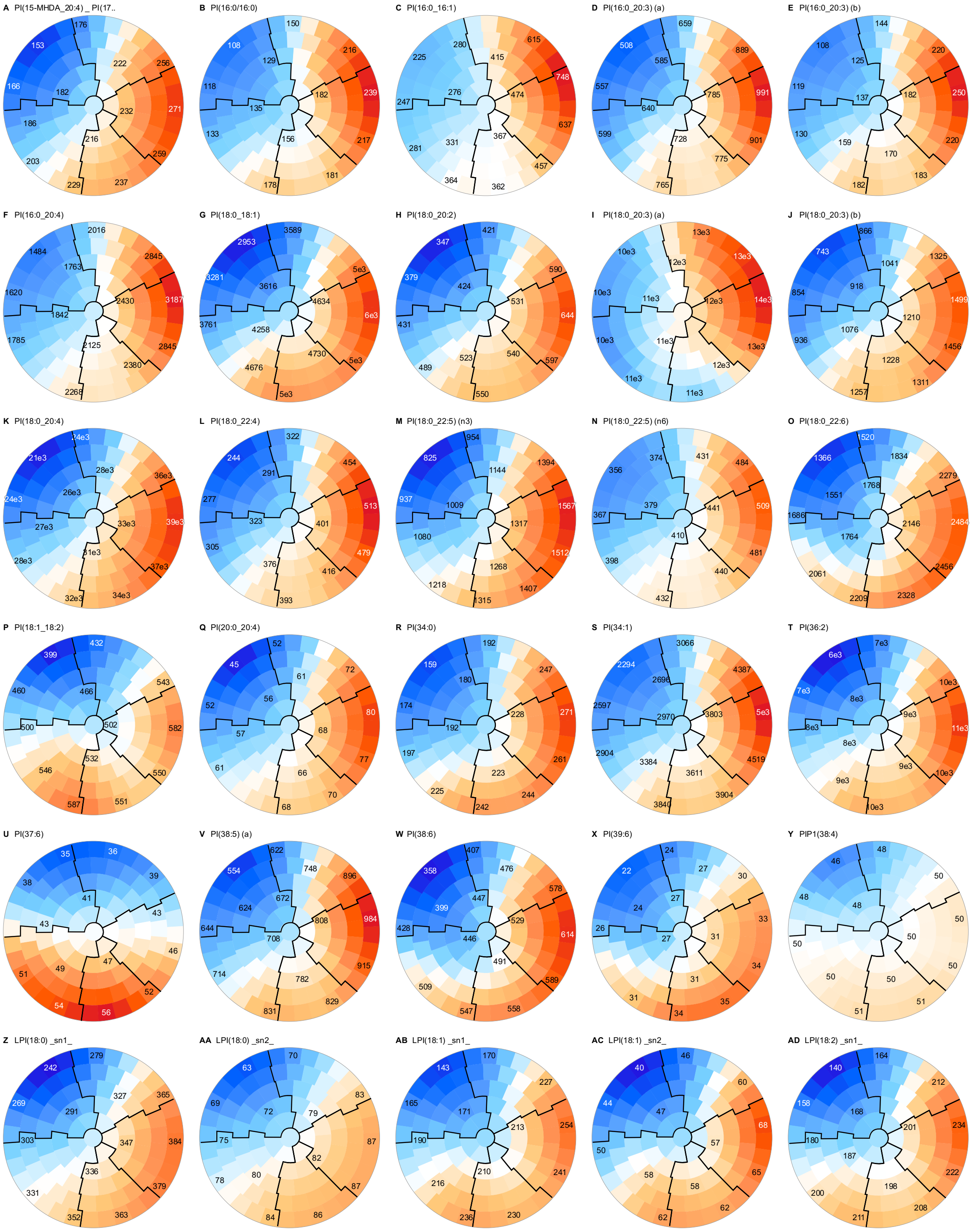
A PI(15-MHDA_20:4) _ PI(17..
176 153 222 256 166 182 232 271 186 216 259 203 229 237
B PI(16:0/16:0)
150 108 129 216 118 135 182 239 133 156 217 178 181
C PI(16:0_16:1)
280 225 415 615 276 474 748 247 367 637 281 331 457 364 362
D PI(16:0_20:3) (a)
659 508 585 889 557 640 785 991 599 728 901 775 765
E PI(16:0_20:3) (b)
144 108 125 220 119 137 182 250 130 159 170 220 182 183
F PI(16:0_20:4)
2016 1484 1763 2845 1620 2430 3187 1842 1785 2125 2845 2380 2268
G PI(18:0_18:1)
2953 3589 5e3 3281 3616 4634 6e3 3761 4258 4730 5e3 4676 5e3
H PI(18:0_20:2)
347 421 590 379 424 531 644 431 540 597 489 523 550
I PI(18:0_20:3) (a)
13e3 10e3 12e3 13e3 10e3 11e3 12e3 14e3 10e3 11e3 13e3 12e3 11e3 11e3
J PI(18:0_20:3) (b)
866 743 1041 1325 854 918 1499 1210 936 1076 1456 1228 1311 1257
K PI(18:0_20:4)
24e3 21e3 28e3 36e3 24e3 26e3 33e3 39e3 27e3 31e3 37e3 28e3 32e3 34e3
L PI(18:0_22:4)
322 244 291 454 277 323 401 513 305 376 416 479 393
M PI(18:0_22:5) (n3)
954 825 1144 1394 937 1009 1317 1567 1080 1268 1512 1218 1315 1407
N PI(18:0_22:5) (n6)
374 356 431 484 367 379 441 509 410 398 440 481 432
O PI(18:0_22:6)
1520 1366 1834 2279 1551 1768 1686 2146 2484 1764 2061 2456 2209 2328
P PI(18:1_18:2)
432 399 543 460 466 500 502 582 532 546 550 587 551
Q PI(20:0_20:4)
52 45 61 72 52 56 68 80 57 77 61 66 70 68
R PI(34:0)
192 159 180 247 174 228 271 192 197 261 223 225 244 242
S PI(34:1)
3066 2294 2696 4387 2597 2970 3803 5e3 2904 3384 3611 4519 3904 3840
T PI(36:2)
7e3 6e3 10e3 7e3 8e3 9e3 8e3 11e3 8e3 9e3 10e3 9e3 10e3
U PI(37:6)
35 36 38 39 41 43 43 46 47 49 51 52 54 56
V PI(38:5) (a)
622 554 748 896 672 624 808 984 644 708 915 714 782 829 831
W PI(38:6)
407 358 476 447 578 399 529 428 446 614 491 509 589 547 558
X PI(39:6)
24 22 27 30 27 24 33 26 27 31 34 31 31 35 34
Y PIP1(38:4)
48 46 50 48 48 50 50 50 50 50 50 51 51
Z LPI(18:0) _sn1_
279 242 327 365 269 291 347 384 303 336 379 331 352 363
AA LPI(18:0) _sn2_
70 63 83 69 72 79 87 75 82 78 80 87 84 86
AB LPI(18:1) _sn1_
170 143 227 165 171 213 254 190 210 241 216 236 230
AC LPI(18:1) _sn2_
46 40 60 44 47 68 57 50 65 58 58 62 62
AD LPI(18:2) _sn1_
164 140 212 158 168 201 234 180 187 222 198 200 208 211

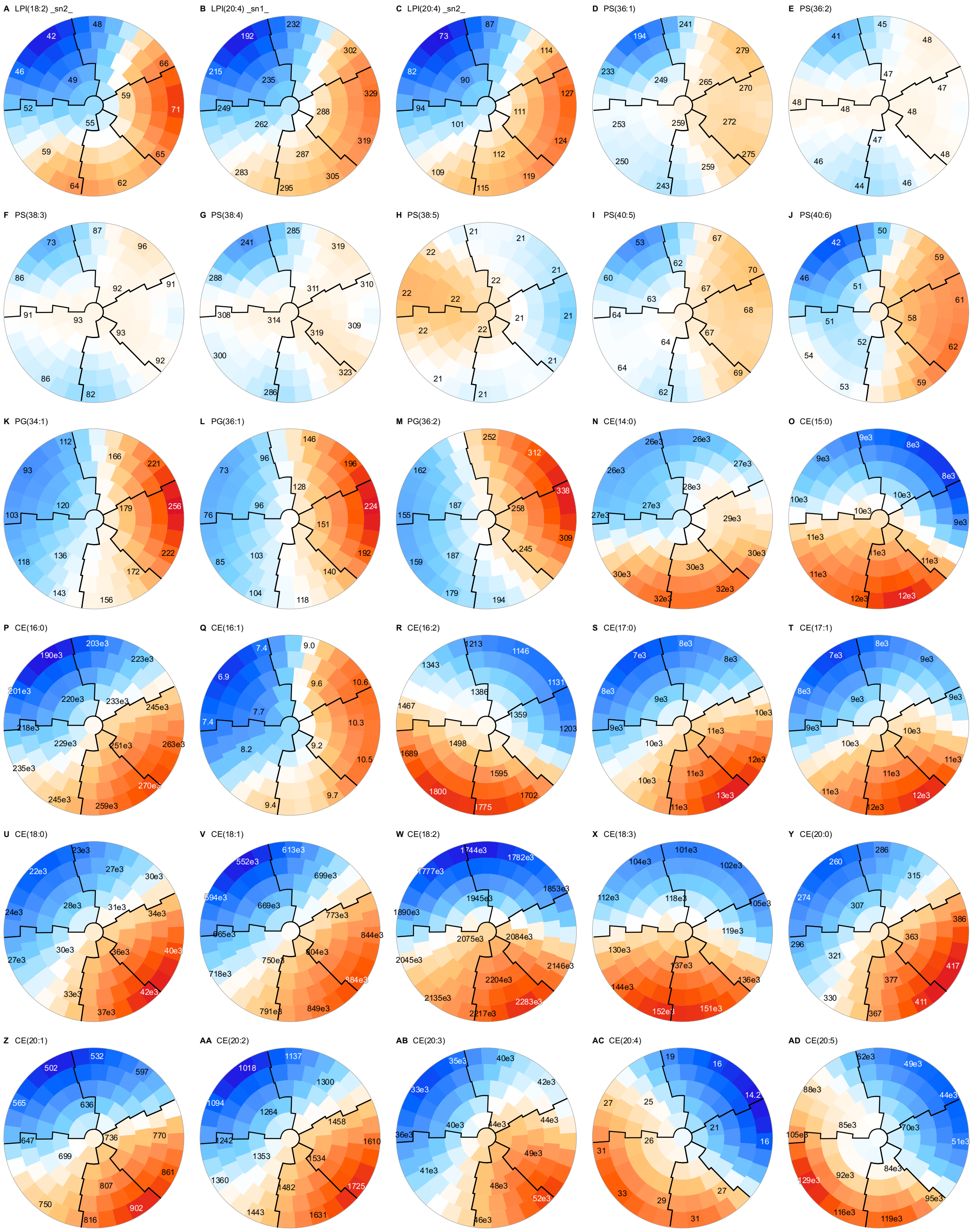
A LPI(18:2) _sn2_
B LPI(20:4) _sn1_
C LPI(20:4) _sn2_
D PS(36:1)
E PS(36:2)
F PS(38:3)
G PS(38:4)
H PS(38:5)
I PS(40:5)
J PS(40:6)
K PG(34:1)
L PG(36:1)
M PG(36:2)
N CE(14:0)
O CE(15:0)
P CE(16:0)
Q CE(16:1)
R CE(16:2)
S CE(17:0)
T CE(17:1)
U CE(18:0)
V CE(18:1)
W CE(18:2)
X CE(18:3)
Y CE(20:0)
Z CE(20:1)
AA CE(20:2)
AB CE(20:3)
AC CE(20:4)
AD CE(20:5)

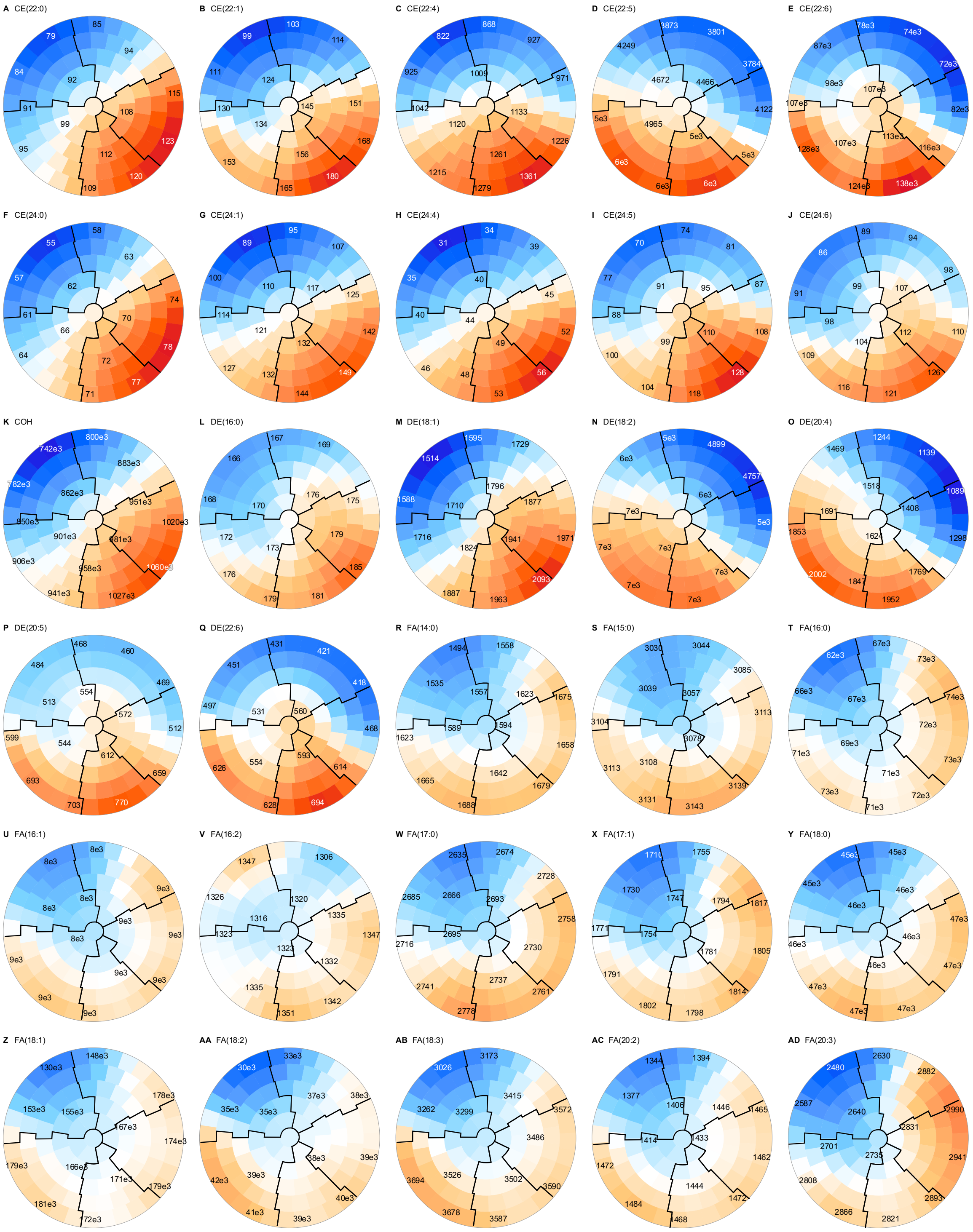
A CE(22:0)
B CE(22:1)
C CE(22:4)
D CE(22:5)
E CE(22:6)
F CE(24:0)
G CE(24:1)
H CE(24:4)
I CE(24:5)
J CE(24:6)
K COH
L DE(16:0)
M DE(18:1)
N DE(18:2)
O DE(20:4)
P DE(20:5)
Q DE(22:6)
R FA(14:0)
S FA(15:0)
T FA(16:0)
U FA(16:1)
V FA(16:2)
W FA(17:0)
X FA(17:1)
Y FA(18:0)
Z FA(18:1)
AA FA(18:2)
AB FA(18:3)
AC FA(20:2)
AD FA(20:3)

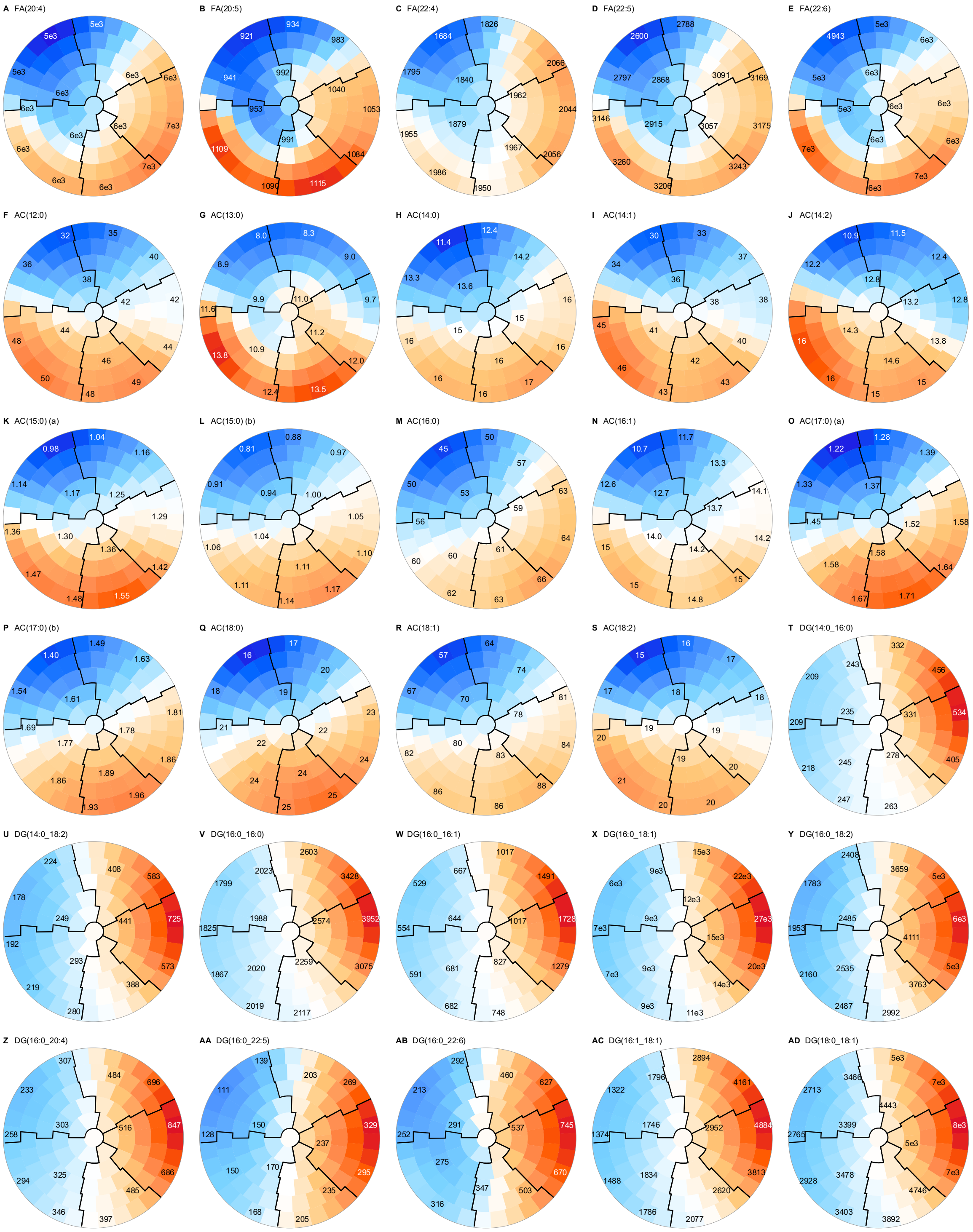
A FA(20:4)
B FA(20:5)
C FA(22:4)
D FA(22:5)
E FA(22:6)
F AC(12:0)
G AC(13:0)
H AC(14:0)
I AC(14:1)
J AC(14:2)
K AC(15:0) (a)
L AC(15:0) (b)
M AC(16:0)
N AC(16:1)
O AC(17:0) (a)
P AC(17:0) (b)
Q AC(18:0)
R AC(18:1)
S AC(18:2)
T DG(14:0_16:0)
U DG(14:0_18:2)
V DG(16:0_16:0)
W DG(16:0_16:1)
X DG(16:0_18:1)
Y DG(16:0_18:2)
Z DG(16:0_20:4)
AA DG(16:0_22:5)
AB DG(16:0_22:6)
AC DG(16:1_18:1)
AD DG(18:0_18:1)

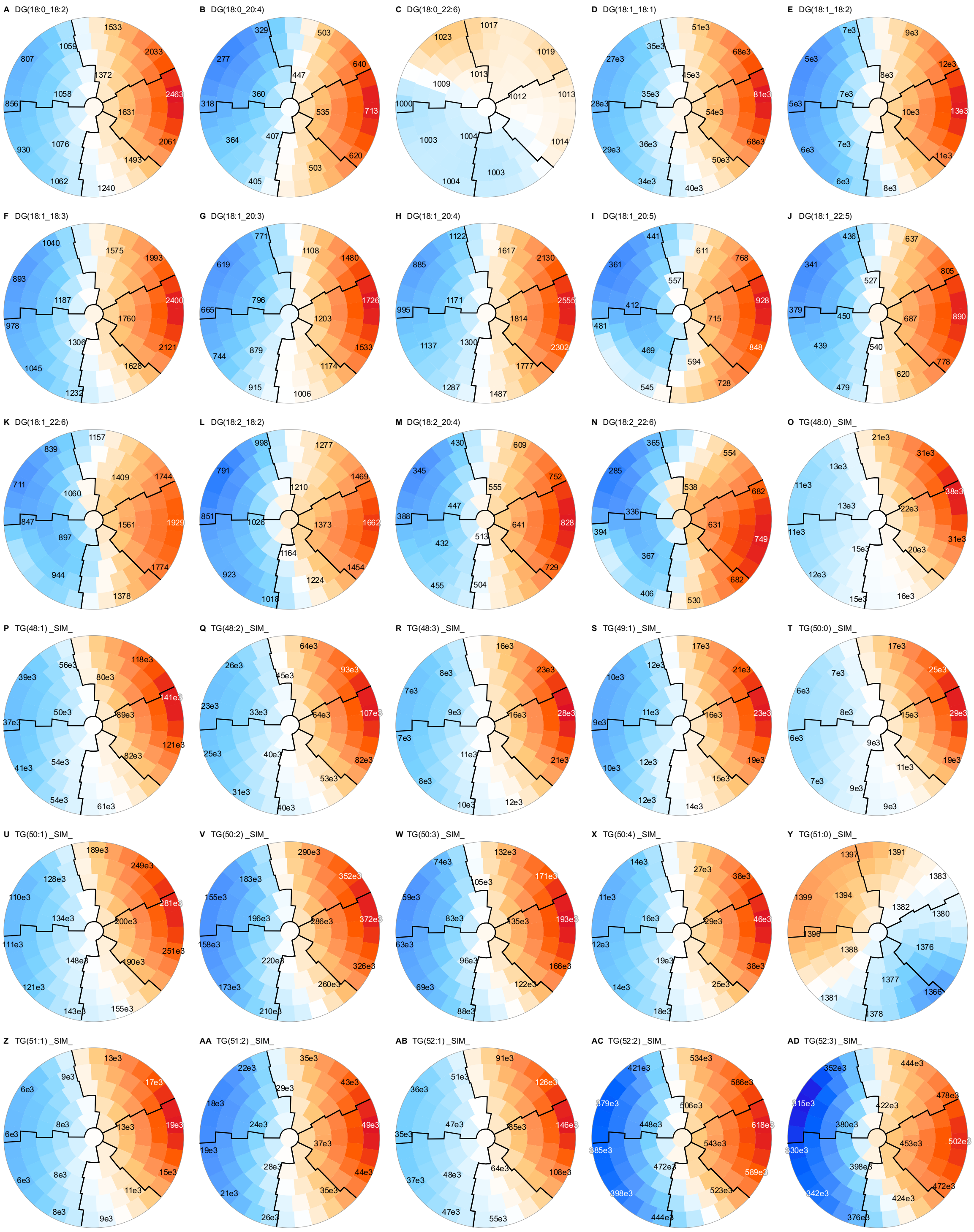
A DG(18:0_18:2)
B DG(18:0_20:4)
C DG(18:0_22:6)
D DG(18:1_18:1)
E DG(18:1_18:2)
F DG(18:1_18:3)
G DG(18:1_20:3)
H DG(18:1_20:4)
I DG(18:1_20:5)
J DG(18:1_22:5)
K DG(18:1_22:6)
L DG(18:2_18:2)
M DG(18:2_20:4)
N DG(18:2_22:6)
O TG(48:0) _SIM_
P TG(48:1) _SIM_
Q TG(48:2) _SIM_
R TG(48:3) _SIM_
S TG(49:1) _SIM_
T TG(50:0) _SIM_
U TG(50:1) _SIM_
V TG(50:2) _SIM_
W TG(50:3) _SIM_
X TG(50:4) _SIM_
Y TG(51:0) _SIM_
Z TG(51:1) _SIM_
AA TG(51:2) _SIM_
AB TG(52:1) _SIM_
AC TG(52:2) _SIM_
AD TG(52:3) _SIM_

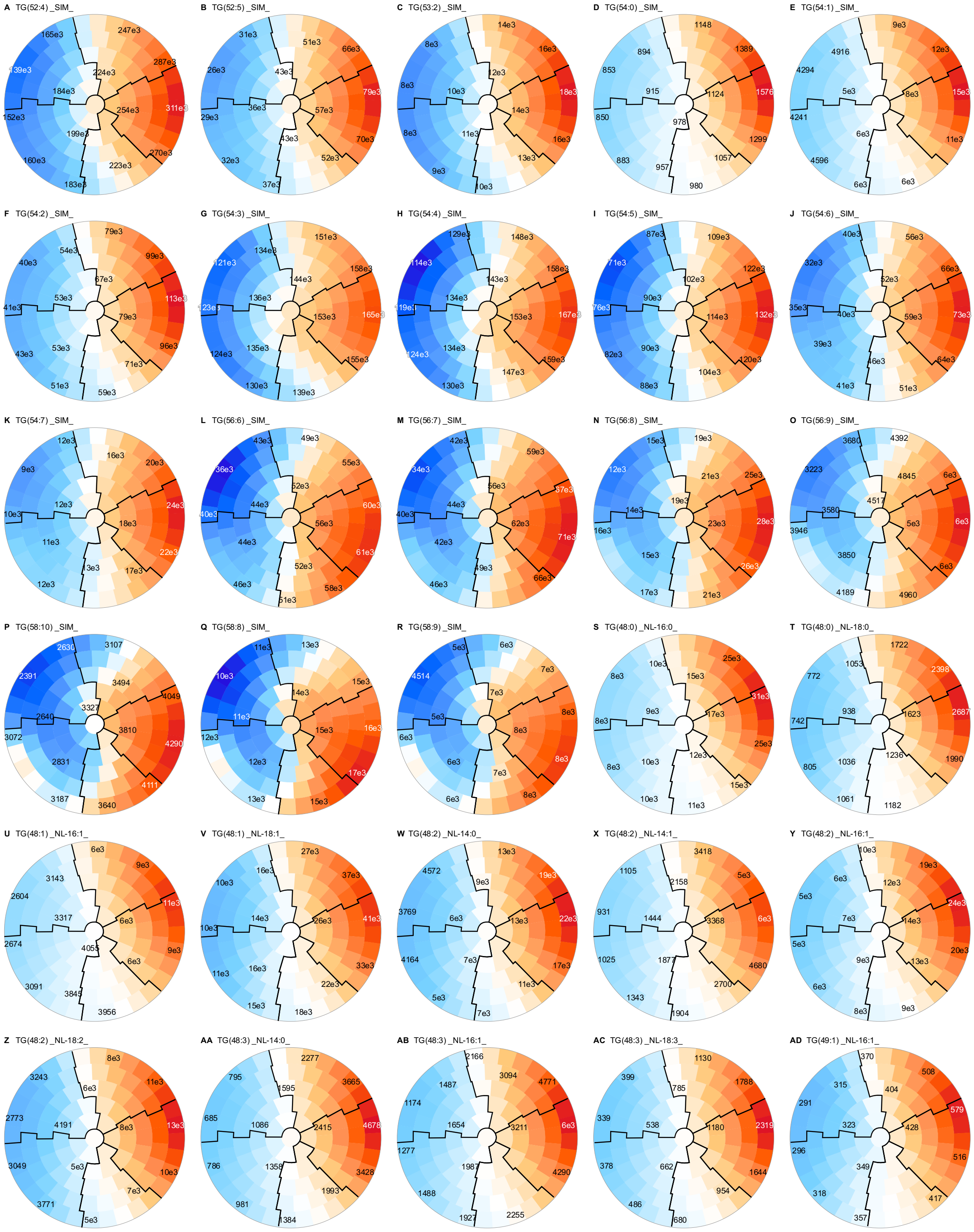
A TG(52:4) _SIM_
B TG(52:5) _SIM_
C TG(53:2) _SIM_
D TG(54:0) _SIM_
E TG(54:1) _SIM_
F TG(54:2) _SIM_
G TG(54:3) _SIM_
H TG(54:4) _SIM_
I TG(54:5) _SIM_
J TG(54:6) _SIM_
K TG(54:7) _SIM_
L TG(56:6) _SIM_
M TG(56:7) _SIM_
N TG(56:8) _SIM_
O TG(56:9) _SIM_
P TG(58:10) _SIM_
Q TG(58:8) _SIM_
R TG(58:9) _SIM_
S TG(48:0) _NL-16:0_
T TG(48:0) _NL-18:0_
U TG(48:1) _NL-16:1_
V TG(48:1) _NL-18:1_
W TG(48:2) _NL-14:0_
X TG(48:2) _NL-14:1_
Y TG(48:2) _NL-16:1_
Z TG(48:2) _NL-18:2_
AA TG(48:3) _NL-14:0_
AB TG(48:3) _NL-16:1_
AC TG(48:3) _NL-18:3_
AD TG(49:1) _NL-16:1_

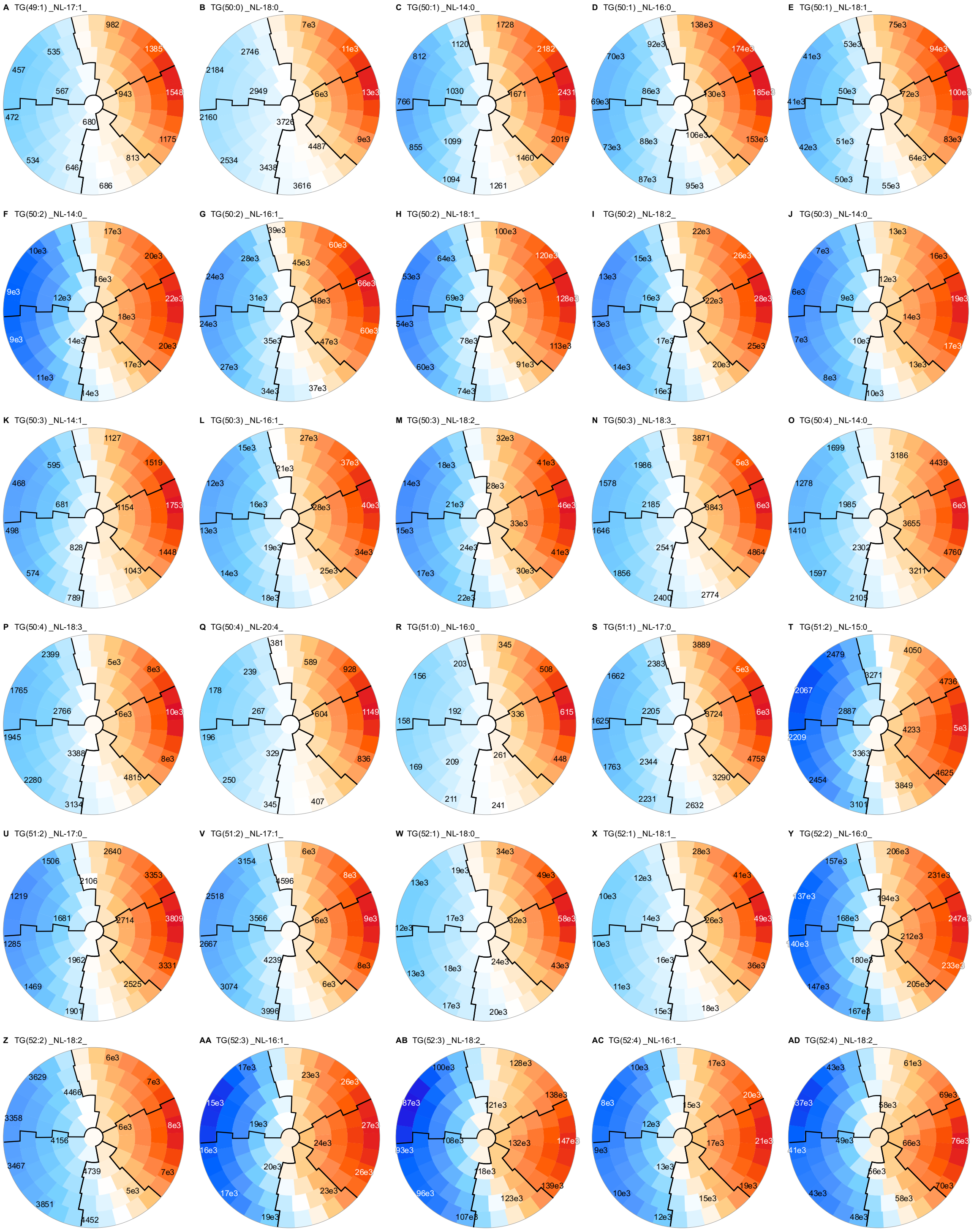
A TG(49:1) _NL-17:1_
982 1385 1548 943 1175 813 686 646 680 534 472 457 567 535
B TG(50:0) _NL-18:0_
7e3 11e3 13e3 6e3 9e3 4487 3616 3438 3726 2534 2160 2184 2949 2746
C TG(50:1) _NL-14:0_
1728 2182 2431 1671 2019 1460 1261 1094 1099 855 766 812 1030 1120
D TG(50:1) _NL-16:0_
138e3 174e3 185e3 130e3 153e3 106e3 95e3 87e3 88e3 73e3 69e3 70e3 86e3 92e3
E TG(50:1) _NL-18:1_
75e3 94e3 100e3 72e3 83e3 64e3 55e3 50e3 51e3 42e3 41e3 41e3 50e3 53e3
F TG(50:2) _NL-14:0_
17e3 20e3 22e3 16e3 18e3 20e3 17e3 14e3 14e3 11e3 9e3 9e3 12e3 10e3
G TG(50:2) _NL-16:1_
39e3 60e3 66e3 45e3 48e3 60e3 47e3 37e3 34e3 35e3 27e3 24e3 24e3 31e3 28e3
H TG(50:2) _NL-18:1_
100e3 120e3 128e3 99e3 113e3 91e3 74e3 78e3 60e3 54e3 53e3 69e3 64e3
I TG(50:2) _NL-18:2_
22e3 26e3 28e3 22e3 25e3 20e3 16e3 17e3 14e3 13e3 13e3 16e3 15e3
J TG(50:3) _NL-14:0_
13e3 16e3 19e3 12e3 14e3 17e3 13e3 10e3 10e3 8e3 7e3 6e3 9e3 7e3
K TG(50:3) _NL-14:1_
1127 1519 1753 1154 1448 1043 789 828 574 498 468 681 595
L TG(50:3) _NL-16:1_
27e3 37e3 40e3 21e3 28e3 34e3 25e3 18e3 19e3 14e3 13e3 12e3 16e3 15e3
M TG(50:3) _NL-18:2_
32e3 41e3 46e3 28e3 33e3 41e3 30e3 22e3 24e3 17e3 15e3 14e3 21e3 18e3
N TG(50:3) _NL-18:3_
3871 5e3 6e3 3843 4864 2774 2400 2541 1856 1646 1578 2185 1986
O TG(50:4) _NL-14:0_
3186 4439 6e3 3655 4760 3211 2105 2302 1597 1410 1278 1985 1699
P TG(50:4) _NL-18:3_
5e3 8e3 10e3 6e3 8e3 4815 3134 3388 2280 1945 1765 2766 2399
Q TG(50:4) _NL-20:4_
381 589 928 1149 604 836 407 345 329 250 196 178 267 239
R TG(51:0) _NL-16:0_
345 508 615 336 448 261 241 211 209 169 158 156 192 203
S TG(51:1) _NL-17:0_
3889 5e3 6e3 3724 4758 3290 2632 2231 2344 1763 1625 1662 2205 2383
T TG(51:2) _NL-15:0_
4050 4736 5e3 3271 4233 4625 3849 3101 3363 2454 2209 2067 2887 2479
U TG(51:2) _NL-17:0_
2640 3353 3809 2106 2714 3331 2525 1901 1962 1469 1285 1219 1681 1506
V TG(51:2) _NL-17:1_
6e3 8e3 9e3 4596 6e3 8e3 6e3 3996 4239 3074 2667 2518 3566 3154
W TG(52:1) _NL-18:0_
34e3 49e3 58e3 19e3 32e3 43e3 24e3 20e3 17e3 18e3 13e3 12e3 13e3 17e3
X TG(52:1) _NL-18:1_
28e3 41e3 49e3 26e3 36e3 18e3 15e3 16e3 11e3 10e3 10e3 14e3 12e3
Y TG(52:2) _NL-16:0_
206e3 231e3 247e3 194e3 212e3 233e3 205e3 167e3 180e3 147e3 140e3 137e3 168e3 157e3
Z TG(52:2) _NL-18:2_
6e3 7e3 8e3 6e3 7e3 5e3 4452 4739 3851 3467 3358 4156 4466 3629
AA TG(52:3) _NL-16:1_
23e3 26e3 27e3 24e3 26e3 23e3 19e3 20e3 17e3 16e3 15e3 19e3 17e3
AB TG(52:3) _NL-18:2_
128e3 138e3 147e3 121e3 132e3 139e3 123e3 107e3 118e3 96e3 93e3 87e3 108e3 100e3
AC TG(52:4) _NL-16:1_
17e3 20e3 21e3 15e3 17e3 19e3 15e3 12e3 13e3 10e3 9e3 8e3 12e3 10e3
AD TG(52:4) _NL-18:2_
61e3 69e3 76e3 58e3 66e3 70e3 58e3 48e3 56e3 43e3 41e3 37e3 49e3 43e3

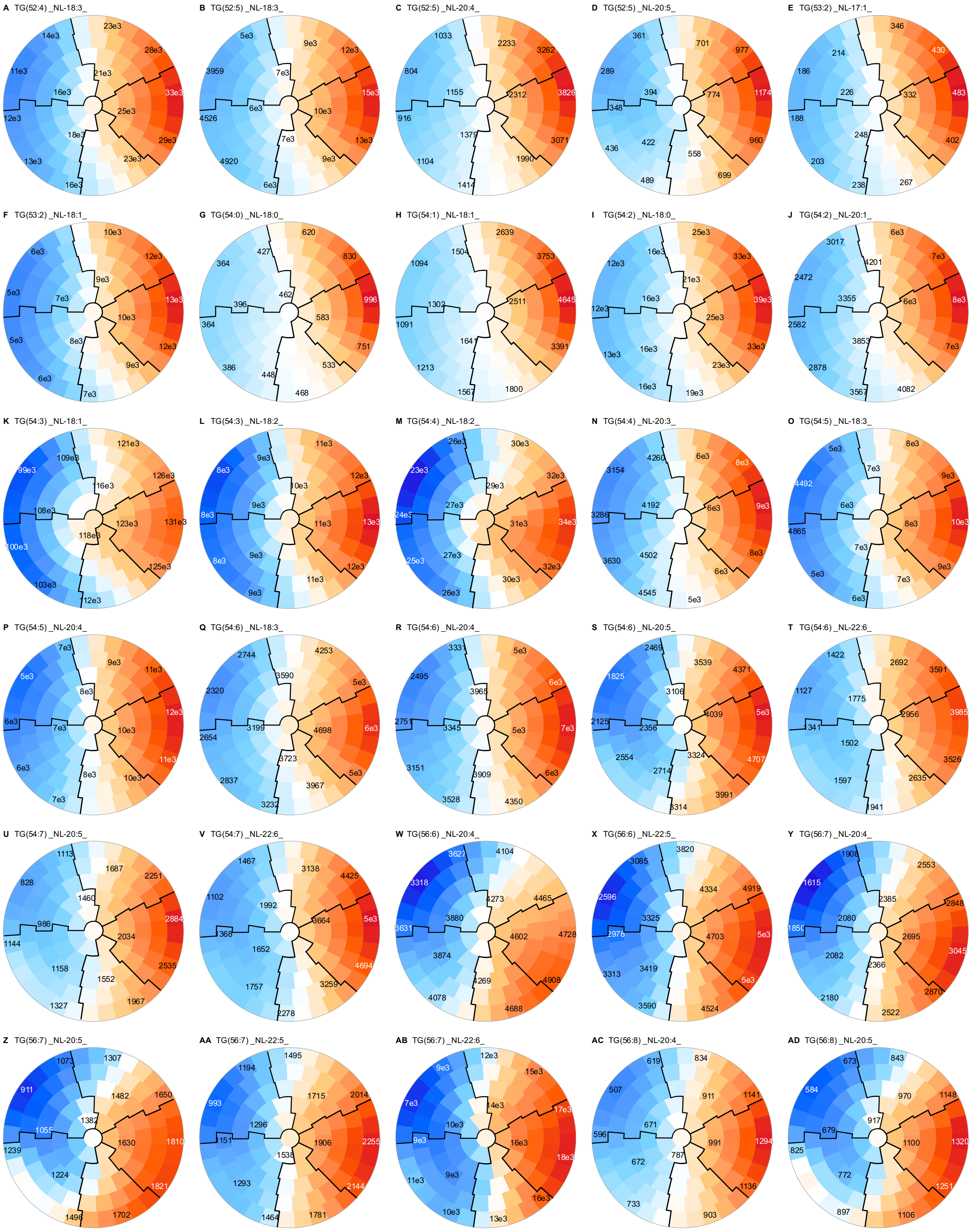
A TG(52:4) _NL-18:3_
B TG(52:5) _NL-18:3_
C TG(52:5) _NL-20:4_
D TG(52:5) _NL-20:5_
E TG(53:2) _NL-17:1_
F TG(53:2) _NL-18:1_
G TG(54:0) _NL-18:0_
H TG(54:1) _NL-18:1_
I TG(54:2) _NL-18:0_
J TG(54:2) _NL-20:1_
K TG(54:3) _NL-18:1_
L TG(54:3) _NL-18:2_
M TG(54:4) _NL-18:2_
N TG(54:4) _NL-20:3_
O TG(54:5) _NL-18:3_
P TG(54:5) _NL-20:4_
Q TG(54:6) _NL-18:3_
R TG(54:6) _NL-20:4_
S TG(54:6) _NL-20:5_
T TG(54:6) _NL-22:6_
U TG(54:7) _NL-20:5_
V TG(54:7) _NL-22:6_
W TG(56:6) _NL-20:4_
X TG(56:6) _NL-22:5_
Y TG(56:7) _NL-20:4_
Z TG(56:7) _NL-20:5_
AA TG(56:7) _NL-22:5_
AB TG(56:7) _NL-22:6_
AC TG(56:8) _NL-20:4_
AD TG(56:8) _NL-20:5_

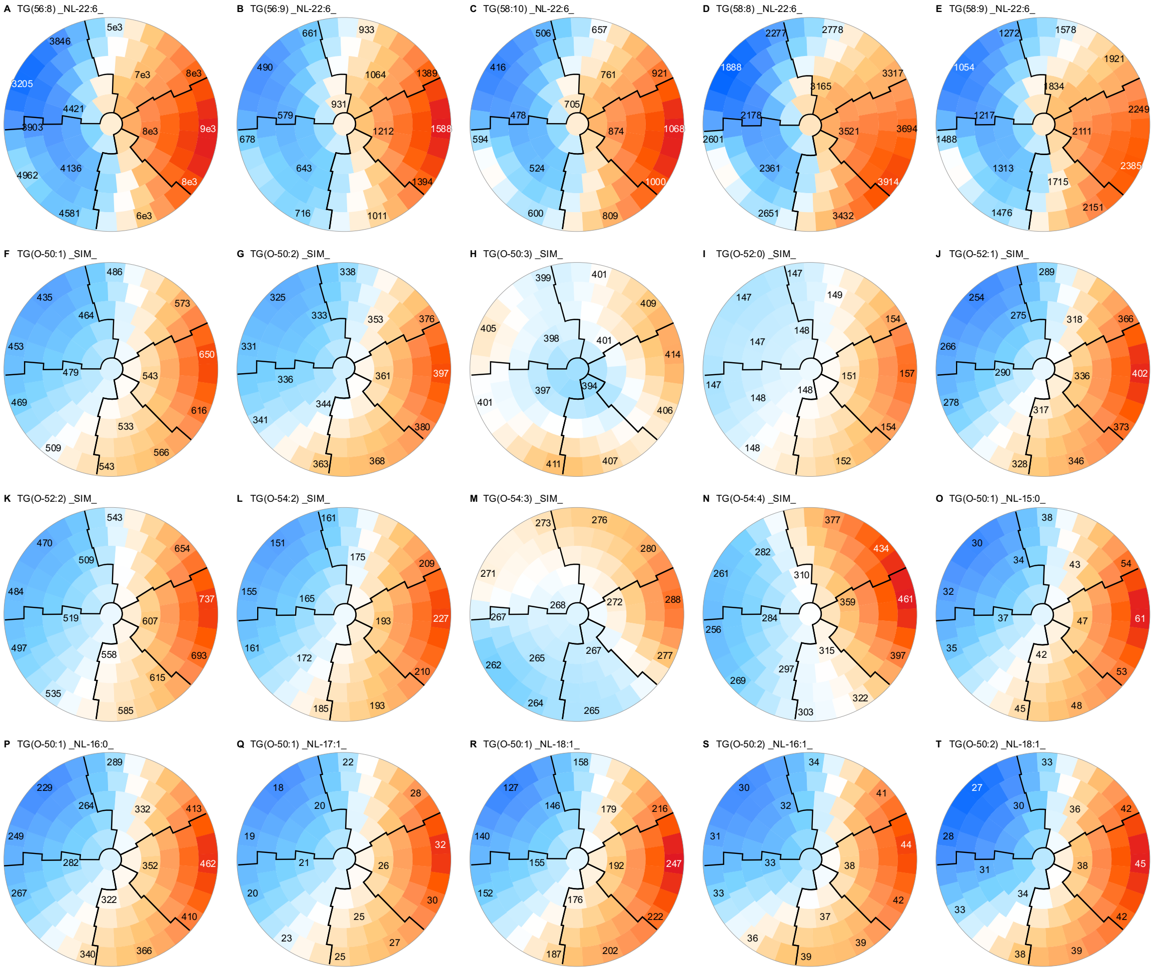
A TG(56:8) _NL-22:6_
B TG(56:9) _NL-22:6_
C TG(58:10) _NL-22:6_
D TG(58:8) _NL-22:6_
E TG(58:9) _NL-22:6_
F TG(O-50:1) _SIM_
G TG(O-50:2) _SIM_
H TG(O-50:3) _SIM_
I TG(O-52:0) _SIM_
J TG(O-52:1) _SIM_
K TG(O-52:2) _SIM_
L TG(O-54:2) _SIM_
M TG(O-54:3) _SIM_
N TG(O-54:4) _SIM_
O TG(O-50:1) _NL-15:0_
P TG(O-50:1) _NL-16:0_
Q TG(O-50:1) _NL-17:1_
R TG(O-50:1) _NL-18:1_
S TG(O-50:2) _NL-16:1_
T TG(O-50:2) _NL-18:1_

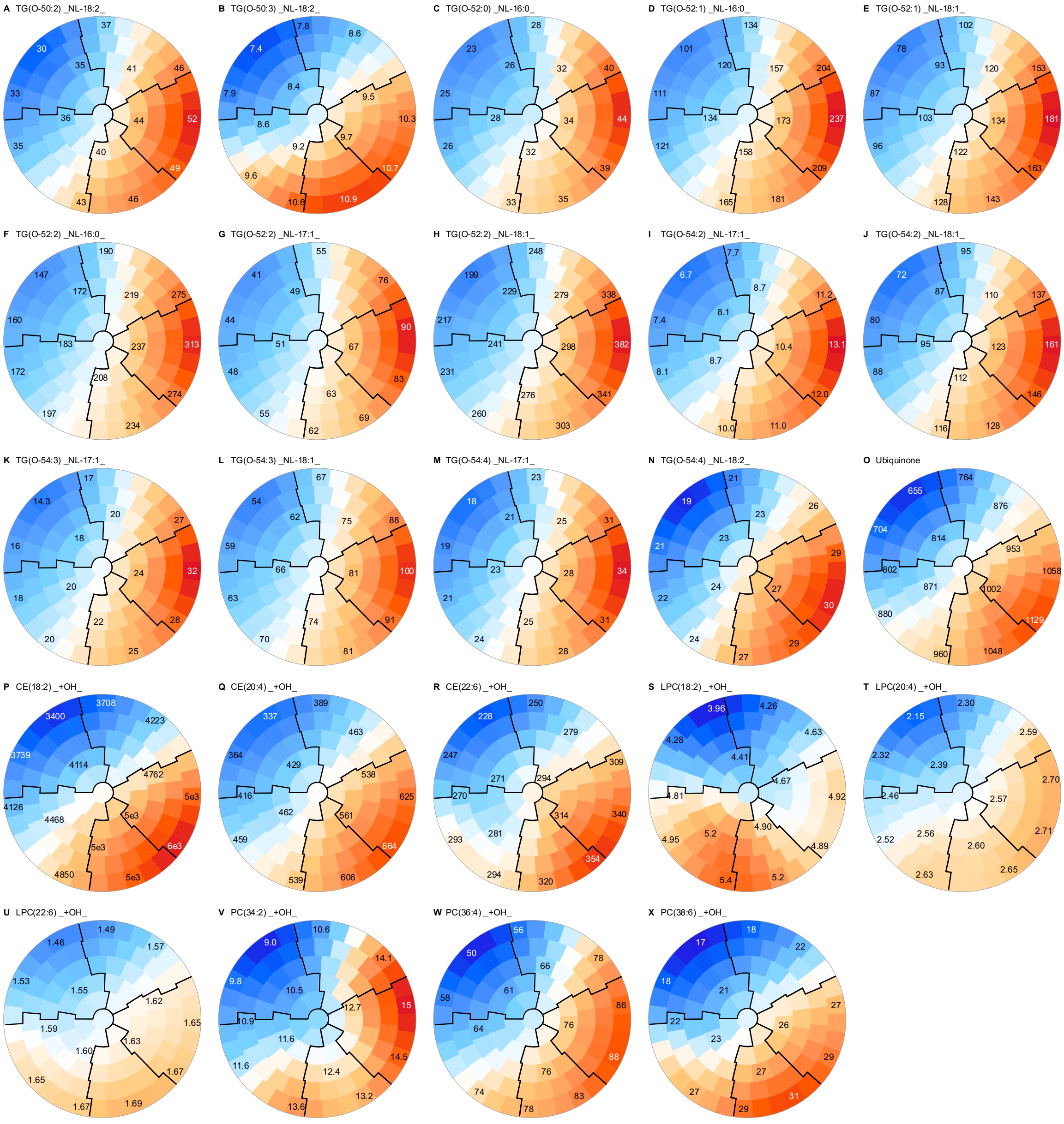
A TG(O-50:2) _NL-18:2_
B TG(O-50:3) _NL-18:2_
C TG(O-52:0) _NL-16:0_
D TG(O-52:1) _NL-16:0_
E TG(O-52:1) _NL-18:1_
F TG(O-52:2) _NL-16:0_
G TG(O-52:2) _NL-17:1_
H TG(O-52:2) _NL-18:1_
I TG(O-54:2) _NL-17:1_
J TG(O-54:2) _NL-18:1_
K TG(O-54:3) _NL-17:1_
L TG(O-54:3) _NL-18:1_
M TG(O-54:4) _NL-17:1_
N TG(O-54:4) _NL-18:2_
O Ubiquinone
P CE(18:2) _+OH_
Q CE(20:4) _+OH_
R CE(22:6) _+OH_
S LPC(18:2) _+OH_
T LPC(20:4) _+OH_
U LPC(22:6) _+OH_
V PC(34:2) _+OH_
W PC(36:4) _+OH_
X PC(38:6) _+OH_
